# *AMBN* mutations causing hypoplastic amelogenesis imperfecta and *Ambn* knockout‐NLS‐lacZ knockin mice exhibiting failed amelogenesis and *Ambn* tissue‐specificity

**DOI:** 10.1002/mgg3.929

**Published:** 2019-08-11

**Authors:** Tian Liang, Yuanyuan Hu, Charles E. Smith, Amelia S Richardson, Hong Zhang, Jie Yang, Brent Lin, Shih‐Kai Wang, Jung‐Wook Kim, Yong‐Hee Chun, James P. Simmer, Jan C.‐C. Hu

**Affiliations:** ^1^ Department of Biologic and Materials Sciences University of Michigan School of Dentistry Ann Arbor Michigan; ^2^ Department of Anatomy and Cell Biology, Faculty of Medicine McGill University Montreal Quebec Canada; ^3^ Department of Pediatric Dentistry, School and Hospital of Stomatology Peking University Beijing China; ^4^ Department of Orofacial Sciences UCSF School of Dentistry San Francisco California; ^5^ Department of Dentistry National Taiwan University School of Dentistry Taipei City Taiwan R.O.C; ^6^ Department of Molecular Genetics and Department of Pediatric Dentistry & Dental Research Institute, School of Dentistry Seoul National University Seoul Korea; ^7^ Department of Periodontics and Department of Cell Systems & Anatomy, School of Dentistry University of Texas Health Science Center at San Antonio San Antonio Texas

**Keywords:** *Ambn*^‐/‐^*Amelx*^‐/-^, amelin, ameloblastin, amelogenin, dental enamel formation, matrix proteins, mineralization, missense mutation, sheath protein, sheathlin

## Abstract

**Background:**

Ameloblastin (AMBN) is a secreted matrix protein that is critical for the formation of dental enamel and is enamel‐specific with respect to its essential functions. Biallelic *AMBN* defects cause non‐syndromic autosomal recessive amelogenesis imperfecta. Homozygous *Ambn* mutant mice expressing an internally truncated AMBN protein deposit only a soft mineral crust on the surface of dentin.

**Methods:**

We characterized a family with hypoplastic amelogenesis imperfecta caused by *AMBN* compound heterozygous mutations (c.1061T>C; p.Leu354Pro/ c.1340C>T; p.Pro447Leu). We generated and characterized *Ambn* knockout/NLS‐lacZ (*Ambn* ^lacZ/lacZ^) knockin mice.

**Results:**

No AMBN protein was detected using immunohistochemistry in null mice. ß‐galactosidase activity was specific for ameloblasts in incisors and molars, and islands of cells along developing molar roots. *Ambn* ^lacZ/lacZ^ 7‐week incisors and unerupted (D14) first molars showed extreme enamel surface roughness. No abnormalities were observed in dentin mineralization or in nondental tissues. Ameloblasts in the *Ambn* ^lacZ/lacZ^ mice were unable to initiate appositional growth and started to degenerate and deposit ectopic mineral. No layer of initial enamel ribbons formed in the *Ambn* ^lacZ/lacZ^ mice, but pockets of amelogenin accumulated on the dentin surface along the ameloblast distal membrane and within the enamel organ epithelia (EOE). NLS‐lacZ signal was positive in the epididymis and nasal epithelium, but negative in ovary, oviduct, uterus, prostate, seminal vesicles, testis, submandibular salivary gland, kidney, liver, bladder, and bone, even after 15 hr of incubation with X‐gal.

**Conclusions:**

Ameloblastin is critical for the initiation of enamel ribbon formation, and its absence results in pathological mineralization within the enamel organ epithelia.

## 1 INTRODUCTION

Ameloblastin (AMBN) (OMIM *601259) is a phosphorylated glycoprotein secreted by ameloblasts into the enamel matrix of developing teeth (Fincham, Moradian‐Oldak, & Simmer, 1999). AMBN is required for enamel formation, as defects in *AMBN* cause non‐syndromic inherited enamel malformations in humans (Poulter et al., 2014; Prasad et al., 2016) and in *Ambn* defective mice (Fukumoto et al., 2004). An intact *Ambn* is consistently found in vertebrates that make enamel, while *Ambn* is pseudogenized in mammals that have lost the ability to make teeth, such as baleen whales (Mysticeti; Demere, McGowen, Berta, & Gatesy, 2008; Meredith, Gatesy, Cheng, & Springer, 2011), turtles (Meredith, Gatesy, & Springer, 2013), birds (Meredith et al., 2013), narwhal (Meredith et al., 2013), aardvark (Meredith et al., 2013), and two‐toed sloth (Meredith et al., 2013). Other tooth‐specific genes implicated in the etiology of non‐syndromic amelogenesis imperfecta in humans, including *ENAM*, *AMEL*, *MMP20*, *ODAPH*, *KLK4*, and *AMTN* (Springer et al., 2016), have been shown to be inactivated in one or more toothless vertebrates and/or in vertebrates with enamel‐less teeth.

The spotted gar (*Lepisosteus oculatus*) is among the most distant extant relatives of tetrapods that make enamel (Sire, 1994, 1995). In the gar, enamel (also called ganoin) forms on both its teeth and scales, and *Ambn* expression is specifically associated with the formation of these mineralized organs, and is not expressed in gar bone, embryos, or any of the nine organs included in the PhyloFish database (Braasch et al., 2016; Kawasaki et al., 2017). AMBN is a tooth‐specific gene that is required for amelogenesis, although there are some reports that AMBN functions in other tissues, such as bone (Atsawasuwan et al., 2013; Jacques et al., 2014). These claims are inconsistent with (1) the absence of AMBN expressed sequence tags (EST) from bone in the human (Hs.272396; 0/71618) and mouse (Mm.8437; 0/34066) EST databases, (2) *Ambn* pseudogenization in mammals that continue to make bone but not enamel, and (3) the absence of reported bone phenotypes in persons with amelogenesis imperfecta (AI) caused by biallelic *AMBN* defects.

### 1.1 Discovery of Ameloblastin and proteolysis by MMP20

AMBN was first discovered by the characterization of its cleavage products in developing pig enamel. AMBN cleavage products were purified from enamel scraped from developing pig teeth, characterized using N‐terminal sequencing and amino acid composition analyses, and used to raise specific polyclonal antibodies (Fukae & Tanabe, 1987a, 1987b; Fukae, Tanabe, Uchida, Yamakoshi, & Shimizu, 1993; Murakami et al., 1997; Shimizu, 1984). Immunohistochemistry of developing pig enamel using these antibodies outlined the enamel rods, producing a honeycomb pattern throughout the inner and middle enamel layers (Uchida et al., 1991), leading to its designation as “sheath protein” (Uchida et al., 1995), although the concept of an organic sheath surrounding the rods and cementing them together had been previously dismissed (Orams, 1964, 1966). Rat and pig cDNA clones encoding the “sheath protein” were cloned independently from three groups and labeled “ameloblastin” (Krebsbach et al., 1996), “amelin” (Cerny, Slaby, Hammarstrom, & Wurtz, 1996), and “sheathlin” (Hu et al., 1997). With ameloblastin being the first cDNA cloning manuscript to be submitted and published, it became the formal and accepted designation. The N‐terminal sequences of six different non‐amelogenin peptides isolated from fresh secretory stage pig enamel were identified within the AMBN deduced amino acid sequences. MMP20 catalyzed the cleavage of recombinant AMBN in vitro at the exact same positions as were shown to occur in vivo (Chun et al., 2010; Iwata et al., 2007). Similar work had previously demonstrated that MMP20 also cleaves amelogenin (AMEL) in vitro at the same sites known to be cleaved in vivo through the isolation and characterization of matrix proteins (Ryu et al., 1999). AMBN is expressed and secreted by ameloblasts (Lee et al., 1996), and in mouse enamel concentrates near the secretory surface of the ameloblast (Nanci et al., 1998).

### 1.2 *Ambn* gene structure

Alternative splicing results in the secretion of two ameloblastin isoforms. Transcript 1 includes (while transcript 2 omits) a 45 nucleotide segment encoding 15 amino acids at the 5' end of Exon 6 (Cerny et al., 1996; Hu et al., 1997). Alignment of 53 functional AMBN sequences, covering over 200 million years (MY) of evolution, identified 80 unchanged and 81 highly conserved amino acid residues (out of 447 residues in the human AMBN protein, inclusive of the signal peptide; Delsuc, Gasse, & Sire, 2015). All 15 amino acids encoded by the 5'‐end of Exon 6 that can be deleted during RNA splicing are highly or absolutely conserved, and this peptide also includes an O‐glycosylation (human secreted protein Ser^112^), suggesting that there may be an important functional difference between the two ameloblastin isoforms generated by alternative splicing (Kobayashi et al., 2007). Evolutionary analysis suggested that ancestral mammalian *AMBN* likely possessed 11 exons (corresponding to exons 1 to 7 and 10 to 13 in humans, as Exons 8 and 9 were added in the human line by tandem duplications of Exon 7; Delsuc et al., 2015). All *AMBN* introns are phase 0 (located between complete codons), so duplication and deletion of exons during evolution, and alternative mRNA splicing, does not shift the downstream reading frame. Virtually all introns in secretory calcium‐binding phosphoprotein genes (SCPPs), including *AMBN*, are phase 0 (Kawasaki et al., 2017).

A potentially important *AMBN* structural feature is the presence of two in‐frame, putative translational start sites (TSS; Hu et al., 1997). The 3' TSS (in Exon 2) is in a much better context for translation initiation, having purines in both the −3 and +4 positions, whereas the 5' TSS (in Exon 1) has pyrimidines in both of these positions (Hinnebusch, 2017; Kozak, 1984). Phylogenetic analyses showed that the TSS in Exon 2 is conserved in all *AMBN* analyzed, while the TSS in Exon 1 appeared recently, in the eutherian (placental mammal) ancestor and was independently lost in various eutherian species (Delsuc et al., 2015). Ribosomes scanning the mRNA from the 5’ end usually skip over the first weak TSS to initiate more frequently at the strong TSS (leaky scanning; Hinnebusch, Ivanov, & Sonenberg, 2016). Initiation from the more 5' TSS (in Exon 1) would add 10 amino acids to the beginning of the signal peptide compared with translation initiation from the more 3' TSS (in Exon 2), with use of either translation start site generating the same secreted protein.

### 1.3 Etiology of human amelogenesis imperfecta

Although *AMBN* has long been a candidate gene for the etiology of amelogenesis imperfecta (AI), only two AI‐causing *AMBN* defects have been reported to date (Figure S2). Both are homozygous recessive mutations, and both are predicted to delete a single exon. The first was a 2,347 bp deletion within *AMBN* (c.294+139_531+478del; p.Tyr99_Glu177del) inclusive of Exon 6 (Poulter et al., 2014). The second was a splice‐junction mutation at the end of Intron 6 (c.532‐1G>C; p.Leu178_Ser190del) that was predicted to delete Exon 7 (Prasad et al., 2016). Both of these *AMBN* defects were associated with a non‐syndromic hypoplastic AI (thin enamel) phenotype. Recently an *AMBN* single allele mutation (NM_016519.5: c.1069C>T; p.Pro357Ser) was reported to cause a dominant condition with both enamel and dentin defects (Lu et al., 2018), but the clinical phenotype exactly matched that of dentinogenesis imperfecta, an autosomal dominant condition caused by mutations in *DSPP*, a gene linked to *AMBN* on chromosome 4 that contains a large repetitive region that cannot be characterized using whole exome sequence analyses (Yang et al., 2016).

### 1.4 *Ambn* mutant mice

Generating a complete knockout of genes belonging to the secretory calcium‐binding phosphoprotein (SCPP) family (which includes enamel‐specific *AMBN*, *AMELX*, and *ENAM*) is complicated because of the ubiquitous phase‐0 introns. Because of them, alternative splicing always maintains the reading frame and potentially generates internally truncated proteins (Kawasaki & Weiss, 2003). Previously the *Amelx^−/−^* mice were generated by deleting only Exon 2, inclusive of the translation initiation codon (Gibson et al., 2001). However, reverse transcription polymerase chain reaction (RT‐PCR) helped detect *Amelx* mRNA transcripts lacking only the 69 nucleotides encoded by Exon 2 (including the translation initiation codon) in the *Amelx^−^* ^/^ *^−^* mice. Dental enamel in the *Amelx^−^* ^/^ *^−^* mice was dramatically malformed, and no AMEL protein was detected on Western blots using multiple antibodies, so clearly no AMEL was being translated from knockout transcripts lacking Exon 2 (Gibson et al., 2001). An *Ambn* “knockout” mouse was generated by deleting Exons 5 and 6 and resulted in significant enamel malformations (Fukumoto et al., 2004). *Ambn* transcripts from this mouse were not detected using RT‐PCR, and ameloblastin protein was not detected using immunohistochemistry (IHC; Fukumoto et al., 2004). Ameloblasts in this knockout mouse differentiated but soon detached from the matrix surface, lost cell polarity, resumed proliferation to form multiple cell layers, and deposited a thin, hypocalcified, unstructured matrix above the dentin surface (Fukumoto et al., 2004). *Amelx* mRNA was reported to be downregulated, and about 25% of the homozygous mice developed soft tissue tumors in the buccal vestibules of the oral cavity starting at 26 weeks (Fukumoto et al., 2004; Fukumoto, Yamada, Nonaka, & Yamada, 2005). However, a subsequent study of this mouse discovered that a shorter *Ambn* mRNA that only lacked exons 5 and 6 was expressed (Wazen, Moffatt, Zalzal, Yamada, & Nanci, 2009). The mutant transcript maintained the *Ambn* reading frame and replaced the two wild‐type 381 and 396 amino acid secreted AMBN isoforms with a single secreted 279 amino acid internally truncated AMBN protein lacking the Tyr^41^‐Glu^157^ segment encoded by Exons 5 and 6. The aberrant AMBN protein was detected on Western blot and using immunohistochemistry using antibodies raised against amino acids encoded by Exons 8 to 11 (Smith et al., 2011; Wazen et al., 2009). They redesignated this knockin mouse *Ambn* ^Δ5,6/Δ5,6^. As the mutant protein might be partially functional or toxic, important additional information about AMBN function could be gained by characterization of a true *Ambn* null mouse.

To gain a better understanding of *Ambn* tissue‐specific expression and to improve our understanding of amelogenesis and the role played by AMBN in it, we have generated and extensively characterized a new *Ambn* knockout/NLS‐lacZ knockin mouse, which we alternately refer to as *Ambn* ^lacZ/lacZ^ or *Ambn* ^−/−^. We also report a novel *AMBN* mutation that causes AI in humans.

## 2 MATERIALS AND METHODS

### 2.1 Enrollment of human subjects

This study was reviewed and approved by the Institutional Review Boards at the University of Texas Health Science Center at San Antonio and the University of Michigan. Subject enrollment, clinical examinations, and collection of saliva samples were performed with the understanding and written consent of each participant in compliance with the Declaration of Helsinki, described previously (Chan et al., 2011; Kim et al., 2006).

### 2.2 Whole exome sequencing and bioinformatics analyses

A Hispanic family with hypoplastic enamel trait was recruited. Mother, proband, proband's half‐sister, her husband, and their daughter participated in the study and provided samples. Subject sample preparation, whole exome sequencing, variant annotation, and mutational analyses were conducted based upon established protocols (Zhang et al., 2019). The *ABMN* mutations were identified by screening of 42 known AI candidate genes (Table S1).

### 2.3 Generation of *Ambn* ^lacZ/lacZ^ mice

The *Ambn* knockin construct was designed, fabricated, and used to establish germline *Ambn* knockout/NLS‐lacZ knockin mice (*Ambn* ^lacZ^) by Ozgene (Bentley DC, Western Australia, Australia). The *Ambn* ^lacZ^ mice were crossed with C57BL/6 mice for at least eight generations. All mice were maintained on a soft diet (DietGel, ClearH2O), as described previously (Simmer, Hu, Lertlam, Yamakoshi, & Hu, 2009). These mice are available at the Mutant Mouse Resource and Research Center (MMRRC) repository (MMRRC:037503‐UCD).

### 2.4 RT‐PCR analyses *Ambn* ^+^ and *Ambn* ^lacZ^ expression products

RNA was extracted from enamel organ epithelia (EOE) dissected from maxillary and mandibular first molars of D5 *Ambn* ^+/+^, *Ambn* ^+/lacZ^, and *Ambn* ^lacZ/lacZ^ mice. Reverse transcription was performed using the Invitrogen SuperScript III First‐Strand Synthesis System, and polymerase chain reaction (PCR) was conducted using Invitrogen Platinum Hot Start PCR Master Mix (2X), according to the manufacturer's instructions (Invitrogen). The primer sequences are shown in Table S2. The band around 700 bp in *Ambn* ^lacZ/lacZ^ mice amplified by primers in Exons 1 and 11 was isolated from the agarose gel and characterized using DNA sequencing.

### 2.5 Whole‐mount X‐gal staining

D2 and D6 *Ambn* ^+/+^, *Ambn* ^+/lacZ^, and *Ambn* ^lacZ/lacZ^ mice were anesthetized with isoflurane and fixed by cardiac perfusion. Blood was cleared from the vasculature using lactated Ringer's solution (30–45 s) followed by fixation with 4% paraformaldehyde (PFA) in phosphate buffered saline (PBS; 135 mM NaCl, 2.7 mM KCl, 4.3 mM Na_2_HPO_4_, 1.4 mM Na_2_H_2_PO_4_; pH 7.3) for 20 min. Following perfusion, the liver, kidneys, submandibular salivary glands, bladder, prostate, ovaries, oviducts, uterus, testis, seminal vesicle, and epididymis were dissected, immersed in the same fixative for 2–3 hr at 4°C, followed by several washes in PBS. Samples were incubated at 45°C for 5 hr in freshly prepared X‐gal solution (0.1 M HEPES, 1 mM MgCl_2_, 5 mM potassium ferrocyanide, 5 mM potassium ferricyanide, 2% Triton X‐100, 1 mg/ml X‐gal substrate; pH 8.0). After staining, the samples were washed in PBS and photographed using a dissection microscope.

### 2.6 X‐gal staining of histology sections

*Ambn* ^+/+^, *Ambn* ^+/lacZ^, and *Ambn* ^lacZ/lacZ^ mouse heads at D2, D6, D14, and 7 weeks were quickly dissected of skin, cut in half, and fixed by immersion in 4% PFA in PBS overnight at 4°C. The following day, the tissues were washed several times with PBS and decalcified at 4°C in 4.13% disodium ethylenediaminetetraacetic acid (EDTA), pH 7.3. The heads of D2 mice were decalcified for 5 days, D6 mice for 3 weeks, D14 mice for 5 weeks, and 7‐week mice for 7 weeks. Decalcified tissues were immersed in 15% sucrose (1–2 hr) followed by 30% sucrose (3–4 hr) at 4°C for cryoprotection and then embedded in optimal cutting temperature (OCT) compound and stored at −80°C. The blocks were cryosectioned at 8 µm thickness at −20 to −22°C on a Leica cryostat microtome. Slides were stored at −80°C until staining. For X‐gal staining of soft tissues, 7‐week‐old *Ambn* ^+/+^ and *Ambn* ^lacZ/lacZ^ mice were anesthetized with isoflurane and fixed by cardiac perfusion. Blood was cleared from the vasculature using lactated ringer's solution (30–45 s) followed by 4% PFA in PBS for 20 min. Following perfusion, the liver, kidneys, submandibular salivary glands, bladder, prostate, ovaries, oviducts, uterus, testis, seminal vesicle, and epididymis were dissected, immersed in the same fixative for 2–3 hr at 4°C, followed by several washes in PBS. The tissues were then immersed in 15% sucrose (1–2 hr) followed by 30% sucrose (3–4 hr) at 4°C for cryoprotection, embedded in OCT media and stored at −80°C. The blocks were cryosectioned at 8 µm at −20 to −22°C on a Leica cryostat. Slides were stored at −80°C until staining.

Slides were removed from −80°C and immediately treated with glutaraldehyde fixative [0.1 M 4‐(2‐hydroxyethyl)‐1‐piperazineethanesulfonic acid (HEPES), 1.25 mM ethylene glycol tetraacetic acid (EGTA), 2 mM MgCl_2_, 0.5% glutaraldehyde, pH 7.3] and then washed with 0.1 M HEPES, 2 mM MgCl_2_ three times for 5 min. The slides were stained with X‐gal solution (0.1 M HEPES, 1 mM MgCl_2_, 5 mM potassium ferrocyanide, 5 mM potassium ferricyanide, 2% Triton X‐100, 1 mg/ml X‐gal substrate; pH 8.0) for 1 hr at 45°C and then washed several times in PBS, and counterstained with 0.1% (w/v) Nuclear Fast Red, coverslipped with Aqua‐Mount (Thermo Scientific) and imaged using a Nikon Eclipse TE300 inverted microscope. A 1 hr staining time was used because a 5 hr staining period caused significant overstaining in developing teeth and bleeding of the stain beyond the nuclei and into adjacent tissue.

### 2.7 Immunohistochemistry

Slides were baked at 60°C for 1 hr and rehydrated in a gradient ethanol series. Antigen retrieval was performed using the Heat Mediated Antigen Retrieval Solution pH 6.0 (ab973; abcam) following the manufacturer's instructions. Sections were blocked and incubated in primary antibody. The primary antibodies were both polyclonal, raised in rabbits, and used at 1:1000 dilution. The anti‐AMBN antibody was raised against peptide CMRPREHETQQYEYS, which is a remake of the AMBN‐63 antibody (Iwata et al., 2007); the anti‐AMEL antibody was raised against recombinant mouse amelogenin rM179 (Simmer et al., 1994). Sections were incubated in Goat anti‐rabbit IgG(H+L) Secondary Antibody, Alexa Fluor Plus 555 (Invitrogen, A32732) at a concentration of 1:1000, then mounted in Invitrogen^™^ ProLong^™^ Gold Antifade Mountant with DAPI (P36935; Invitrogen). Pictures were taken using a Nikon SP8 confocal microscope system (Biomedical Imaging Core, University of Michigan).

### 2.8 Dissecting microscopy

*Ambn* ^+/+^, *Ambn* ^+/lacZ^, and *Ambn* ^lacZ/lacZ^ mice at 7 weeks were anesthetized with isoflurane for facial photos, sacrificed, and perfused with PBS for 10 min. Their mandibles were denuded of soft tissues, postfixed by immersion in 4% PFA overnight, and rinsed with PBS three times, for 5 min each. The teeth were cleaned with 1% bleach (sodium hypochlorite), rinsed with PBS, air dried, displayed on the Nikon SMZ1000 dissection microscope, and photographed using a Nikon DXM1200 digital camera as described previously (Kim et al., 2019).

### 2.9 Backscattered scanning electron microscopy

The backscattered scanning electron microscopy procedures were described previously (Wang et al., 2015). Briefly, for molar enamel surface evaluation, D14 mandibles were fixed in 4% PFA overnight. The hemi‐mandibles were carefully dissected and submerged in 1% NaClO for 5 min, rinsed, and acetone dehydrated. The hemi‐mandibles were coated with conductive carbon. For incisor enamel surface evaluation, the 7‐week‐old mandibular incisors were carefully freed from the surrounding bony caps and soft tissue. For cross‐section evaluation, 7‐week hemi‐mandibles were embedded in Epon, cross‐sectioned at 1 mm increments, and imaged using a Hitachi S‐3000N microscope.

### 2.10 Focused ion beam scanning electron microscopy

The sample preparation and Focused ion beam scanning electron microscopy (FIB‐SEM) procedures were described previously (Smith, Hu, Hu, & Simmer, 2016).

## 3 RESULTS

### 3.1 Novel *AMBN* mutations causing AI

**Figure 1:**
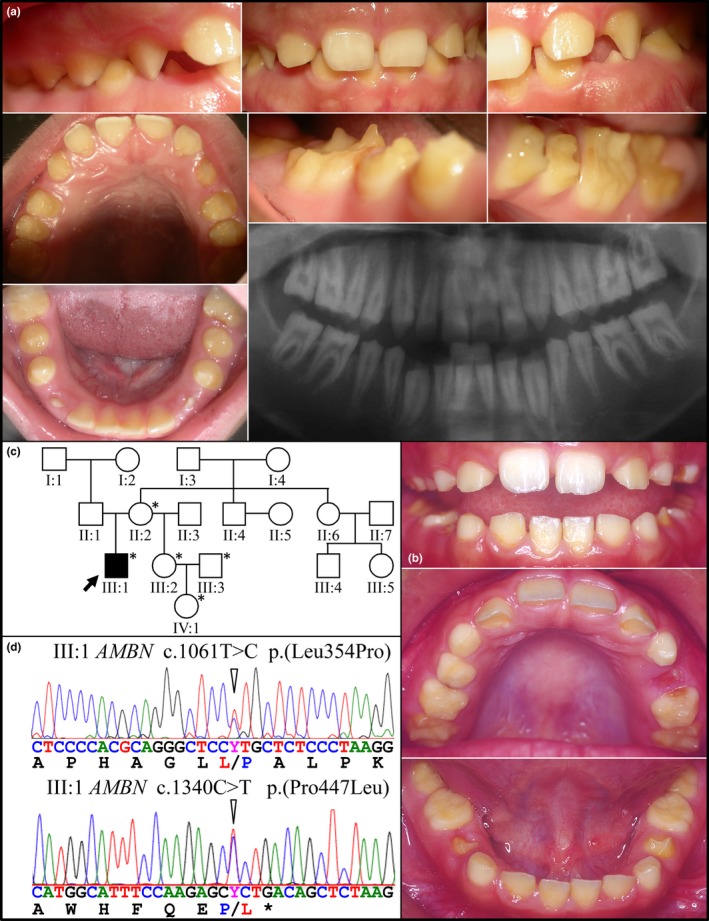
The AI family with compound heterozygous *AMBN* mutations. (a) Intraoral photos of the proband (III:1) at age 12 revealing dental attrition and extreme enamel hypoplasia in the secondary (permanent) dentition. No enamel is detected on the panoramic radiograph. (b) Intraoral images of proband at age 10 showing enamel hypoplasia and attrition in the primary dentition during the mixed dentition stage. (c) Pedigree of the *AMBN* AI family with five individuals recruited (asterisks). Only the proband was affected, which is consistent with a recessive pattern of inheritance. (d) Chromatography showing the *AMBN* compound heterozygous mutations: NG_042078.1:g.19190T>C; NM_016519.5:c.1061T>C; p.(Leu354Pro) and NG_042078.1:g.19469C>T; NM_016519.5:c.1340C>T; p.(Pro447Leu)

We recruited a three‐generation Hispanic family with recessive AI characterized by extremely thin or nonexistent enamel affecting both the primary and secondary dentitions, with no radiopaque layer covering dentin dental radiographs (Figure 1). The enamel defects segregated with the presence of compound heterozygous missense mutations in *AMBN* (NG_042078.1: g.19190T>C; NM_016519.5: c.1061T>C; p.Leu354Pro and NG_042078.1: g.19469C>T; NM_016519.5: c.1340C>T; p.Pro447Leu). Leu354 is a highly conserved amino acid (Delsuc et al., 2015). The p.Leu354Pro substitution gave a Sorting Intolerant from Tolerant (SIFT; Kumar, Henikoff, & Ng, 2009) score of 0.001 and a Polymorphism Phenotyping v2 (Polyphen2; HDIV; Adzhubei et al., 2010) score of 1. This mutation is rare. Its frequency is 0.042 in the ExAC (Lek et al., 2016) and 0.0176 in the 1000 genomes databases. The mother (II:2) and half‐sister (III:2) of the proband (III:1) were heterozygous for the p.Leu354Pro substitution. Very minor enamel pitting was observed in the two subjects heterozygous for the c.1061T>C/ p.Leu354Pro defect (Figure S1). The second *AMBN* mutation (p.Pro447Leu) changes the codon of the C‐terminal amino acid and is invariant in all 53 mammalian species expressing a functional ameloblastin protein (Delsuc et al., 2015). The *AMBN* p.Pro447Leu substitution gave a SIFT score of 0.000 and a Polyphen2 score of 1, supporting a deleterious effect on the protein. This mutation is rare and not listed in the ExAC (0.3.1) or 1000 genomes databases. The p.Pro447Leu variant was apparently inherited from the unrecruited father and was not observed in the other four recruit family members besides the proband. As no simple heterozygote carrying this c.1340C>T/ p.Pro447Leu mutation was recruited, the potential for it causing a heterozygous phenotype could not be assessed. Whole exome sequencing did not help identify any other potentially disease‐causing mutation among our current list of 42 AI candidate genes (Table S1), nor was any other potential disease‐causing candidate gene identified using whole exome analyses. The *AMBN* genomic reference sequence with the locations of known AI‐causing *AMBN* mutations is provided in Figure S2.

### 3.2 Generating *Ambn* ^lacZ/lacZ^ knockin mice

**Figure 2:**
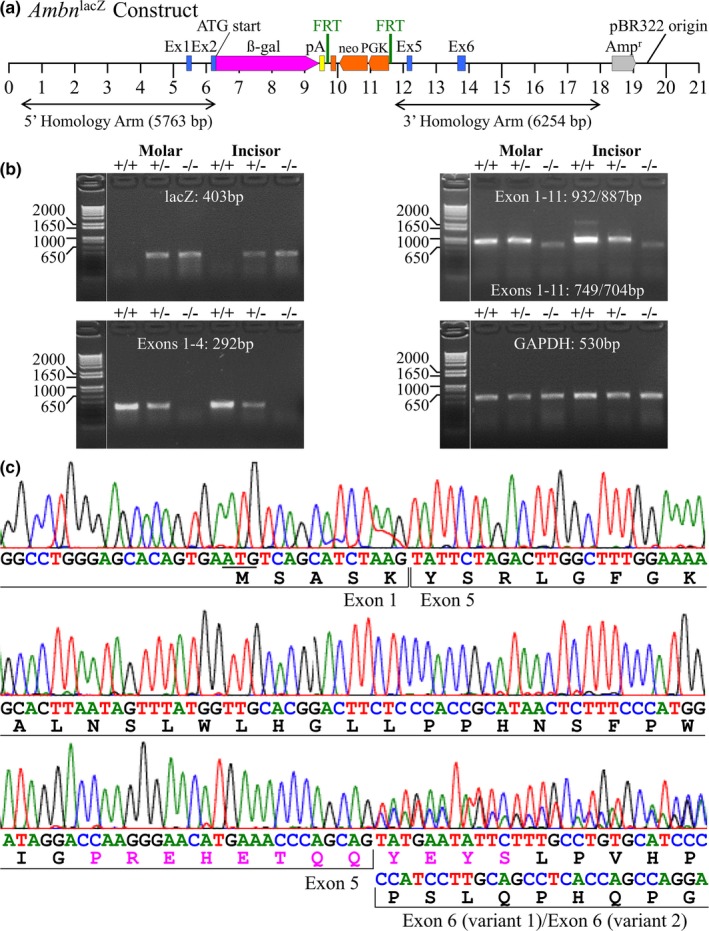
Ameloblastin knockout/NLS‐lacZ knockin mouse (*Ambn* ^lacZ/lacZ^) generation and RT‐PCR verification. (a) Diagram of the *Ambn* ^lacZ^ construct used to delete 4,204 bp from the 5' end of the mouse C57BL/6J *Ambn* starting with the translation initiation codon in Exon 2 and ending in Intron 4. The deleted segment encoded 56 amino acids including the signal peptide, signal peptide cleavage site (after Ala^26^) and key phosphorylated residues, Ser^20^ and Ser^22^. This 4,204 bp segment was replaced with NLS‐lacZ encoding ß‐galactosidase with a nuclear localization signal (NLS) followed by two downstream polyadenylation signals (pA) and an FRT‐PGK‐neo‐FRT selection cassette [that expressed a neomycin (neo) resistance gene driven by the mouse phosphoglucokinase (PGK) gene promoter]. After the selection cassette was excised by Flp recombinase, the size of the insert was 3,419 bp. The sequences of *Ambn^+/+^* and *Ambn* ^lacZ^ are provided in Figures S3, S4 respectively. (b) Ethidium bromide stained agarose gels showing RT‐PCR amplified mRNA extracted from D5 mouse first molar enamel organ epithelium. (c) Chromatography showing partial sequence of *Ambn* ^lacZ/lacZ^ mouse cDNA amplified by primers in Exon 1 and 11. Amino acids colored in magenta indicate epitope used to generate AMBN antibody

An *Ambn* knockout/NLS‐lacZ knockin mouse was generated using a knockin construct (Figure 2a) to delete 4,204 bp segment from the 5' end of *Ambn* in the C57BL/6J mouse background. The knockin insert started at the evolutionarily invariant strong translation initiation codon in Exon 2 and ended in Intron 4 (Figure S3). The deleted *Ambn* segment was replaced with a 3,419 bp knockin sequence so that the ATG translation start site of NLS‐lacZ precisely replaced the ATG of the strong *Ambn* translation start site in Exon 2 (Figure S4), which, like *Ambn* in the wild‐type mouse, was also positioned in‐frame with the weak translation start site in Exon 1. To minimize transcription of the remaining downstream *Ambn* exons, two transcription termination signals were included in the 3' untranslated region following the NLS‐lacZ translation termination codon (TAA). Whole‐mount X‐gal staining of D2 (Figure S5) and D6 (Figure S6) mice showed strong X‐gal staining (for ß‐galactosidase activity) restricted to the incisor/molar regions with only trace staining in these areas in the wild‐type mice. X‐gal staining of D2, D6, and D14 mouse head sagittal sections showed strong ß‐galactosidase activity specific for ameloblasts in molars (Figures S7–S9) and incisors (Figures S10, S11), with spotty ß‐galactosidase activity along the roots (Figures S8, S9) in *Ambn* heterozygous (*Ambn* ^+/lacZ^) and *Ambn* ^lacZ/lacZ^ mice. X‐gal staining demonstrated that NLS‐lacZ knockin was strongly expressed from the *Ambn* promoter in *Ambn* ^+/lacZ^ and *Ambn* ^lacZ/lacZ^ mice, but was not expressed in wild‐type mice. These studies provide strong evidence that NLS‐lacZ knockin is expressed from the *Ambn* promoter, and nuclear X‐gal staining provides an accurate and sensitive signal reporting its tissue‐specific expression.

### 3.3 Verifying the *Ambn* knockout

To assess the possibility of AMBN protein expression from our *Ambn* ^lacZ^ knockin allele, we performed RT‐PCR analyses using mRNA isolated from the enamel organ epithelia (EOE) of developing molars extracted from *Ambn* ^+/+^, *Ambn* ^+/lacZ^, and *Ambn* ^lacZ/lacZ^ mice. Amplification using primers that annealed to *Ambn* Exon 1 and lacZ in the modified Exon 2 generated the expected 403 bp RT‐PCR product in both the *Ambn* ^lacZ/lacZ^ and *Ambn^+^* ^/lacZ^ mice, demonstrating that NLS‐lacZ knockin mRNA was correctly expressed (Figure 2b). RT‐PCR amplification of mRNA isolated from D5 first molar enamel organ epithelia (EOE) using primers that annealed to *Ambn* Exons 1 and 4 produced no product in the *Ambn* ^lacZ/lacZ^ mouse (which lacks *Ambn* Exons 4 annealing site). These primers produced the expected 292 bp RT‐PCR product in wild‐type controls, demonstrating that no wild‐type *Ambn* transcripts were expressed in the *Ambn* ^lacZ/lacZ^ mouse (Figure 2b). RT‐PCR amplification using primers that annealed to *Ambn* Exons 1 and 11 produced a single band in *Ambn* ^+/+^ mice that contained the expected 932 and 887 bp RT‐PCR products corresponding to the two alternatively spliced wild‐type *Ambn* transcripts (*Ambn* transcript variant 1, NM_001303431.1 and transcript variant 2, NM_009664.2; Figure 2b). A faint amplification band lower on the gel was also detected in the *Ambn* ^lacZ/lacZ^ mouse. This band was characterized using DNA sequencing and contained a mixture of two (749 bp and 704 bp) amplification products corresponding to alternatively spliced *Ambn* transcripts missing *Ambn* Exons 2 through 4, and thus lacking the *Ambn* coding region for the strong translation initiation site, signal peptide, signal peptide cleavage site, and the N‐terminal 40 amino acids from the secreted AMBN protein (Figure 2c). It was apparent that this amplicon was generated by amplification of rare *Ambn* ^lacZ^ transcripts that failed to terminate transcription at the two transcription termination signals at the 3' end of NLS‐lacZ knockin, so transcription sometimes continued all the way to the 3' end of *Ambn* and generated transcripts that were spliced from *Ambn* Exon 1 to Exon 5, and then alternatively spliced to include or delete the 5' end of Exon 6 (as occurs in wild‐type transcripts). Inclusion of the 5' end of Exon 6 was favored over its deletion, based upon the relative sizes of the superimposed peaks on the DNA sequencing chromatogram; Figure 2c). SignalP 4.1 predicted that these transcripts, if translated from the weak translation start site in Exon 1, would not have generated a functional signal peptide (Petersen, Brunak, von Heijne, & Nielsen, 2011). Immunohistochemistry of the continuously growing mandibular incisors was performed to determine if any AMBN protein could be detected in *Ambn* ^lacZ/lacZ^ mice.

### 3.4 AMBN and AMEL immunohistochemistry

**Figure 3:**
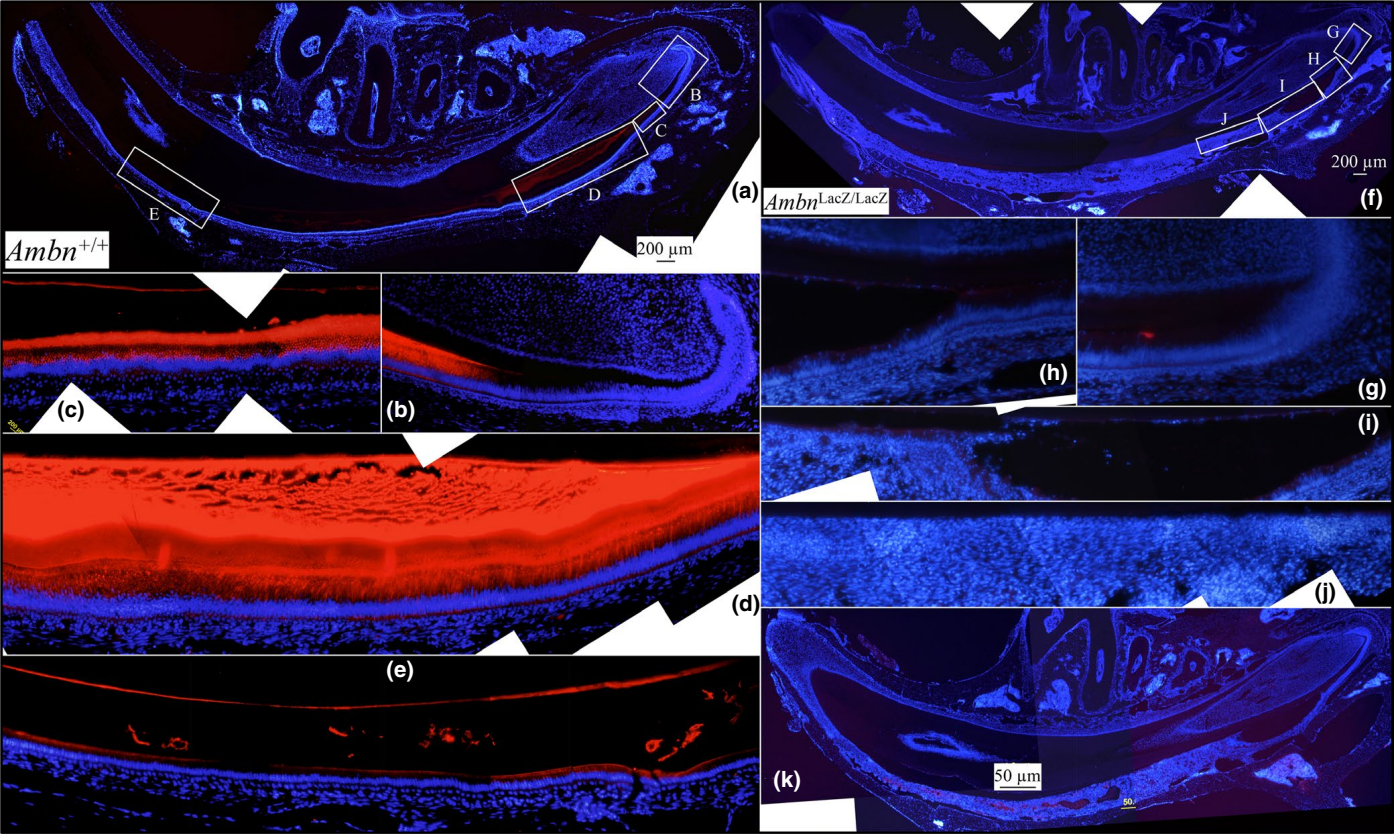
Immunohistochemistry of hemi‐mandible longitudinal sections of 7‐week *Ambn* ^+/+^ (a‐e) and *Ambn* ^lacZ/lacZ^ (f‐k) incisors using an antibody raised against the AMBN peptide CMRPREHETQQYEYS. Red stain is AMBN; blue is nuclei stained with 4',6‐diamidino‐2‐phenylindole (DAPI). (a) Low magnification montage of WT section. Boxes labeled B‐E show positions of high magnification montages below. (b‐d) Strong AMBN signal is observed in secretory stage ameloblasts and the enamel extracellular matrix. (e) Much weaker AMBN signal is observed in maturation stage ameloblasts in the maturing enamel matrix. (f, k) Low magnification montages of *Ambn* ^lacZ/lacZ^ mouse sections. Boxes in f labeled g‐j indicate positions of the high magnification montages below. (h‐j) AMBN signal is absent in the *Ambn* ^lacZ/lacZ^ mouse mandibular incisor sections. (k) A second longitudinal section from *Ambn* ^lacZ/lacZ^ mouse showed minor autofluorescence within blood vessels in pathological areas. Similar autofluorescence was observed in this region using the amelogenin antibody (Figure 4)

AMBN immunohistochemistry was performed on 7‐week‐old *Ambn^+^* ^/^ *^+^* and *Ambn* ^lacZ/lacZ^ littermates using an affinity‐purified antipeptide antibody recognizing an epitope encoded by *Ambn* Exons 5 and 6 (Iwata et al., 2007). In *Ambn^+/+^* incisors, AMBN signal was specific for ameloblasts and their associated extracellular matrix and was significantly stronger in the secretory, relative to the maturation stage (Figures 3a‐e). No AMBN immunofluorescence was observed in odontoblasts, pulp, or bone. In the *Ambn* ^lacZ/lacZ^ incisors, there was no AMBN immunofluorescence in ameloblasts or in the enamel matrix (Figure 3f‐k). The only positive signal was cross‐reaction with material in vascular spaces in the thick pathological epithelium covering the enamel space, which was characteristic of the pathology evident in the enamel organ epithelia *Ambn* ^lacZ/lacZ^ mice. As no AMBN could be detected, we conclude that the enamel phenotype in *Ambn* ^lacZ/lacZ^ mice is due to the absence of AMBN protein.

**Figure 4:**
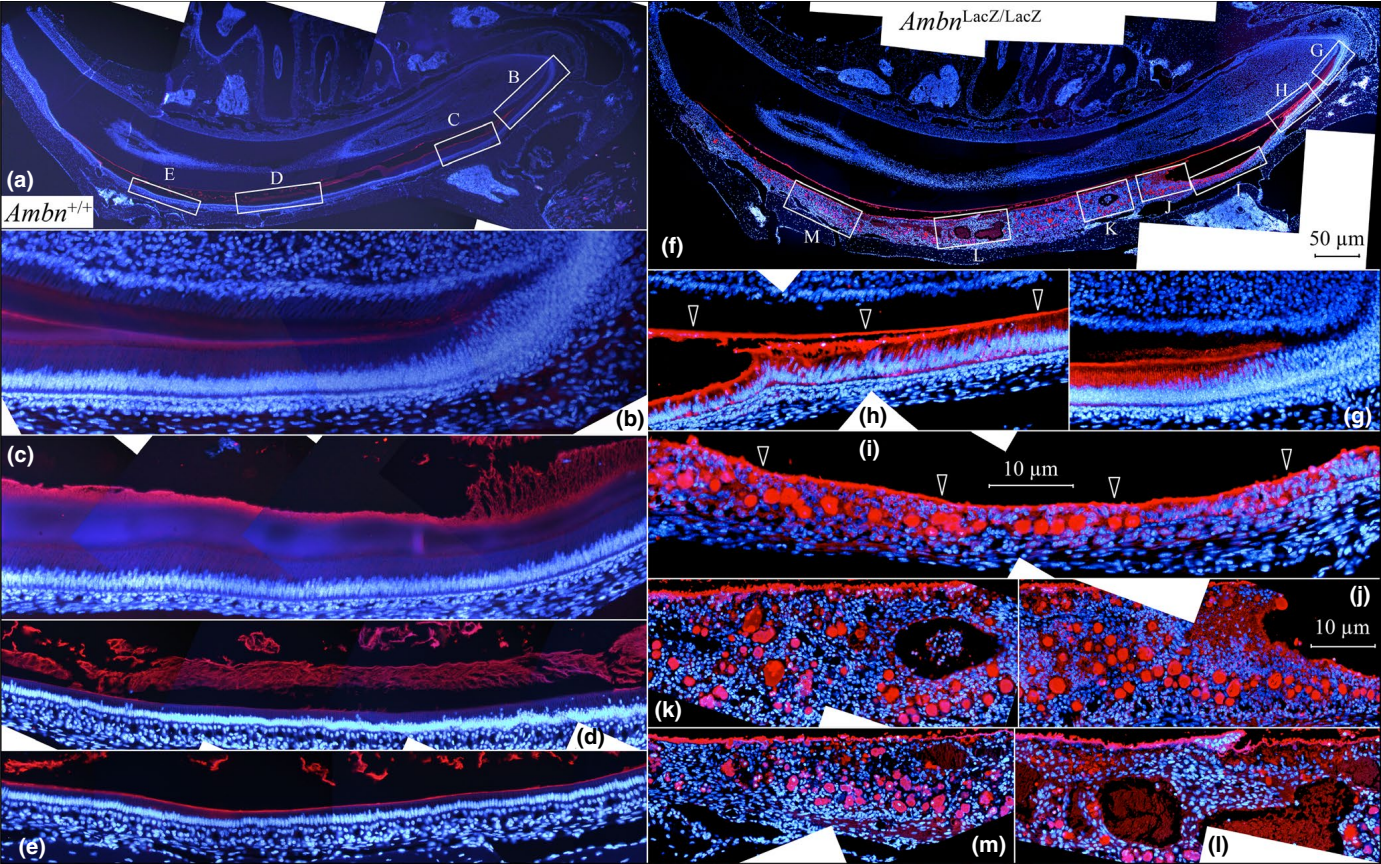
Immunohistochemistry of hemi‐mandible longitudinal sections of 7‐week *Ambn* ^+/+^ (a‐e) and *Ambn* ^lacZ/lacZ^ (f‐m) incisors using an antibody raised against the recombinant mouse amelogenin rM179 (Simmer et al., 1994). Red stain is AMEL; blue is nuclei stained with 4',6‐diamidino‐2‐phenylindole (DAPI). (a) Low magnification montage of *Ambn* ^+/+^ section. Boxes labeled B‐E show positions of high magnification montages shown below. (b, c) Amelogenin signal is detected in the enamel matrix during the secretory stage and in the early dentin matrix. (d, e) Amelogenin signal diminishes in the enamel matrix during the maturation stage. (f) Low magnification montages of *Ambn* ^lacZ/lacZ^ mouse section. Boxes in f labeled g‐m indicate positions of the high magnification montages below. (g) AMEL stain is strong in ameloblasts and in the matrix along their distal membrane. Some amelogenin has penetrated into dentin. (h‐j) Amelogenin accumulates along the dentin surface and stratum intermedium (arrowheads). A cyst has formed beneath the ameloblasts and is associated with the onset of severe ameloblast pathology and circular pockets of amelogenin within the enamel organ epithelia. (k‐l) Brown autofluorescence is observed in sinuses containing blood elements

Histologically, the *Ambn* ^lacZ/lacZ^ enamel organ epithelia showed extensive pathology (Figure 3f‐k), which was particularly evident following AMEL immunostaining (Figure 4f‐m). Differentiating ameloblasts and preameloblasts appear to be normal and secrete AMEL onto the dentin surface, some of which penetrated into the dentin layer (Figure 4g). The ameloblasts degenerated and detached from the dentin surface (Figure 4h‐j). Separation appeared to occur within the unmineralized enamel matrix, leaving distinct layers of accumulated AMEL on the dentin surface and also along the detached distal surface of now pathological EOE (Figure 4h‐i). Amelogenin seemed to accumulate in pockets between the degenerating ameloblasts and the stratum intermedium (Figure 4h). A cyst‐like structure formed between the dentin surface and the EOE (Figure 3h‐j), which compressed the epithelial layer as the enamel organ epithelia progressively lost its characteristic tissue organization evident in wild‐type mice (Figures 4b‐e). Amelogenin continued to be secreted, but within the disorganized epithelium, generating numerous AMEL‐rich spherical nodules. The pathology in the epithelial layer covering dentin in the *Ambn* ^lacZ/lacZ^ incisors was similar to that previously described in the *Ambn* ^Δ5,6/Δ5,6^ mice, with two apparent differences: the *Ambn* ^Δ5,6/Δ5,6^ mice expressed an internally truncated AMBN protein that accumulated in the soft tissue nodules (Wazen et al., 2009), and amelogenin expression appeared to be suppressed (Fukumoto et al., 2004). In contrast, our *Ambn* ^lacZ/lacZ^ mice were negative for AMBN expression, but continued secreting large amounts of amelogenin (Figure 4f‐m).

### 3.5 Enamel surface in *Ambn* ^lacZ/lacZ^ mice

**Figure 5:**
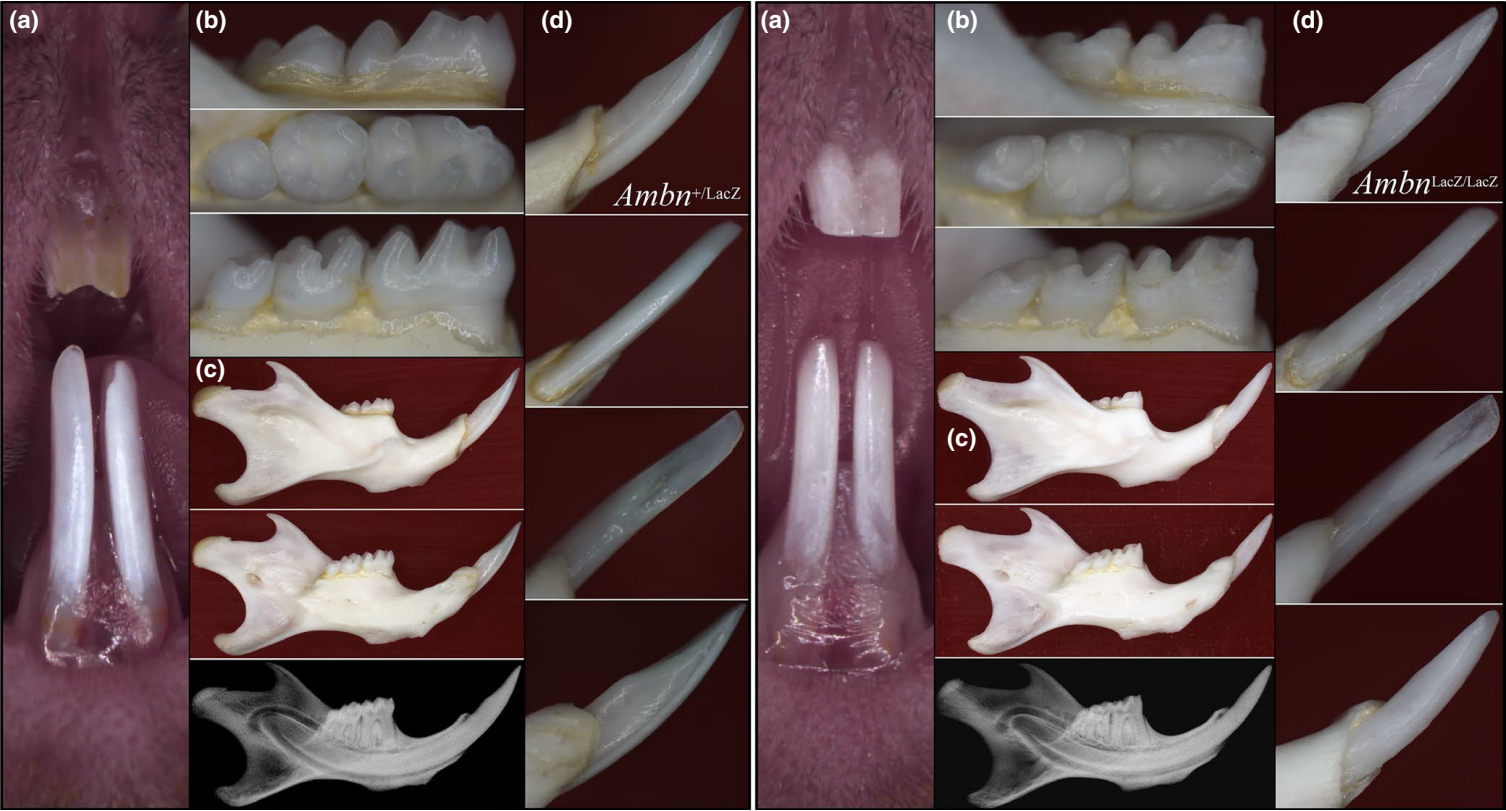
Dissecting microscope photographs of 7‐week‐old *Ambn* ^+/lacZ^, and *Ambn* ^lacZ/lacZ^ mice. (a) Frontal view of maxillary and mandibular incisors. (b) Buccal (upper), occlusal (middle), and lingual (lower) views of mandibular molars. As shown in the radiographs, no appreciable abnormality in root number, size, and shape was observed in the *Ambn* ^+/lacZ^ and *Ambn* ^lacZ/lacZ^ mice. (c) Lateral (top), medial (middle) views, and radiograph (bottom) of hemi‐mandibles. (d) Lingual (top), labial, lingual, and buccal (bottom) views of mandibular incisors. Comparable images from a 7‐week‐old *Ambn* ^+/+^ mouse are shown in Figure S12

Malformation of the enamel layer was evident by inspection under a dissection microscope of the mandibular incisors and molars of *Ambn* ^lacZ^ homozygous and heterozygous mice (Figure 5) relative to those of the wild‐type (Figure S12). The *Ambn* ^lacZ/lacZ^ maxillary and mandibular incisors at 7 weeks were chalky‐white with rough surfaces and no detectable enamel layer. The *Ambn* ^lacZ/lacZ^ molar crowns also lacked enamel and underwent rapid attrition. *Ambn* ^+/lacZ^ mice displayed a well‐defined enamel layer. Their maxillary incisors appeared normal in color, whereas the mandibular incisors were chalky‐white and sometimes chipped. The molars look essentially normal, but their surface texture was slightly rougher than that of wild‐type molars. Radiographs revealed no abnormalities in root number, size, or shape in the *Ambn* ^lacZ/lacZ^ mice.

**Figure 6:**
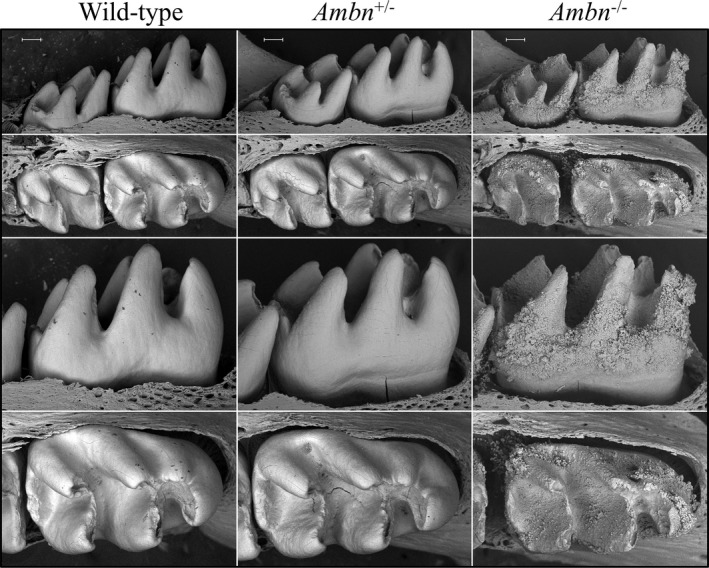
Backscattered SEMs of D14 mandibular molars of *Ambn* ^+/+^ (Wild‐type), *Ambn* ^+/lacZ^ (*Ambn* ^±^) heterozygous, and *Ambn* ^lacZ/lacZ^ (*Ambn* ^−/−^) null mice following soft tissue removal. These images show the molars immediately prior to their eruption into occlusion. The top and third rows are lingual views; the second and bottom rows are occlusal views. The null molars show thin cusps and surface accumulation of an irregular crusty material. Mild surface roughness of the heterozygous molar relative to the wild‐type is most evident from the occlusal views. Another set of molars is shown in Figure S13. Scale bars = 500 µm

**Figure 7:**
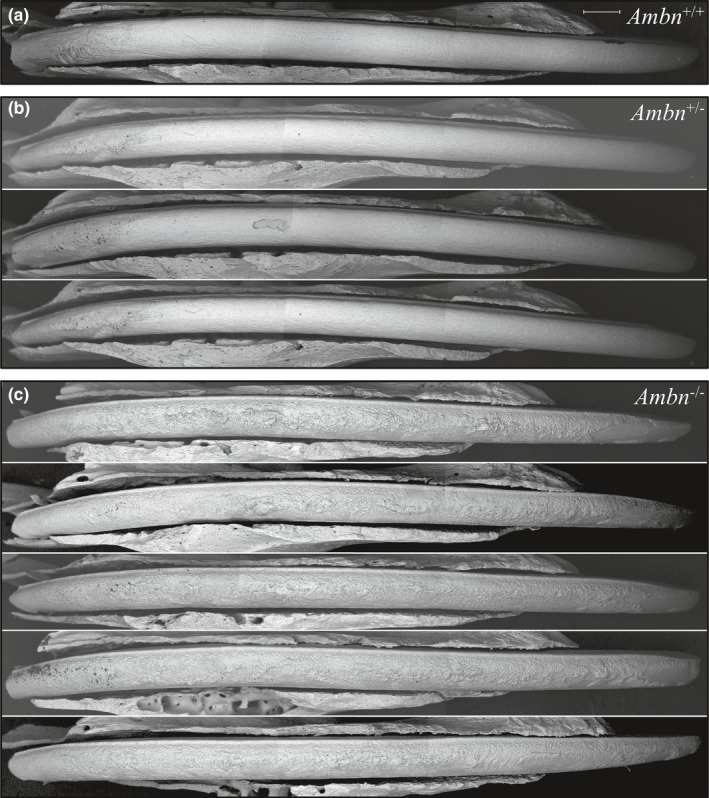
bSEM montages of 7‐week mandibular incisors denuded of overlying tissues. The labial surfaces are shown, from the basal (left) to erupted (right) ends of the incisors. Dark areas contain less surface mineral than white areas. The surface of secretory stage enamel (left ~1/4 of the incisor length) appears rough in all genotypes. The maturation stage enamel surface of the *Ambn* ^+/+^ incisor (a) was similar to those from the *Ambn* ^+/lacZ^ mice (b) whereas the *Ambn* ^lacZ/lacZ^ homozygous incisors (c) exhibited very rough, poorly mineralized (dark), crusty surfaces with protruding nodules. Scale bar = 500 µm

To get a closer look at fully formed enamel surfaces prior to their alteration in the oral cavity, nearly erupted D14 mandibular first molars were characterized using backscattered scanning electron microscopy (bSEM) following removal of the thin overlying soft tissue (Figure 6 and Figure S13). The *Ambn* ^+/lacZ^ mandibular first molar crowns were indistinguishable from wild‐type crowns except for a slightly rougher surface texture. The *Ambn* ^lacZ/lacZ^ mandibular first molars, in contrast, had thin cusps, and their crowns were covered by irregular masses of crusty mineral. The enamel surfaces of carefully extracted 7‐week mandibular incisors were inspected using bSEM (Figure 7). The wild‐type (Figure 7a) and the *Ambn* ^+/lacZ^ mandibular incisors (Figure 7b) looked similar with respect to surface roughness and degree of mineralization (grayscale). The *Ambn* ^lacZ/lacZ^ incisors were hypomineralized (dark), rough‐surfaced (Figure 7c), and still retained the fragile crusty mineral nodules on their unerupted, unabraded surfaces.

### 3.6 bSEM of mandibular incisor cross‐sections

**Figure 8:**
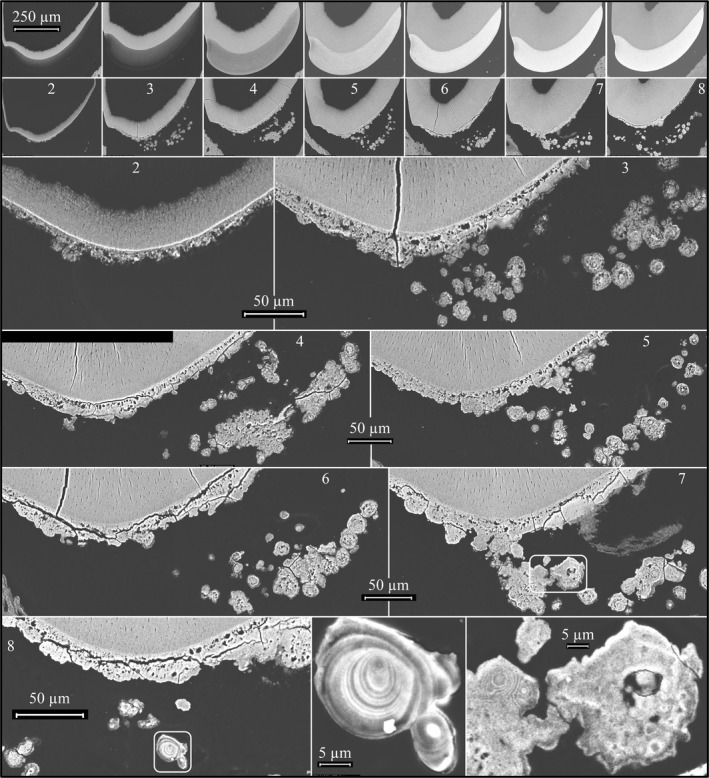
bSEM images of a wild‐type and an *Ambn* ^lacZ/lacZ^ homozygous 7‐week mandibular incisor cross‐sections at 1 mm increments. Numbers in panels indicate distance in mm from apical loop. The higher the number the more advanced is the dentin and enamel development. *Top*: Lower magnifications views of wild‐type above the corresponding *Ambn* ^lacZ/lacZ^ homozygous mouse incremental sections. Note that dentin formation is comparable in the wild‐type and *Ambn* null mice, but that enamel formation is not. *Below*: Higher magnification bSEM images of sections 2 through 8 and details of boxed concretions

To gain information about the formation of the crusty mineral surface, *Ambn* ^lacZ/lacZ^ incisors were cross‐sectioned at 1 mm increments and imaged using bSEM (Figure 8). Dentin formation was comparable in the wild‐type and *Ambn* ^lacZ/lacZ^ mice, while the entire enamel layer was pathological in the *Ambn* ^lacZ/lacZ^ incisors. During what corresponded to the secretory stage in wild‐type incisors (levels 1–3 located 1 to 3 mm from the apical loop), an irregular mineral crust formed on the surface of dentin that was already evident at level 2. The level 2 crust was less mineralized than dentin and contained unmineralized voids within it, evidently caused by the trapping of soft tissue (epithelia). The crust was continuous along the dentin surface but varied significantly in thickness throughout, averaging about 15 µm. By level 3 (nearing the end of what would be the secretory stage in wild‐type mice), the crust on the dentin surface had expanded to about 20 µm in thickness, while a large number of spherical mineral foci, often fused together, appeared within the epithelial layer at various distances from the irregular crusty surface. Some of these mineral foci showed concentric rings in cross‐section and looked like concretions. The concretions were more abundant laterally and appeared to expand in size incisally, being larger at levels 6 through 8 when compared to the nodules in levels 3 through 5. These detached nodules correlated with the presence of amelogenin‐filled pockets within the EOE that characterized the histological sections of *Ambn* ^lacZ/lacZ^ mice immunostained for amelogenin (Figure 4). In wild‐type incisors, the enamel was constant in thickness from levels 4 through 8 (being about 114 µm thick at the height of contour) as ions excreted by maturation stage ameloblasts do not continue to add mineral ions to the tips of the enamel crystals (which would progressively thicken the enamel layer as a whole), but instead the ions penetrate into the enamel matrix and adsorb exclusively onto the sides of the enamel crystals (Smith, 1998).

### 3.7 FIB‐SEM

To view events on the surface of dentin at the point on the mandibular incisor where enamel normally initiates as oriented mineral ribbons (Smith et al., 2016), an *Ambn* ^lacZ/lacZ^ incisor was cross‐sectioned 1 mm from the basal loop and characterized using FIB‐SEM, with overlapping images taken at 5000x, 10000x, 20000x, and 35000x (Figure S14–S23). The series spanned 155 µm of developing tooth along the ameloblast distal border. The earliest developmental stage on the section was the most lateral and shows the distal part of the ameloblasts intimately associated with pre‐dentin matrix following the breakdown of the basement membrane and onset of amelogenin secretion but prior to the onset of dentin mineralization (Figure S14). The series progressed to show the onset of dentin mineralization within collagen fibers oriented parallel to the long axis of the ameloblast and terminating against its distal membrane (Figure S15). Mineralization of the collagen‐rich pre‐dentin matrix expanded toward the ameloblast membrane and also in the opposite direction toward the odontoblasts, while coalescing into a continuous mineral mass of steadily increasing density. At this early stage pathology was evident in the form of droplets of enamel matrix, presumed to be comprised of amelogenin, that accumulated along the ameloblast membrane where the enamel ribbons normally initiate (Figures S16, S17). The dentin mineral increased in density and thickness, but no enamel ribbons formed (Figure S18). As the process of dentin mineralization continued normally, no enamel ribbons formed in the *Ambn* ^lacZ/lacZ^ incisor. Instead, enamel proteins (particularly amelogenin) accumulated between the mineralized dentin surface and the nearby ameloblast distal membrane (Figures S18–S23).

To test our interpretation that the pathological accumulations in the *Ambn* ^lacZ/lacZ^ were amelogenin, we crossed the *Ambn* ^lacZ/lacZ^ and *Amelx* ^‐/‐^ mice to generate *Ambn* ^−/−^ *Amelx* ^−/−^ double null mice. We extensively characterized the mandibular incisors in the double null in the region where enamel ribbons should have formed (Figures [Link], [Link]), starting prior to dentin coalescing into a continuous mineral layer (Figure S24), and continuing long past the normal onset of enamel formation and on to a point where overt cell pathology was evident and crusty mineral started to form on dentin (Figures S38–S40, S42). The accumulations of enamel protein observed between ameloblasts and the underlying dentin surface in *Ambn* ^lacZ/lacZ^ mice were absent in the *Ambn* ^−/−^ *Amelx* ^−/−^ mice, demonstrating that the material accumulating on the dentin surface was largely amelogenin, which was supported by the finding of strong amelogenin immunostaining on the dentin surface using light microscopy (Figure 4).

**Figure 9:**
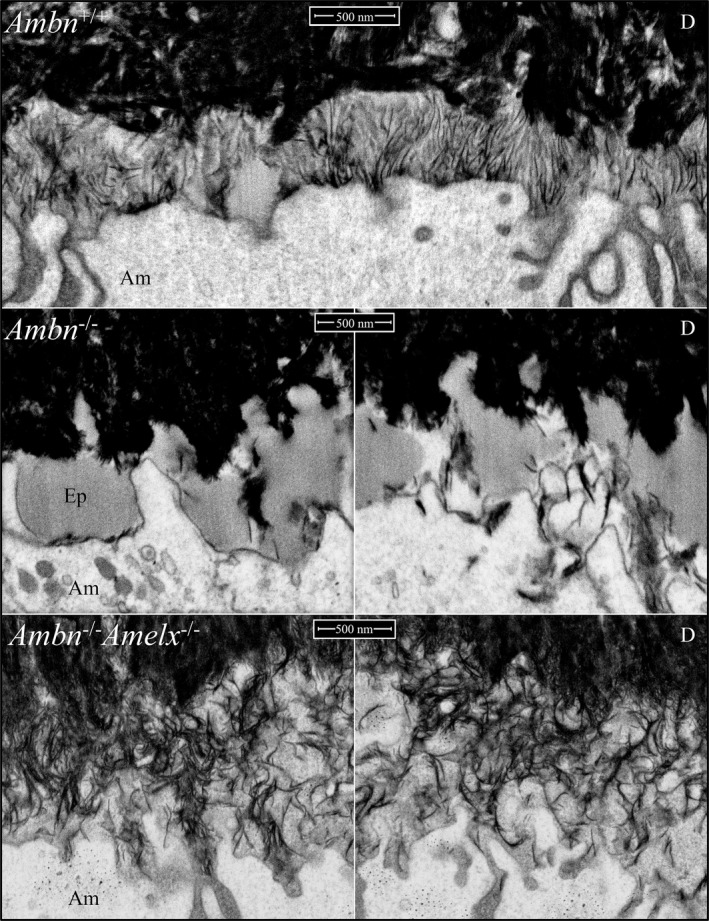
FIB‐SEM images of corresponding dentin surfaces in wild‐type (*Ambn* ^+/+^), *Ambn* null (*Ambn* ^lacZ/lacZ^), and *Ambn*‐*Amelx* double null (*Ambn* ^lacZ^ *^/^* ^lacZ^ *Amelx* ^−/−^) mice. *Top*: Formation of a characteristic field of initial enamel ribbons extending from dentin mineral to the ameloblast membrane. *Middle*: Accumulation of enamel protein, mostly amelogenin, on the dentin surface and absence of a field of enamel ribbons in *Ambn* ^lacZ/lacZ^ mice. *Bottom*: *Ambn*‐*Amelx* double null mice show an absence of amelogenin accumulation and no field of enamel deposition. *Key*: Am, ameloblast; D, dentin; Ep, enamel protein

The series of high magnification FIB‐SEM images detailing the early dentin and enamel formation in the *Ambn* ^lacZ/lacZ^ and *Ambn* ^−/−^ *Amelx* ^−/−^ double null mice strongly support the interpretation that dentin forms normally in the absence of ameloblastin, but the mineralization front along the ameloblast distal membrane was defective and no enamel ribbons formed. Instead, a very thin (~1 µm), sparse layer of curled mineral was deposited on dentin (Figure 9) well after the normal onset of enamel mineralization, when dentin mineralization was well underway and ameloblasts had started to deteriorate. The curled ribbons were not oriented from the mineralized dentin to the ameloblast distal membrane and did not elongate (Figure 9). For comparison, a full series of wild‐type FIB‐SEM images covering the onset of dentin mineralization through formation of the initial enamel are provided (Figures S45–S66). *Enam* ^‐/‐^ mice also fail to form enamel ribbons (lack enamel), but do not accumulate deposits of amelogenin between the ameloblasts and dentin (Smith et al., 2016). *Amelx* ^‐/‐^ null mice initiate enamel ribbon formation almost normally, but the process degenerates rapidly and the ribbons convert into octacalcium phosphate and branch into fan‐like structures, forming plates (Hu et al., 2016).

### 3.8 NLS‐lacZ expression in non‐dental tissues

**Figure 10:**
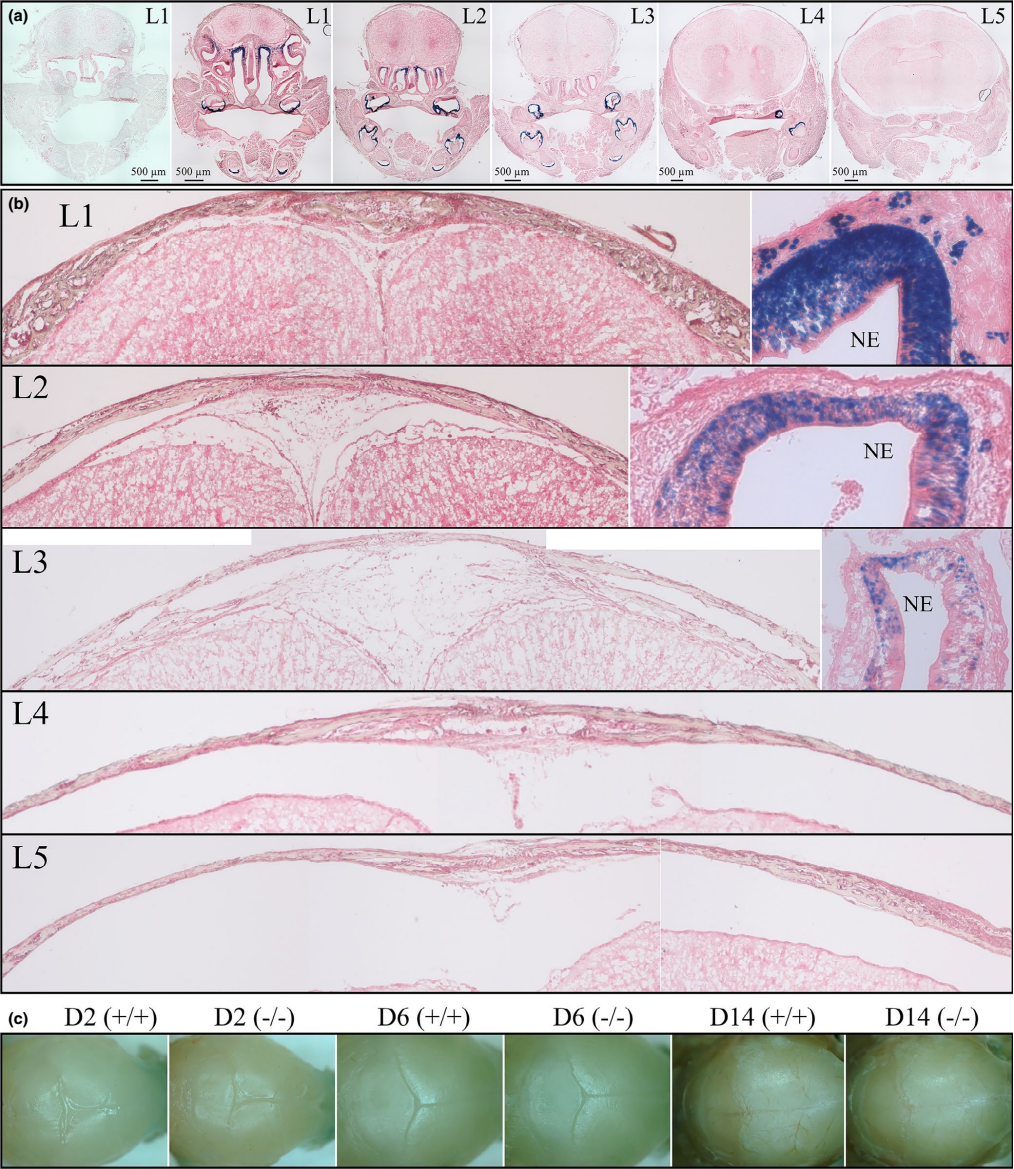
LacZ staining of mouse heads. Slides were stained in X‐gal solution for 1 hr at 45°C and then counterstained with Nuclear Fast Red. (a) Successive coronal plane sections (ventral L1 to L5 dorsal) of mouse heads. (b) Sagittal suture at L1 to L5 and nasal epithelium (NE) at L1 to L3 under higher magnification. (c) Whole‐mount lacZ staining of mouse heads from D2, D6 and D14 wild‐type (*Ambn^+/+^*) and null (*Ambn* ^lacZ/lacZ^) demonstrating no appreciable NLS‐lacZ positive staining. Scale bars = 500 µm

Because NLS‐lacZ was inserted at the *Ambn* Exon 2 translation initiation codon (TIC) while deleting the *Ambn* coding region from the TIC through Exon 4, the *Ambn* ^lacZ^ knockin mice allowed us to assess tooth formation in the absence of *Ambn* expression and also detect ß‐galactosidase reporter expression from the *Ambn* promoter using the sensitive X‐gal staining assay. X‐gal staining of tissues containing developing teeth helped confirm that the *Ambn* ^lacZ/lacZ^ mouse was established properly. Strong X‐gal staining of ameloblasts was observed with 1 hr incubations (Figures S6–S12). Trace expression was detected in the molar junctional epithelium at D28, but only after incubation overnight (15.5 hr; Figure S67). We sought to detect even trace expression in non‐dental tissues by using 15.5 hr (overnight) X‐gal incubations. To assess cranial tissues and calvaria, we cross‐sectioned D6 *Ambn* ^lacZ/lacZ^ mouse heads at five different levels (Figure 10). X‐gal staining was intense in the ameloblasts of developing teeth and strong in the nasal epithelium, but no staining was detected in the calvarium or the frontal suture. The calvarium and sutures were also negative in D2, D6, and D14 whole‐mounts (Figure 10c). Soft tissue organs were also tested for X‐gal staining. Duct cells in the tail of the epididymis were positive after 1 hr of X‐gal staining (Figure S68). All other organs were negative even after 15.5 hr of X‐gal staining, including the ovary and oviduct, uterus, prostate, seminal vesicle, and testis (Figure S69), as well as the submandibular salivary gland, kidney, liver, and bladder (Figure S70).

## 4 DISCUSSION

### 4.1 *AMBN* and amelogenesis imperfecta

Despite the early identification of AMBN cleavage products in developing enamel (Fukae & Tanabe, 1987a, 1987b; Shimizu, 1984), the cloning and characterization of *Ambn* cDNA from multiple organisms, the early recognition of AMBN as a major constituent of developing enamel (Fincham et al., 1999), its association with dramatic enamel defects in *Ambn* mutant mice (Fukumoto et al., 2004), and linkage of *AMBN* to a chromosomal region associated with human enamel defects (MacDougall et al., 1997), only two *AMBN* mutations have been found to cause amelogenesis imperfecta (AI). A homozygous genomic deletion (c.294+139_531+478del; p.Tyr99_Glu177del; Poulter et al., 2014) and a homozygous splice‐junction defect predicted to cause a deletion by exon skipping during RNA splicing (c.532‐1G>C; p.Leu178_Ser190del; Prasad et al., 2016) are the only *AMBN* mutations shown to cause AI. Both of these mutations caused autosomal recessive AI (ARAI), with no defects reported besides those in enamel. Here we report two *AMBN* missense mutations (c.1061T>C; p.Leu354Pro/ c.1340C>T; p.Pro447Leu) that caused severely hypoplastic ARAI in compound heterozygotes, with minor pitting in single heterozygotes for the *AMBN* c.1061T>C; p.Leu354Pro defect. In contrast, over 20 *ENAM* mutations have been reported to cause AI (Zhang et al., in press).

### 4.2 The *Ambn* ^lacZ/lacZ^ enamel phenotype and tissue‐specific expression

The most fundamental feature of dental enamel formation is the deposition (on dentin mineral) and elongation of thin mineral ribbons along the secretory surface of the ameloblast distal membrane. This occurs in all vertebrates that make true enamel, and existed 450 MY ago in the most recent common ancestor of humans and gar. Ameloblastin (*Ambn*) and enamelin (*Enam*) are expressed during tooth and scale formation in the gar, whereas amelogenin (*Amel*) is not (Braasch et al., 2016; Kawasaki et al., 2017). Amelogenin apparently evolved shortly thereafter, as it is found in sarcopterygian genomes, such as the coelacanth (Kawasaki & Amemiya, 2014), but not in gar or teleosts (Kawasaki, 2018). No enamel ribbons form in *Enam* ^‐/‐^ mice. Enamel ribbons form in *Amelx* ^‐/‐^ mice, but the ribbons are short, elongate more slowly than normal (Hu et al., 2008; Smith et al., 2016), and develop into a thin layer of octacalcium phosphate plates (Hu et al., 2016). Here we demonstrate that no field of initial enamel ribbons form in the absence of ameloblastin (Figure 9). Dentin forms normally, but enamel ribbons do not form on its surface. This is true even though amelogenin accumulates on the dentin surface where the enamel ribbons would normally initiate.

A remarkable feature observed in both the *Enam* ^‐/‐^ and *Ambn* ^lacZ/lacZ^ mice, in addition to the complete failure to deposit a field of initial enamel ribbons (Figure 9), is the pathology and ectopic mineral evident within the enamel organ epithelia (Hu et al., 2011; Figure 4). Histologically, the *Ambn* ^lacZ/lacZ^ ameloblasts degenerate and detach from the dentin surface following their failure to deposit the initial enamel. Amid this pathology, an irregular mineral crust is deposited on the molar (Figure 6) and incisor (Figures 7 and 8) surfaces, which does not form through the normal process of enamel mineral ribbon deposition (compare WT Figures S58–S65 to *Ambn* ^lacZ/lacZ^ Figures S36–S44). The mineral crust covering dentin in *Ambn* ^lacZ/^ ^lacZ^ mice was readily observed in the bSEM analyses of the D14 first molar (Figure 6) and 7‐week incisor (Figure 7) surfaces, and the 7‐week incisor cross‐sections (Figure 8). The crust underwent rapid attrition after the teeth erupted into occlusion. A unique feature of the failed amelogenesis in the *Ambn* ^lacZ/lacZ^ mice was the accumulation of organic material on the dentin surface where the enamel ribbons failed to form (Figure 9; Figures S16–S23). These accumulations were absent in the *Ambn* ^lacZ/lacZ^ *Amelx* ^‐/‐^ double null (Figures S26–S36), indicating that the deposits were comprised principally of amelogenin. While amelogenin deposits are normally associated with the formation and elongation of enamel ribbons along the ameloblast membrane, the accumulations in *Ambn* ^lacZ/lacZ^ mice were larger than those is observed in wild‐type and *Enam* ^‐/‐^ mice (Smith et al., 2016). We do not infer that amelogenin expression increases, simply that amelogenin accumulates more than normal on the dentin surface as a feature of the failed amelogenesis in *Ambn* ^lacZ/lacZ^ mice. Without ribbon elongation, the ameloblasts do not retreat and expand the enamel volume, so the amelogenin must accumulate on the mineralized dentin after it is secreted.

The phenotypes of the *Ambn* ^lacZ/lacZ^ mice are similar to those of the *Ambn* ^Δ5,6/Δ5,6^ mice, including ameloblast detachment, lack of enamel formation, ectopic mineral formation in the epithelium, and normal dentin formation (Fukumoto et al., 2004; Wazen et al., 2009). The *Ambn* ^Δ5,6/Δ5,6^ mice however, showed reduced *Amelx* expression and developed odontogenic tumors, which were not observed in *Ambn* ^lacZ/lacZ^ mice. The absence of enamel formation in the *Ambn* ^lacZ/lacZ^ and *Ambn* ^Δ5,6/Δ5,6^ mice indicates that ABMN function is dependent upon inclusion of the peptide segment encoded by Exons 5 and 6. We observed strong ectopic expression of amelogenin in the EOE of *Ambn* ^lacZ/lacZ^ mice (Figure 4) that correlated with the formation of ectopic mineral in the EOE (Figure 8). The importance of amelogenin in forming this ectopic mineral is supported by formation of ectopic mineral in the *Ambn* ^Δ5,6/Δ5,6^ mice (Fukumoto et al., 2004), but not in *Ambn* ^Δ5,6/Δ5,6^ *Amelx^−/−^* double mutants (Hatakeyama et al., 2009). An increasing body of evidence suggests that amelogenin does not play a critical role in the shaping of enamel mineral ribbons, including the absence of amelogenin in gar (which forms enamel ribbons; Prostak, Seifert, & Skobe, 1989; Sire, 1995), formation of enamel ribbons in *Amelx^−^* ^/^ *^−^* mice (Smith et al., 2016), enamel mineral adopting its ribbon shape before it is crystalline (and therefore cannot be shaped by amelogenin binding to selected crystal faces; Beniash, Metzler, Lam, & Gilbert, 2009), and ectopic mineral deposited in amelogenin‐laded spaces in *Ambn* ^lacZ/lacZ^ mice that is not comprised of enamel‐like ribbon‐shaped crystals (Figure 8). Amelogenin is a critical matrix constituent, but it can only function properly in the context of a functioning mineralization front apparatus that initiates and shapes the enamel mineral ribbons, which requires both enamelin (ENAM) and ameloblastin (AMBN; Smith et al., 2016).

The NLS‐lacZ reporter expressed nuclear localized ß‐galactosidase activity strongly in ameloblasts, with spotty expression along the developing molar roots. Mildly positive tissues included the tail of the epididymis and nasal epithelium, and trace, spotty signal was detected in the junctional epithelium. Except for enamel deposited by the strongly positive ameloblast layer, no phenotypic differences were observed in any other tissues, including the nasal epithelium, epididymis, and junctional epithelium where reporter signal was detected. We conclude that ameloblastin functions at the mineralization front along the secretory surface of the ameloblast membrane to establish and extend enamel mineral ribbons, a specialized function it has performed for over 450 MY in all vertebrates that produce true enamel, and does not serve any critical function in any other tissues.

## CONFLICT OF INTEREST

None declared.
