# Supplementary material for: AMBN mutations causing hypoplastic amelogenesis imperfecta and Ambn knockout‐NLS‐lacZ knockin mice exhibiting failed amelogenesis and Ambn tissue‐specificity
Source: Mol Genet Genomic Med. 2019 Aug 11;7(9):e929. doi: 10.1002/mgg3.929 (PMC6732285; doi:10.1002/mgg3.929)
Supplement: Supplementary file 1 [file MGG3-7-e929-s001.pdf]

AMELX	*ENAM*	*AMBN*	*MMP20*	*FAM83H*	*KLK4*	*SLC24A4*
WDR72	*ITGB6*	*ODAPH*	*COL17A1*	*LAMB3*	*GALNS*	*GPR68*
ACPT	*LAMA3*	*ODAM*	*AMTN*	*LAMC2*	*ITGB4*	*CNNM4*
FAM20A	*PEX1*	*PEX2*	*PORCN*	*LTBP3*	*DLX3*	*ORAI*
STIM1	*GJA1*	*ROGDI*	*AIRE*	*NHS*	*SLC4A4*	*SLC13A5*
DSPP	*CLDN1*	*CLDN16*	*ALPL*	*GPR98*	*RAI1*	*VDR*

Table S1. List of AI Candidate Genes. While the entire exome was analyzed, AI candidate genes were also specifically examined manually. No potential AI-causing mutations in the proband were observed in these AI candidate genes except *AMBN*.

	Forward Primer	Reverse Primer	Amplicon size
Exons 1-4	5'-TACAGAGAGAGCCCCAGGAA-3'	5'-TGAGAAAGTGCGTTGAGTCC-3'	292 bp
Exons 1-11	5'-TACAGAGAGAGCCCCAGGAA-3'	5'-TCCCTTGGGTGAATTCTGAT-3'	WT: 932/887 bp;<br>null: 749/704 bp.
NLS-lacZ	5'-TACAGAGAGAGCCCCAGGAA-3'	5'-GAGGGGACGACGACAGTATC-3'	403 bp
Gapdh	5'-AGGCCGGTGCTGAGTATGTC-3'	5'-TGCCTGCTTCACCACCTTCT-3'	530 bp.

Table S2. Oligonucleotide primers used to check for *Ambn* expression in *Ambn*^{lacZ} mice by RT-PCR. DNA sequencing confirmed the exact sizes of the amplicons (see Fig. 2).

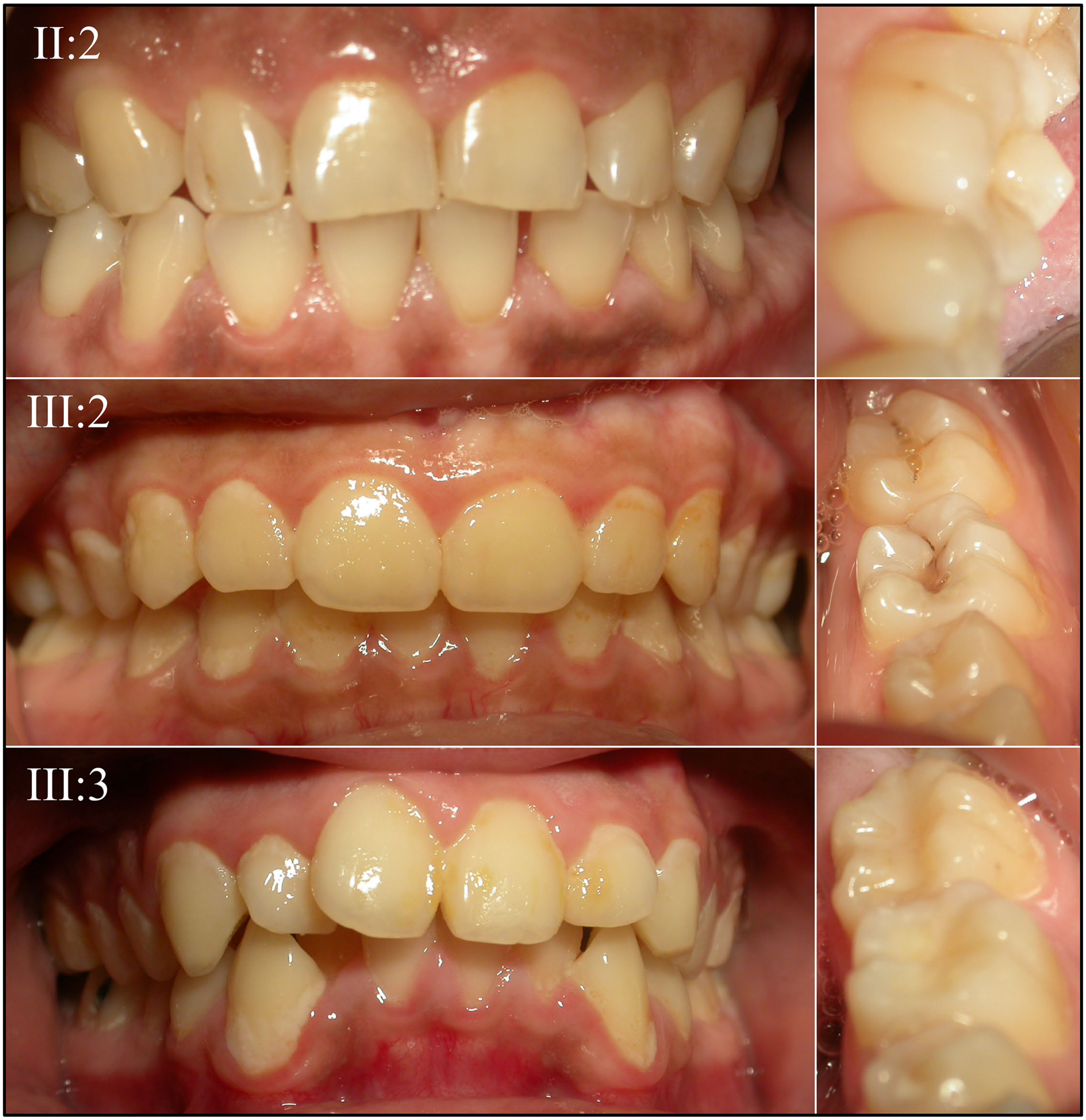

Figure S1. Clinical Photos of Unaffected Family Members. The proband's mother (II:2) (age 38) and half-sister (III:2) (age 18) were heterozygous for the *AMBN* NG_042078.1:g.19190T>C; NM_016519.5:c.1061T>C; p.(Leu354Pro) mutation and exhibited very minor enamel pitting. The proband's brother in law (III:3) (age 20) had no *AMBN* mutation.

Figure S2. NCBI Reference Sequence NG_042078.1 for Homo sapiens *AMBN* on chromosome 4 showing its intron-exon structure and AI-associated mutations. The exons are highlighted in yellow, with the corresponding amino acid sequences underneath. AI-causing mutations are highlighted in red. The AI causing AMBN mutations include:

1) *AMBN* 2347 bp deletion causing AI (c.294+139_531+478del; p.Tyr99_Glu177del) (Poulter et al., 2014).
2) *AMBN* splice junction mutation at the end of Intron 6 (c.532-1G>C; p.Leu178_Ser190del) (Prasad et al., 2016).
3-4) *AMBN* compound heterozygous missense mutations causing AI in this report: NM_016519.5:c.1061T>C p.(Leu354Pro); NM_016519.5:c.1340C>T p.(Pro447Leu).
5) The *AMBN* single allele mutation NM_016519.5:c.1069C>T; p.(Pro357Ser) was reported to cause a dominant condition with enamel and dentin defects (Lu et al., 2018), but the clinical phenotype exactly matched that of dentinogenesis imperfecta, a dominant condition caused by mutations in *DSPP*, a gene linked to *AMBN* on chromosome 4 that contains a large repetitive region that cannot be characterized by whole exome sequence analyses (Yang et al., 2016) (not caused by *AMBN* mutation).

Exon 1: 116 bp (Gene NT 5001 to 5116; cDNA NT 1 to 116; cDNA coding NT 1-15); 5 amino acids (Met[1] to Lys[5])

GAAGTACAGAGCAAGTCCCACGCACAGTCCTGAAAAAAATTTTAATCTTCTTTTCTTAGAACTATCTTGGTTG
GCATCATCAGGCCCTGAGAGCACAGTGC**ATG**TCAGCATCTAAG
M S A S K

Intron 1: 953 bp (NT 5117 to 6069)

GTAAAATGGGATTTTATGATTTCCATGTGTTTCCTGTAAATTCTTTTTTTCCTATCTCCTGACAGCTTTTATAAAGAGGATTTATCTT
CATTTGTCACCAGTAGCTGAATTATTGCTTGTTGTAATCCATTAGCATGTCCCAGAGATGACATGCCTAACACCCATTCAACTAAGAA
AAAAAAAAAGACTTTTATTTATAGATTGTGTTTTTTTTAATGTTTTTGATTATAGTGCCTAATTCTTTTTAATATTAGAAGATGAGTA
AATTTCTAGGCTATTCAGATCTCTATTCATGTAAGAATAAAGAGCTTTTTGAATTTATAGAATAAATTTTAAAAGATAATTCATTTTA
GGAAGAGGAAAGCTAAATTTGAGACATATTTTCCACTAACATCAAAGAAAATTAAGATAGCCAATTTACTCTCATTTGAAAAGAAAAA
TTTTGTCATGGTTACGTAGATTACCTAAACATGCAATGATGTACCCTCTATTCCTGCAAATAAAGGTTTGTTATAAAATGACTTAGTT
GCATTACATTTCACATGCATTTCAAAATGTGTCCCAAAGGGTAAGCAAAGTATAAACTTCTGTGAATAATACCTTATAGACCAATATT
TTACTTAGTGGGAAGGATTTTTAAACTCATAATTCACTTTATGAAATGAAATATATCACAAAGAATATCGATGGAGGTTGTTCCTACA
TACTTATTTAGGCTGTAGATCCATTTAAAACGTGCTTAGATATTTTTTAATTGCTTGTTAATTTATTTCAGAAACTACTACATACAAA
TCGGTTGTTTAGTAACATTTAGTTTTTGTTTTTGTTCTTCTAACCTTTATCCCGGTGGTTTTTGTAAGAGCAGAGACTCAGGCTCATT
TTCAAAAATTGTCTTTTAACCATTCTGAATAAAAATTCATCTGATTTAATTCTCCAACTTAATTATGTTTTAG

Exon 2: 69 bp (Gene: 6070 to 6138; cDNA 117 to 185; coding 16-84); 23aa (Ile[6] to Pro[28]) Signal Peptide cleavage is after Ala[26].

ATTCCACTTTTCAAA**ATG**AAGGACCTGATACTGATCCTATGCCTCCTGGAAATGAGTTTTGCAGTGCCG
I P L F K **M** K D L I L I L C L L E M S F A V P

Intron 2: 3603 bp (NT 6139 to 9741)

GTAAGTCAGTCTTTAGAGTGTGTCCCAGAAAAGGTTATTGATTGTCGAACTCCAAACAACACTGAAGGGATATGGACTGAGAGTCCAC
GCATAGACTCACTTGATTTAATCCAGTGGTTTAGTCCTATTGAGTCAAAAGCCATAGTTGCTCAGATGTCAGTCTTGCAATCCAAAAG
CGGTAAGTGATAGCCGGGTGCGGTGGCTCACGCCTGTAATCCCAGCACTTTGGGAGGTTGAGGCAGGAGGATGACCTGAGGTGAGGAG
CTTGAGACCAGCCTAGCCAACATGGCTAAACCCTGTCTCTACTAAAAATACAAAAATTAGCCAGGCGTGGTGGTGGGCACCTGTAATC
CCAGCTACTTGGGAGGCTGAGGCAGGAGAATTGCTTGAACCTGGGAGGCGGAGGTTGCAGTAAGCCAAGATTGTGCCACTACACTCCA
GCCTGGGTGACGGAGTGAGACTCCATTTAAAAAAAAAAAAAAAAAAGCAGTAAGTGAAGTATGATATTTCTACTAAAAAGTTATTTAAA
ATAGTCCCAAGACAATTTCTAGATCTTATTCCACTCATTCAAAGTTAAAAGCTTAGATAGTCTCCACAATGCATGGCGAAATTTTATT
ATCAACTTATTGCTATCATTATAAAGTTGAAATGGAAAGAGGAAATTTCTTTGAAAAAAATACATTTTCTTCATTTCAAAAAGAACTA
TCTCTAAATTTCTATGGTTACAAAACTCAGCTAGATAATTCCATTTAAAGCTAGTAATTAACTAGCAAATTTACCTTGTAAACATGAA
ATTGTGTGACTGAGAGCTGAAGTTTCATAAATCAAAACATATCATAGTGATGGGGCTAATTGTGTATTACAGTCCCACACCTGATCCA
TTCTAGTATATATTGAAATGATCTCATACAAACACAATCTTTTGAACACTGAACTACTCCAGCCAACAGCATGCAATTCTGTTATCAC
AAAAACTTTATTTGGCTGCTATATGCATTTAGCAAATATGTTGAGCAGCTGCCATGCATCCAGTAAATAAGACATGGAAAAATGAAAG
TAAATAAGACATGATCTCTTGTCTCCAGTAGCTCACAGTTGGCCTCAAATGAACTTTAGAGTATTTTAATTCCTTGAAAGAAAAGGTT
AGCTACTTGGTCTTCTTAAAGTGTTTTAGAGAAAGAGAATGGAAATGGAGAAAGAGGTTGAAAACTGATTATAATGATGAAAACCTTA
ATTCCACAAATAAAAATAGTCTCTGTTCCATCACGCAAGATGAATTTTAAATGAAAGAAAAATTGAGGGTGCCACCCTAGCTTACCTT
TATATGATACTGACGTTGGGATGAGCCTCAGTCATTCACTATCAGTCTAACACATTGGCATTCTTTGAACCTATAATGATCCACACCT
GAATCAATTGGGTCCACTCTGAGGAACAGAACAATTGCTTATTATTGCCAACTCATTGCCTGTCAAATCAGATGTTCTTCTCCTATTA
ATTCTGCATGGGAAATGTATCCCCTATCTGTATACATTCGTGTAAATATTCTTTAAGTCATGACTTAGAGCAGATGGAAAGCAAAGTA

CACTTTGTTCTGTGGCTCAATCCAGGATGTGTTATTTATGCACTGGCAAATAGTGGGGAAGAAGGTCCTGCTCTCACGATTCCCCCAA
ATTCTCAACAAGCTAACCACCACATACACACATACATGCACAATCACTCTTCCTGTCTACACAGCTAACCTCTTTCCTACCCAGAGTA
TTTGCTACACATTTGTAATGCCTTTCCCTCTATTCTTTTTTTTTTTTTTTTTTTACAGTCTGGCTCTGTTGCCCGGGCTGGAGTGCA
ATGCTGAGATCTCAGCTCACTGCCACCTCCTCCTTCTGGGTTCAAGCGATTCTCCTGCCTCAGCCTCCCAAGTAGCTGGGATTACAGG
CACGTGTCACCACGCCCAGCTAATTTTTGTATTTTTGTAGAGACGCGGTTTCGCCATGTTGGCCAGGCTGATCTCAAATTCCTGACCT
CATGATTTGCCCACCTCAGCCTCCCAAAGTTCCGGGATTAGTCTTGAGCCACCATGCCTGGCCCTTTCCCTCTATTCTTGTCCAAAAC
TGAATTATTTAAAGAAATTCTCACTATGACCCAGAAGGTGGGATTTTACTTACATGAAAAAGGTGGTGGGTGGAAAAAGTAGGGGGTG
TAATTCTTTAACTTCTTACTCATAAAGGAAAAATAATACTGATATATTTGGTAAACATTCCCTAAATCCTTGTGGTATAACTGAAGGA
AAGACGGCATTACAACCCCTTGGTCTTAGAAAGTGAGAATATCCAAAGAACCAGAGACTCTCAAAATCCAATTTACTCTAATTCCTAC
TCTTCCATTCGCCATGTTTTCTACCGGTATTTGTGTAAAGTAATAAGGATTGGTTTCAAAAGAAAAATATATTTAACAAAAAAACAAA
AAACAAAAGGAAAAGGCTAGGCATGGTGGCTCATGCCTATCATCCTGGCACTTTGGGAGGCCAAGGCAGGAGGATTACTTGAGCTCAG
GAGTTTAAGACAAGCCTGGGCAACATAGTGAGACCCCCATCTCTATTTTTTAAAAAAGGAAGCCAGATGCAGTGGCTCACATCTGTAA
TCCCAGCACTTTGGGAGGCTAAGGTGGGCGGATCACTTGAGCTCAGGAGTTCAAGACCAGCCTGGCCAACATGGTGAAACTCTGTCTC
TACCAAAAATACAAAAATTAGCCGGGCTTGGTGGTGCACACCTGTAATCCCAGCTACTTGGGAGGAGAATCACTTGAACCCAGCAGGC
AGAGGTTGCAGTGAGCCGAGATCACGGTACTGCACTCCAGCCTGGGTGACACAGTGAAACTTAGTCTCAAAAAAAAAAAAAAAAAGGAA
AATATCTTACATTTTAAAAAGCTAATTGTATAGATAGGTATATTTTTGAACAACTTTTAAGTGAACACATTAAGTATAATAGTATTTT
GTGCTAGAATGCTTTTTATTTAAGGGAAAAAAACCTTTGATATGATCCCAAAGTATAGTAGGTTTACACATAAAATAATCTTTCACTA
AAAATGATAGGGAATTTGCTGAAGCCCTTTGAATGGGAATCCAGTTAGATTTTTCATATGATTTTTCTGAATGTCATGATCCCATGCC
ATGTCTTGTAAACCAAAATCATATAGATTTGTCGAAAGCACATGAAAATTCAGCTTGGGTTTGTCCCCTTTACTTTTTTCTGTGGTAA
TTTTTAAAGAAAATAAAGTACTAGTAGTTTTCTATTTTGTTGCTTTATTCTATTTCTCCTTGTGTGTAACTTTAAATCATATTTTGCT
TTTAAAGCAATGAAGCATTAAGATGCAGACAAAGGATAGATGATGCAGGTGGGAGCAAAATTATCAAATACTATGTTGATTCTCCCAA
ATAACTTATGCTCTTCTTCATTCTGCCCAAGCACTCAAGTCATTTGCATAATAAACCAAAATGGTGGCCTCTCAGGTAGCCCGTATGT
ACTGGAGCAATATCCTACGCCCAATGGGCATTGAAGGAAGTTTTGTACCAATAAGACTCAAAATTATTCTTATTTTCATTCAG

Exon 3: 51 bp (Gene 9742 to 9792; cDNA 186 to 236; coding 85-135); 17 aa (Phe[29] to Glu[45]).
TTCTTTCCTCAGCAATCTGGAACACCGGGTATGGCTAGTTTGAGCCTTGAG
F F P Q Q S G T P G M A S L S L E

Intron 3: 1306 bp (NT 9793 to 11098)
GTATGTATTGTCAGAATTTTTAACATATTAACTTTACATTGGTATATTTGTGGTACAGGAGAAATATAATCGTGTGCTTCTCAGTTTT
CCTAGCCAGAAGTTTGTGGCTGTTATCCTGAGGAGTGCTAGAAAAGTTATGACATTTACTAGAGCTTTGTCTTATAATTCTACTGCAG
TTTTAAACTCTCAGGTGTTTTAAGTTTTAGAACATTGTTTCTATGCAAACCAAAGCAATATTAGTATTTAATGCCTTATATATAATAT
GCACATTAATAAAATTACTACCTTAATTAAAATACATTGCTCCATACTCACAAATAGTTTATTTTTAATCCTGCTCCTTATACTATCT
TACATGATAAAGTTCTCATGAAAAATTTTGTACCTGTACAAAATTTAATTTTTTATAAGTCTGGTAAATAACATTAACATTGTACTAT
GATACTTCCTGCATAAGTTGTACAGTGTATTTGTCTTATTGCAGTTCTCATGTAATATATATTCTGTTTAGAAAACTCCAGCAAAACA
GAAATTTTGGTCATGTCAATGAAAAATATTGACAGTTATATATAGAAAGATATTTATAAGATTATAAATTTTTCACAAGGTCTTGTGA
AATTTCCATGTGTCATGGTAAGCTGGGGGCAGTCAATACCTATTTGAGCTAGACGCTTATTTCACATGGAAGCTAATTTCGCTGAGGG
TTTTTTTCTCACTTTTTAGCTTAAACTCAATCCAAAGCATTCTAAATCTCTTCCCGTGAAAAAAGTTAAATTGGCATTCTGTTGAAAT
GGTAGCATTCTCGATAATCTATCCAGATACTTAACATTTCTTTCAATAGTTTTTATTTTTAATATTTTGAAAGATTTATTTTATGGGA
AAGAGGTAGAAGTTTTATTTGTTTGTCTTGTTTGTTTGTTTCAAATTCCTCTTCTGGCTGAACTTGTAAAGGGCAAAGTAAATTAAAT
GGGGAGAAAGAGTCAATGAAGCAAAGTCCTCACAATTACACTTATATTTCAATGGAATGAGCTATTTTCCCATGTTTCAATGTAACCT
CCACTTAAATAATACTTTTTCTCCTTTAATTAATGATAAAAGCAACTGCATTATTTTAATAGCAATAATAATAACAATGGCAGAACCA
AATTTAATATTTCCACCTTTCAGTGATGATTTGTGTCTACTTTCATTTTATTAAATTGATTCACACCAAACTTTGTGTTGATAATGTC
AAATATCATGAGAAATCAGTTGTGTCAAACACAGCATATGCGATAAACAGTAACCCACTTTTTTTTCTTGATAG

Exon 4: 48 bp (Gene NT 11099 to 11146; cDNA 237 to 284; coding 136-183); 16 aa (Thr[46] to Gln[61])
ACAATGAGACAGTTGGGAAGTCTGCAGAGATTAAACACACTTTCTCAG
T M R Q L G S L Q R L N T L S Q

Intron 4: 1132 bp (NT 11147 to 12278)
GTAATCATATTTCTTATTGCAAGTATTCATGGTGGTGGTAGTGTTAATATTAGCAGCAGCATTATAATTCATCAAATTTATTTATGAA
TAGACAATATACTAAAGATTGGAAGAACCAAGAAGTAAATGAGAGCCTAAAACAAAATAAGATACAGTGACAGGGAAAAACAAAAACA
ATATTGAATTTTATTGAGTTAAGAAAAGAATCTTTTCCTGCATTATTAATTTTATTATGTGGCACAAAACACAAGTGCTTAAAAAGCT
AAATCTCAAACAATTTATGCAAATATTGCAGACTAGCTCAAAACGAAATTTAAGTTCATTGAAAGGAAGAGTTCACCTACCCATTTGA
TTTACTTATTTTATTTTTATTTATTTATTTATTTATTTAGACAGAGTGTCGCTCTGTTGCCCAGGCTGAAGTGCAATGGCATGATCTC
GGCTCACTGCAACCTCCATCTCCTGGGTTCAAGCAATTCTCCTGTCTCAGCCTCCCAAGTAGCTGGGATTACAGGCATCTGCCACCAC
GCCAGGCTAATTTTTGTATTTTTTAGTAGAGACGGGGTTTTGCCATGTTGCTAGGCTGATCTCGAATGCCTGACCTCAGGTGATCCAC
CCACCTCGGCCTCCCAAAGTGCTGGGATTAGAGGCGTGAGCCACCACGTCCAGCCATACCTATCCATTTTAAATTAACCAACATCACT
TTTGCTTTTGCTATATTTATGCTATGTGTTGATTTTTTAGAAAATGAAAACCAGGCCAGGAGCAGTGGCTCACGCCTGTAATTCCAAC
ATTTTGGGAGGCCAAGGTGGGCGGATCATGAGGTCAAGAGATCAAGACCATCCTGGCCAACACGGTGAAACCCCGTCTCTACTAAAAA
TACAGAAATTAGCTGGGCATGGTGGTGCGTGCCTGTAGACCCAGCTACTCAGGAGGCTGAGGCATGAGAATCGCTTGAACCCGGGAGG

TGGAGGATGCAGTGAGCTGAGATTGCGCCACTGCACTCCAGCCTGGCGACAGAGCAAGATTCCATCTCAAAAAAAAAAAGAAAAAGGA
AAAGGAAAACCAAATATAACCAATGTTATATTTAACATTTAAATATAAGCATGTCTTTTTTTATCCATGTCTTTAG

Exon 5: 111 bp (Gene 12279 to 12389; cDNA 285 to 395; coding 184-294); 37 aa (Tyr[62] to Gln[98])

TATTCTAGATACGGCTTTGGAAAATCATTTAATTCTTTGTGGATGCACGGTCTCCTC
 Y S R Y G F G K S F N S L W M H G L L

CCACCACATTCCTCTCTTCCATGGATGAGGCCAAGAGAACATGAAACTCAACAG
 P P H S S L P W M R P R E H E T Q Q

Intron 5: 1771 bp (Gene NT 12390 to 14160) Red highlight indicates *AMBN* 2347 bp deletion causing AI (c.294+139_531+478del; p.Tyr99_Glu177del) (Poulter et al., 2014).

GTGAGTGAATAGCATCAATATGTTTGAAACCTCAGGCTTTAAAACCTCTTCTTACTTGCCTTCTTTAAGTTATGATATATTAGAAACA
GCCAAGTCCTAGCATTAACAAAAAATTATTCTCGTGCCAAATAAATCGAAGCCTATAACTGCTTTTAGCGCTTAAGAAATATTTTTAA
ATTTAAAACTATTTGCATATTTATTTAAAATAGGAAGATACAGCCTCCAAAATATGAGACCTATATTAAAGATATCAGTAAACAATCC
TCGAAAATCAAAGCAGGCTACATCCTGGACAATTTTCATTTGAATCAAGATTTCCTAAATAATTCCTCAAAGGCTGATTTCAAATGAT
GACAATAATCCTGGTTAAAATACCATAGCCAGGTGCGATGGCTGACGCCTGTAATCCCAGCATTTTGGGAGGCCAAGGTGGGTGGATC
ACTTGAGGTCAGGAGTTCAAGACCAGCCTGGCCAACATGGTGAAACCCCGTCTCTACTAAAAATACAAAAATTAGCCAGGCATGGTGG
CAGGCACCTGTAATCTCAGCTACTCTCAGGAGGCTGAGGCAGGACAATAGCTTGAACCTGGGAGGCAGAGGCTGCAGTGAGCCGAGAT
TGTGCCACTGCACTCCAGCCTGGGCAATAGAGTGAGACTCTGTCTCAAAAAAAATTAAGAAATTTAAAAAAAAAAAACATCATGAACC
CTACAGAAGTCAATAAATATATCAATCTGTTAACCATTATTGATTAGGTGCCTAATAAATACATAAACCAAATTGTGGTTTGGTAGTA
AAAGCGAAGACATTTAAGAATGGCTCCAAGGATTGTGAAATTTCATCTTATCTGATTCAGATAGGACAAGACAACATATTTCAAAGTG
CTGAATTGTGACATGTGAGTAATGAATACTGCGTGAGCTCAGAGAGGGGTGATGGCTGGAGAGGCAAAGCAGAGCCCCAAATTGTTGC
GAGACTTCCTTGCTGGTCTGAGGAGTTTACAATCAATCTACAAACAATAGGGAGCCATTCACCAAAAGTAATTTCTACAAGAAGTAAC
AAAACATGGTGATTTTAACGGGGAATTTTTTCGTTTAATATATGGACTCAGAACCAAGATTTAACAGGGAATTTTAGTTCCAGGCGGT
CTTCCACAGCAGGAGCATTACCACGAGGTCACATTCAGTAGAACAATAAATTATGTTAATGGTGACTGTTTCTTAACTTAATTGTTTC
CAAAGTGGACTCCATGATGCAAGAATTTGATTGGCATTTTAATTAATTACCCTTTATCCTTTCATTAACTCGAAAAATATATTAGGCA
CCCACCATGGGCCAGGCACTTGTTAGGAGCTATGTTATGTACACGGGCTATAAAGAAAAACAAAACATATCATTCTCTCTCCGTTGAC
TGGGGGACAAAGATTTATAACAAATAATTATCATCCAGTGAGAAAGTGGTAAAATAAAGATACAGATACATACAGACAGCTATGGGAA
CTTAGAGAAGGACATGACTCATCCTACCAGGAGGTTCATGTAGCTTCACCTTTACGAGCAATGGTGGTCCCAAAGGAGGTAGGGGAGG
CACACACAAGACCTCCCCAAACAACATTAACACTAACTGGTGCTTGCTATGTAAACTCAACTTCAAGACAAGTCTCCTAGCCTCCCTT
CCAGATAGAAAGCGCCCCAAGCCCCTTTGTTTAGAAATTCTAGGCACCGTTGTTTAATGAGCCATCCCTTCCTAACACTCTTTTCAAA
TTTCTCTGCAG

Exon 6: 237 bp (Gene 14161 to 14397; cDNA 396 to 632; coding 295-531); 79aa (Tyr[99] to Gln[177])

TATGAATATTCTTTGCCTGTGCATCCCCCACCTCTCCCATCACAGCCATCCTTGAAGCCT
 Y E Y S L P V H P P P L P S Q P S L K P

CAACAGCCAGGACTGAAACCTTTTCTCCAGTCTGCTGCTGCAACCACCAACCAGGCCACA
 Q Q P G L K P F L Q S A A A T T N Q A T

GCACTGAAAGAAGCACTTCAGCCTCCAATTCACCTGGGACATCTGCCCTTGCAGGAAGGA
 A L K E A L Q P P I H L G H L P L Q E G

GAACTGCCTCTGGTTCAGCAGCAGGTGGCACCATCAGATAAGCCACCAAAGCCTGAG
 E L P L V Q Q Q V A P S D K P P K P E

Intron 6: 969 bp (Gene NT 14398 to 15366) The single nucleotide highlighted in red at the end of intron 6 was reported as a homozygous splice junction mutation causing AI: c.532-1G>C (p.Leu178_Ser190del) (Prasad et al., 2016)

GTACTTCCTTTCTCTTGAAGTCAGATCAGAATCACCAGCTAACCTAATAGAAGAAAACGAATTTGCAGGGCACTTTTAAATAATCGTA
GCCTTCAGGCATAATACGTGATATAAATATATTCTTAATCAGTCTTCCACATCTGAGATTTATTTTTTGAGTAAAACGTAAATATCCA
TTCCTATCTTTTGCCATCAGAGACAGGGCATGGATTCTAAAAGATACTGTGCCTATAAAACTGGGTTTCTTAGTTGCGAAAGATGAAA
ACTCCCATAGGAAATAGAATAAAACCCCAGAGAAGTCCAGGTTTCCTATCACTGGTCTTTTTTGGTATTGGAAAAACTGAAAAAAGTG
ATTGATCCCTCAGCATAACAAGTAGGATTTTGCATTATTATCTCTCTGACAAAGAGGTGACCACTATGCAAAAAGTATCTTGTAATAA
TGTAGAGAAAAGTGTAATGGATTAAAAAAAAAAATGAAGGTACCAAGTAACCAGTATGTTGTGGGCATTTATCATTCCCCCATATCACC
CTGCCTACAATCCTATAAAAATATATATTATCAGCCTTTTTAAAGAAAGGAAGACAGATTTTGAGAAGTTACAAAATTTGCCCAGGAG
TACACAAAAATTAAGTGGCAGAGCCAGGGTTCAAACCAACCAGGTATGTTCCAGTTCCAAAATTCATTACTTTCTAATCTGCAAGAGA
TAGCAAAGCCATGATAGATGTGCCTCATCTCCCTGCAGGTACATTTGAGTAATGTTAGTAAAAGACCATTGCAAACTCTTGGGTCATA
CCTCCCAAAACACTAAAAGAAAGTAAAATAATGAAACTCATTTTAATTGTTTTATTGTAATTGTAAACAAGATAAGGATGTGATCAGT
TCACTTTGTCTATTTTTTTTATTTTTAATGTCACTTTGTCTATTTTGTTTATTTTTTGACTGATAATTTTAATATTTATCTGTGATATA
G

Exon 7: 39 bp (Gene 15367 to 15405; cDNA 633 to 671; coding 532-570); 13aa (Leu^{178} to Ser^{190})
CTCCCAGGAGTAGATTTTGCTGATCCACAAGGTCCATCA
L P G V D F A D P Q G P S

Intron 7: 135 bp (Gene 15406 to 15540)
GTAAGTACAGATCTCAATGAGACACTTTCTGTATTTTATTTTTTAATTTTTATTTGTTCACTTTTTTATTTTTATTTGTATTTAACTA
CTTTGTTCATTTTTTTACTGATAATTTTAATATTTATCTACAATATAG

Exon 8: 39 bp (Gene 15541 to 15579; cDNA 672 to 710; coding 571-609); 13 aa (Leu^{191} to Ser^{203})
CTCCCAGGAATGGATTTTCCTGATCCACAAGGTCCATCA
L P G M D F P D P Q G P S

Intron 8: 135 bp (Gene 15580 to 15714)
GTAAGTACAGATCTCAGTGAGACACTTCTATTTTTTTATTTTTATTTTTATTTGTTCACTTTGTCTATCTTTTATTTGCATTTATCTAT
TTTGTTCCTTTTTTGACTGATAATTTTAATATTTATCTGTAATATAG

Exon 9: 39 bp (Gene 15715 to 15753; cDNA 711 to 749; coding 610-648); 13 aa (Leu^{204} to Ser^{216})
CTCCCAGGATTGGATTTTGCTGATCCACAAGGTTCAACA
L P G L D F A D P Q G S T

Intron 9: 249 bp (Gene 15754 to 16002)
GTAAGTACAGATCTCAATGAGACACTGTCTTGCAAAAACTGTCTCTATATTTCAAAAAATAAGAGTGAATTTTTTCATTGTCCCATTT
ATCCATGAATATGAAGTATAATGGGTTCCTTTGTTCTCTTAAATATTAAATACTTATTTTCTATACTAATCCATCTGAAAATGTGTTT
CATATACCTCAAAAATTACTGTTATGGGGATGTGCCTGTGAGAATTACTTATTCTGTTTTCTACCATTTAAAG

Exon 10: 60 bp (Gene 16003 to 16062; cDNA 750 to 809; coding 649-708); 20 aa (Ile^{217} to Pro^{236})
ATTTTCCAAATAGCCCGTTTGATTTCTCACGGACCAATGCCACAAAATAAACAATCTCCA
I F Q I A R L I S H G P M P Q N K Q S P

Intron 10: 96 bp (Gene 16063 to 16158)
GTAAGTTTTTTTAATACCACATCTCTGTTTGCAATTTACTTAAAAGTTTTTCCATTGGAAAATGCATTTTGTGATAATGATTGTATT
TTATTTAG

Exon 11: 45 bp (Gene 16159 to 16203; cDNA 810 to 854; coding 709-753); 15 aa (Leu^{237} to Leu^{251})
CTTTATCCAGGAATGTTGTACGTGCCTTTTGGAGCAAATCAATTG
L Y P G M L Y V P F G A N Q L

Intron 11: 416 bp (Gene 16204 to 16619)
GTAAGTCCATATTCTATAAAAAGTATTGTTTTTAATTAAATTTTATCTTAAGAGCTAAAATTCAAAAATCTAGGTCTCACACAAGAGA
TTCCAAATAAACATTCTCTGAACACCTACATTAGTACTGAAATATTAAGGAGGCAAACTTCCTATTTGGATAATCTTGGCCAGACACA
CACACACACACACGCACACGCACACACACGCACACAAGGGTATTAAAGAAAAAACAAATTTTAGATCTGAATTATCCGAGTTGACTAC
ATTTATTCCATAATGACTCACCTCTCCACTTTGCCTCTGTACACAGCATTAATATTAAGGTTTAAGTGAAAACTAAATAACTTTTAAC
TTTGTCTTGAGTAGATAAGAAGGTAGCTGGTTCGTAATTTGACGCAATATTTCTTTTTGAACAG

Exon 12: 45 bp (Gene 16620 to 16664; cDNA 855 to 899; coding 754-798); 15 aa (Asn^{252} to Ala^{266})
AATGCCCCTGCCAGACTTGGCATCATGAGTTCAGAAGAAGTGGCA
N A P A R L G I M S S E E V A

Intron 12: 2263 bp (Gene 16665 to 18927)
GTGAGTAATGTCTTCTAACTCTTCTTAAAATAGTGGCCAGGGAAGAACAGTCACTTATGACTGGCTGCAAGAGACGTCTGCCATGAAT
GTAGCCAAATGAGCATGGGACTCAGATTTTTCCACTCCAAAAACTAAGCTTAATTCTGAAGCAATATCCAAAGATTTCATCATGTTTC
CTATGTAATCTTTCATTTCTGGGTGATGACTATTCTTGAGCATAGAGTTTTGCCCCATTGCTTGTAGATGAACAGAGGAGAAAACTCC
CCTTGAAAGGCCGTCATTGAACTATAAACACTTCAAACCAAAAGGATACACGTGGTTCATACAGAGTTCTTGTTCTTTCTGGGACCAT
TTAACCCCTCAAAACATCAAATTCAACAGCATTACAAGTAATTCAGATCTCAAAATTGAAAGATCAAAAAGCAAAAGAAAACCAAGTT
TAAAATGTGATTATTATAAATGTCAATGTGCATTGTCAATGAGCATATACTCTTATTATGTTATCATTATCCATGCAAGTTTAAGATT
TGAGATCAATAAAGTCATACAACTTGACTTTTTCCCTAAGAAAATTTTTGCCTGTTACATCTGTTAGAGCACAAGGCAAGACAAAACA
AAAGCTATATTGAATTAACAAATCACTGTCAACTACAGATCCTGTCAAGTTAACAAACACACACACATATTATCTGTGAAAGAAAAAT

TACATCTCAGTATTTTTGTGTGCCTTCCATGTGCCACACACATAGGAAAATACTGTAAACAAGACAAGGTAAGATTTTCGTTTTTGGC
TGGGCGCAGTGGCTCATGCCTATAATCCCAGCACTTTGGGAGGCTGAAGCGGATGGATCACCCGAGATCAGGAGTTTGAGACCAGCCT
GGCCAACATGGTAAAACCCCGTCTCCACTAAAAATACAAAAATCAGCCAGGTTTAGTGGCGCGAGCCTGTAGTCGCAGCTACTCAGGA
GGCTGAGGCAGGAGAATCACTCGAAACTGGGAGGCAGAGGTTTCAACGAGCTGAGATCACGCCACTGCACTCCAGCCTGGGCAACAGA
GCAAGACCCTGCCTTAAAAAAAAAAAAAAAAAGTCTTGTTTTCAAGGAACTTACAGTCTAATGAATGGAAAAGATAGGTACAAGGGCAG
ATACCTAATGTCAAAATCATGGGCTAAAAGTAATAACAATTTTATTAGAGAGAAAGAGTCAGAGAGGAAGGGCAGTTAGCCCAGTCCA
GGGATTCACAGTATCATCCCTGGAGGAGATGATGTGAAAGCTGAGAGAGAATGAAGAGTCAGGAGAAAGGAGAAGGAAAGAGTATTGT
ATCTATATAGCACCAATACAAGTGGCTGGAAGTTCAAAATTCAAGTAAGTGTTAATTGCCATCACATAGTATTAATTATTTCTATTCT
GCCTCATTCCCAAAAGATTTTGTAGAGCTTATGAAAAACACACAATAAATATAACAAAAAATTGAAAGTAGGATTTAAAATGAGTCAT
AATTTCAAGAAAATCAGAATACAGATATGCAAGTCACACAGGCCTGCTCAATTGCTACAAACAGCCTAAGATTTTGGACCCCATATTT
CTGGCAGCAAAGGTAGAAAAAGAAACATGGTCAGTGCTGGTATTCTATTTGTCCACAAGAAGAAAATATTTTAGTTACTCCAGGGAAG
TCACATTTTTTTTCTTAAACTTTGTGCTTTAAAAAAATTAAAGTCTTAGCTGGGTGCAGTGTCTCACCCCTGTGATCCCAGCTACTAG
GGAGGCTGAGGCAAGAAGATCACTTGAGCCTAGGAGTTCTAGACCAGCAGCCTGGGCCGTATAATGAGACCCCTAAAAGAAAAAAAAA
GTACATGGAACTTCATTTGGGGGACACTGAAAAATGCCATTGACAAGATTCTCGCTCAACAAATGCAGTTATGAATTTCAGTTCTTAT
TTGTTTTGTGATGGTAGTACTCATTTCAACTGCTTGTTTTCTTAAAATTAAATTCAAACTCACCTTTGCCTCAAGTCTTGCTTCTGCC
CTGTTATCTCCCTTTTACGGAAATCATGCCGAGATCCACCCAGATAAGTGCAGAATCCTCTTCATTATGTTTCAAAGCCAGTTTAGTG
AGAAATGAGCTTACACAAAGCTATCAATCAGCCAACTTCCTATTCTCCACCAGTTTTTTAGAGATCCTTGGTGAATGTGACCAGGTAT
AGCTGCATGGTATAGTTAATAGCATGTGATGATGGCATCTTTGACGAATGTTTTTTTTTCCAG

Exon 13: 1104 bp (Gene 18928 to 20031; cDNA 900 to 2003; coding 799-1344); 181 aa (Gly^{267} to Pro^{447})
Red highlight indicates *AMBN* compound heterozygous missense mutations causing AI in this report: NM_016519.5:c.1061T>C p.(Leu354Pro); NM_016519.5:c.1340C>T p.(Pro447Leu).

GGCGGGAGAGAAGACCCAATGGCCTATGGAGCCATGTTTCCAGGATTTGGAGGCATGAGGCCCGGCTTT
G G R E D P M A Y G A M F P G F G G M R P G F

GAGGGAATGCCCCACAACCCAGCTATGGGCGGTGACTTCACTCTGGAATTTGACTCCCCAGTGGCTGCC
E G M P H N P A M G G D F T L E F D S P V A A

ACCAAAGGCCCTGAGAACGAAGAAGGAGGTGCACAAGGCTCCCCTATGCCGGAGGCCAACCCAGACAAT
T K G P E N E E G G A Q G S P M P E A N P D N

CTAGAAAACCCAGCTTTCCTTACAGAGCTAGAACCTGCTCCCCACGCAGGGCTCC**T**TGCTCTCCCTAAG
L E N P A F L T E L E P A P H A G L L A L P K

GATGACATTCCCGGCCTGCCAAGGAGCCCTTCAGGGAAGATGAAGGGACTCCCCAGCGTCACCCCAGCA
D D I P G L P R S P S G K M K G L P S V T P A

GCTGCTGACCCACTGATGACCCCTGAATTAGCTGATGTTTATAGGACCTACGATGCTGACATGACCACA
A A D P L M T P E L A D V Y R T Y D A D M T T

TCCGTGGATTTCCAGGAAGAAGCAACCATGGATACCACGATGGCCCCAAACTCTCTGCAAACATCCATG
S V D F Q E E A T M D T T M A P N S L Q T S M

CCAGGAAACAAAGCCCAGGAGCCCGAGATGATGCATGACGCATGGCATTTCCAAGAGC**C**C**TGA**CAGCTC
P G N K A Q E P E M M H D A W H F Q E P *

TAAGATATTAGCTACTTTCTGTATGCACAAGCTTCCCAGCTTTGTCCCCACAGTGTACCTTTTTGCTAAAACA
CTTATTACCCTTCTGCAGCAAAGGCATTAAAAGCGCTAAGCATATATTAATAAATGCAAGTGGCTAGAAATAG
TGTAGGTCCCCTTCTTGCTTTCAATATCTTGTTGAAATAAAATGTGTCAATTGTCTCTGTGATTTAGAAACAC
TATTAATAACATCAGAGCAAGGTTCTAAGGGTCTCAGCATTTGATCATCACTTTTTCTTAGCTGTCTTAAGCA
TTATAGAATTTCTCTTACCAGCATGACACTATTATATTCAGGAAACATGGCACTGCTTTTTTCTCTAAGCAAA
GGCAAATATCCTCATAATTCTAAGCTAATTCATTTAAACTTTATTAAATGGGGATTGGTGGAAAACTCCTGAC
TGGTATTACTGGGTTTGGTATAATTTAATTTAAAATTCTCATTTATAGAGTATTTTATTTAATCTAGTATAAA
AAATCTAGCCTATTTTAAATAAAGAATTTTTCTCACTGAAA

Figure S3. NCBI Reference Sequence NC_000071.6 for Mus musculus *Ambn* on chromosome 5 in strain C57BL/6J. Important structural and functional features are noted. The *Ambn* region deleted in the *Ambn*^{lacZ} knock-in is highlighted in yellow.

Exon 1 (116 bp) Gray highlight shows forward Exon 1 primer site for RT-PCR analyses. **ATG** is a weak, in-frame translation initiation site with a pyrimidine in the -3 position that appears to initiate translation by leaky scanning (Hinnebusch, Ivanov, & Sonenberg, 2016). Translation initiation at the leaky **ATG** near the end of Exon 1 adds 10 amino acids to the signal peptide, which are displayed in lower case.

> …a purine at the –3 position from the AUG both is conserved and functionally predominates over other positions in all organisms. When an upstream AUG (uAUG) is in-frame with a downstream AUG without an intervening stop codon, leaky scanning may occur at some frequency to allow production of two protein isomers differing only by an N-terminal extension, with the longer form often targeted to a particular cellular compartment.

```
GAGAGATACAGAGAGAGCCCCAGGAACAGTCCAGAAAAAAAATTAATCTTCTTTTCTTAGAACTGTTTTGATTGGCATCATCA
GGCCTGGGAGCACAGTGAATGTCAGCATCTAAG
                  m  s  a  s  k
```

Intron 1 (778 bp)

```
GTAAAATGTGATTTTATCATTTCCATGTGTTTGCTATAAATTCTCTTTTCCTGTCTCCAGATAACCTTTACAAAAAAGATTTATCTTCATTTATCACAAGATGCTGAATA
ACAGCTTATTTCAACCATTAGAATATTCCAGAAAATATATTACCATTAACCTAAGAAAATAAACTTATTTGTATGACTTTTTAAAACTATGTTTGTGATTATTAGTGATA
GTGCTAATTTATAAAAGTAAAGAGGATAAGCACACCTGCTGATCAGATCTGCTTCCATTCGAGAGTGAACTGACAAAATGCATCTCAAGATTATTCATTTTTAGGAAGGA
AAATCACTATCCAAGCAGATAAATATACTCAACTTTAGCTTGAAGGCTGAGTTTTGACATGGTTCTGCATTATGACCCAATCATAGGTTGTATTTCAAATTCTGAGTTTA
AACAAAGAATACAATTCTGTAAATGGTGACTGAAGGATGCATTTTATGAAGCAAAGCATAGTGTTCAAGGAATATTCCTGGAGGTTGTTCTTAAACATTCATTCAAATTG
CAGCTTCATAAAAATAAGTACTCAGATCCATAGTTTGGCTGCTCGTCATTCCATTCTGATAGCCACTGGTTTAGCAGCTTTTTGCTTTTGCATTTTAGCACTTTTGCATT
TTTGAGTTTTTTAACCTGATTGTCTTTGGAAGAACAGCCACACGCTCTTAGAAATATACTTTGACCACTATGAATGCAATTTTGCCTGAGGTGATTTTACAACCCAACTG
TGTTTTAG
```

Exon 2 (69 bp) encodes 18 amino acids (16 are from signal peptide) starting at the strong translation initation codon (**ATG**). Use of the weak translation initiation codon in Exon 1 adds 10 amino acids to the n-region of the signal peptide, with the h-region and c-region being added after the strong initiator codon. Both translation initiation codons generate signal peptides that are cleaved at the red highlighted box and secrete identical proteins. Please see Figure 7 from (Hu et al., 1997)

```
ATTCCACTTTTCAAAATGAAGGGCCTGATCCTGTTCCTGTCCCTAGTGAAAATGAGCCTCGCCGTGCCG
 i  p  l  f  k  M  K  G  L  I  L  F  L  S  L  V  K  M  S  L  A  V  P
```

Intron 2: (2405 bp)

```
GTAAGTCCATCCCTTACCCGGGATCTCGGGCTCCTGCATCTGACTACAGCAAACCCAGGGACACTCTGTCCTGACACTTCAGGCATGCACTCACTCAG
TTTGTAGCTCCCAGTGAGTCAATGGTCACAGTGGCCACATGTCAGCCTTATCATCGAAAGGGGATAAGTAAATATAATTTTTCTACTCTAAACAACAA
TTTGGAATATTTCAAAGGCAAATTTTATGTCTTATGACACTCTTTTAACAGGTAGAAGACTACAGCATCCTAGTTTTCATCATGTTTTATTATCAAGT
CCTTGCTAGCACTATGAAATCGAAATGAAAAGGAAAAATATCTCTTGGGCAGGGGGAGTATATGTCTCTTTATATTAGCAACATTATCACATGGCTAT
AGACTGTAGTTCCAGTTAAAGCTAGTATTTAGCTGGCACATTTAGCTGTTCTACTACCCAACAAGAATTTTTAAATGAAAGAAAAATGGAGGCTGGCT
CTCTAACTTGCCTTTGGATGATAACTGAGACTGGGGTCCGCTTCAGCCATCAATCATCAATCTAAAACATTGGCATTCTCTGAATATACAGTGATCCA
CACATTAGTAATTTAGAGTCACACTGAACAATGGGGAAATCACGTGTTTCCTAAGTGACTGCCTGTCAGATCAGGTGTTTCTGCTTTTAATTTTCCAT
AGGGAATGTATCCTCTGTCTATTCACATCCATGTAAAAAATGTTCCTCTTTAACTGGTGACTTGGAGCAGATAGAAATCAGACTACCTTGCCCTGTAG
TTAGAGTACTCCAAAATGTGGAACTAGGGCACTTACCAACAAAGGTCAAGGAGGCTGTGCTCTCATAATACCCCGAACATCTGAACATCCTCCTGTTA
CACACACTGTCATGCACACATGCACATAGGCACACACAATCAGTCCCCTTGTCTACACAACTCATCTTTTTCTCTACCAGAAGTGTTTACTGCACATT
TTTAGTGCATTTCCTTTAACACATACCCACAATGAATTGTTTAGAAAATTTTGCACTGTAATTAAGAAAATGGAGTACTTGCATAAAAAAATTAAGGG
TTAAAAAAAAAGTACAGGGTGTAATACCTTAGCCTCTTACTCCTGAAGGATAATGGCCCCACTGTTCCATACTTGAGGGATGGATGGAGAGGCAGACA
GACAGGTACTTTCAGTATAAATGAAAGAAAAATTACAATACTAACACAACAAAAATGAGGATATCCATGAAATCAGAGAATTCTCAAAATCCAATTTT
CTAGTAGGTAGAGTAACTTATAATTGACCCAGCACATTGGAAGCTGGAAATATTGTGAATTAGAAGCCAGTTTTGGCTACATAACAAGTTCATACCCA
GCCTGAGCTAATTACCAAAACCTTATTTAAAATAAATGAGTAATTTTTGTTTGAAATTTTAAAATTTAATTTCTCTAATTCTATTCTTCCAGGCTCCA
CTCACACTCACACACTGGTATCTGTGCTTATTAACATATGAAAATTAAAGATGTTTAGAAAGAGAAGTATTTTATAATTTTGAAAAGATATTTGTAAA
TATGTTTATGCTTCACAACAGCATCCTACTAAGTACAAGAATACTTTTCATTTAAAGAAAGATCCCCACTATCTGATCTGAACAATTTAAATATATAA
TTTTATTATTTTTATTTATGCACATGTGTGTGTACATATGGGTATGTGCACATAAGTGTAGGTCCCCAAAGGTGTCAGAGGCATCAAATCCCCTGAGA
GCTGTCATTACAGATGGTTATGAGCCACCTGACATGGATACTAGGAACCAAACCCAGGACCTCTGGAAGAGCAACAGGGGCTGTTAATGTACCATAAG
TCCAGCTCTGATCTAAAAAAATTAAACAGAGATCCACAAGCTTAAACTTAAAATAATCATTAAATAATAGCACATAAGACTTGTTAAAACCTTCTGAA
TGGGAAGCAAGATAGGTTCTGTATATGATTTTTCTCAATGAATAACCTCATACCATATCCCATAACCAAAAGCACACCCATTTATAAAAAGCACAAGG
```

AAATTCAACTCCTTTGCTTTTACATGTGATCGTATTTATGACTATAAAAATCTTGTGTTTTCTTCCCTTCCTTTAATTCTCACTGTGAATGTTTCCTT
TTAAAGCAACATAAAGCTAAGATAAACATACACAAAAATTCTGATGTGAGAGAAAAATTACTAAGTATAATAATTCCTCCAGATAACTTTAATTCTTC
ATTCTATCTGAAAATCCTAGTCATCTGAGTAATAAGCCAATATGGCAGCCTCTCCACTTGACATTTCATTCTGCCGTGACCTCCTACTCCAAACGTAC
ACTTAGAGAAGTTTCTCAATAAAGAACTCAAGATCATTCATATTTCTATTTAG

Exon 3 (66 bp) encodes 22 amino acids.
GCATTTCCTCAACAACCTGGGGCTCAAGGCATGGCACCTCCTGGCATGGCTAGTTTGAGCCTTGAG
A F P Q Q P G A Q G M A P **P** G M A S L **S** L E

Intron 3 (1278 bp)
GTATGTATCATCAGAGGCGTGTGTGTGTGTGTGTGTGTGTGTGTGTGATTTCTATACTAGCATGTCCCTGATTGTAGATAAGCATGATGCTTTGTTTC
TCAGTTTCCCTATTCCAGAAATCTTTGACTATCATTCGGAAGAAATCTAAAATTGTTGATGACGTTCACTACAACTTTATCTCATGATTCTACTTCAT
TTTTAAATTCAGAGATATTTTAAGTTTTAGGACATTCATATGTGAATCAAAGCAACTGTAACAATTAATAATAATAATAATAATAATATATATATATA
TATATATATTATATTATATTTTCTGTGTTGAAACCCATTATTCTGTGCTTGGAAACAGCTTACACTTAGTTCTGTTGCTTATATCTTAGATGATAAAG
TCTCATGACATAAACACACTTGGACACACTTTAGCTTTCTAAAACAAAGTGCTATCAGTATCAGTAACATGACTTTATAATTGCTCCTGTACAGGTTG
CTTTTTTTTAACTGCAGATCCCATAAATAATGCATTTTTTGTTTTTGAAAACCATGGTGAAATAGGAAGATTTTATAATGAAGAAGCTAATGGAAACA
CAGATAAATTTCACAGTTTTCACAAGGTCAGGCAAGATTTCATCTGAGTGCAATCAGTACATGTGTGGGCCAGATAGTTATTTCACATAGAGGCTAA
TTTCATTTAGGTGGTTTTTCTCTCAGCTTTTAGCTCAAATTCAATACAAGCAACATTCTTTTCCATTTAAAAAGTAAATCTAGGCCAGGTGTTCACCT
ATAATCCCAACATCCAGGGAGACTGAAGAGAGGAGGAGGATCATGATTTAGAGACCAACATGGACTGTCCGGGGAGACCCATCTCAGAAGGAAGGAAA
GAGGAAAGGAAGGAGGGAAGGGAGACAGGGAGGAAGGGAAGAAGGAAGGAAAAAGAAATTTAAGTGAAGGAAAAAGAACGGGCTGATAAAGCAATAGT
CTTACAACTGTCCGTGTATTGTATTTGAATAGACTGTTTTCCTATGTCTCAGTGTGACCCCAGATTAACTAGTATTTTCCCATTTAATCAGTGACAAA
AGCAAGTGTACTGTTTTCAGACTGAACATGGCAAAGCTACATTTTACATTTCTTTCTTTCCGTAACAATTTGTGTCTACTTTAGCTCTATTCAAGTTG
CCTTACCCCAAGACTTGTATTGCTATTAAATGTCATGAGAACTCAGTTGTGTCAAACGGACATCTAACATATGTTAGAGCATTAACCTACTTTTTCTT
ATAG

Exon 4 (48 bp) encodes 16 amino acids. Green highlight is reverse priming site for Exon 1-4 RT-PCR
ACAATGAGACAGTTGGGAAGCTTGCAGGGACTCAACGCACTTTCTCAG
T M R Q L G S L Q G L N A L S Q

Intron 4 (882 bp)
GTAACTATACGAGATGAATGAGATCTTCATAAACACTTTTTAAAATATTTATTACAACATTGATAGTTTGTAGTTATGACAGCAACAGTACTATAGTT
TCTAGAATTTACTTATTAATAGGTAAATACCTAAACACTGAGAGAAGAAATGAGTGATTAAGAAAGCCTTAAACAGCCAGGCATGGTGCCACACACCT
TTAATCTCATCACTCGGGAGGCAGAGGCAGGCGTATTTCTGAGTTCAAGGCCAGCCTGGTCTACAAAGTGAGTTCCAGGACAGACAGGGCTATACAGA
GAAACCCTGTCTCAAAACAAAAAAAATCCTTAAGCAAAATAGTGTGATGGTCAAACATGAGCTCCACTCCAATAACCCCAATCTTTGGGAGACTGAGA
CAGGAAGAATGGTTTGAGTTCGCAGTCAGCCTGGGCTACACAGTTAATATCAAAAGACACATTTTAGTAATATTAATATGTGCTGGCAAATGAAAGTA
GAAATAATATATCCAGTACTCGGGAGCTGATGCACAGAGAATGCAATTCAAGGCCAAGCTGGGAACATGCTGAAGTATAAGCCTTCCTCAGAACACTA
ACAAAATAAGCCATCAAAATATATACATTGTTACTTTAAGTAAAATAAAATTACTTATTACACTAATAATTTTGTTAAGTTGTCCCAAACAGAAATTT
CTAGAATGATAAATCACCAACAAGTTTATGGTATATTGTGACATGCCCAGAACATAATTTAATTTTGTTGGAAGAGAGATAATTTTGTTGGAAGAGAG
AATTGGCTTACCCATTCCACACAAACCTGAATTACTTTTGCTTTTGGTTGCTATCTCACAATCCTGAAATGTATGTATGCTTTCTTTGTCTCTGTCAG

Exon 5 (111 bp) encodes 37 amino acids. Magenta is AMBN-89 antibody target sequence.
TATTCTAGACTTGGCTTTGGAAAAGCACTTAATAGTTTATGGTTGCACGGACTTCTC
Y S R L G F G K A L N S L W L H G L L

CCACCGCATAACTCTTTCCCATGGATAGGACCAAGGGAACATGAAACCCAGCAG
P P H N S F P W I G P R E H E T Q Q

Intron 5 (1528 bp)
GTAAGCTAATTGCATCCATGTGTTCAAACTCAAGGTTTAAGACTTTTTCCTACTTTTGTTATTACAATAGCAGCAAACACAAATTAGTAATTGACCAA
AAACTCCCCTAATACCAAATAAATCAAGTCCTATAACTCCTGTTACTTAGGGAATATTTTTATTTTAAATTTGTAATTTATTTCAAATAGCCTCTGAA
AGAAGAGACCTACAATAAAGATATCAGGAATAACAATAAACCTCAAAAATAAAAACAGACTCTAGTCTGGATAACTTTCATTTTAATCAAAATTTCCT
AATTACCTAAAGACTAATTTCAAATGACAATAATCATCCTGATTGAAAACACAATGAGCCAAACAGGAACCAATATATTAATCTGTCAACTATTATTA
ATTAGATTCCTAATAAGCATATTATCCAAACTGTGGTCTGGGGGGGAGGGGGGCACGAAAATAAGGACATGTTAGAATGGCTCAAAGGCTCATGGGAT
TTCCAACTGATGGGACATATTTCAAGGTGCTAATAGTGAAGTAGAAGTAATGGATGGCATAGAAGCTGACACATGAGCAATGGCTGAAGATAAAAGGC
AGAGCCAAGTTAATATGAGACTTCCATACCCATTCCAGGAGTATGCAGTTAACATATAGATAATATGCAGCCAATCACAAGTAATTGGTAAAAAACAA
AACCCACAATAGTTTAATATTACTCTTAATTCTACATGGACTTGCATAACAGCATTATCTACAAGGCCACAATGACCATATGAATTGTGTTATTATTC
ACTTCACTTGATTGCTTCCAAAATAGATTTATGATGAAACATATAAGTATTTTAACTATCCTTTTAATAAATCACAAAATATTGTAAGTACCCTTCAT
ATATCAGGTGTTTATTAGTAGCAATAGTAATTTGTGAAATACCAAGGTATAAAATCTGTCAGACAATTCTTTTTTTTTTTTGCTCGTAATTTATTTTT
CTATCCACATTACATCCCAATTGCTGTGCCCCTCCCTACTCTGTTCCCTGTCCCACCCTTACAAATCCCTTCCACAATTACTCCCTCCCCTTCTCAGA
AAAGAGGAAGCCCCCCTTGGCAACCCTGGGATATCTAGTCCAAGCAAGACCAAGCACTTCCTCTCCCATTGACAAATTCATATAAGCCAGTAAGAAAG
TGATGAGATAAGATATATACAGATAGTGGTAATTACCCAGAGGGAATGAGTCACACCAACAAAGAGTGCAGGTGTGGAGATTTCCGCACCCCATGAAA
CAATGGTCCCAACAAAGGTGGAGAGGTAAGTATGTTTAAAACTTTCTCAAATACTTTCTCTGAAGTGTCACCTCTCCTCCTGTGTAAACTCTGCTTTA
AGACTACTCCCTTCCAAACAGCAAGCCTCCAAGTACTTTGCTGTAGAAATTCTAGGCACTGTGGTTCAATGAACCATCTCTTTCTGACATTCTTTTCA
AATTTCTCTACAG

Long Exon 6. This 45 bp/15 amino acid segment distinguises *Ambn* isoform 1 (NM_001303431.1), which includes it during RNA splicing from *Ambn* isoform 2 (NM_009664.2), which deletes this segment through use of an alternative 3' acceptor during RNA splicing. (Magenta is AMBN 89 Ab target sequence).

TATGAATATTCTTTGCCTGTGCATCCCCCACCTCTCCCATCACAG
 Y E Y S L P V H P P P L P S Q

Short Exon 6 (195 bp) encodes 65 amino acids.

CCATCCTTGCAGCCTCACCAGCCAGGACTGAAACCCTTCCTCCAGCCCACTGCTGCAACCGGTGTC
 P S L Q P H Q P G L K P F L Q P T A A T G V

CAGGTCACACCCCAGAAGCCAGGGCCTCAGCCTCCAATGCACCCTGGACAGCTGCCCTTGCAGGAA
 Q V T P Q K P G P Q P P M H P G Q L P L Q E

GGAGAGCTGATAGCACCAGATGAGCCGCAGGTGGCACCATCCGAAAACCCACCAACACCTGAG
 G E L I A P D E P Q V A P S E N P P T P E

Intron 6 (1004 bp)

GTAATTCTTTGGAGCCCACCTAAGTTACTTCTTTAAAATCAGATCAGGATCACCCACTAACACAATGAAACAAAAACCAACTGGTAGGGCATACTTAA
ATAATCACATCTCTCAGACCTAACATGCTAAAAAAATGAACACTTTATTAATATTCTTTCATGAGACATAAATATCTATTTCAATCTTTTGCCATCAG
AGATGTCAGGTTGCAAAAGATCTGCATCTCTAACCTTGGCTTAATGAGACCATTCCTTAGGAATAGGACACCTCAGAGAAGTCCAGGCTCATCAGCTG
GGGGTTTTCAGTGTTGGAAAAAGTATAGTACAGTGATTGTGTCCTCTGCAGATAAAATACAGCTATGCAAAATTACCTTGTAATATTGTAAGGAAGAG
AACAATGGACAGAAAAGGGAAGGTGCTATGTAACTCTCAATACTATGTAGCATGCATTTAATATTCCTTCATATCATCCTGACTACACCCTATAAAGA
AAACATTAGGAACCCTTTTAAGCCCAAGAAGGTGCCAGGCTTGGGACACACCTTTAATCCCAGGACTTGATCACAGGCAAAGAAAAGAGGATCTTTGA
GGTCAAAGTGAGCCTGATCCACAGATCGAATTCCAGGATATCCAGAACTACACAGAGAAACCCTGACTTAAAAAAAAATCAATAAATAAATAAACCTA
AGCTTTGAGAGGCTACAAAGTTTTCCAAGGATTACACTAGTGGCCGAGTAAAAATTGAAACCACTCAAGTATGTGCCAATTCCAAACCTCATTACCAT
TCCCTCTATAATGTAGAACAAACCCAAGATAGACATTCTCATCTCCCTGTCATTCACTGGAGCCATGTCAATATAAGACAACTTTAAAAGAATAGGTT
ATCCCAAGACTTAGAGAATAAGACAGAGAGACATCTTAAACTGCTCTATTATAATCACAAACTAGAGGTGTACTCAGTTTGTCTGTTTTGAGTGACAG
TTTTAATATTTCCTCATGACACAG

Exon 7 (39 bp) encodes 13 amino acids.

GTACCAATAATGGATTTTGCTGATCCACAATTTCCAACC
 V P I M D F A D P Q F P T

Intron 7 (621 bp)

GTAAGTACAGATTTCAATAAGGTTCTATCCTGGAGAAAATATATATATGTGTGTGTCTATATTTCAAAGATTTTGAATGAATTTTCTTCTTTTCTTAT
TTACCTATAGGTATAGAGTATTTCTGAGTTTCTTTTTCTAAATATTAAGTGCTAAGTTTTCACACTAATCAATCAAAAAAATGTGTTCCTGGAACTCT
TAAAAGTCCTTCAGTATGTGCATATCATGTGTGTTCTTTTGTGATTGGGTTACGGAAGTCCACATTCTGTCAAAAGCATCAGGATGATATCCTCCAGA
TGCATCTATTTGCCTAAGAATTTCATGAATTCATTGTTTTTAATTGCCAGAGCTGTGTCTCTAGTTGCATATGTAGCAGAGGATGGCCTGTCAGCCAT
CAATGGGAGGAGAGGCCCTTGGTATTGCAAAGATCATATGCCCCAGTACAGGGGAATACTAGGGCCAGGAAGCGGGAGTGGGGGGTTGGGGAGCAGGG
TGGGGGGAGGGTATAGGGGGCTTTGGGGATAGCATTTGAAATGTAAATGAAGAAAGTATTTAATAATAAAACAGTCTGTCAGTGGAGGTTTGCCTGTT
TTGACTGCCTTTTCTGCTTTCCACCTCTTAAAG

Exon 8 (60 bp) encodes 20 amino acids.

GTGTTCCAGATCGCCCGTTCAATATCTCGGGGACCAATGGCACACAACAAAGCATCCGCT
 V F Q I A R S I S R G P M A H N K A S A

Intron 8 (99 bp)

GTAAGTGTCTGCTTTCTACTTCCACATCTGTGTTTGAAACTACTTAAAAGGTTGATCTTGGGGAGATTCACTTTTTTATAACTATTTT
GTTCTGCTCAG

Exon 9 (45 bp) encodes 15 amino acids.

TTTTACCCAGGAATGTTTTACATGTCTTATGGAGCAAACCAATTG
 F Y P G M F Y M S Y G A N Q L

Intron 9 (371 bp)

GTAAGTCCACATTCTGTCAAAAGCAGCAGTTAAAAATCAATAGCATCTTGAGGGCTAAAAACTGTAGAATCCATACCTAACACAAGAT
ATTCCAAATATATAATCCACAAATACCTACAGTCATGATTTACATAGTAAAGTGGCACACTTTCCTATTTGCATAATTCCAAACAGAC
ACAAACACAATCACATTAAAGAAAAGGTTAATTTTAAATCTGTTAAATTGGCTCTATTTACTCAATAATAGTCAACTCTGTACTCTGC
TCTGGACACAGAGCTATTGAGTCCTAAGAGGATACTGAGTGGCTTTTGATATTGTCTTGAGTAGATGGCTTGAGTTCATACAGTGACG
CACTGTTTCTTTTGAACAG

Exon 10 (45 bp) encodes 15 amino acids.

AATGCTCCTGCCAGAATTGGCTTCATGAGTTCAGAAGAAATGCCT
N A P A R I G F M **S** **S** E E M P

Intron 10 (1701 bp)

GTGAGTACCACCTTCTAACACTTCTTAAAACAATGGCCAGGGAGAACAGCTGCTGTCTACCGGAGGCAATGACTGTAAAAGAACCTAAAGGATTATTA
GACTTCAATTCCTCTATTCTAAAACTGACAGCTCAATTCTGAAGCAGTATCAAAAGATTTAAAACACTTCAGAGCTGAGAATGGTGGTGATGCACAGA
ATCGCAGCCCTCAGGATGTGGAGGCAAGAAGATCAAGAAGAGTTTAAGGCCAGCCTTGACTTCATAAGACCCTGACTCAAAAATAATTGCTTTTTCAT
CACATTTTCTAATCATTTGTTCCTGAGTGGTGGCTATTTTTGAGCCCAGGATTTTTGTTCCACTCCTCGTATATGAACAGAAGAGTCACCCTTCCAAG
AGGAGTCAGTAGAGTGAACCACAAATCCTTCAAAGTGAAAAAGATGTGTGGTTCATACAGATATCTCTTGCCTTTTAGGACTATTTAGCTCCCCCCCC
CCCCTGCCCTCATGTACCTATAGGATTCTAGAACTTAATGTATTTCTTTTGCTGCCATGACCATGGGAGGCATAATCTAGGACTATTTATACTTGAGC
AAAGCTGTGTGATTTTGTCATTGTTTTGACTTATTTTATTTTTTTTTGGGGGGGGGGAAAGGTTTTATTTTGTTTATGCTTCCAGATCACTGTTTATC
AATGAAGGAAAGGAAAGGAAAGGAAAGGAAAGGAAAGGAAAGGAAAGGAAAGGAAAGGAAAGGAAAAAGAAAAGAAAAAGGAAAGAAAAAAGGAAAGG
AAAAAGGAAAGGAAAAAGGAAAGGAAAAGGAAAGGGAAAGGGAAAGGGAAAGGGAAGGGAAGGGAAGGGAAGGGAAGGGAAGGGAAGGGAAGGGAAGG
GAAGGAAGAAGAGAAGGGGGAAAGGAAAATGAGGAAGAGGAGACAGGGGGACATAAAAGATAGATGGAGGGAAAGAGTTAAAACTCATACATAGAGCT
ATATTTGAGGGATACTGAAAACATGTCCCCAGACATTCCTCCTCAACCAAGGCATAACGAAGCTTGTGTTTTGTTTCATGATGATAGAGCCCTTTTCA
ACTGCTTACTGTTTTCTTTTTTTTTTTTTAATTTTTTATTAGGTATTTTCCTCATTTACATTTCCAATGCTATCCCAAAAGTCCCCCATACCCACCCT
CCCACCCCCCTACCCACCCACTCCCCTTTTTGGCCCTGGCGTTCCCCTGTACTGGGGCATATAAAGTTTGCAAGTCCAATGGGCCTCTCTTTCCAGTG
ATGGCCGACTAGGCCATCTTTTGATACATATGCAGCTAGAGTCAAGAGCTCCGGGGTACTGGTTAGTTCATAATGTTGTTCCACCTATCAGGTTACAG
ATCCCTTTAGCTCCTTGGGTAATTTCTCTAGCTCCTCCATTGGGGGCCCTGTGATCCATCCAATAGCTGACTGTGAACATCCACTTCTGTGGCAGAGT
AGTAGTAGAGACCTGCCTTTCCTGAAAGAGGCTTTCTCCTGCCCAGATAAGTGGAGAATCCTCTCCCCTATACTTCAAAGCCAGTTCTATAAGAAGTG
TGTTTCGACAAAGCAGTCAGCCAACTCGCTGTTGTGTTAGTATTTATAGCTTCTCAATGAATATAATCATGTACAATTGTATGGAATGAATAACACAC
AGTAATTGTGTCACTGATGGACGTTTGTTTTCCAG

Exon 11 (1074 bp) Gray highlighting shows the reverse Exon 11 primer annealing site for RT-PCR analyses. **TGA** is the translation termination codon. **AATAAA** sequences are transcription termination signals.

GGAGAAAGAGGAAGTCCCATGGCCTATGGAACTCTGTTCCCAAGATTTGGAGGCTTCAGGCAAACCCTTAGGAGACTG
G E R G S P M A Y G T L F P R F G G F R Q T L R R L

AATCAGAATTCACCCAAGGGAGGAGACTTTACTGTGGAAGTAGATTCCCCAGTATCTGTTACCAAAGGCCCTGAAAAA
N Q N S P K G G D F T V E V D S P V S V T K G P E K

GGAGAAGGTCCAGAAGGCTCTCCACTGCAAGAGGCCAACCCAGGCAAACGGGAAAACCCCGCTCTCCTTTCACAAATG
G E G P E G S P L Q E A N P G K R E N P A L L S Q M

GCACCTGGGGCCCATGCAGGACTTCTTGCTTTCCCCAATGACCACATCCCCAGTATGGCAAGGGGTCCTGCAGGGCAA
A P G A H A G L L A F P N D H I P S M A R G P A G Q

AGACTCCTTGGAGTCACCCCTGCAGCTGCAGACCCACTGATCACCCCTGAATTAGCAGAAGTTTATGAAACCTATGGT
R L L G V T P A A A D P L I T P E L A E V Y E T Y G

GCTGATGTTACCACACCCCTGGGTGATGGAGAAGCAACCATGGATATCACCATGTCCCCAGACACTCAGCAGCCACTG
A D V T T P L G D G E A T M D I T M S P D T Q Q P L

CTACCTGGAAACAAAGTGCACCAGCCCCAGGTGCACAACGCATGGCGTTTCCAAGAGCCC**TGA**TAACCTTGACATAGC
L P G N K V H Q P Q V H N A W R F Q E P *

AGCTACTTTGTGTATGCACAAGCTTTCCAGCTTTGTCCCCATAGTACCTTGTTGCTAAAACACTTACTATCCTTCCGCAGTGAAGGTGTTAAGAGCAC
TAAGCATATATTAATAAATACAAGTGGCTAGAAATAGTGTAGGTCCCTTCTTGCTTCCATTCTTATCAA**AATAAA**ATGTATCAACTGTCTCCGTGACT
TAGAAATACTATCAATGATATCAGAGCAAGTCTAAGTGTCAGCACTTGGTGGTATAGCATGTAGCTGTCTTAGGCATTATAAAATTTCTCTTACTACA
TGACATTATTATGCCCAGGAAATGTGACACTGCTTCTTTCTCTACACAAAAGCACTTAGTTTCAGAATTCCAAAGTATTTCATTTAAACCTTATTAAA
TGGTGATTGGTGGAAAACCCTGACTGCTATTACTGGTTTCATATATTGGATTTAAAATTCTTATTTATAGAATATTTTATTTAATCTAGGAAAAGAAA
GGGCAATTGGCCTGTTTTA**AATAAA**GAATTTTTCTCAC

Figure S4. Sequence of the *Ambn*^{lacZ} allele following deletion of the PGK driven neo selection cassette by FLP-FRT recombination

Exon 1 (116 bp) underlined ATG is a weak, in frame translation initiation site. Gray highlighting shows forward Exon 1 primer for RT-PCR analyses.

GAGAGATACAGAGAGAGCCCCAGGAACAGTCCAGAAAAAAAATTAATCTTCTTTTCTTA
GAACTGTTTTGATTGGCATCATCAGGCCTGGGAGCACAGTGAATGTCAGCATCTAAG
X m s a s k

Intron 1 (778 bp)

GTAAAATGTGATTTTATCATTTCCATGTGTTTGCTATAAATTCTCTTTTCCTGTCTCCAGATAACCTTTACAAAAAAGATTTAT
CTTCATTTATCACAAGATGCTGAATAACAGCTTATTTCAACCATTAGAATATTCCAGAAAATATATTACCATTAACCTAAGAAA
ATAAACTTATTTGTATGACTTTTTAAAACTATGTTTGTGATTATTAGTGATAGTGCTAATTTATAAAAGTAAAGAGGATAAGCA
CACCTGCTGATCAGATCTGCTTCCATTCGAGAGTGAACTGACAAAATGCATCTCAAGATTATTCATTTTTAGGAAGGAAAATCA
CTATCCAAGCAGATAAATATACTCAACTTTAGCTTGAAGGCTGAGTTTTGACATGGTTCTGCATTATGACCCAATCATAGGTTG
TATTTCAAATTCTGAGTTTAAACAAAGAATACAATTCTGTAAATGGTGACTGAAGGATGCATTTTATGAAGCAAAGCATAGTGT
TCAAGGAATATTCCTGGAGGTTGTTCTTAAACATTCATTCAAATTGCAGCTTCATAAAAATAAGTACTCAGATCCATAGTTTGG
CTGCTCGTCATTCCATTCTGGTACCACTAGTGCGGCCGCGATAGCCACTGGTTTAGCAGCTTTTTGCTTTTGCATTTTAGCACT
TTTGCATTTTTGAGTTTTTTAACCTGATTGTCTTTGGAAGAACAGCCACACGCTCTTAGAAATATACTTTGACCACTATGAATG
CAATTTTGCCTGAGGTGATTTTACAACCCAACTGTGTTTTAG

Exon 2. The 3419 bp knocked in sequence is highlighted in cyan and begins at the strong *Ambn* translation initiation site (**ATG**). The annealing sites for the *Ambn-LacZ*R (double underlined) RT-PCR primer is bold. The translation termination codon (**TAA**) and the two polyadenylation signals (**AATAAA**) in the 3' untranslated region are shown in bold.

ATTCCACTTTTCAAA**ATG**GCTCCCAAGAAGAAGAGGAAGGTGATGGAAGATCCCGTCGTTTTACAACGTCGTGACTGGGAA
i p l f k M A P K K K R K V M E D P V V L Q R R D W E

AACCCTGGCGTTACCCAACTTAATCGCCTTGCAGCACATCCCCCTTTCGCCAGCTGGCGTAATAGCGAAGAGGCCCGCACC
N P G V T Q L N R L A A H P P F A S W R N S E E A R T

GATCGCCCTTCCCAACAGTTGCGCAGCCTGAATGGCGAATGGCGCTTTGCCTGGTTTCCGGCACCAGAAGCGGTGCCGGAA
D R P S Q Q L R S L N G E W R F A W F P A P E A V P E

AGCTGGCTGGAGTGCGATCTTCCTGAGGCC**GATACTGTCGTCGTCCCCTC**AAACTGGCAGATGCACGGTTACGATGCGCCC
S W L E C D L P E A D T V V V P S N W Q M H G Y D A P

ATCTACACCAACGTAACCTATCCCATTACGGTCAATCCGCCGTTTGTTCCCACGGAGAATCCGACGGGTTGTTACTCGCTC
I Y T N V T Y P I T V N P P F V P T E N P T G C Y S L

ACATTTAATGTTGATGAAAGCTGGCTACAGGAAGGCCAGACGCGAATTATTTTTGATGGCGTTAACTCGGCGTTTCATCTG
T F N V D E S W L Q E G Q T R I I F D G V N S A F H L

TGGTGCAACGGGCGCTGGGTCGGTTACGGCCAGGACAGTCGTTTGCCGTCTGAATTTGACCTGAGCGCATTTTTACGCGCC
W C N G R W V G Y G Q D S R L P S E F D L S A F L R A

GGAGAAAACCGCCTCGCGGTGATGGTGCTGCGCTGGAGTGACGGCAGTTATCTGGAAGATCAGGATATGTGGCGGATGAGC
G E N R L A V M V L R W S D G S Y L E D Q D M W R M S

GGCATTTTCCGTGACGTCTCGTTGCTGCATAAACCGACTACACAAATCAGCGATTTCCATGTTGCCACTCGCTTTAATGAT
G I F R D V S L L H K P T T Q I S D F H V A T R F N D

GATTTCAGCCGCGCTGTACTGGAGGCTGAAGTTCAGATGTGCGGCGAGTTGCGTGACTACCTACGGGTAACAGTTTCTTTA
D F S R A V L E A E V Q M C G E L R D Y L R V T V S L

TGGCAGGGTGAAACGCAGGTCGCCAGCGGCACCGCGCCTTTCGGCGGTGAAATTATCGATGAGCGTGGTGGTTATGCCGAT
W Q G E T Q V A S G T A P F G G E I I D E R G G Y A D

CGCGTCACACTACGTCTGAACGTCGAAAACCCGAAACTGTGGAGCGCCGAAATCCCGAATCTCTATCGTGCGGTGGTTGAA
R V T L R L N V E N P K L W S A E I P N L Y R A V V E

CTGCACACCGCCGACGGCACGCTGATTGAAGCAGAAGCCTGCGATGTCGGTTTCCGCGAGGTGCGGATTGAAAATGGTCTG
L H T A D G T L I E A E A C D V G F R E V R I E N G L

CTGCTGCTGAACGGCAAGCCGTTGCTGATTCGAGGCGTTAACCGTCACGAGCATCATCCTCTGCATGGTCAGGTCATGGAT
L L L N G K P L L I R G V N R H E H H P L H G Q V M D

GAGCAGACGATGGTGCAGGATATCCTGCTGATGAAGCAGAACAACTTTAACGCCGTGCGCTGTTCGCATTATCCGAACCAT

E Q T M V Q D I L L M K Q N N F N A V R C S H Y P N H

CCGCTGTGGTACACGCTGTGCGACCGCTACGGCCTGTATGTGGTGGATGAAGCCAATATTGAAACCCACGGCATGGTGCCA

P L W Y T L C D R Y G L Y V V D E A N I E T H G M V P

ATGAATCGTCTGACCGATGATCCGCGCTGGCTACCGGCGATGAGCGAACGCGTAACGCGAATGGTGCAGCGCGATCGTAAT

M N R L T D D P R W L P A M S E R V T R M V Q R D R N

CACCCGAGTGTGATCATCTGGTCGCTGGGGAATGAATCAGGCCACGGCGCTAATCACGACGCGCTGTATCGCTGGATCAAA

H P S V I I W S L G N E S G H G A N H D A L Y R W I K

TCTGTCGATCCTTCCCGCCCGGTGCAGTATGAAGGCGGCGGAGCCGACACCACGGCCACCGATATTATTTGCCCGATGTAC

S V D P S R P V Q Y E G G G A D T T A T D I I C P M Y

GCGCGCGTGGATGAAGACCAGCCCTTCCCGGCTGTGCCGAAATGGTCCATCAAAAAATGGCTTTCGCTACCTGGAGAGACG

A R V D E D Q P F P A V P K W S I K K W L S L P G E T

CGCCCGCTGATCCTTTGCGAATACGCCCACGCGATGGGTAACAGTCTTGGCGGTTTCGCTAAATACTGGCAGGCGTTTCGT

R P L I L C E Y A H A M G N S L G G F A K Y W Q A F R

CAGTATCCCCGTTTACAGGGCGGCTTCGTCTGGGACTGGGTGGATCAGTCGCTGATTAAATATGATGAAAACGGCAACCCG

Q Y P R L Q G G F V W D W V D Q S L I K Y D E N G N P

TGGTCGGCTTACGGCGGTGATTTTGGCGATACGCCGAACGATCGCCAGTTCTGTATGAACGGTCTGGTCTTTGCCGACCGC

W S A Y G G D F G D T P N D R Q F C M N G L V F A D R

ACGCCGCATCCAGCGCTGACGGAAGCAAAACACCAGCAGCAGTTTTTCCAGTTCCGTTTATCCGGGCAAACCATCGAAGTG

T P H P A L T E A K H Q Q Q F F Q F R L S G Q T I E V

ACCAGCGAATACCTGTTCCGTCATAGCGATAACGAGCTCCTGCACTGGATGGTGGCGCTGGATGGTAAGCCGCTGGCAAGC

T S E Y L F R H S D N E L L H W M V A L D G K P L A S

GGTGAAGTGCCTCTGGATGTCGCTCCACAAGGTAAACAGTTGATTGAACTGCCTGAACTACCGCAGCCGGAGAGCGCCGGG

G E V P L D V A P Q G K Q L I E L P E L P Q P E S A G

CAACTCTGGCTCACAGTACGCGTAGTGCAACCGAACGCGACCGCATGGTCAGAAGCCGGGCACATCAGCGCCTGGCAGCAG

Q L W L T V R V V Q P N A T A W S E A G H I S A W Q Q

TGGCGTCTGGCGGAAAACCTCAGTGTGACGCTCCCCGCCGCGTCCCACGCCATCCCGCATCTGACCACCAGCGAAATGGAT

W R L A E N L S V T L P A A S H A I P H L T T S E M D

TTTTGCATCGAGCTGGGTAATAAGCGTTGGCAATTTAACCGCCAGTCAGGCTTTCTTTCACAGATGTGGATTGGCGATAAA

F C I E L G N K R W Q F N R Q S G F L S Q M W I G D K

AAACAACTGCTGACGCCGCTGCGCGATCAGTTCACCCGTGCACCGCTGGATAACGACATTGGCGTAAGTGAAGCGACCCGC

K Q L L T P L R D Q F T R A P L D N D I G V S E A T R

ATTGACCCTAACGCCTGGGTCGAACGCTGGAAGGCGGCGGGCCATTACCAGGCCGAAGCAGCGTTGTTGCAGTGCACGGCA

I D P N A W V E R W K A A G H Y Q A E A A L L Q C T A

GATACACTTGCTGATGCGGTGCTGATTACGACCGCTCACGCGTGGCAGCATCAGGGGAAAACCTTATTTATCAGCCGGAAA

D T L A D A V L I T T A H A W Q H Q G K T L F I S R K

ACCTACCGGATTGATGGTAGTGGTCAAATGGCGATTACCGTTGATGTTGAAGTGGCGAGCGATACACCGCATCCGGCGCGG

T Y R I D G S G Q M A I T V D V E V A S D T P H P A R

ATTGGCCTGAACTGCCAGCTGGCGCAGGTAGCAGAGCGGGTAAACTGGCTCGGATTAGGGCCGCAAGAAAACTATCCCGAC

I G L N C Q L A Q V A E R V N W L G L G P Q E N Y P D

CGCCTTACTGCCGCCTGTTTTGACCGCTGGGATCTGCCATTGTCAGACATGTATACCCCGTACGTCTTCCCGAGCGAAAAC

R L T A A C F D R W D L P L S D M Y T P Y V F P S E N

GGTCTGCGCTGCGGGACGCGCGAATTGAATTATGGCCCACACCAGTGGCGCGGCGACTTCCAGTTCAACATCAGCCGCTAC

G L R C G T R E L N Y G P H Q W R G D F Q F N I S R Y

AGTCAACAGCAACTGATGGAAACCAGCCATCGCCATCTGCTGCACGCGGAAGAAGGCACATGGCTGAATATCGACGGTTTC

S Q Q Q L M E T S H R H L L H A E E G T W L N I D G F

CATATGGGGATTGGTGGCGACGACTCCTGGAGCCCGTCAGTATCGGCGGAATTCCAGCTGAGCGCCGGTCGCTACCATTAC

H M G I G G D D S W S P S V S A E F Q L S A G R Y H Y

CAGTTGGTCTGGTGTCAAAAA**TAA**TAATAAGCTAGCTGATCATAATCAGCCATACCACATTTGTAGAGGTTTTACTTGCTT

Q L V W C Q K *

TAAAAAACCTCCCACACCTCCCCCTGAACCTGAAACATAAAATGAATGCAATTGTTGTTGTTAACTTGTTTATTGCAGCTTATA
ATGGTTACA**AATAAA**GCAATAGCATCACAAATTTCACA**AATAAA**GCATTTTTTTCACTGCATTCTAGTTGTGGTTTGTCCAAAC
TCATCAATGTATCTTAATTAAGAAGTTCCTATACTTACTAGAGAATAGGAACTTCGGAATAGGAACTTCTTTAATTAAGCTAGC
TGCGGCCGCGGCGCGCCGGATCC

3’ 529 bp from intron 4 (originally 882 bp) follows the lacZ knockin gene.

AGCTCCACTCCAATAACCCCAATCTTTGGGAGACTGAGACAGGAAGAATGGTTTGAGTTCGCAGTCAGCCTGGGCTACACAGTTAATATCAAAAGACA
CATTTTAGTAATATTAATATGTGCTGGCAAATGAAAGTAGAAATAATATATCCAGTACTCGGGAGCTGATGCACAGAGAATGCAATTCAAGGCCAAGC
TGGGAACATGCTGAAGTATAAGCCTTCCTCAGAACACTAACAAAATAAGCCATCAAAATATATACATTGTTACTTTAAGTAA**AATAAA**ATTACTTATT
ACACTAATAATTTTGTTAAGTTGTCCCAAACAGAAATTTCTAGAATGATAAATCACCAACAAGTTTATGGTATATTGTGACATGCCCAGAACATAATT
TAATTTTGTTGGAAGAGAGATAATTTTGTTGGAAGAGAGAATTGGCTTACCCATTCCACACAAACCTGAATTACTTTTGCTTTTGGTTGCTATCTCAC
AATCCTGAAATGTATGTATGCTTTCTTTGTCTCTGTCAG

Exon 5 (111 bp; encodes 37 amino acids)

TATTCTAGACTTGGCTTTGGAAAAGCACTTAATAGTTTATGGTTGCACGGACTTCTC
Y S R L G F G K A L N S L W L H G L L

CCACCGCATAACTCTTTCCCATGGATAGGACCAAGGGAACATGAAACCCAGCAG
P P H N S F P W I G P R E H E T Q Q

Intron 5 (1528 bp)

GTAAGCTAATTGCATCCATGTGTTCAAACTCAAGGTTTAAGACTTTTTCCTACTTTTGTTATTACAATAGCAGCAAACACAAATTAGTAATTGACCAA
AAACTCCCCTAATACCAAATAAATCAAGTCCTATAACTCCTGTTACTTAGGGAATATTTTTATTTTAAATTTGTAATTTATTTCAAATAGCCTCTGAA
AGAAGAGACCTACAATAAAGATATCAGGAATAACAATAAACCTCAAAAATAAAAACAGACTCTAGTCTGGATAACTTTCATTTTAATCAAAATTTCCT
AATTACCTAAAGACTAATTTCAAATGACAATAATCATCCTGATTGAAAACACAATGAGCCAAACAGGAACCAATATATTAATCTGTCAACTATTATTA
ATTAGATTCCTAATAAGCATATTATCCAAACTGTGGTCTGGGGGGGAGGGGGGCACGAAAATAAGGACATGTTAGAATGGCTCAAAGGCTCATGGGAT
TTCCAACTGATGGGACATATTTCAAGGTGCTAATAGTGAAGTAGAAGTAATGGATGGCATAGAAGCTGACACATGAGCAATGGCTGAAGATAAAAGGC
AGAGCCAAGTTAATATGAGACTTCCATACCCATTCCAGGAGTATGCAGTTAACATATAGATAATATGCAGCCAATCACAAGTAATTGGTAAAAAACAA
AACCCACAATAGTTTAATATTACTCTTAATTCTACATGGACTTGCATAACAGCATTATCTACAAGGCCACAATGACCATATGAATTGTGTTATTATTC
ACTTCACTTGATTGCTTCCAAAATAGATTTATGATGAAACATATAAGTATTTTAACTATCCTTTTAATAAATCACAAAATATTGTAAGTACCCTTCAT
ATATCAGGTGTTTATTAGTAGCAATAGTAATTTGTGAAATACCAAGGTATAAAATCTGTCAGACAATTCTTTTTTTTTTTTGCTCGTAATTTATTTTT
CTATCCACATTACATCCCAATTGCTGTGCCCCTCCCTACTCTGTTCCCTGTCCCACCCTTACAAATCCCTTCCACAATTACTCCCTCCCCTTCTCAGA
AAAGAGGAAGCCCCCCTTGGCAACCCTGGGATATCTAGTCCAAGCAAGACCAAGCACTTCCTCTCCCATTGACAAATTCATATAAGCCAGTAAGAAAG
TGATGAGATAAGATATATACAGATAGTGGTAATTACCCAGAGGGAATGAGTCACACCAACAAAGAGTGCAGGTGTGGAGATTTCCGCACCCCATGAAA
CAATGGTCCCAACAAAGGTGGAGAGGTAAGTATGTTTAAAACTTTCTCAAATACTTTCTCTGAAGTGTCACCTCTCCTCCTGTGTAAACTCTGCTTTA
AGACTACTCCCTTCCAAACAGCAAGCCTCCAAGTACTTTGCTGTAGAAATTCTAGGCACTGTGGTTCAATGAACCATCTCTTTCTGACATTCTTTTCA
AATTTCTCTACAG

This is followed by the rest of the *Ambn* gene (Exon 6 to Exon 11), as detailed in the *Ambn* reference sequence: NC_000071.6 Reference GRCm38.p4 C57BL/6J.

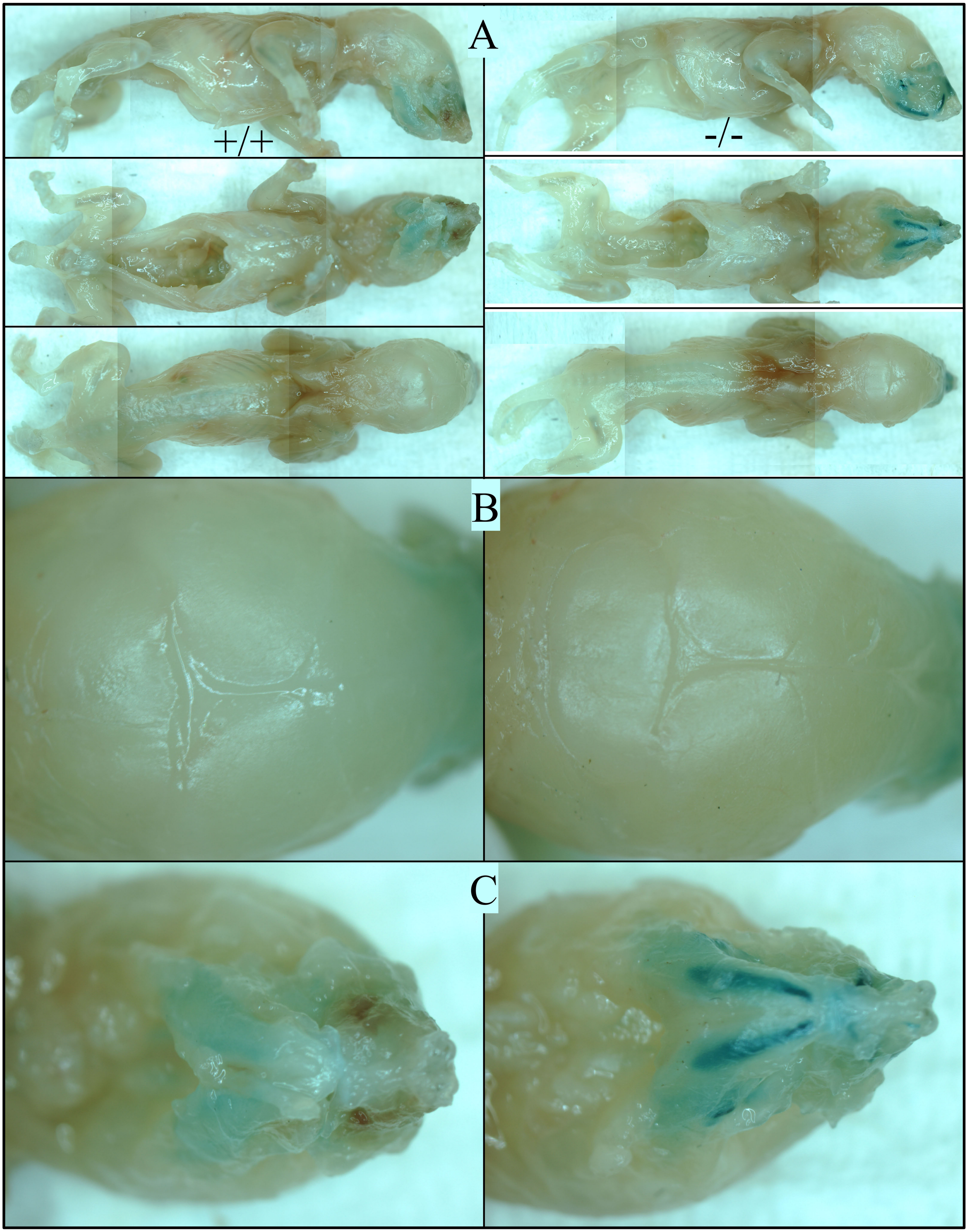

Figure S5. Day 2 whole mount X-gal staining. Skin and abdominal organs were removed. Staining was for 5 h. Wild-type mice (+/+) showed mild endogenous staining in vertebrate, the ends of arm bones, in the mandible, eye and nasal regions. The *Ambn*$^{lacZ/lacZ}$ knockin (-/-) mouse showed very strong staining in the incisor and molar regions. ***A:*** whole body; ***B:*** calvaria; ***C:*** Mandible.

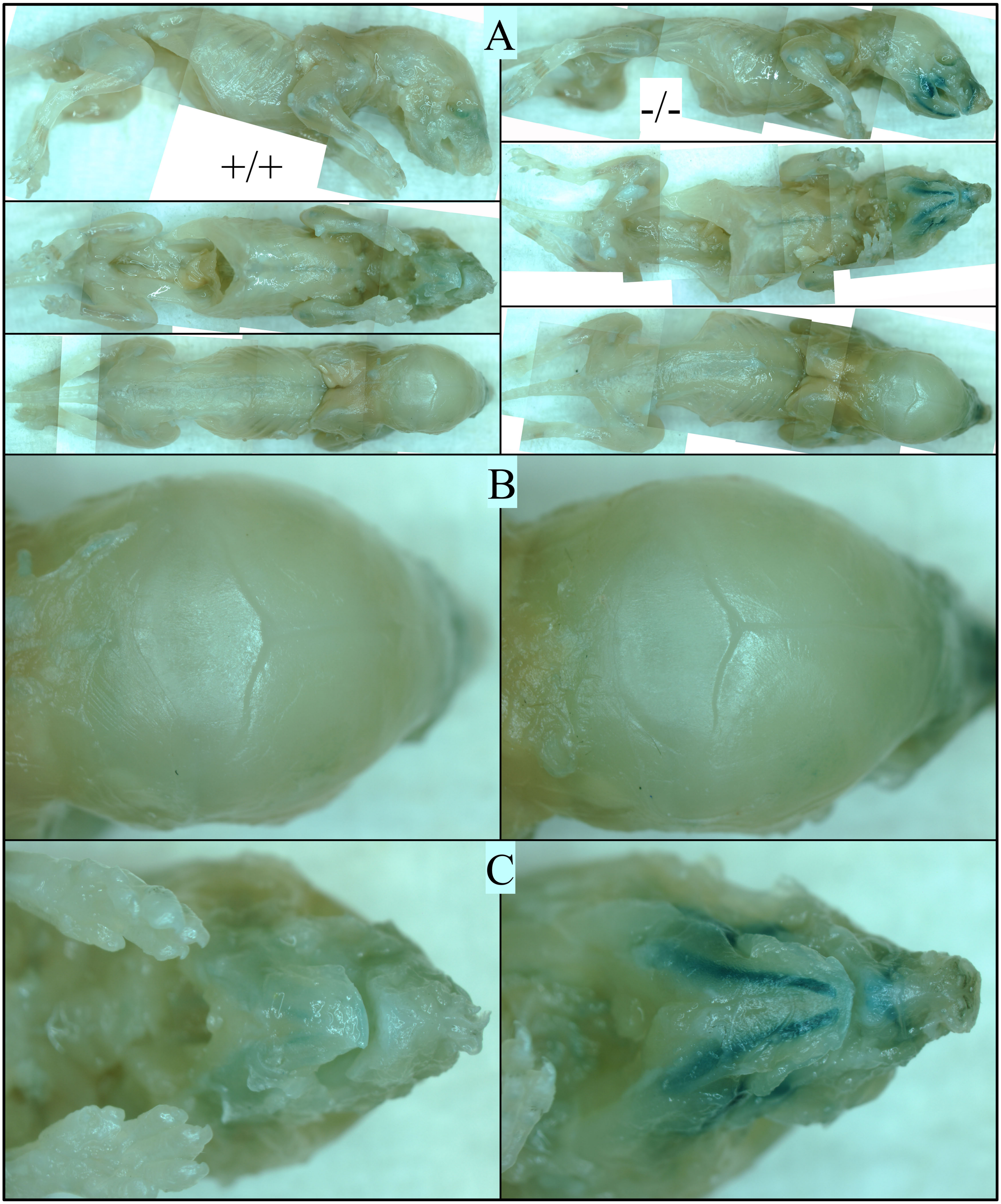

Figure S6. Day 6 whole mount X-gal staining. Skin and abdominal organs were removed. Staining was for 5 h. Wild-type mice (+/+) showed mild endogenous staining in vertebrate, the ends of arm bones, in the mandible, eye and nasal regions. The *Ambn*$^{lacZ/lacZ}$ knockin (-/-) mouse showed very strong staining in the incisor and molar regions. ***A:*** whole body; ***B:*** calvaria; ***C:*** Mandible.

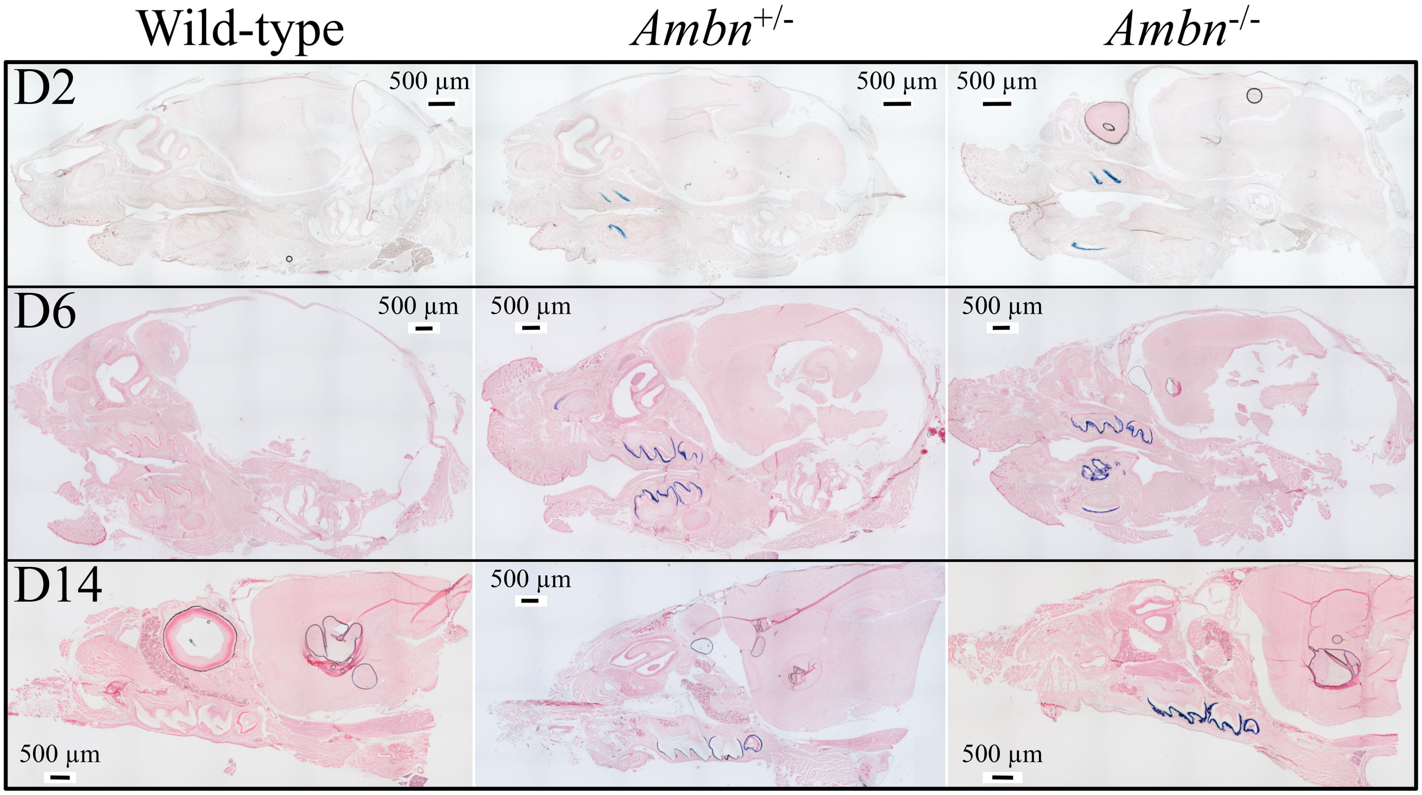

Figure S7. Mouse head sagittal section X-gal staining. Slides were stained in X-gal solution for 1 h at 45 ˚C and then counterstained with Nuclear Fast Red. Wild-type mice (+/+) showed no staining. The *Ambn* heterozygous (+/-) and *Ambn*$^{lacZ/lacZ}$ (-/-) mice showed strong staining in the ameloblast layer in the incisor and molar regions.

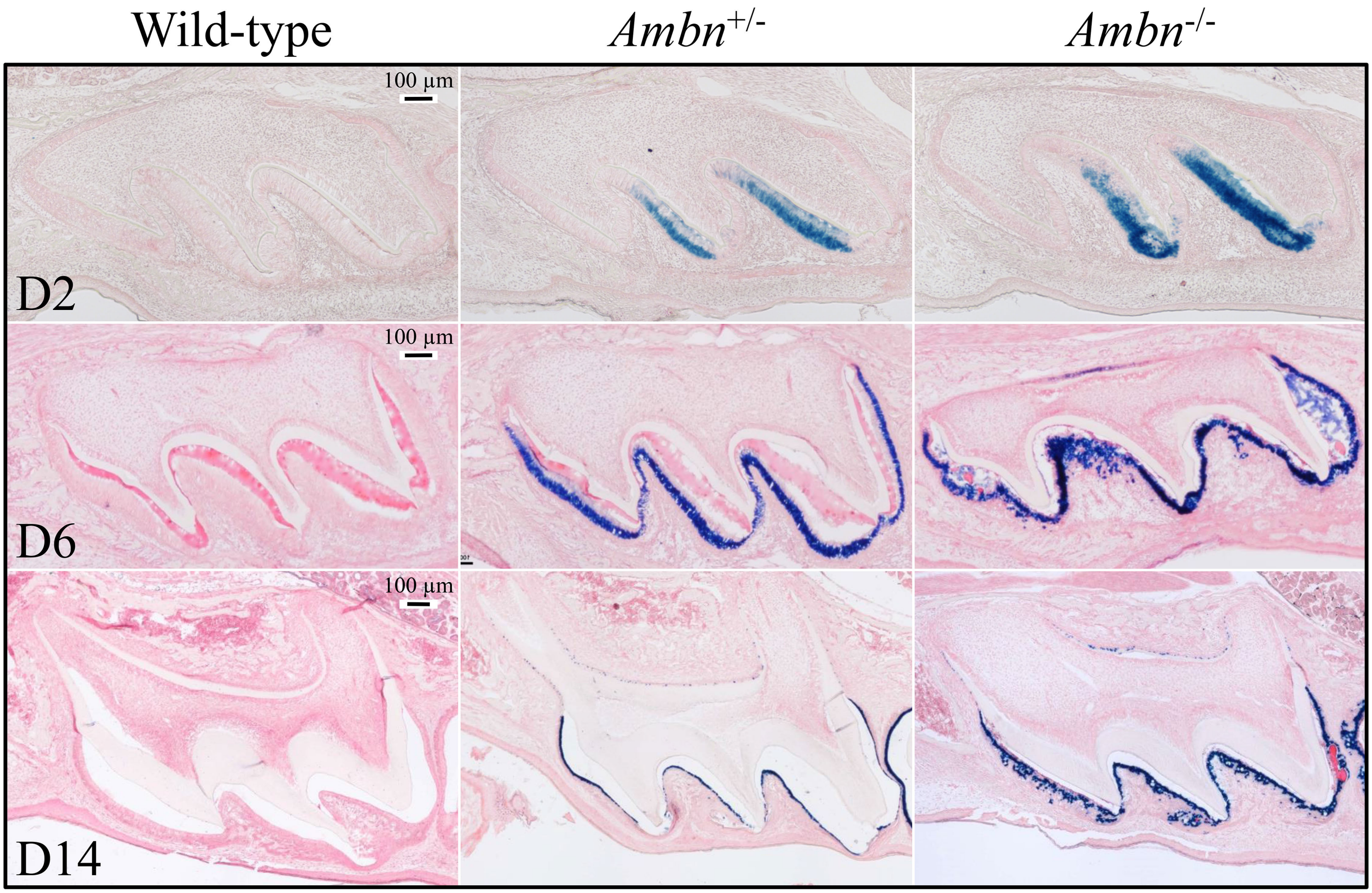

Figure S8. Maxillary first molar X-gal staining. Slides were stained in X-gal solution for 1 h at 45 ˚C and then counterstained with Nuclear Fast Red. Wild-type mice showed no staining. The *Ambn*$^{+/lacZ}$ heterozygous (+/-) and *Ambn*$^{lacZ/lacZ}$ homozygous knockin mice showed strong staining in the ameloblasts, and spotty staining along the outer root surface at postnatal day 14.

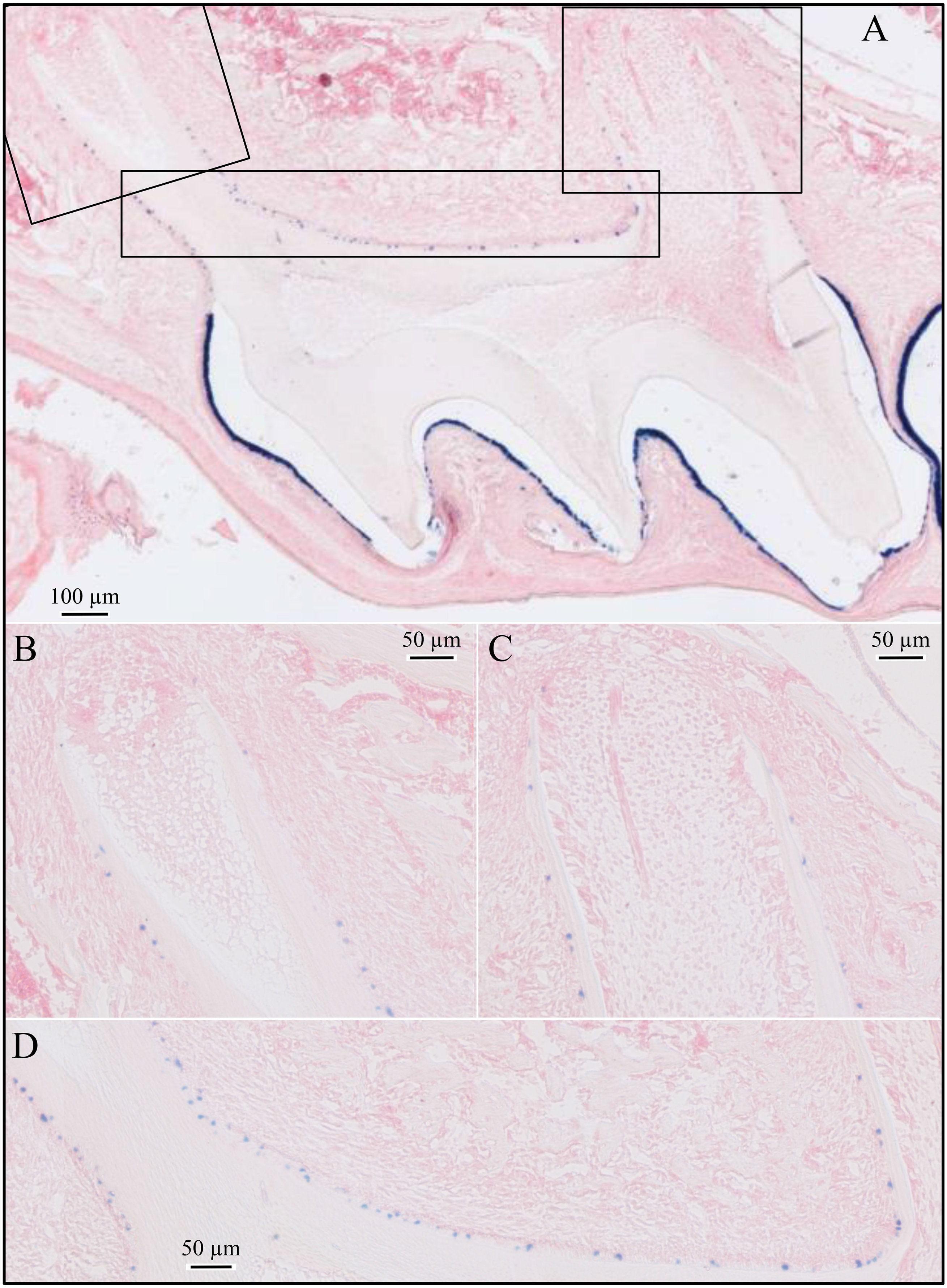

Figure. S9. Day 14 maxillary first molar X-gal staining in *Ambn*$^{+/lacZ}$ heterozygous mice. ***A:*** Maturation stage ameloblasts are strongly positive, odontoblasts are negative. ***B-D:*** Higher magnification of the boxed areas in A show relatively weak, spotty AMBN signal along the roots.

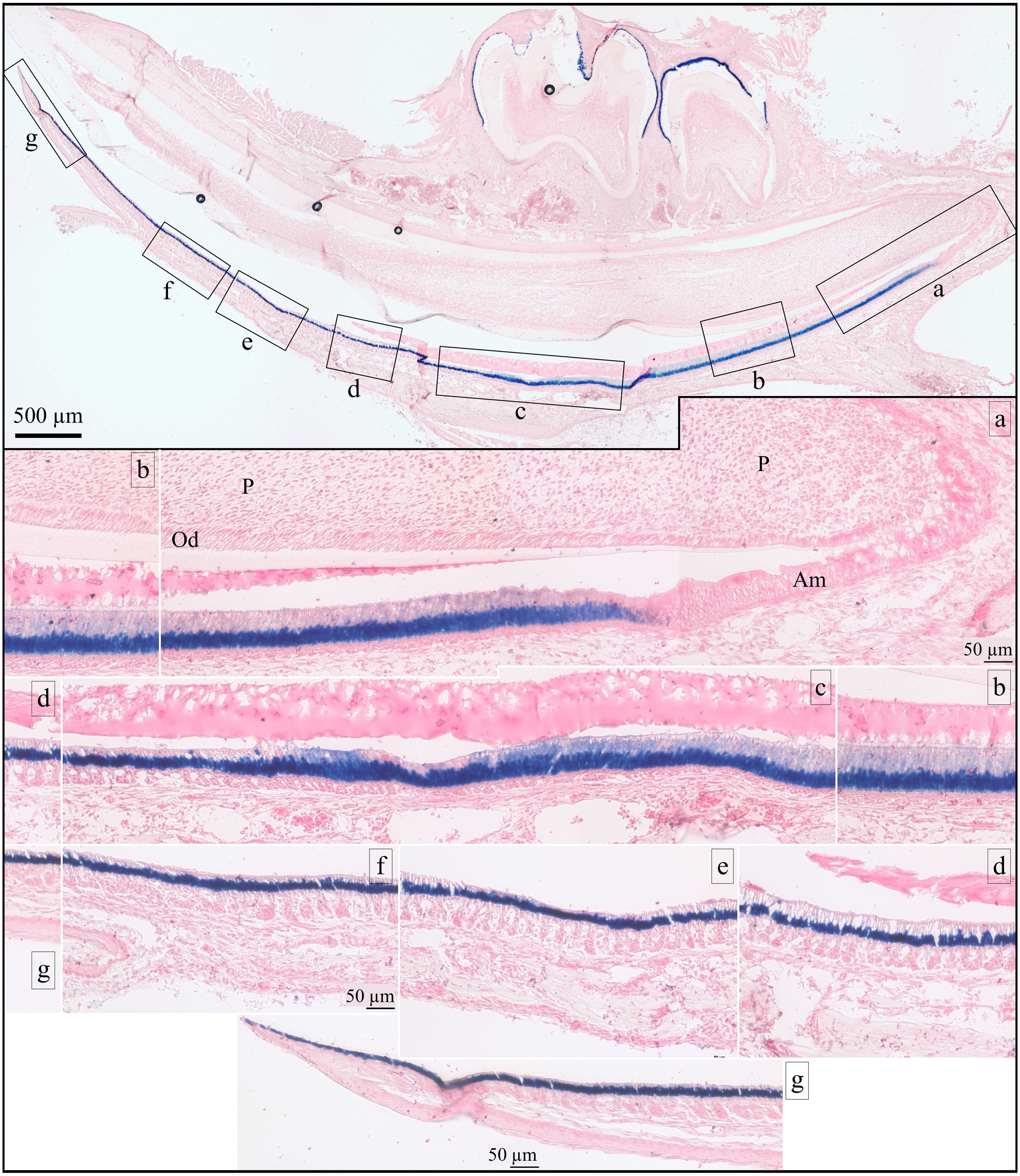

Figure S10. Day 14 mandibular incisor X-gal staining in *Ambn*$^{+/lacZ}$ (heterozygous) mouse. ***Top:*** Low magnification image of D14 *Ambn*$^{+/lacZ}$ mouse mandibular incisor. Boxes indicate positions of high magnification images shown below.

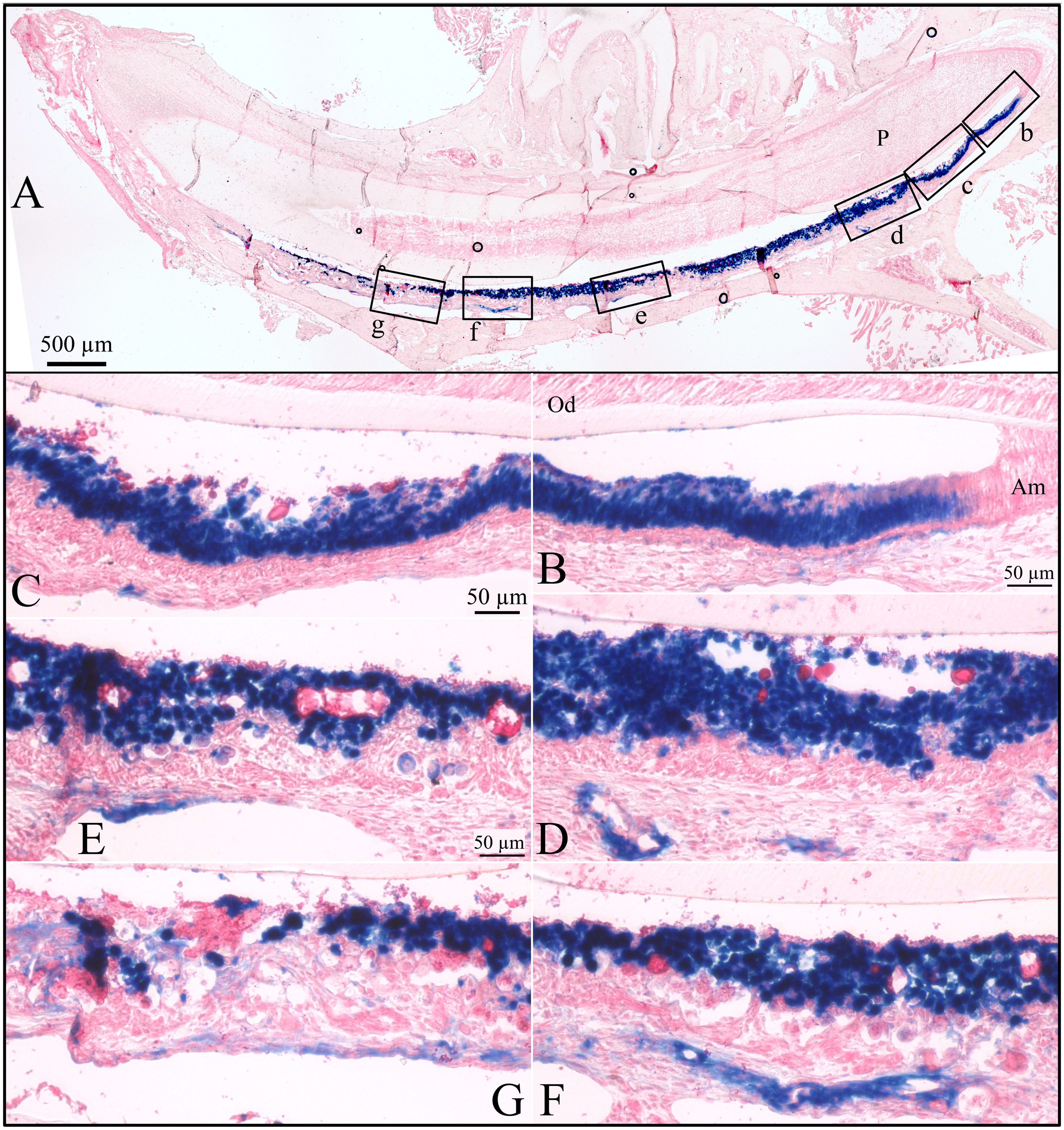

Figure S11. Mandibular incisor X-gal staining in *Ambn*$^{lacZ/lacZ}$ mouse. ***A:*** Low magnification image of 7-week *Ambn*$^{-/-}$ mouse mandibular incisor. Boxes indicate positions of high magnification images shown below. Note the strong staining in ameloblasts and the obvious ameloblast pathology.

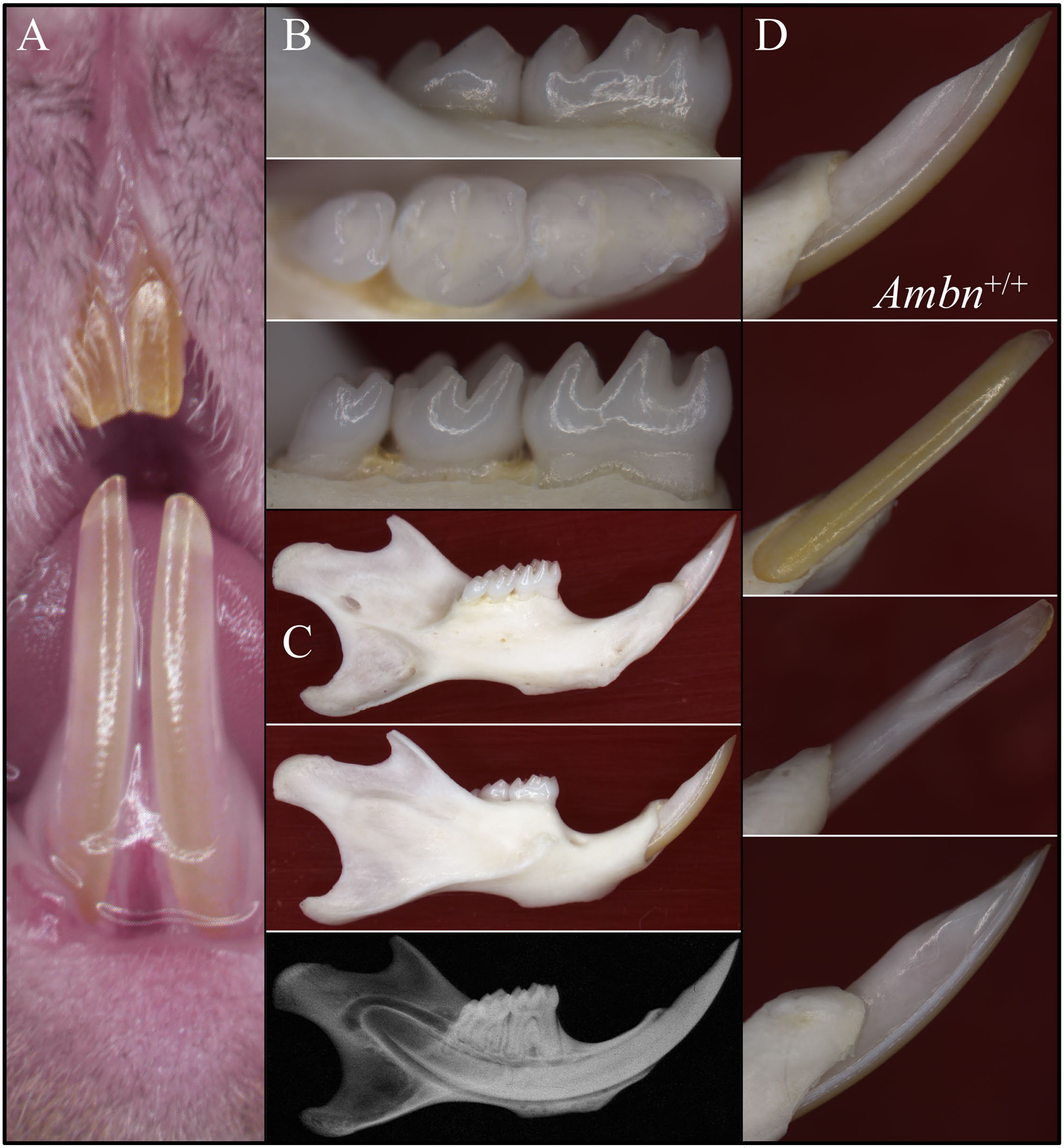

Figure S12. Dissecting microscope photographs of 7-week-old *Ambn*$^{+/+}$ mice. These images are for comparison to those of *Ambn*$^{+/lacZ}$ and *Ambn*$^{lacZ/lacZ}$ mice shown in Fig. 5. ***A:*** Frontal view of maxillary and mandibular incisors. ***B:*** Buccal (upper), occlusal (middle), and lingual (lower) views of mandibular molars. ***C:*** Lateral (top), medial (middle) views, and radiograph (bottom) of hemi-mandibles. ***D:*** Lingual (top), labial, lingual, and buccal (bottom) views of mandibular incisors.

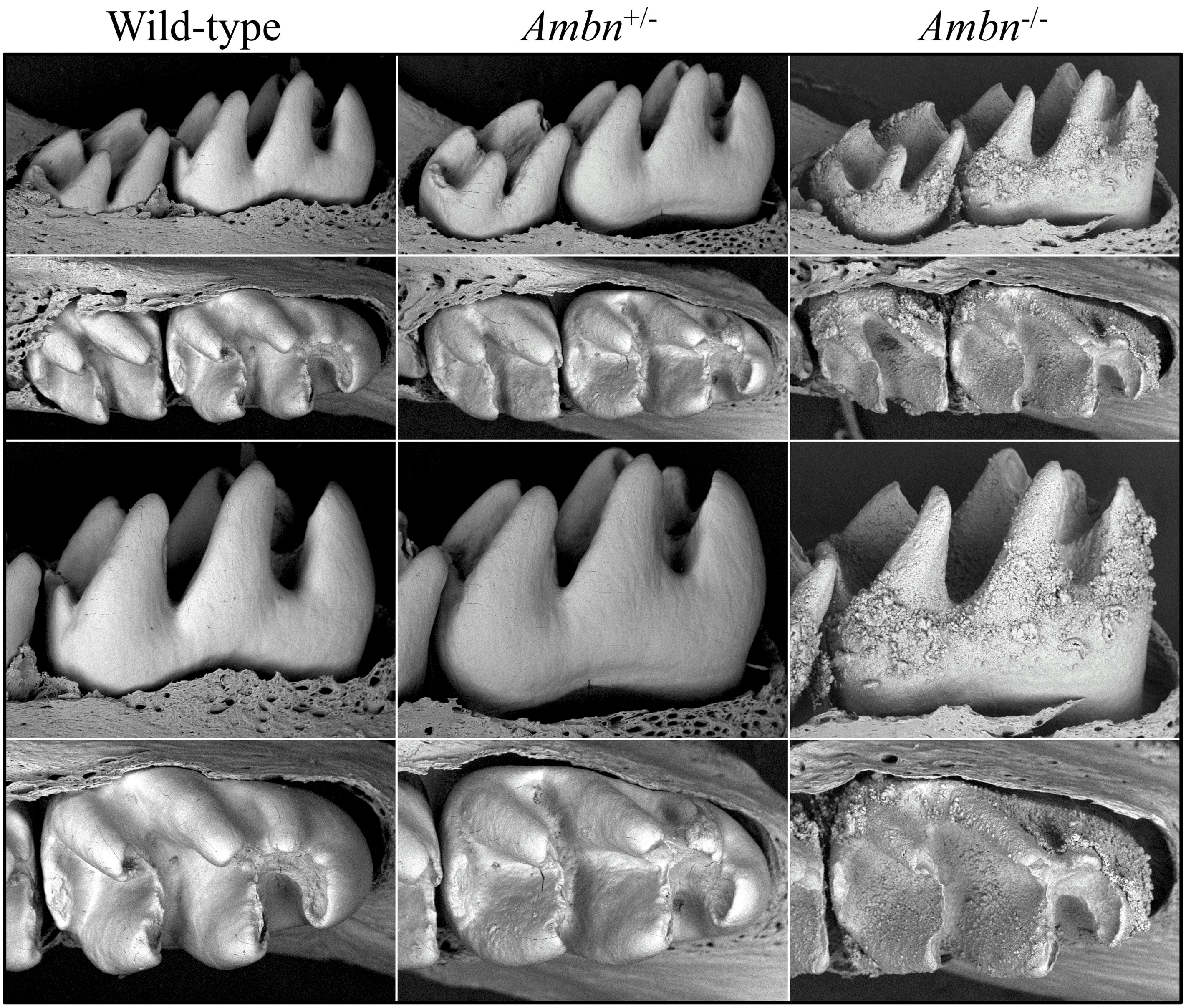

Figure S13. Backscattered SEMs of Day 14 mandibular molars of *Ambn*$^{+/+}$ (Wild-type), *Ambn*$^{+/lacZ}$ (*Ambn*$^{+/-}$) heterozygous, and *Ambn*$^{lacZ/lacZ}$ (*Ambn*$^{-/-}$) null mice following soft tissue removal. These images show the molars immediately prior to their eruption into occlusion. The top and third rows are lingual views; the second and bottom rows are occlusal views. The null molars show thin cusps and surface accumulation of an irregular crusty material. Mild surface roughness of the heterozygous molar relative to the wild-type is most evident from the occlusal views.

References in Supplemental Material.

Hinnebusch, A. G., Ivanov, I. P., & Sonenberg, N. (2016). Translational control by 5'-untranslated regions of eukaryotic mRNAs. *Science, 352*(6292), 1413-1416. https://doi.org/10.1126/science.aad9868

Hu, C. C., Fukae, M., Uchida, T., Qian, Q., Zhang, C. H., Ryu, O. H., . . . Simmer, J. P. (1997). Sheathlin: cloning, cDNA/polypeptide sequences, and immunolocalization of porcine enamel sheath proteins. *J Dent Res, 76*(2), 648-657. https://doi.org/10.1177/00220345970760020501

Lu, T., Li, M., Xu, X., Xiong, J., Huang, C., Zhang, X., . . . Xiong, F. (2018). Whole exome sequencing identifies an AMBN missense mutation causing severe autosomal-dominant amelogenesis imperfecta and dentin disorders. *Int J Oral Sci., 10*(3), 26. https://doi.org/10.1038/s41368-018-0027-9

Poulter, J. A., Murillo, G., Brookes, S. J., Smith, C. E., Parry, D. A., Silva, S., . . . Mighell, A. J. (2014). Deletion of ameloblastin exon 6 is associated with amelogenesis imperfecta. *Hum. Mol. Genet., 23*(20), 5317-5324. https://doi.org/10.1093/hmg/ddu247

Prasad, M. K., Geoffroy, V., Vicaire, S., Jost, B., Dumas, M., Le Gras, S., . . . Bloch-Zupan, A. (2016). A targeted next-generation sequencing assay for the molecular diagnosis of genetic disorders with orodental involvement. *J. Med. Genet., 53*(2), 98-110. https://doi.org/10.1136/jmedgenet-2015-103302

Yang, J., Kawasaki, K., Lee, M., Reid, B. M., Nunez, S. M., Choi, M., . . . Hu, J. C. (2016). The dentin phosphoprotein repeat region and inherited defects of dentin. *Mol Genet Genomic Med, 4*(1), 28-38. https://doi.org/10.1002/mgg3.176
