# Supplementary material for: AMBN mutations causing hypoplastic amelogenesis imperfecta and Ambn knockout‐NLS‐lacZ knockin mice exhibiting failed amelogenesis and Ambn tissue‐specificity
Source: Mol Genet Genomic Med. 2019 Aug 11;7(9):e929. doi: 10.1002/mgg3.929 (PMC6732285; doi:10.1002/mgg3.929)
Supplement: Supplementary file 2 [file MGG3-7-e929-s002.pdf]

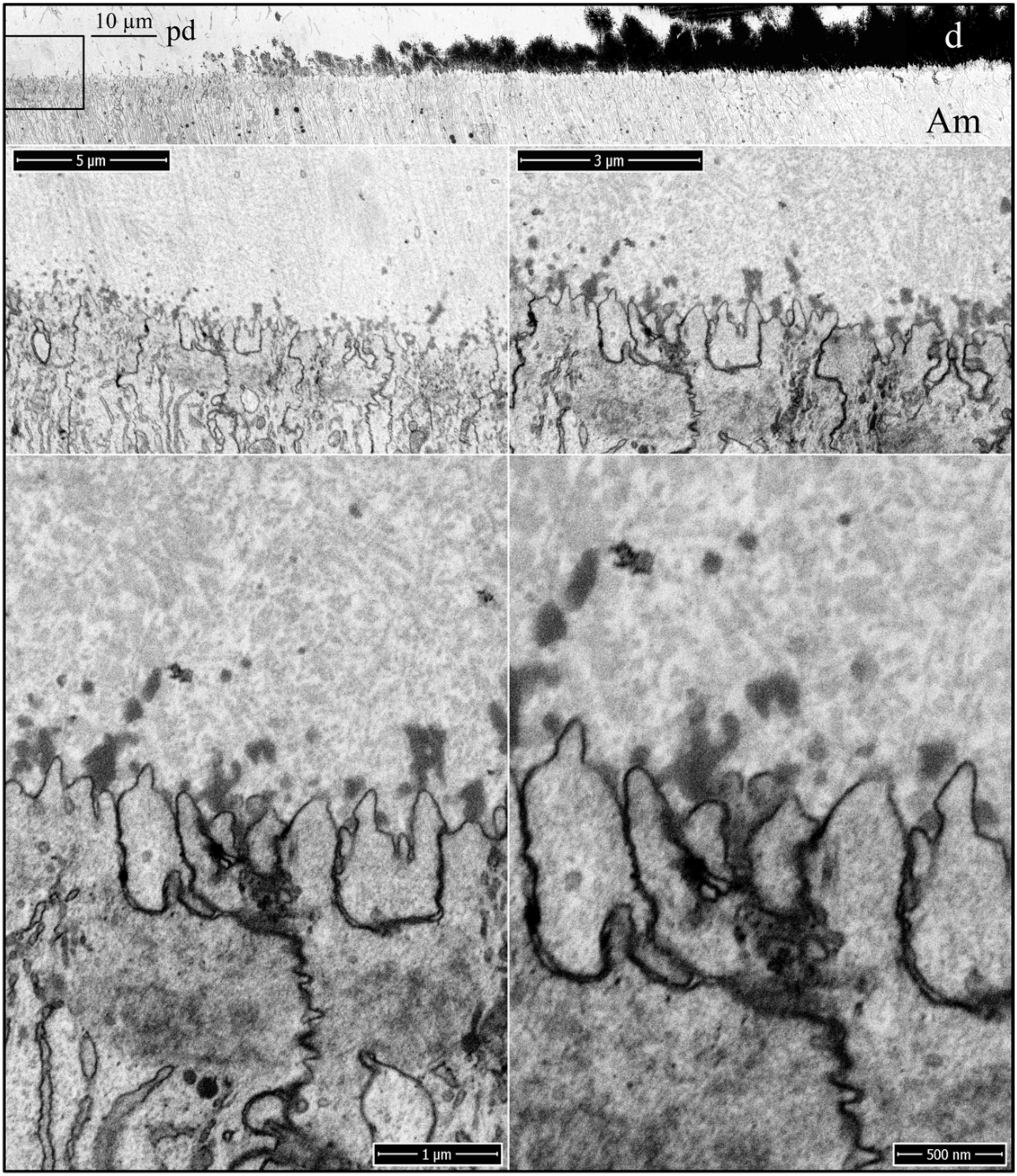

Figure S14. FIB-SEM images of the ameloblast-extracellular matrix interface ~1 mm from the apical loop (box 1) in an *Ambn*$^{lacZ/lacZ}$ mandibular incisor. ***Top:*** Montage of 155 µm wide segment taken at 5000x magnification. The box at the left shows the location of the 5000x (5 µm), 10000x (3 µm), 20000x (1 µm) and 35000x (500 nm) images shown below. **Key:** Am, ameloblast; D, dentin; pD, predentin. The basement membrane has been degraded and finger-like ameloblast process extend into the predentin. The darker material in the predentin matrix is mainly amelogenin.

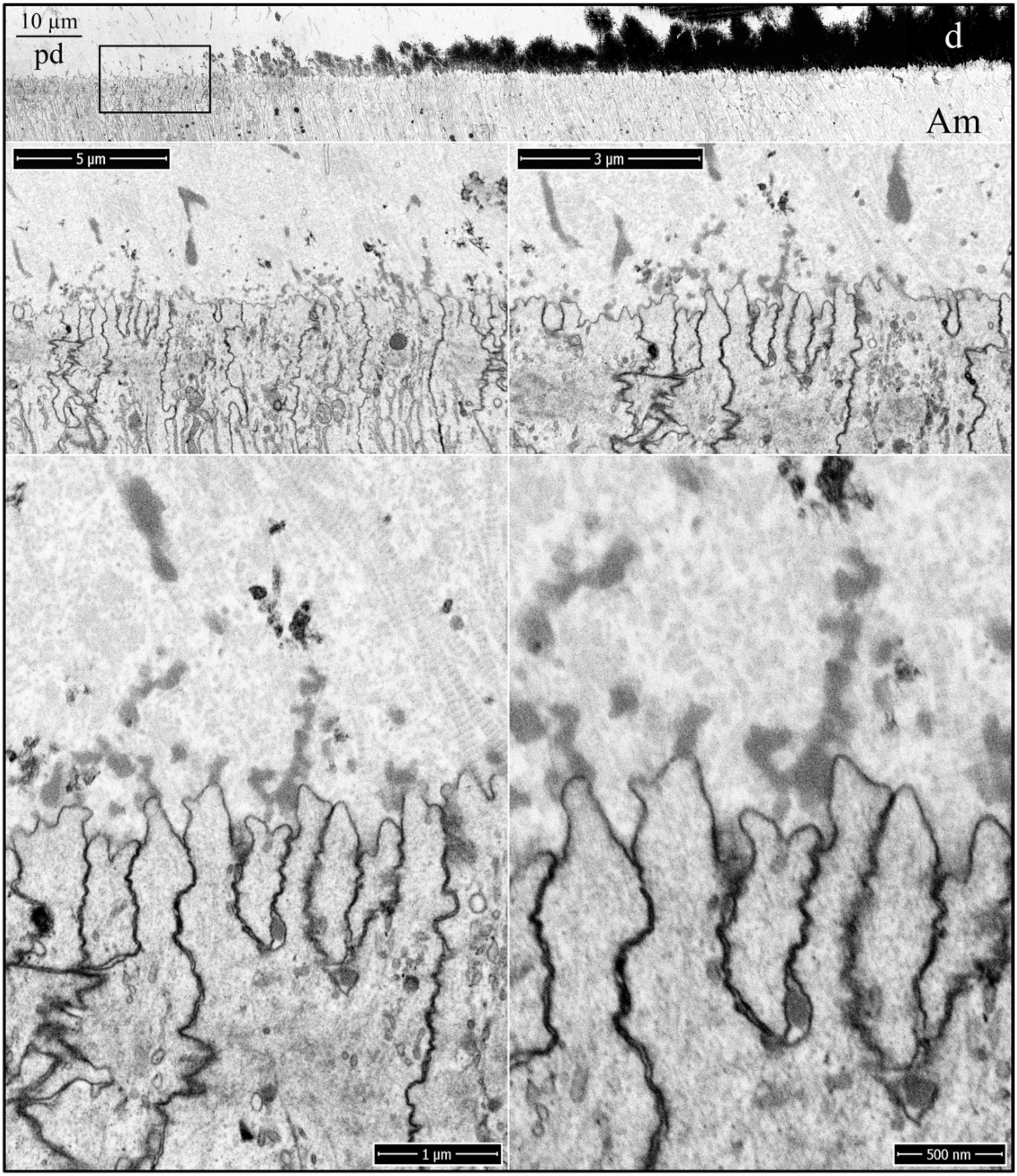

Figure S15. FIB-SEM images of the ameloblast-extracellular matrix interface ~1 mm from the apical loop (box 2) in an *Ambn*$^{lacZ/lacZ}$ mandibular incisor. ***Top:*** Montage of 155 µm wide segment taken at 5000x magnification. The box shows the location of the higher magnification images shown below. **Key:** Am, ameloblast; D, dentin; pD, predentin. The dark mineral foci herald the onset of dentin mineralization. Many mineral foci form and will coalesce into a continuous mineral layer and expand to associate with the ameloblast finger-like projections.

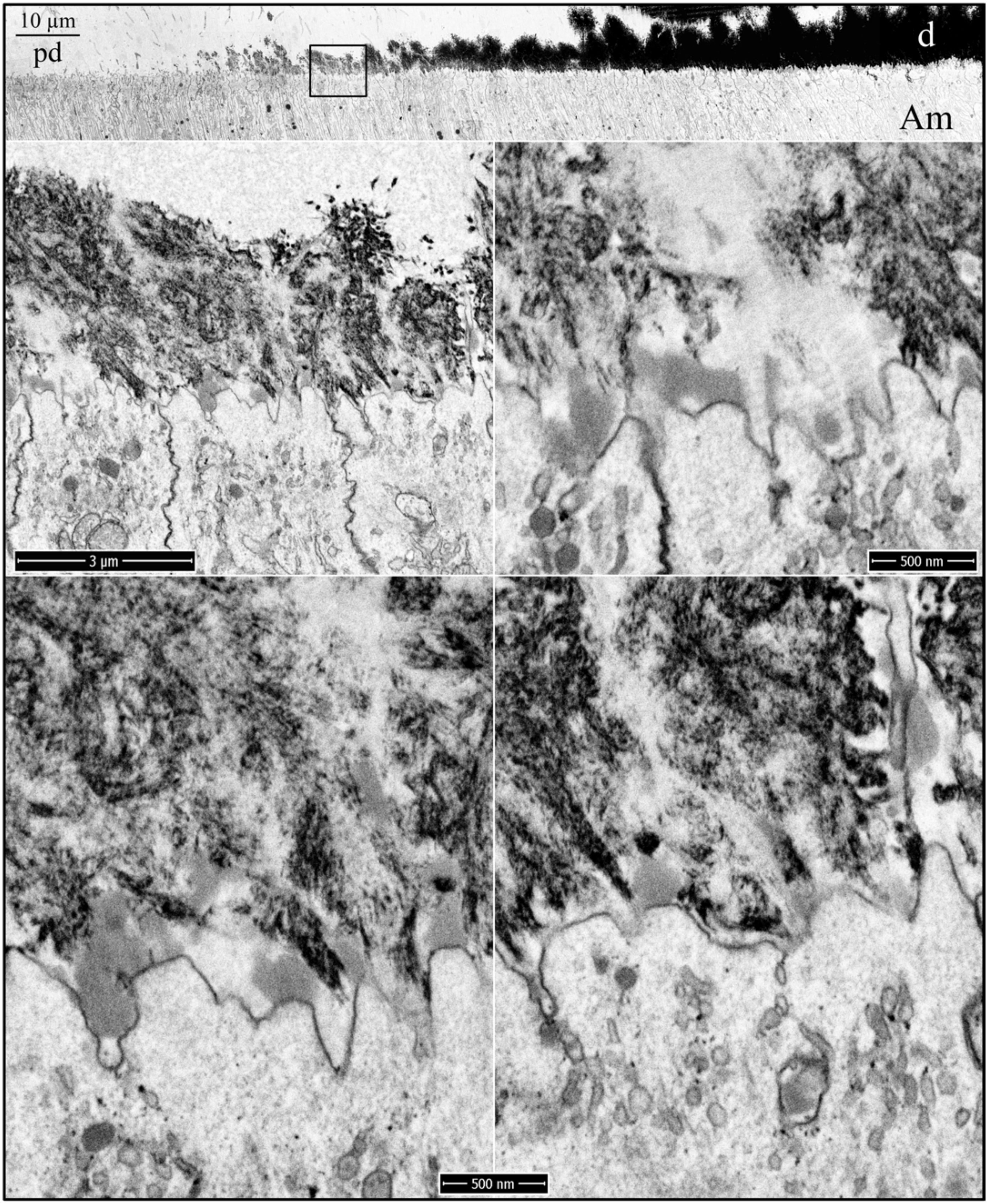

Figure S16. FIB-SEM images of the ameloblast-extracellular matrix interface ~1 mm from the apical loop (box 3) in an *Ambn*$^{lacZ/lacZ}$ mandibular incisor. ***Top:*** Montage of 155 µm wide segment taken at 5000x magnification. The box shows the location of the higher magnification images shown below. **Key:** Am, ameloblast; D, dentin; pD, predentin. The dentin has coalesced into a continuous mineral layer. The size and abundance of the gray patches (mostly amelogenin) on the dentin surface already exceeds what appears in wild-type incisors and is a cardinal feature of the *Ambn* null incisors.

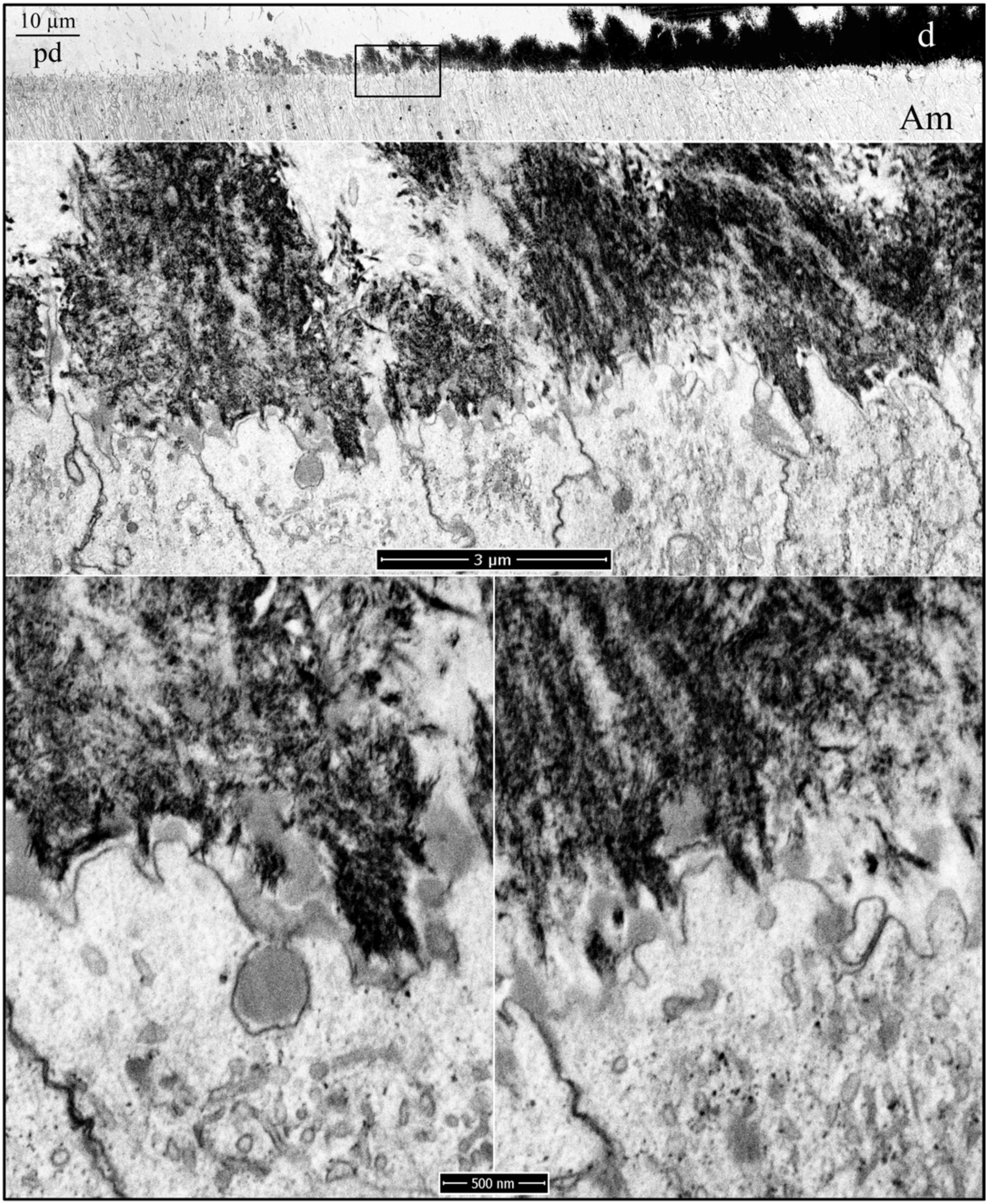

Figure S17. FIB-SEM images of the ameloblast-extracellular matrix interface ~1 mm from the apical loop (box 4) in an *Ambn*$^{lacZ/lacZ}$ mandibular incisor. ***Top:*** Montage of 155 µm wide segment taken at 5000x magnification. The box shows the location of the higher magnification images shown below. **Key:** Am, ameloblast; D, dentin; pD, predentin. The dentin has coalesced into a continuous, highly mineralized layer. Despite this, enamel mineral ribbons have not formed and enamel protein (mainly amelogenin) accumulates.

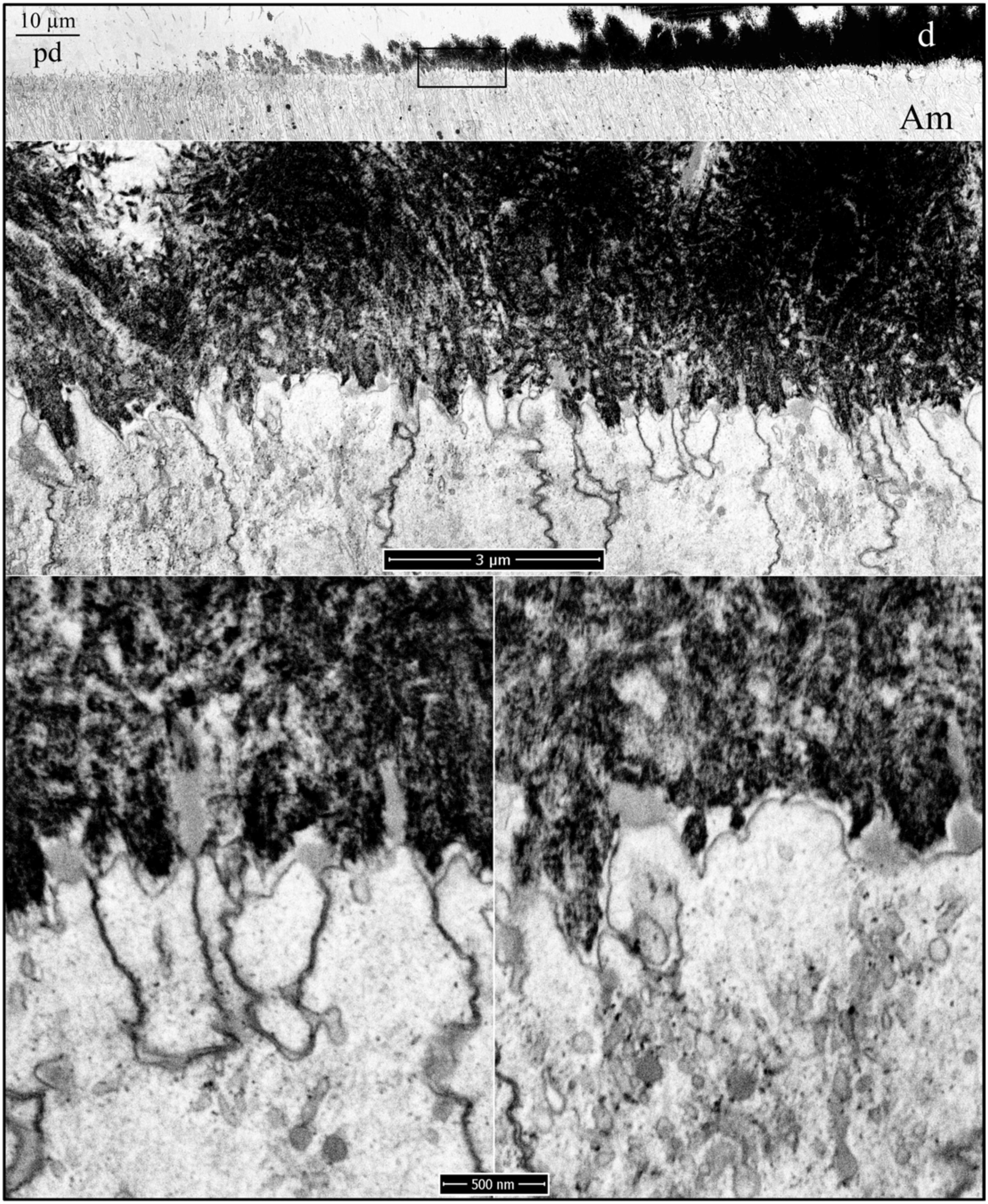

Figure S18. FIB-SEM images of the ameloblast-extracellular matrix interface ~1 mm from the apical loop (box 5) in an *Ambn*$^{lacZ/lacZ}$ mandibular incisor. ***Top:*** Montage of 155 µm wide segment taken at 5000x magnification. The box shows the location of the higher magnification images shown below. **Key:** Am, ameloblast; D, dentin; pD, predentin. Dentin continues to mineralize, enamel protein (mainly amelogenin) accumulates, no enamel ribbons form.

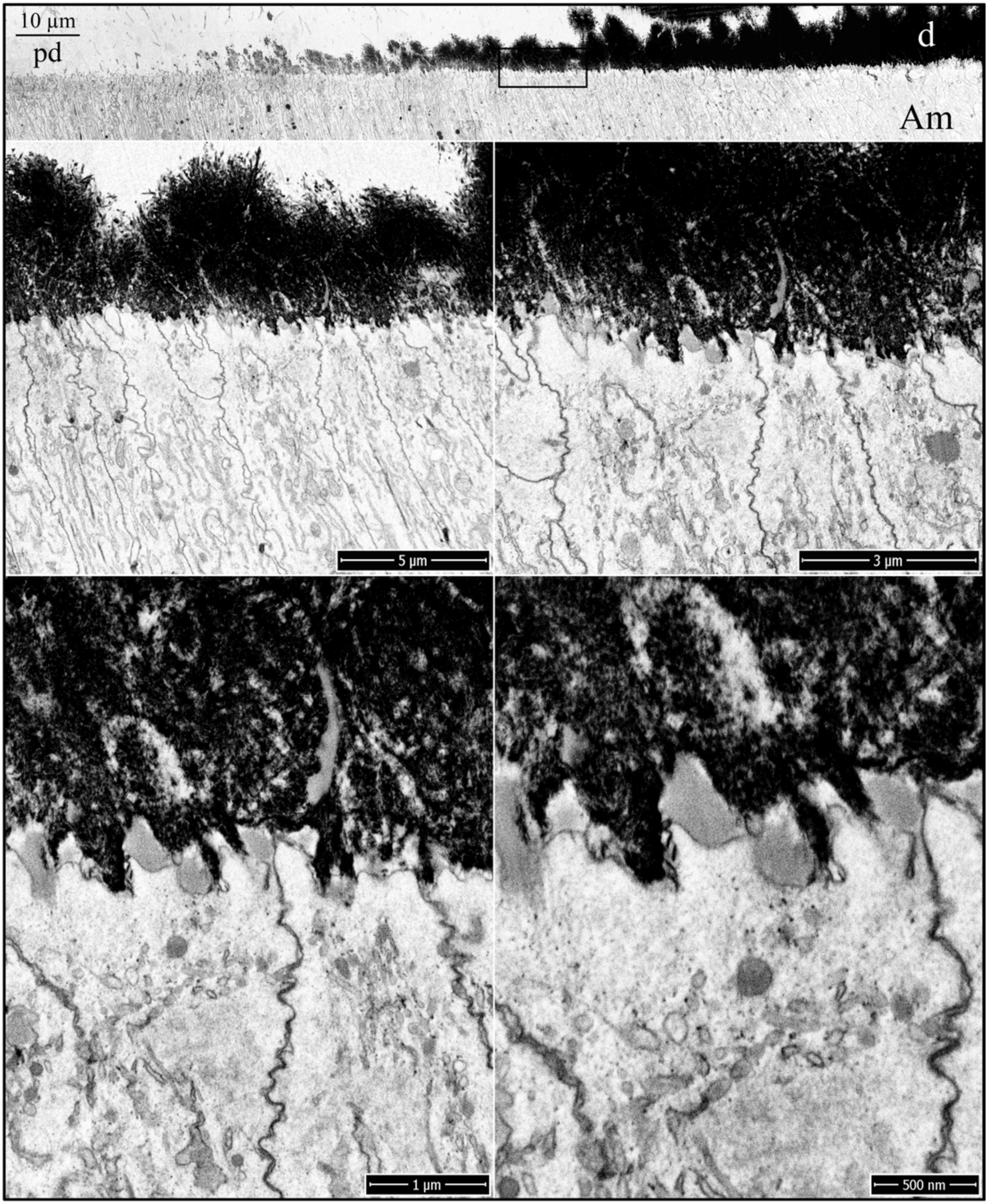

Figure S19. FIB-SEM images of the ameloblast-extracellular matrix interface ~1 mm from the apical loop (box 6) in an *Ambn*$^{lacZ/lacZ}$ mandibular incisor. ***Top:*** Montage of 155 µm wide segment taken at 5000x magnification. The box shows the location of the higher magnification images shown below. **Key:** Am, ameloblast; D, dentin; pD, predentin. Dentin continues to mineralize, amelogenin accumulates, no enamel ribbons form.

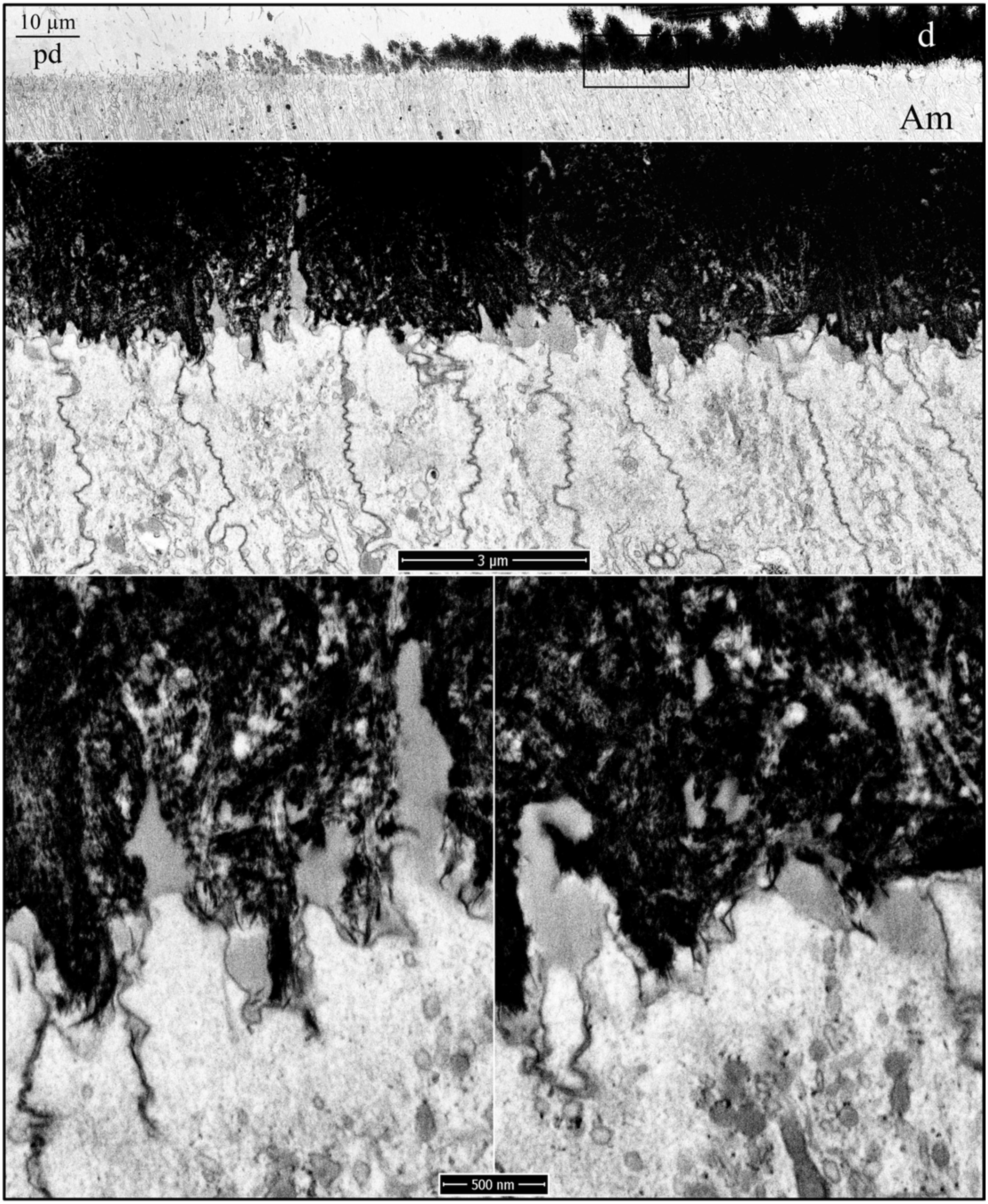

Figure S20. FIB-SEM images of the ameloblast-extracellular matrix interface ~1 mm from the apical loop (box 7) in an *Ambn*$^{lacZ/lacZ}$ mandibular incisor. ***Top:*** Montage of 155 µm wide segment taken at 5000x magnification. The box shows the location of the higher magnification images shown below. **Key:** Am, ameloblast; D, dentin; pD, predentin. Dentin continues to mineralize, amelogenin accumulates, no enamel ribbons form.

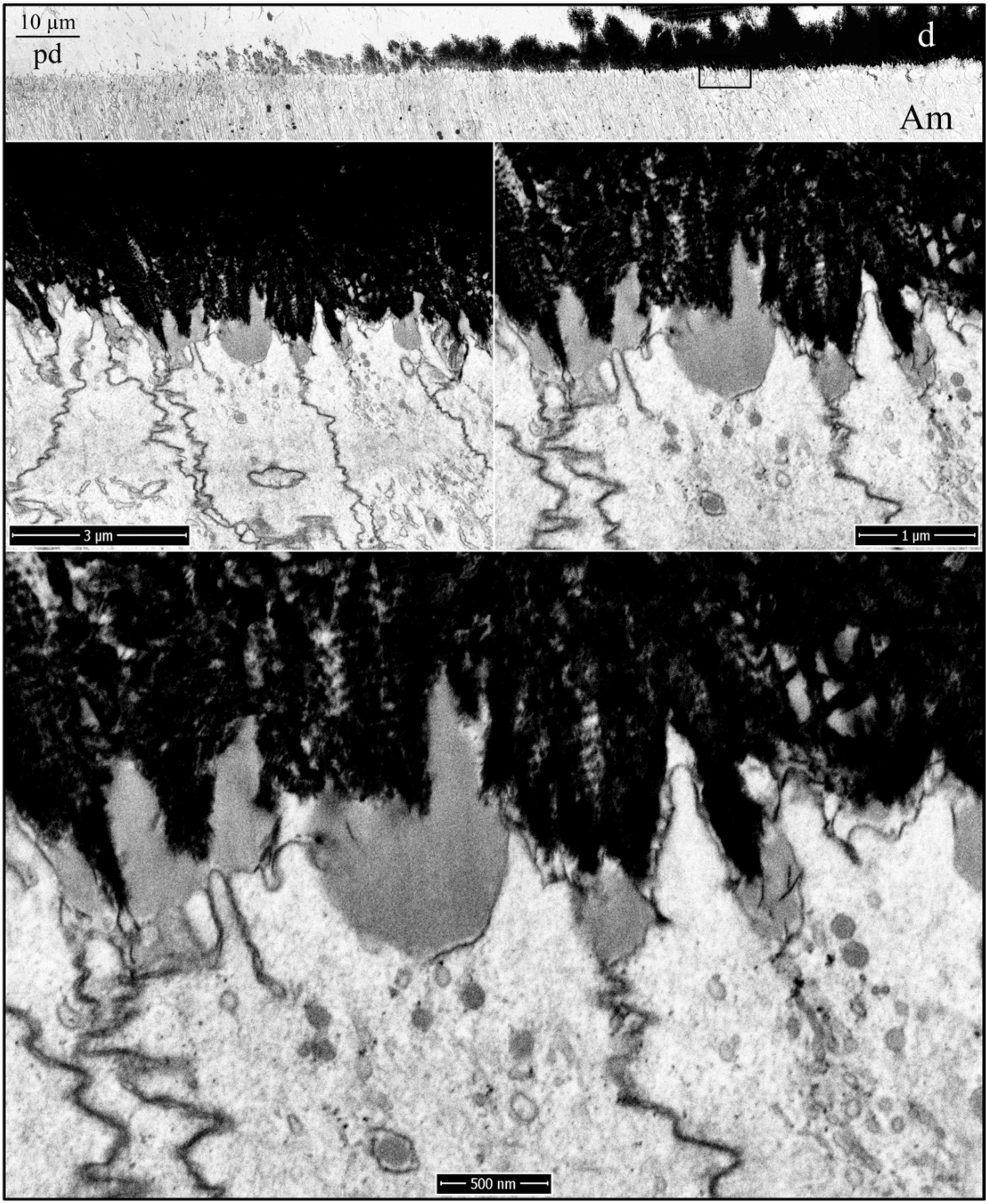

Figure S21. FIB-SEM images of the ameloblast-extracellular matrix interface ~1 mm from the apical loop (box 8) in an *Ambn*$^{lacZ/lacZ}$ mandibular incisor. ***Top:*** Montage of 155 µm wide segment taken at 5000x magnification. The box shows the location of the higher magnification images shown below. **Key:** Am, ameloblast; D, dentin; pD, predentin. Dentin continues to mineralize, amelogenin accumulates, no enamel ribbons form.

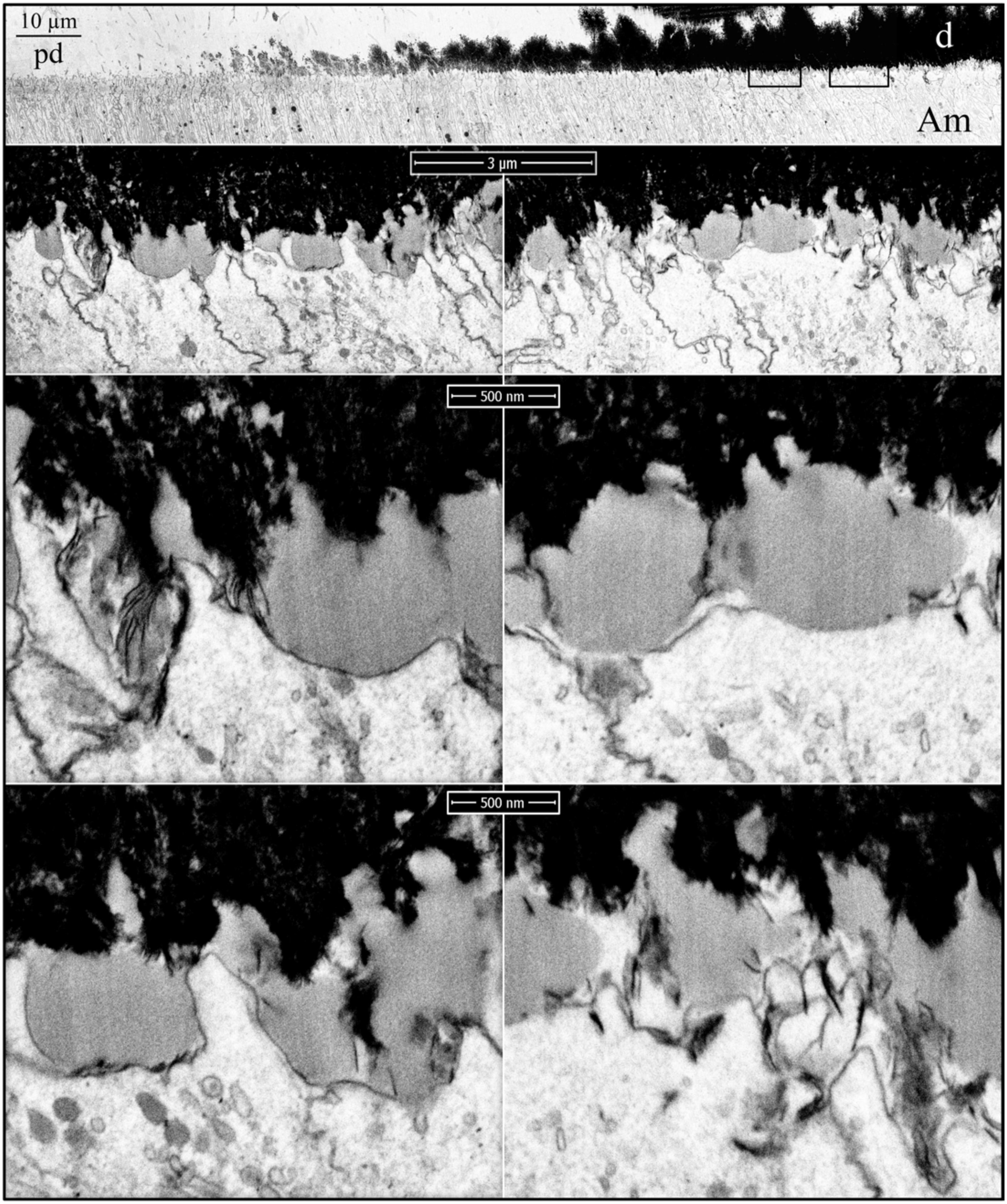

Figure S22. FIB-SEM images of the ameloblast-extracellular matrix interface ~1 mm from the apical loop (box 9) in an *Ambn*$^{lacZ/lacZ}$ mandibular incisor. ***Top:*** Montage of 155 µm wide segment taken at 5000x magnification. The box shows the location of the higher magnification images shown below. **Key:** Am, ameloblast; D, dentin; pD, predentin. Dentin continues to mineralize, amelogenin accumulates, no enamel ribbons form. Some curved ribbon-like mineral is deposited near the surface of ameloblast distal membrane.

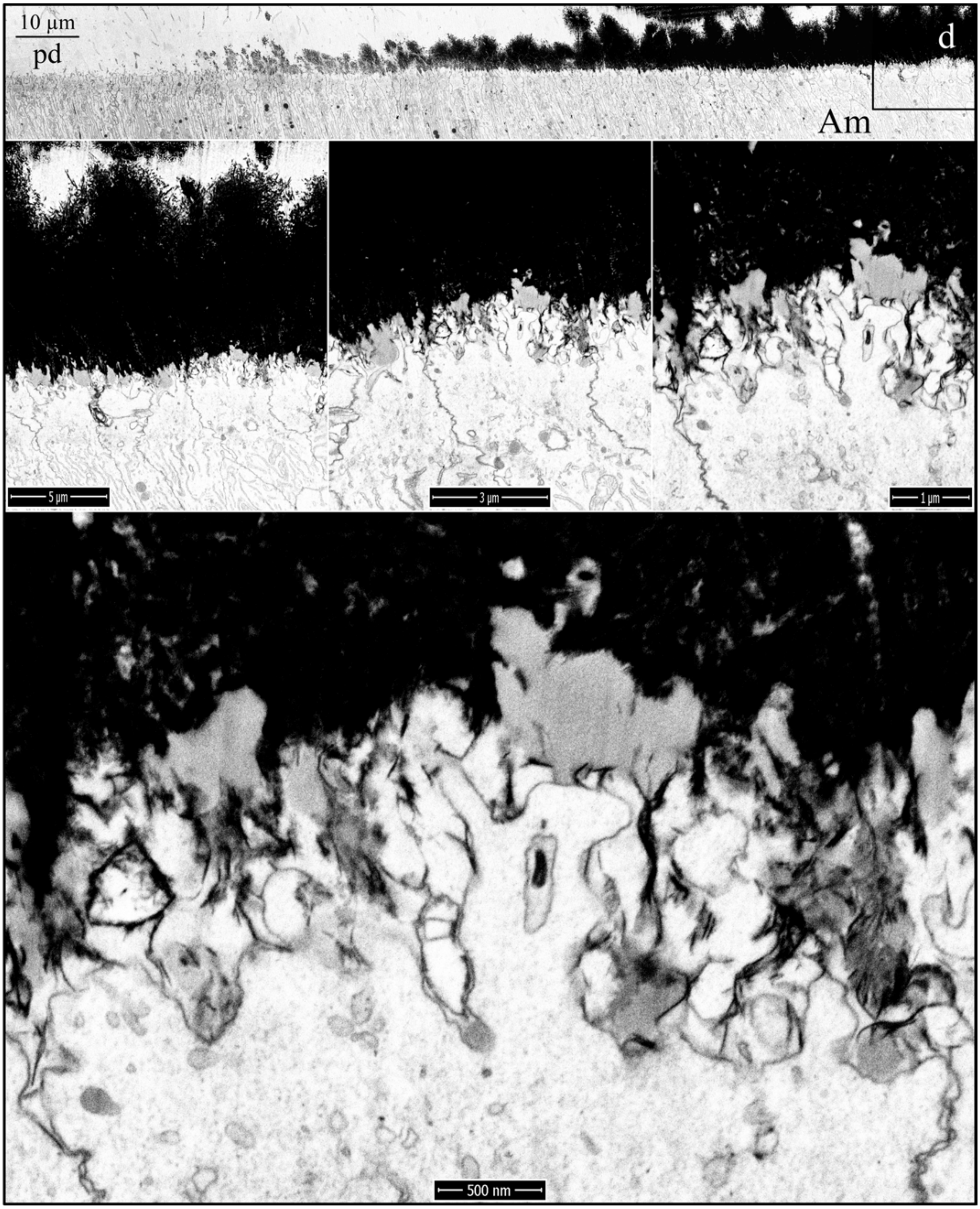

Figure S23. FIB-SEM images of the ameloblast-extracellular matrix interface ~1 mm from the apical loop (box 10) in an *Ambn*$^{lacZ/lacZ}$ mandibular incisor. ***Top:*** Montage of 155 μm wide segment taken at 5000x magnification. The box shows the location of the higher magnification images shown below. **Key:** Am, ameloblast; D, dentin; pD, predentin. Dentin continues to mineralize, amelogenin accumulates, no enamel ribbons form. Some curved ribbon-like mineral is deposited near the surface of ameloblast distal membrane.
