# Supplementary material for: AMBN mutations causing hypoplastic amelogenesis imperfecta and Ambn knockout‐NLS‐lacZ knockin mice exhibiting failed amelogenesis and Ambn tissue‐specificity
Source: Mol Genet Genomic Med. 2019 Aug 11;7(9):e929. doi: 10.1002/mgg3.929 (PMC6732285; doi:10.1002/mgg3.929)
Supplement: Supplementary file 3 [file MGG3-7-e929-s003.pdf]

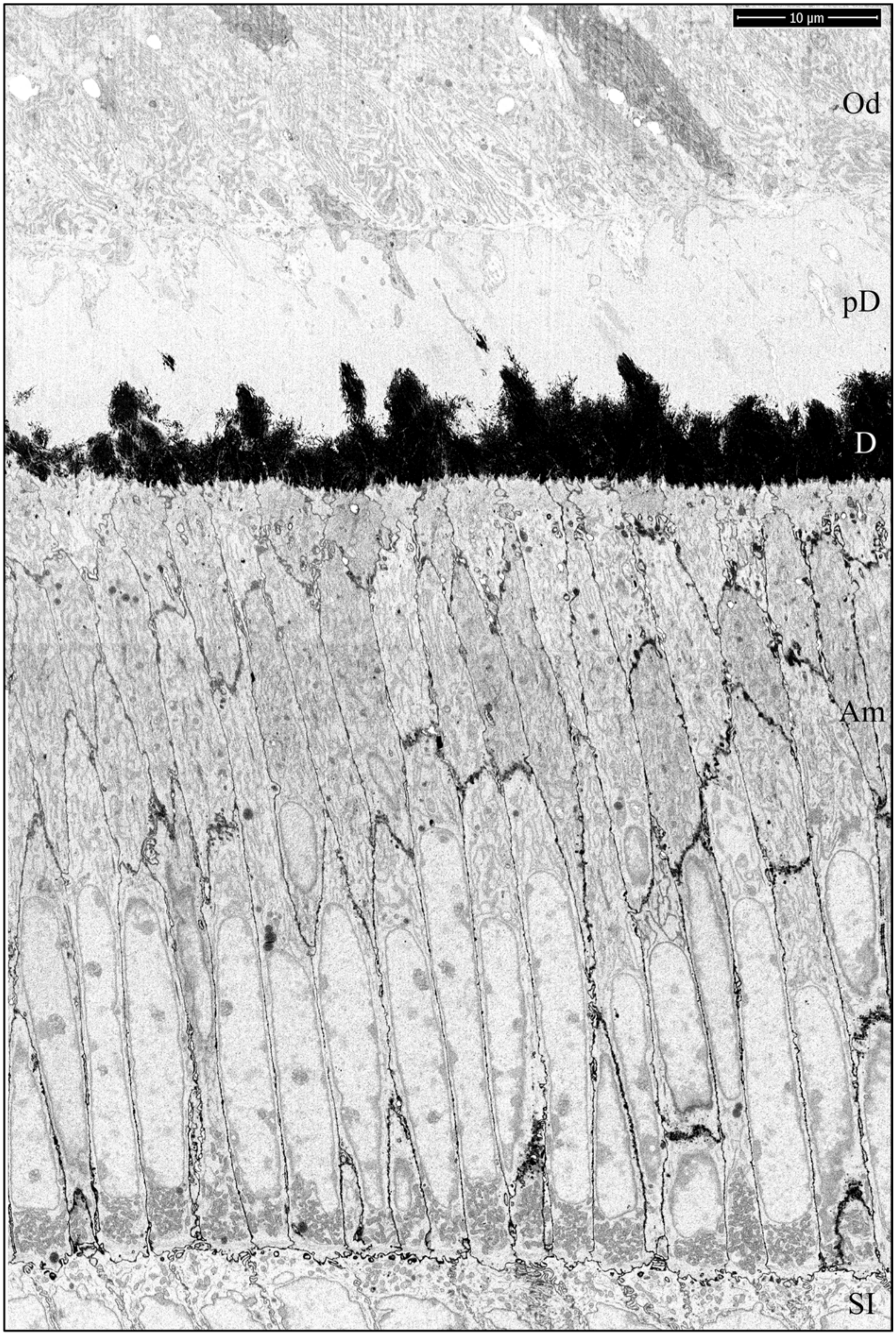

Figure S24 Region 3. Montage of *Ambn*/*Amelx* double null FIB-SEM images (2500x TLD) showing a lack of enamel initiation despite progression of dentin mineralization to almost 10 µm in thickness (Fig. S34). ***Key:*** Am: Ameloblast; Od: Odontoblast; pD: predentin; SI: stratum intermedium.

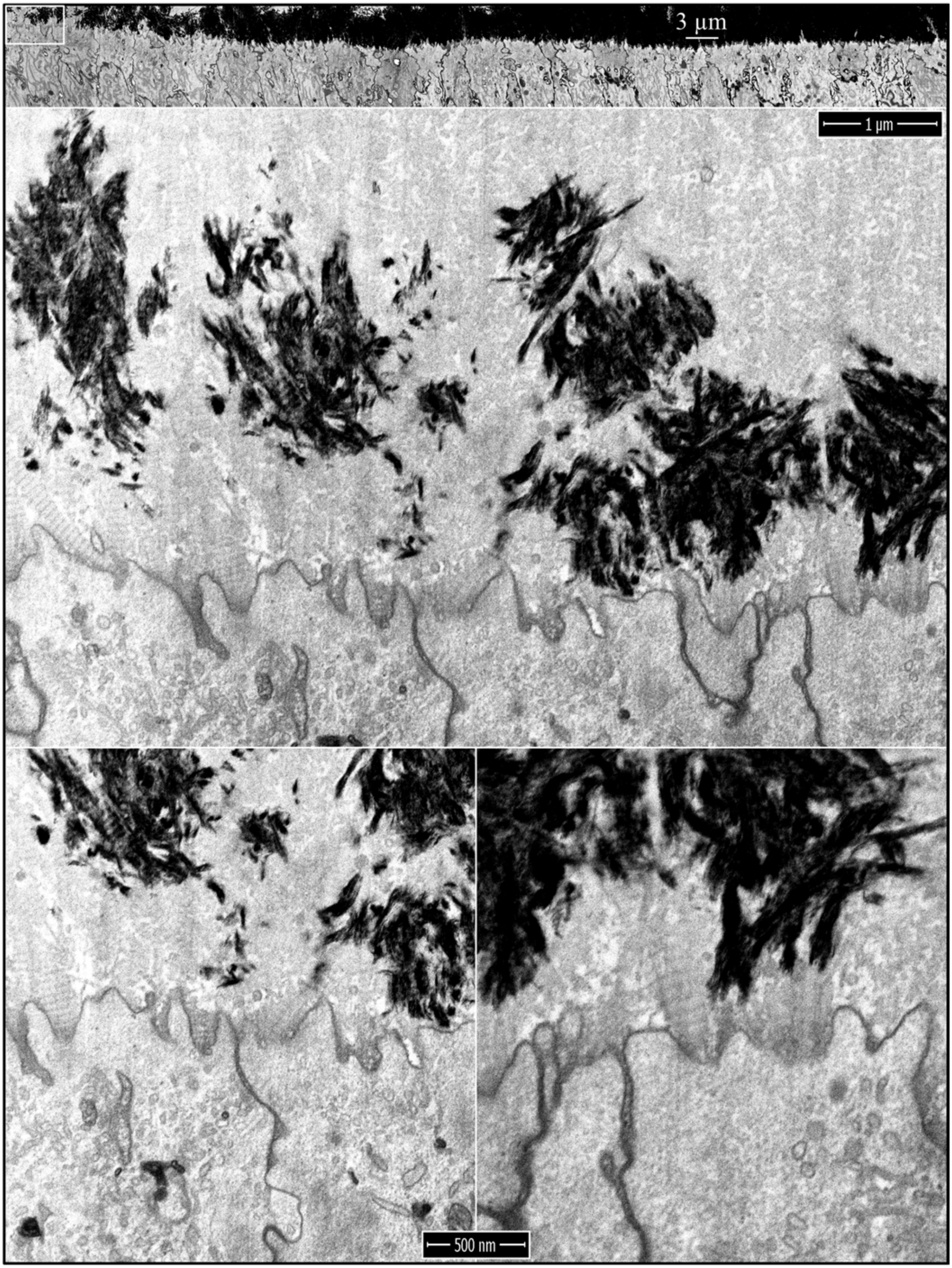

Figure S25 Region 3a. *Ambn*/*Amelx* double null FIB-SEM images. ***Top:*** Montage of 10000x TLD images. ***Middle:*** Montage of 20000x TLD images from boxed area. ***Bottom:*** 35000x TLD images. Note the expansion of dentin mineral, often extending along collagen fibers and extending near the ameloblast membrane.

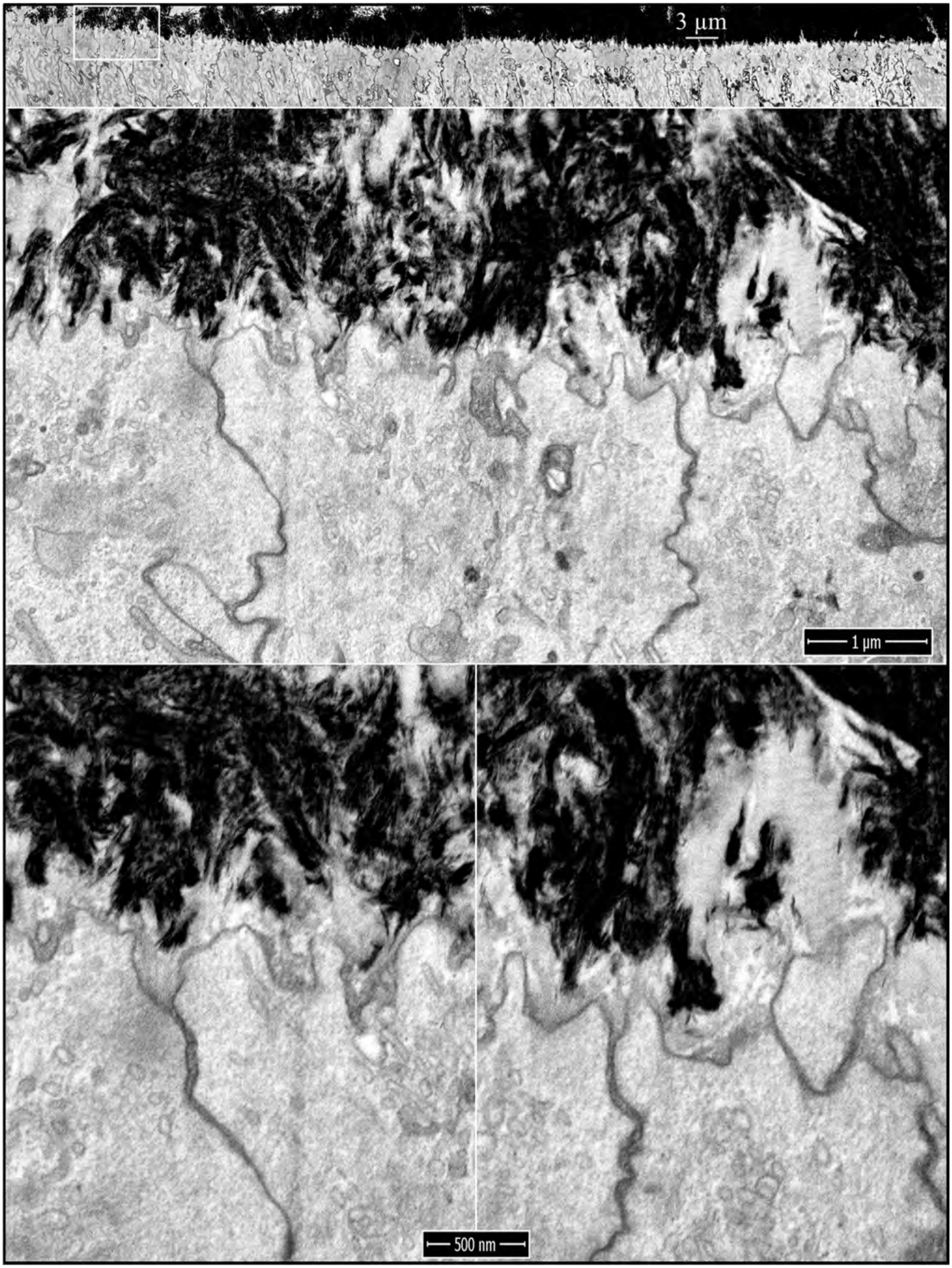

Figure S26 Region 3b. *Ambn*/*Amelx* double null FIB-SEM images. ***Top:*** Montage of 10000x TLD images. ***Middle:*** Montage of 20000x TLD images from boxed area. ***Bottom:*** 35000x TLD images. Note dentin mineral has coallesced into a continuous layer and closely approximates the ameloblast membrane. The initiation of characteristic enamel ribbons would normally occur approximately at this position.

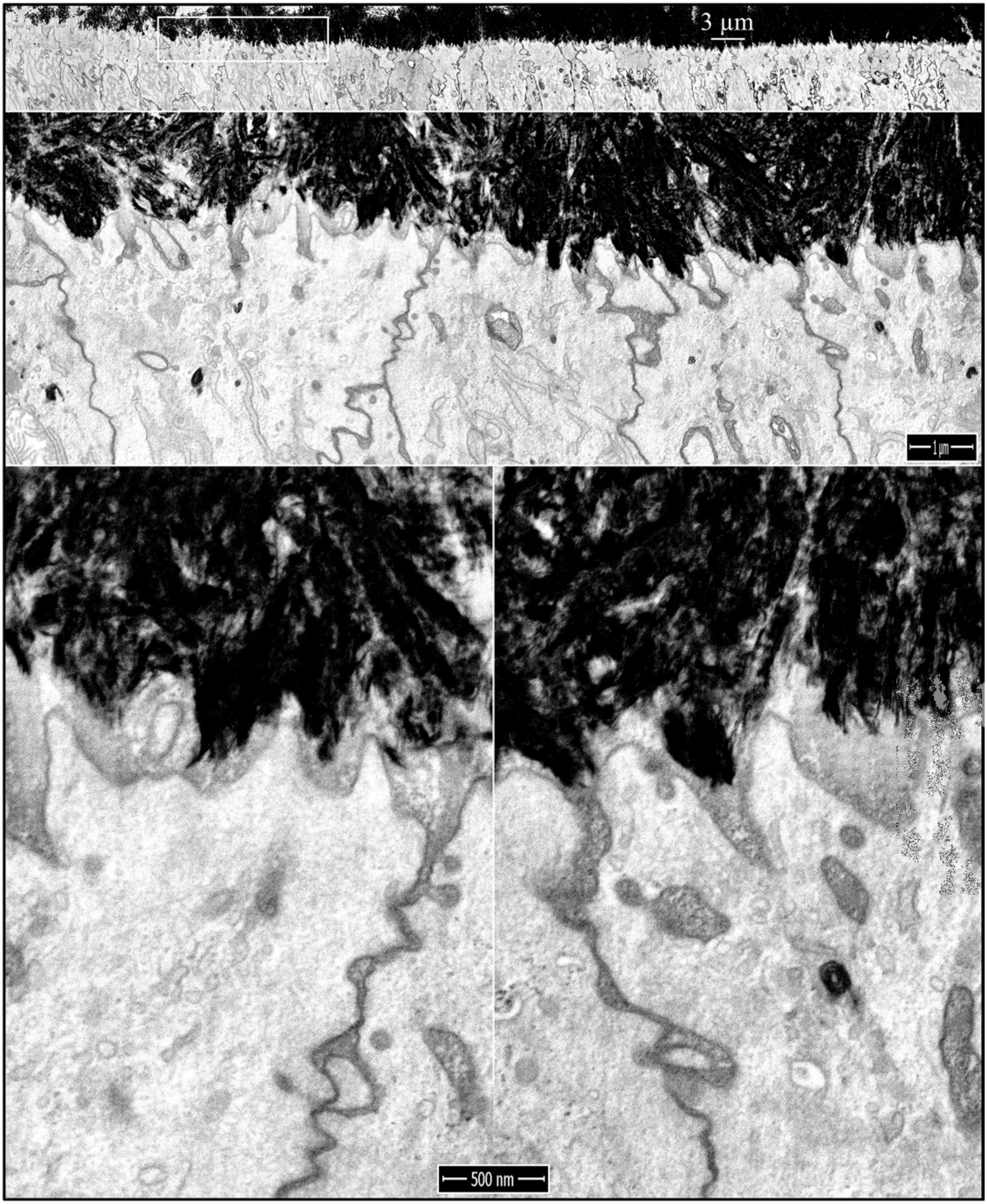

Figure S27 Region 3c. *Ambn*/*Amelx* double null FIB-SEM images. ***Top:*** Montage of 10000x TLD images. ***Middle:*** Montage of 20000x TLD images from boxed area. ***Bottom:*** 35000x TLD images.

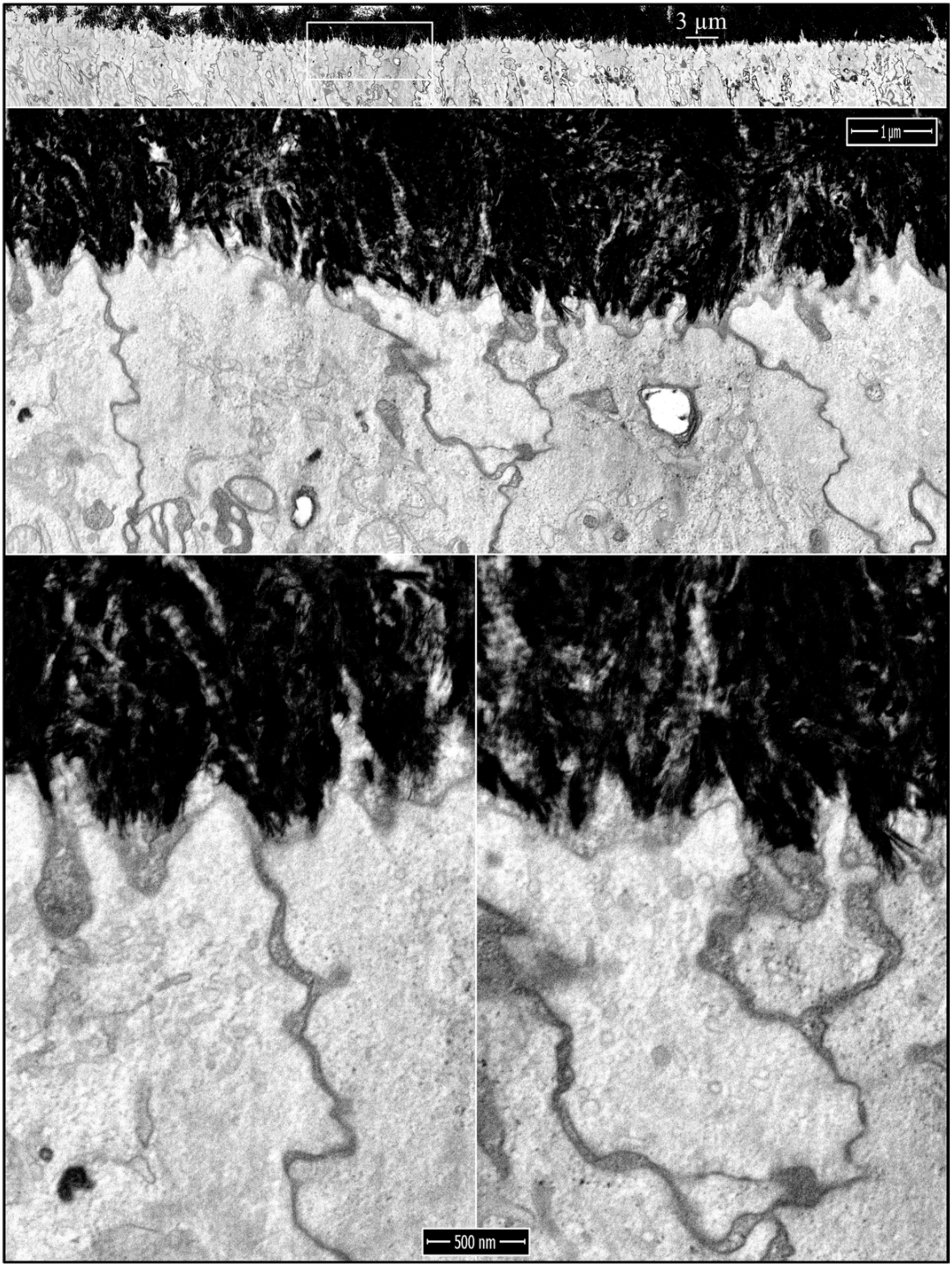

Figure S28 Region 3d. *Ambn*/*Amelx* double null FIB-SEM images. ***Top:*** Montage of 10000x TLD images. ***Middle:*** Montage of 20000x TLD images from boxed area. ***Bottom:*** 35000x TLD images.

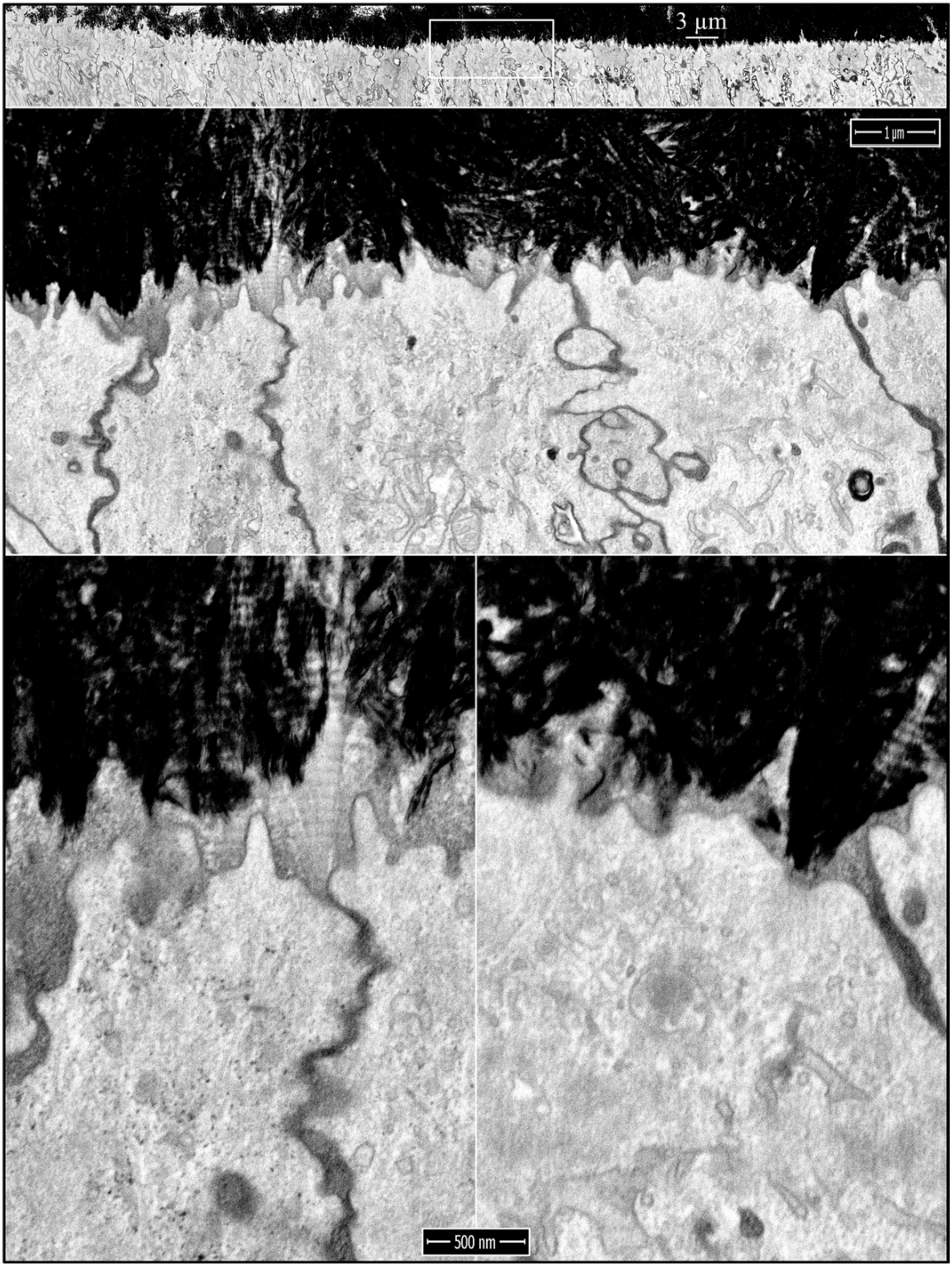

Figure S29 Region 3e. *Ambn*/*Amelx* double null FIB-SEM images. ***Top:*** Montage of 10000x TLD images. ***Middle:*** Montage of 20000x TLD images from boxed area. ***Bottom:*** 35000x TLD images.

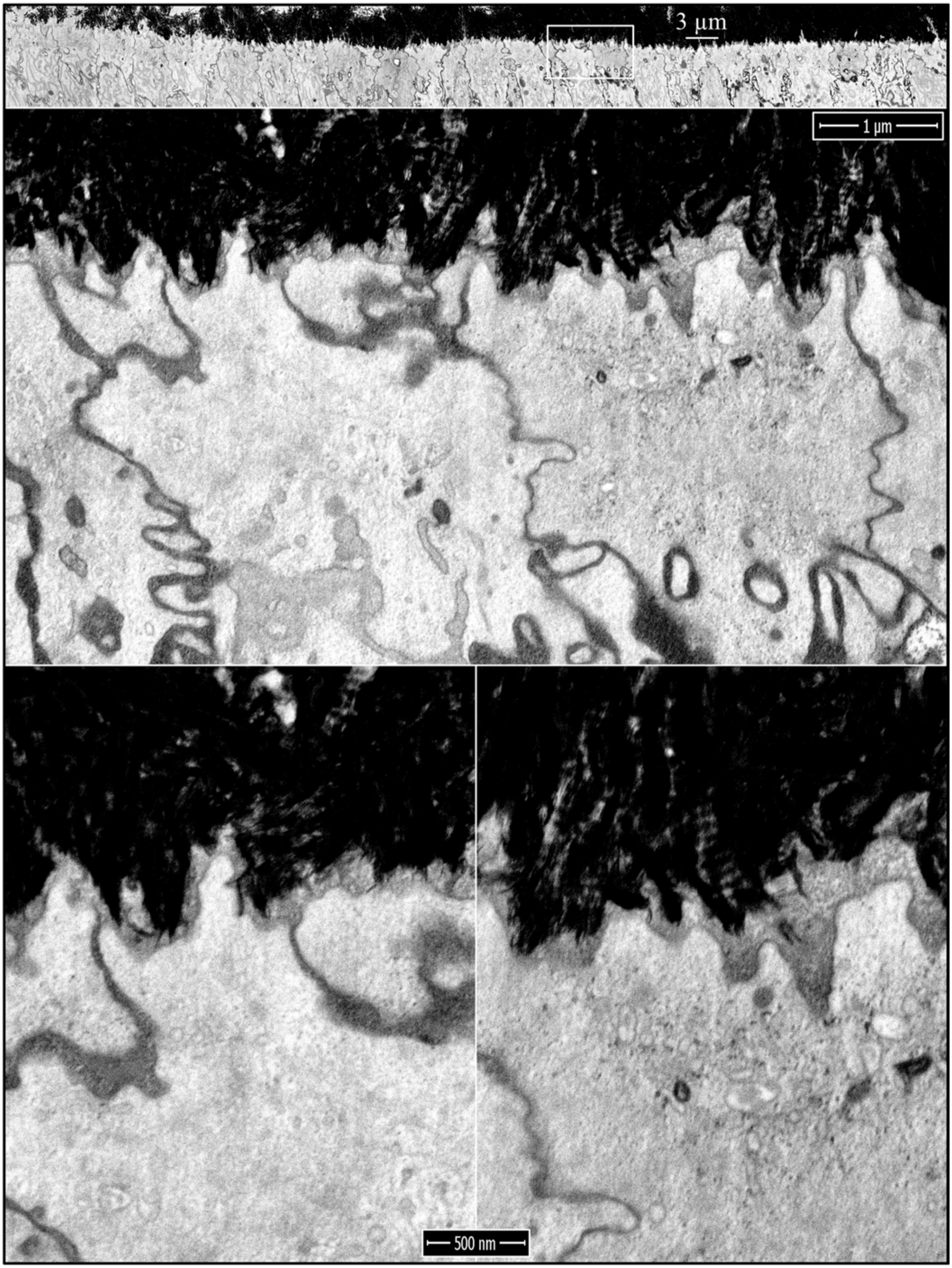

Figure S30 Region 3f. *Ambn*/*Amelx* double null FIB-SEM images. ***Top:*** Montage of 10000x TLD images. ***Middle:*** Montage of 20000x TLD images from boxed area. ***Bottom:*** 35000x TLD images.

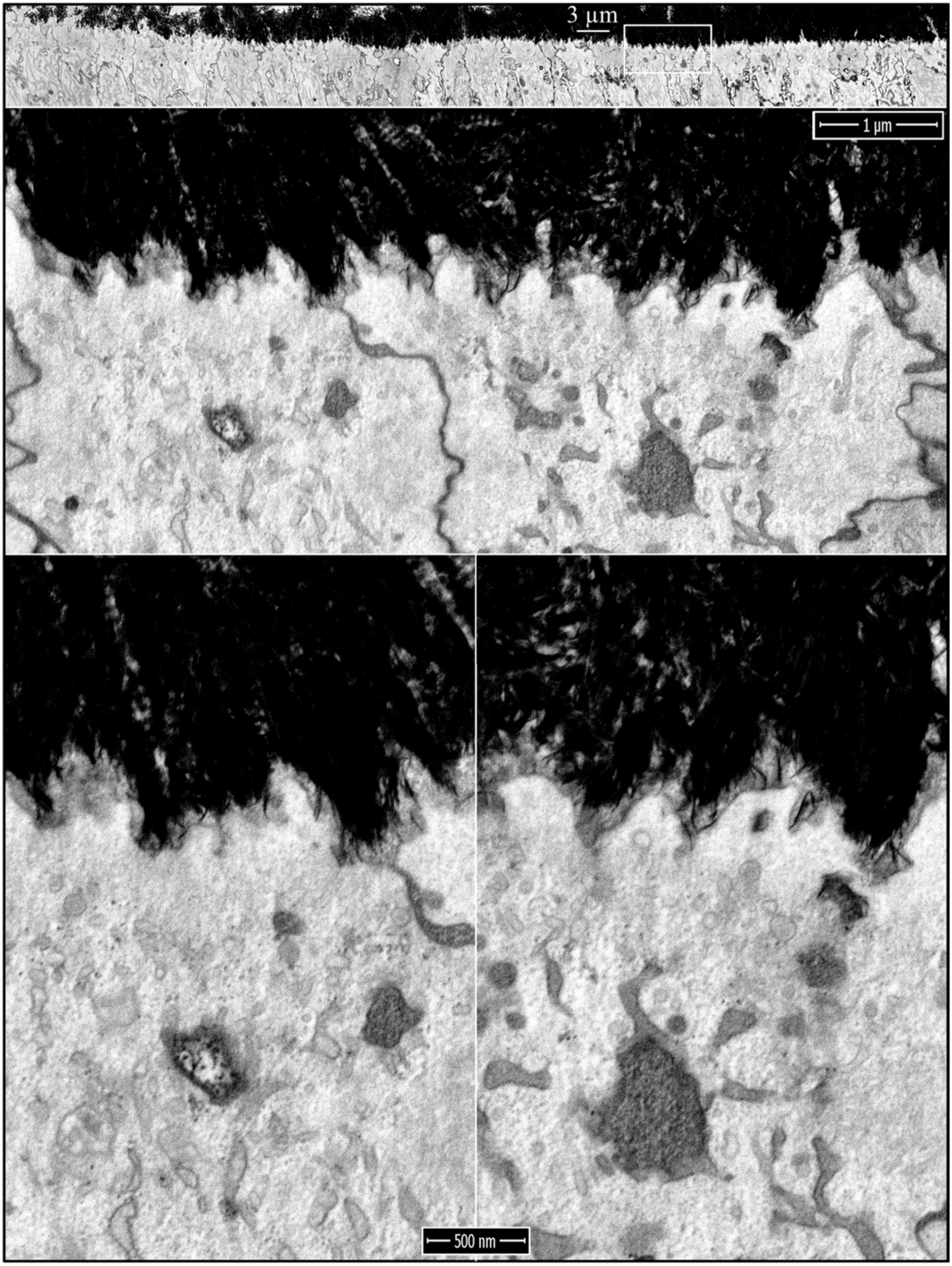

Figure S31 Region 3g. *Ambn*/*Amelx* double null FIB-SEM images. ***Top:*** Montage of 10000x TLD images. ***Middle:*** Montage of 20000x TLD images from boxed area. ***Bottom:*** 35000x TLD images.

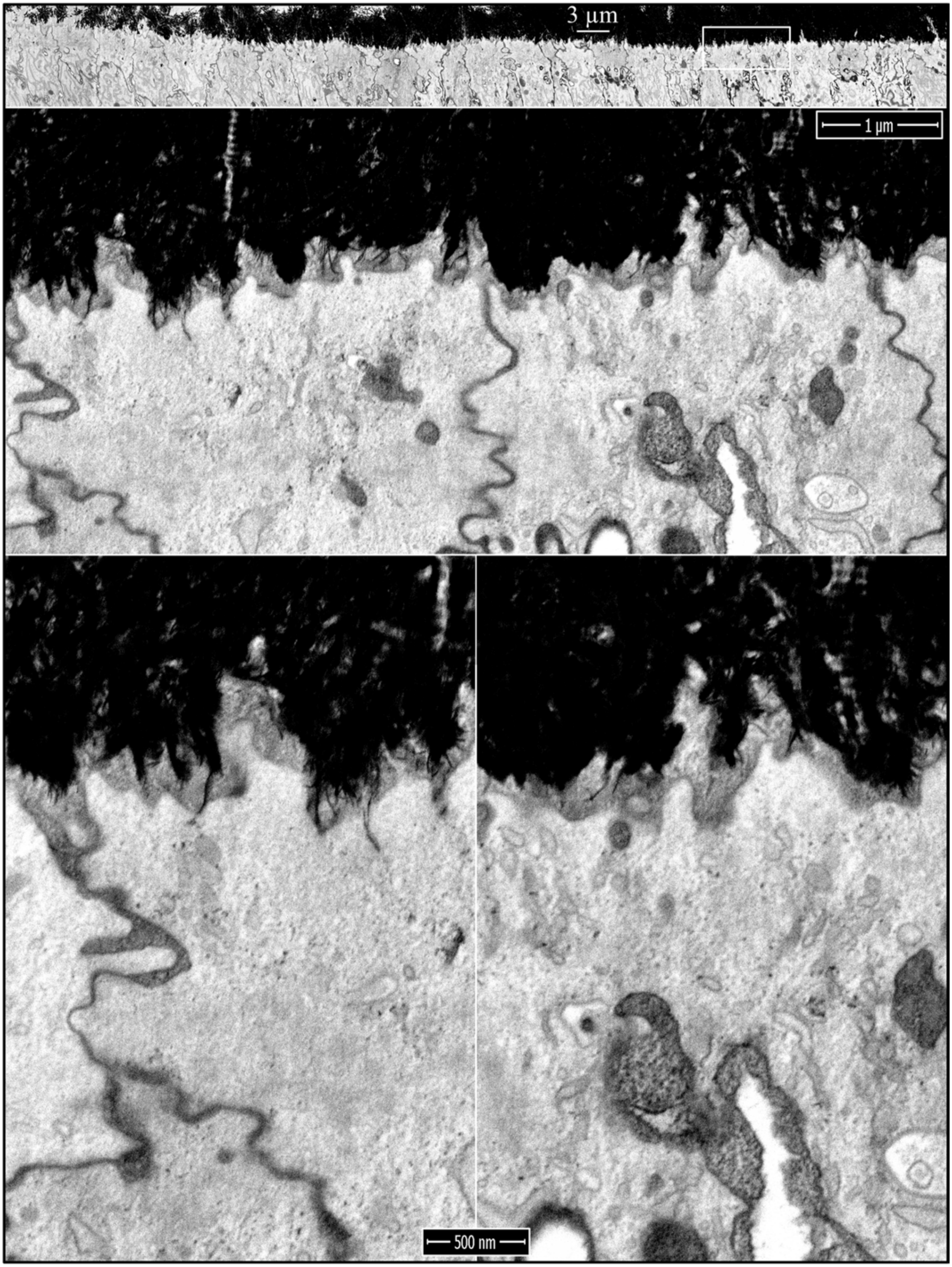

Figure S32 Region 3h. *Ambn*/*Amelx* double null FIB-SEM images. ***Top:*** Montage of 10000x TLD images. ***Middle:*** Montage of 20000x TLD images from boxed area. ***Bottom:*** 35000x TLD images.

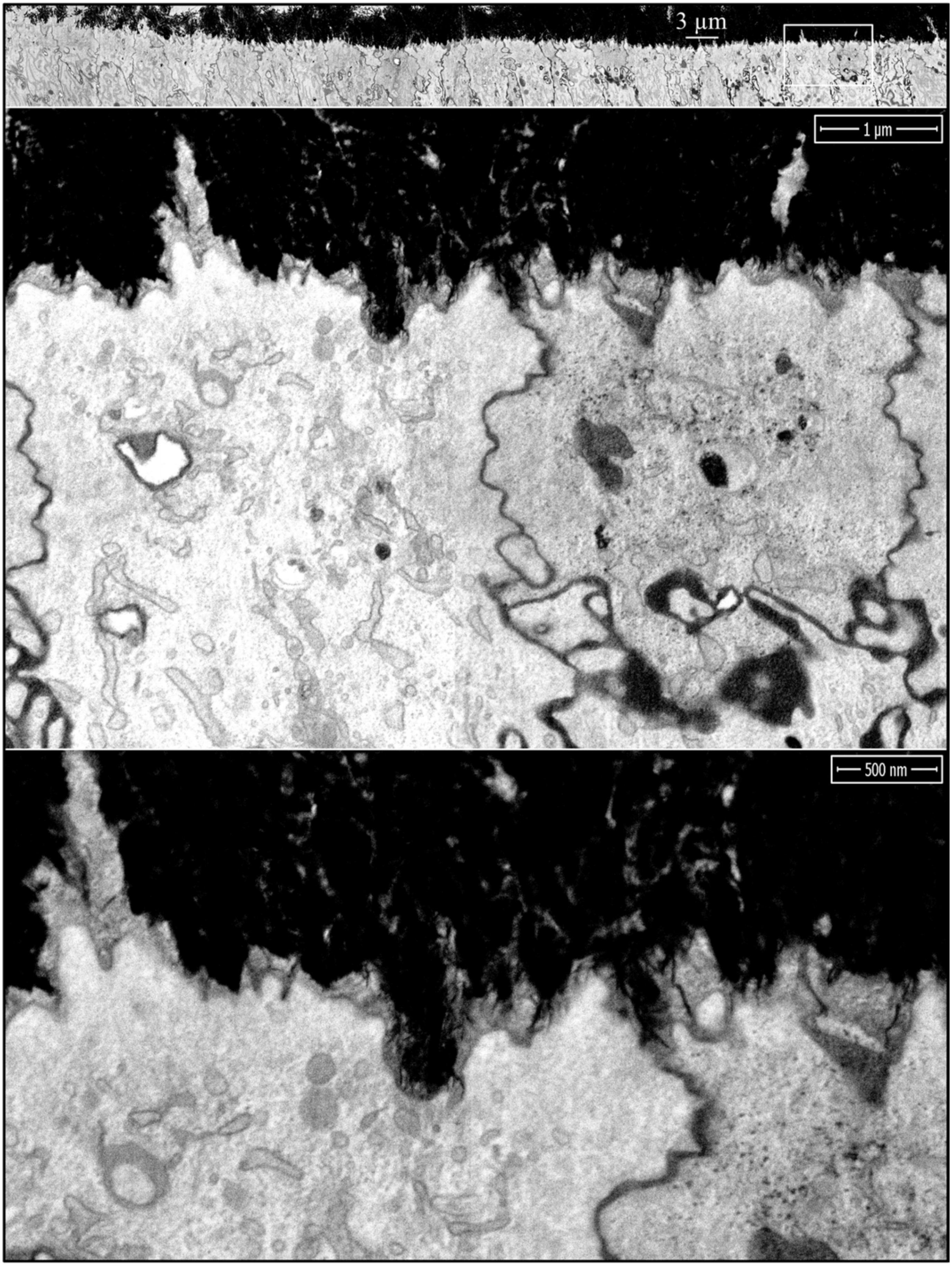

Figure S33 Region 3i. *Ambn*/*Amelx* double null FIB-SEM images. ***Top:*** Montage of 10000x TLD images. ***Middle:*** Montage of 20000x TLD images from boxed area. ***Bottom:*** 35000x TLD images.

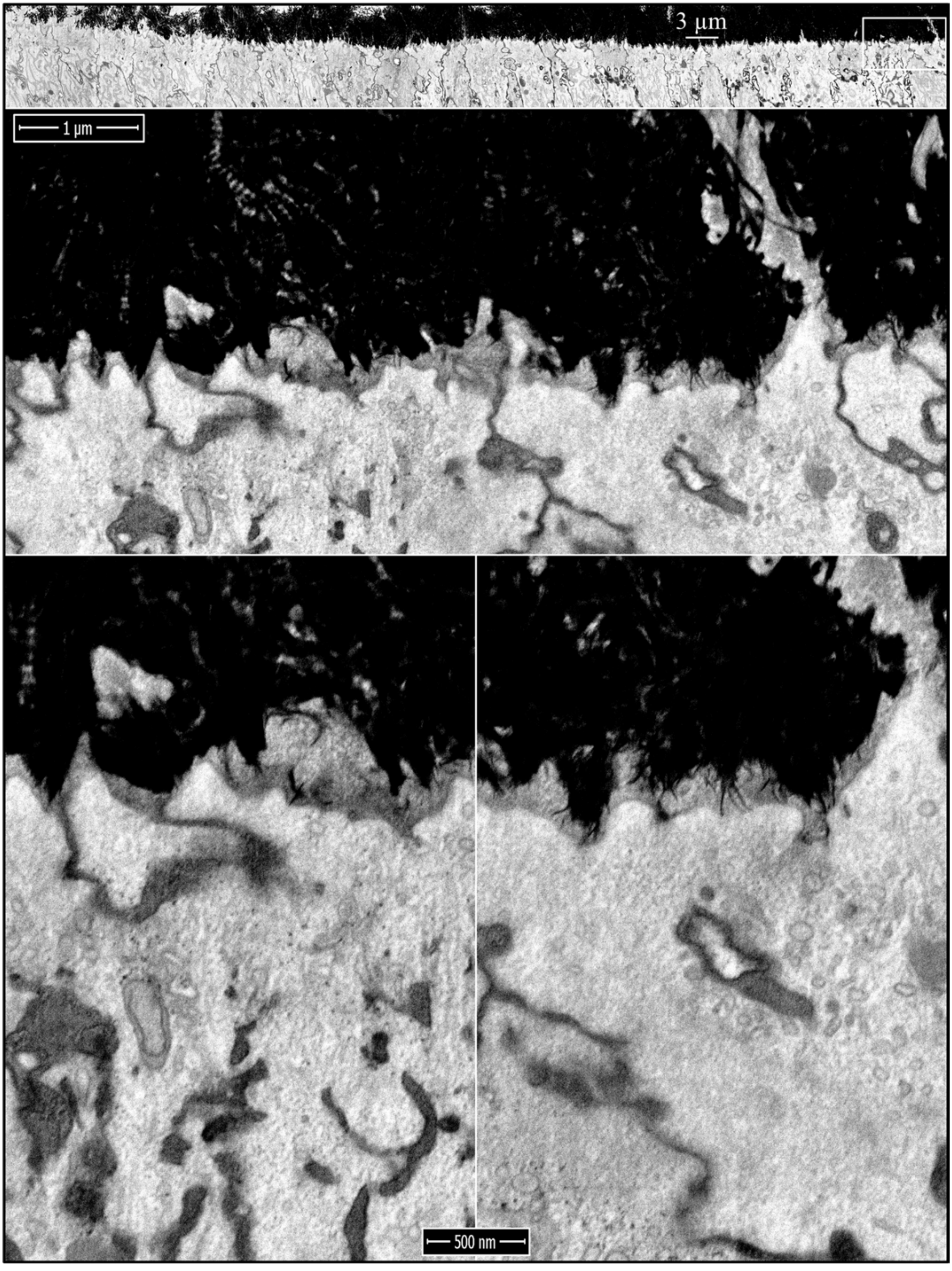

Figure S34 Region 3j. *Ambn*/*Amelx* double null FIB-SEM images. ***Top:*** Montage of 10000x TLD images. ***Middle:*** Montage of 20000x TLD images from boxed area. ***Bottom:*** 35000x TLD images.

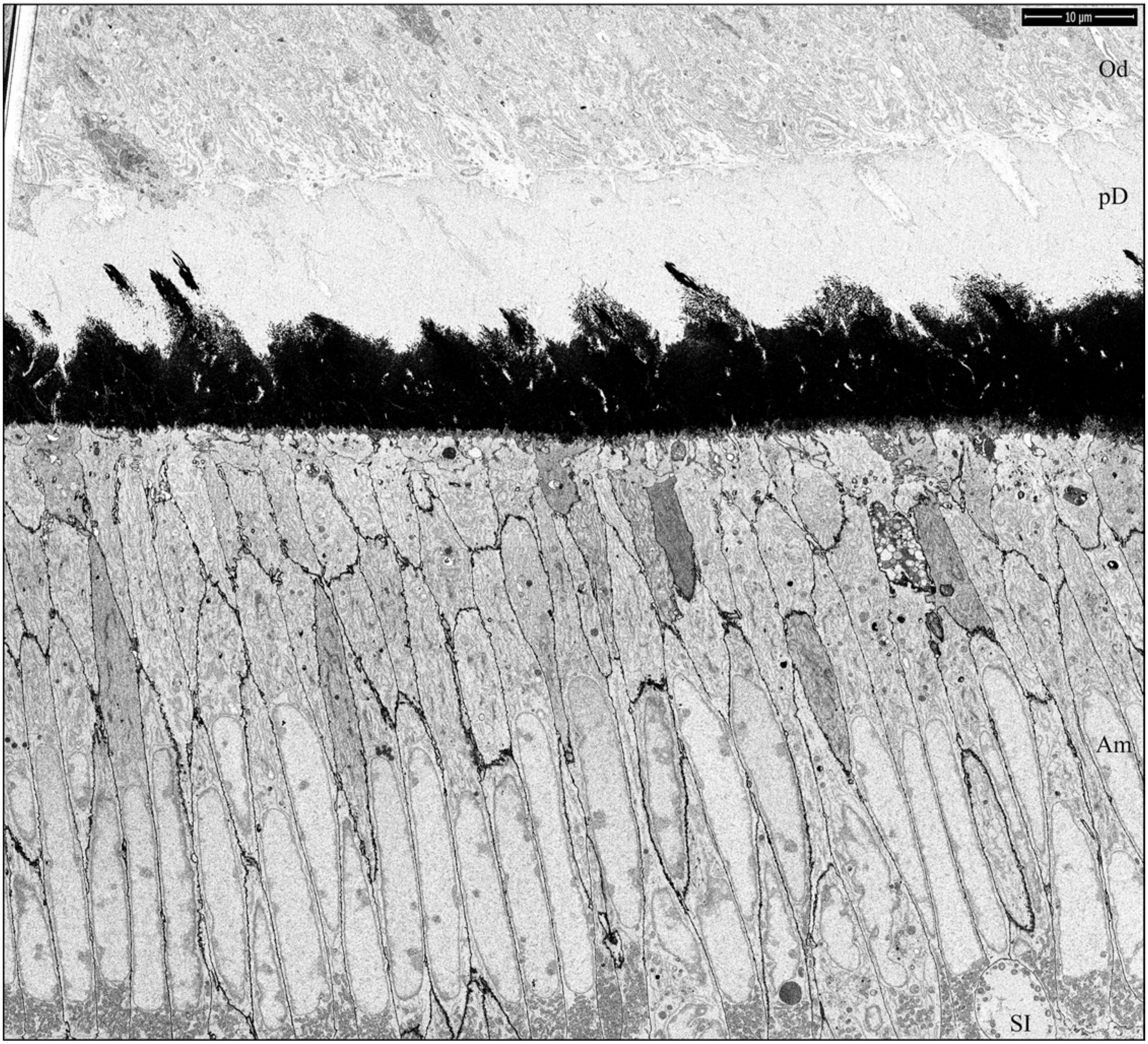

Figure S35 Region 4. Montage of *Ambn*/*Amelx* double null FIB-SEM images (2500x TLD) showing the region immediately incisal to Region 3 (Figs. S24-S34) showing a lack of enamel initiation despite progression of dentin mineralization to high density and 15-20 µm in thickness. Moving incisally, the ameloblasts increasingly show increasing signs of cell pathology. **Key:** Am: Ameloblast; Od: Odontoblast; pD: predentin; SI: stratum intermedium.

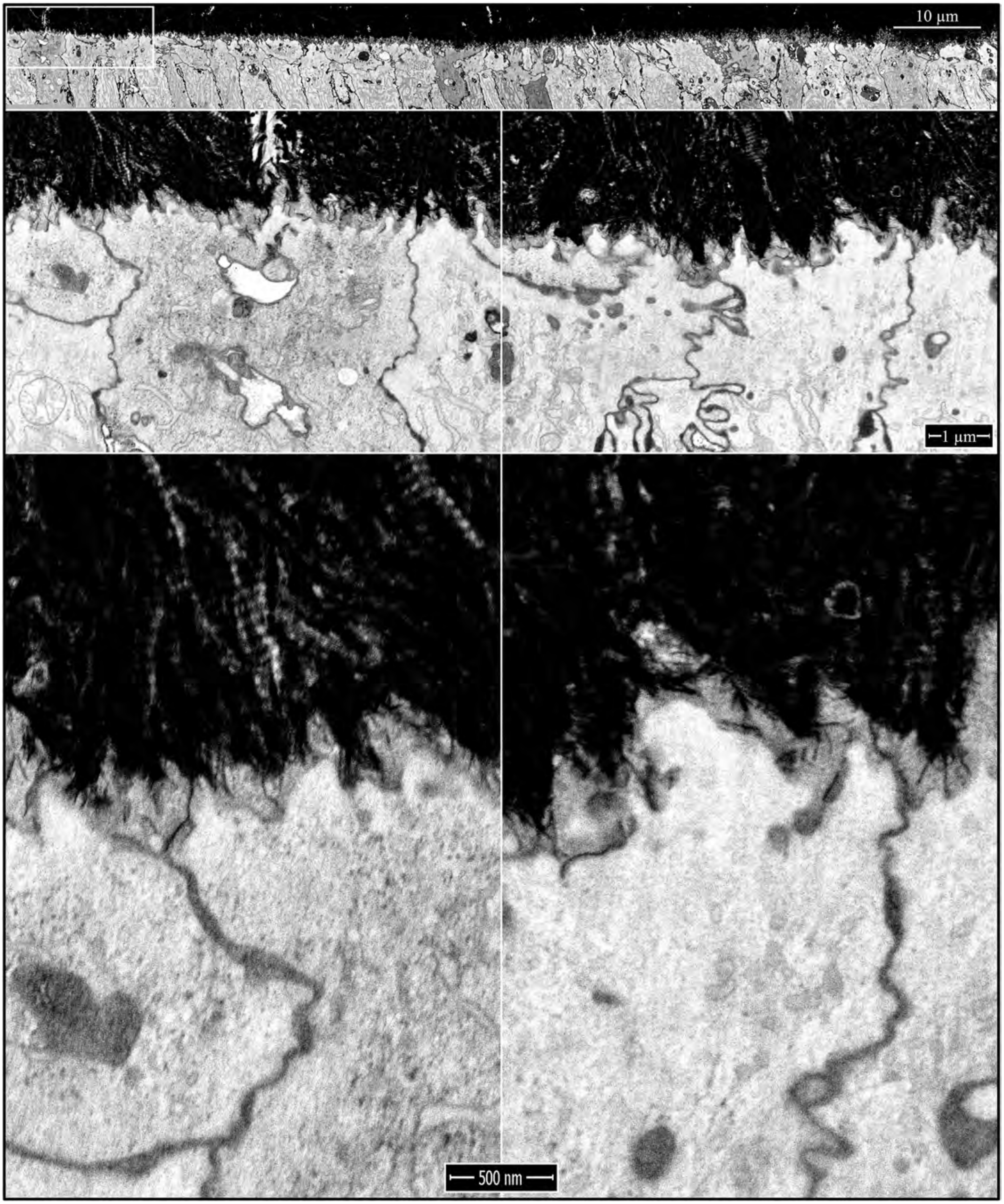

Figure S36 Region 4a. *Ambn*/*Amelx* double null FIB-SEM images. ***Top:*** Montage of 10000x TLD images. ***Middle:*** Montage of 20000x TLD images from boxed area. ***Bottom:*** 35000x TLD images.

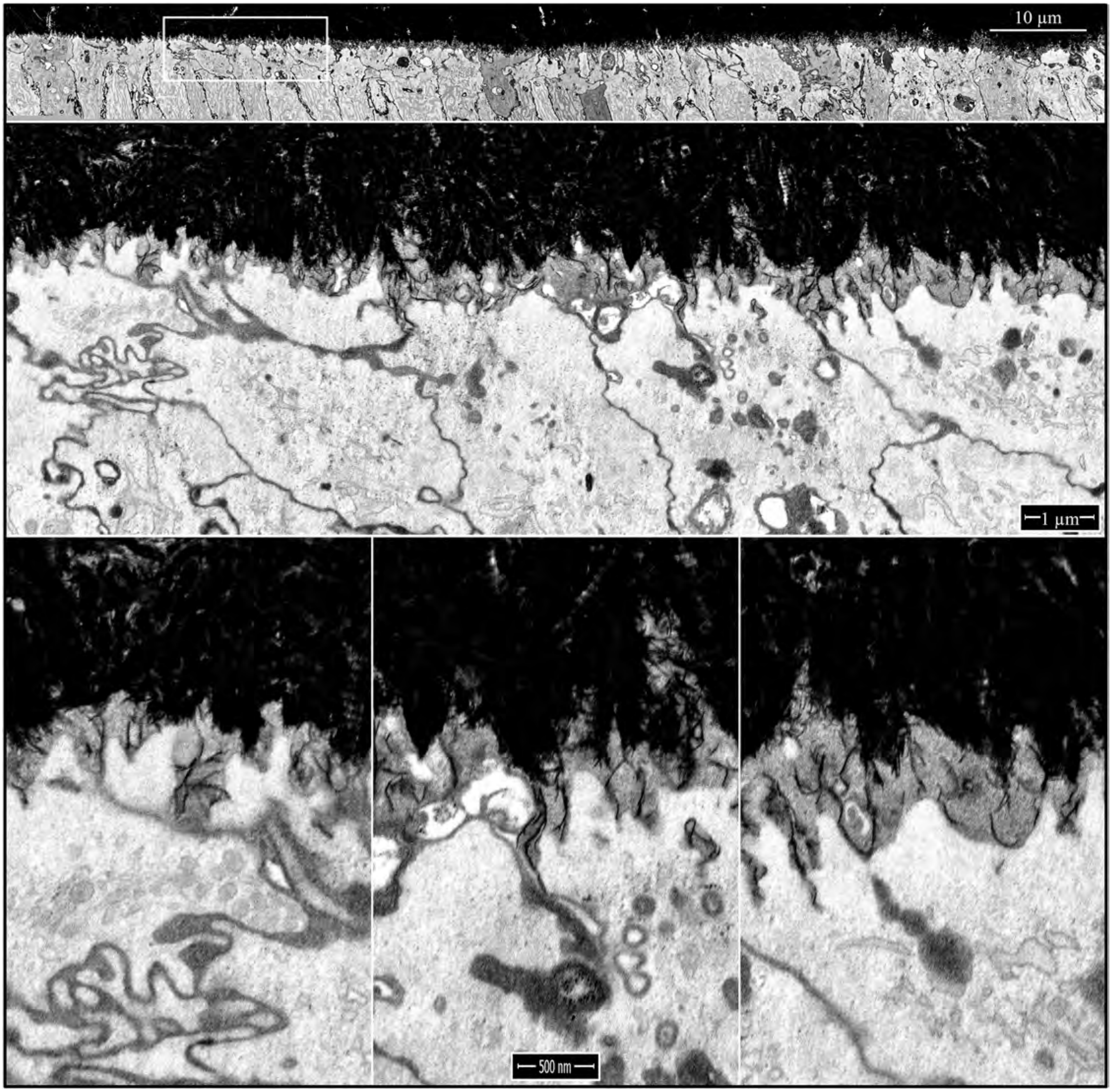

Figure S37 Region 4b. *Ambn*/*Amelx* double null FIB-SEM images. ***Top:*** Montage of 10000x TLD images. ***Middle:*** Montage of 20000x TLD images from boxed area. ***Bottom:*** 35000x TLD images.

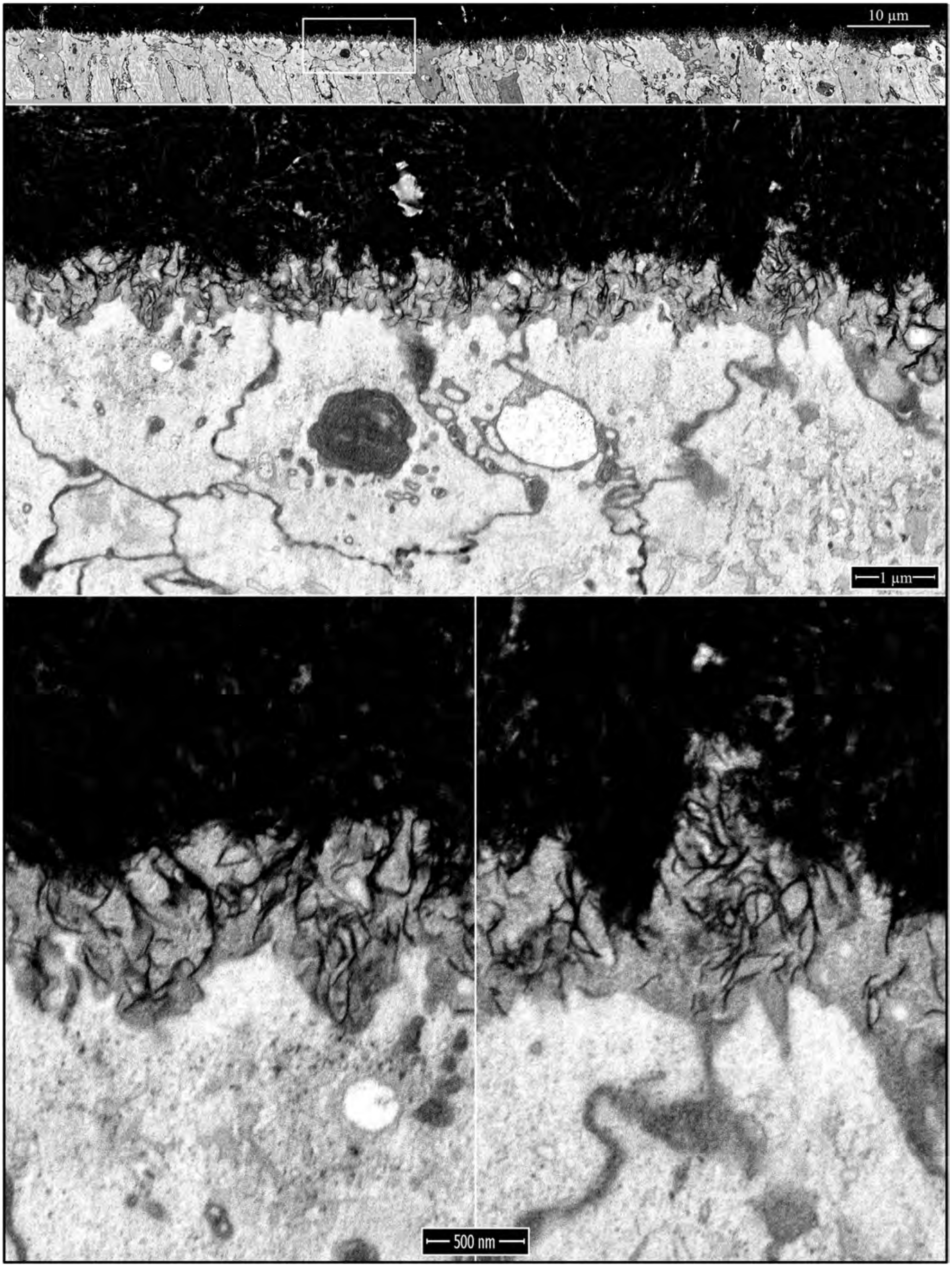

Figure S38 Region 4c. *Ambn*/*Amelx* double null FIB-SEM images. ***Top:*** Montage of 10000x TLD images. ***Middle:*** Montage of 20000x TLD images from boxed area. Note the large dark and light vacuoles within the ameloblasts at the time strange curled ribbon-like deposits form. ***Bottom:*** 35000x TLD images.

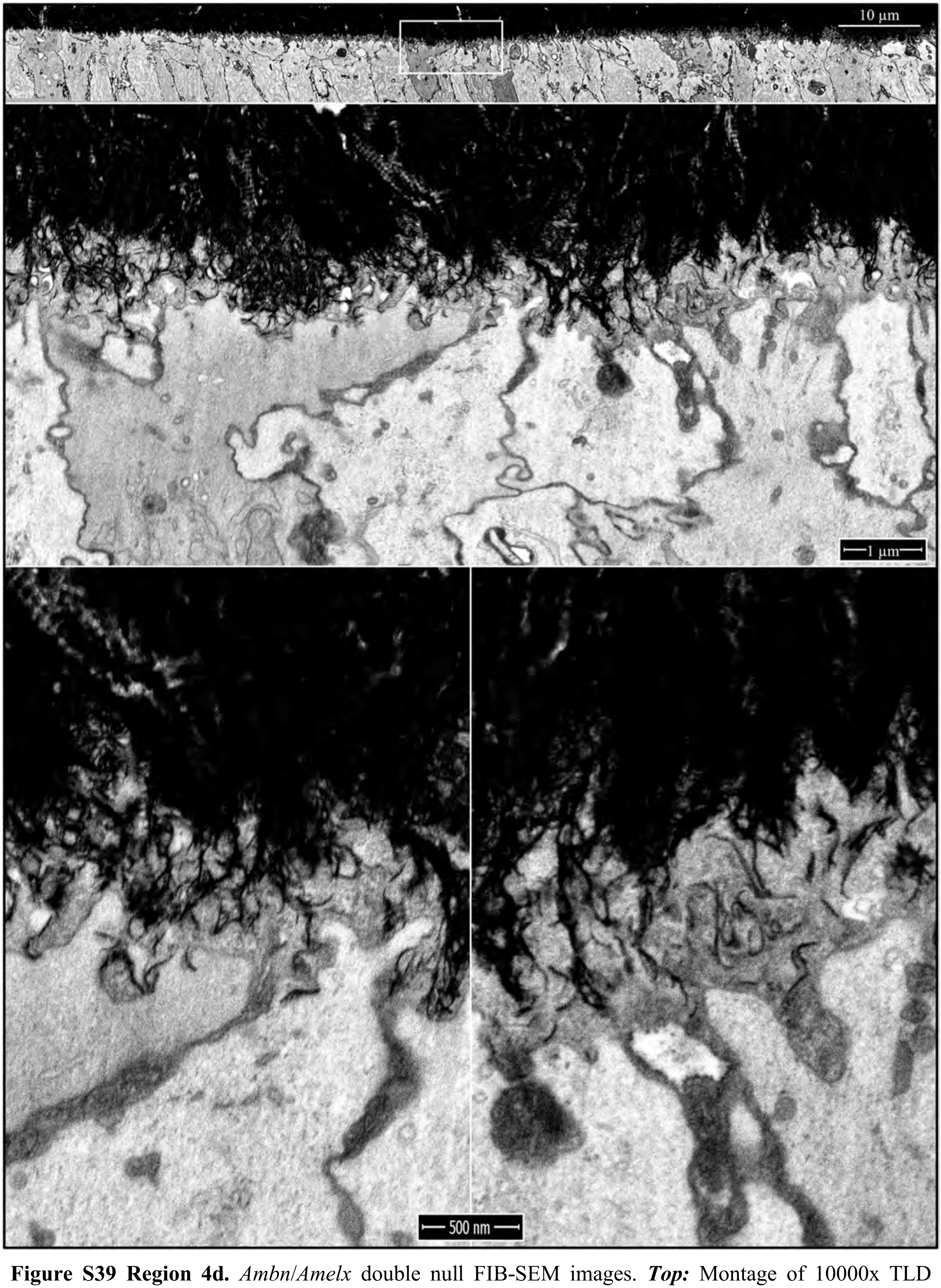

Figure S39 Region 4d. *Ambn/Amelx* double null FIB-SEM images. ***Top:*** Montage of 10000x TLD images. ***Middle:*** Montage of 20000x TLD images from boxed area. ***Bottom:*** 35000x TLD images.

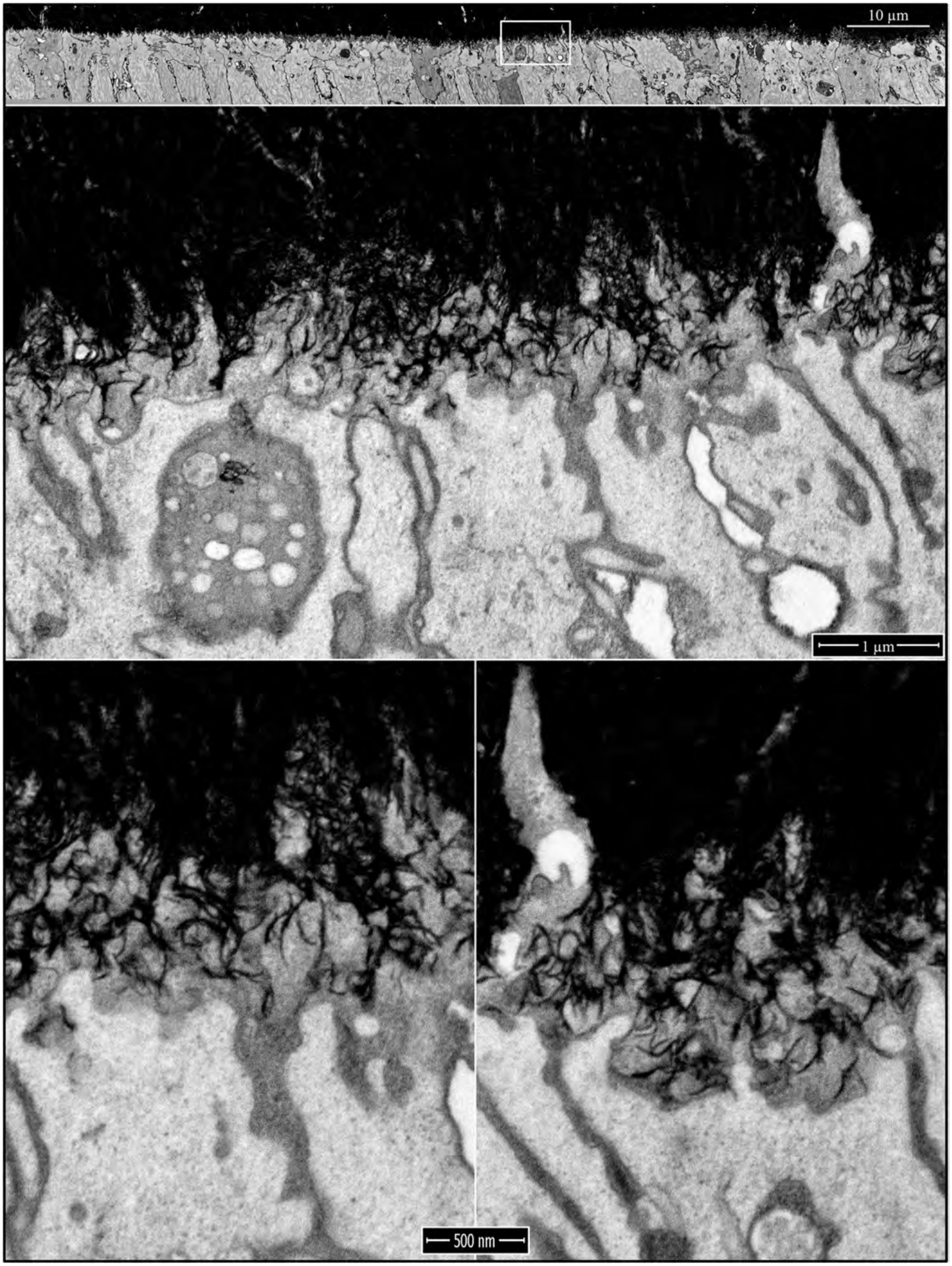

Figure S40 Region 4e. *Ambn*/*Amelx* double null FIB-SEM images. ***Top:*** Montage of 10000x TLD images. ***Middle:*** Montage of 20000x TLD images from boxed area. Note the large vacuole containing smaller vacuoles or vesicles. ***Bottom:*** 35000x TLD images.

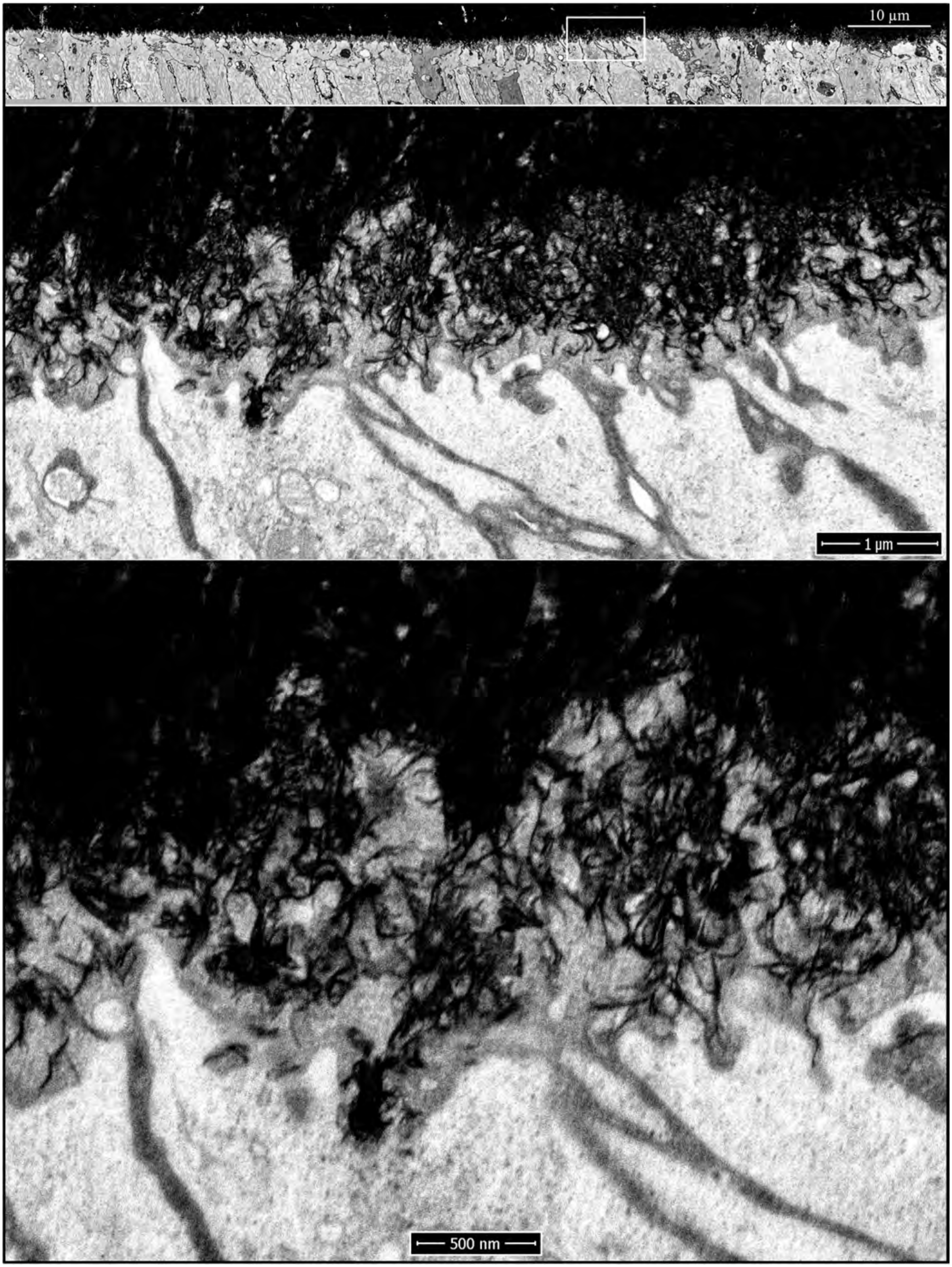

Figure S41 Region 4f. *Ambn/Amelx* double null FIB-SEM images. ***Top:*** Montage of 10000x TLD images. ***Middle:*** Montage of 20000x TLD images from boxed area. ***Bottom:*** 35000x TLD images. Disorganized, mineral crust, not oriented toward the ameloblast membrane forms on dentin.

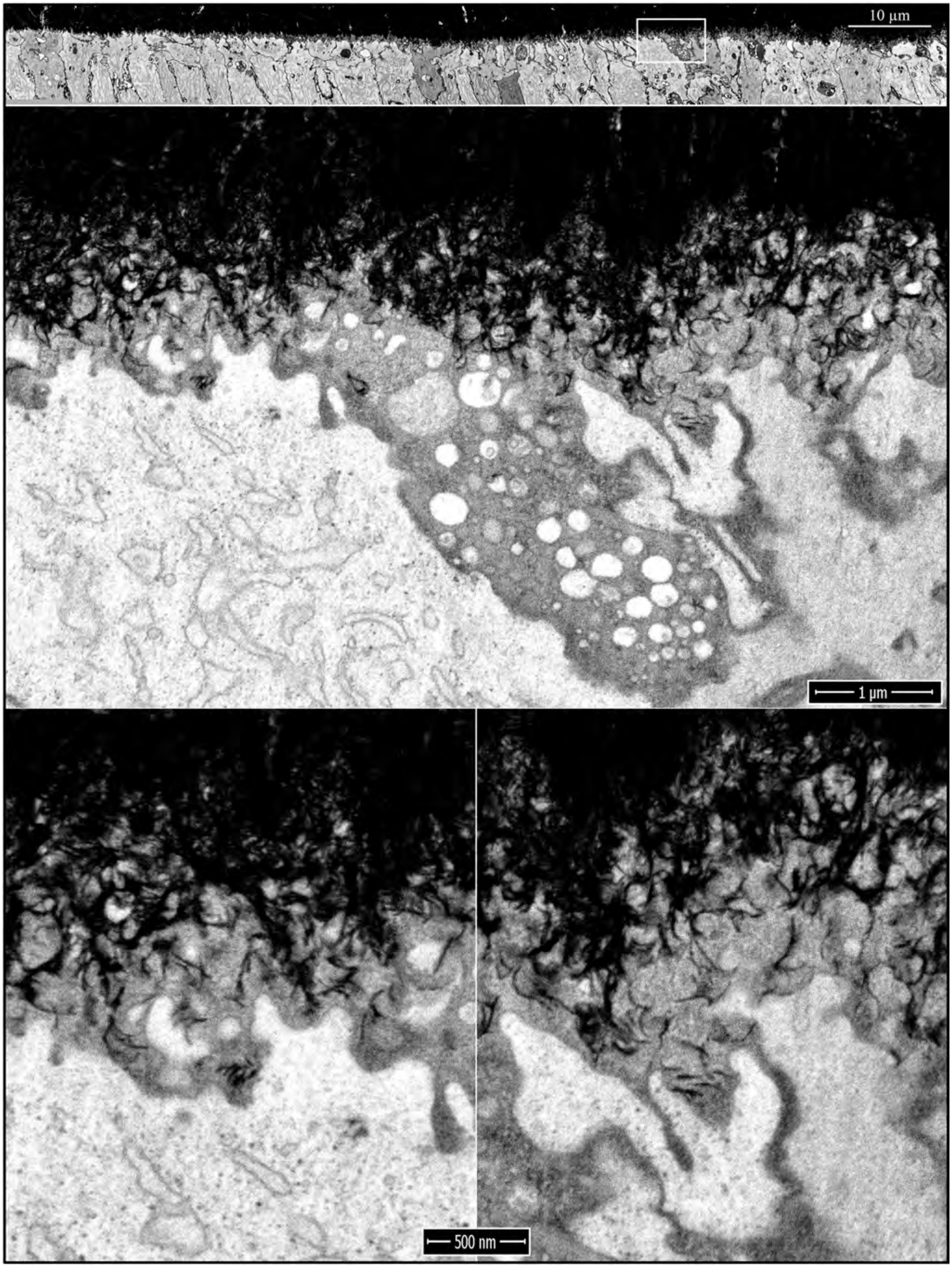

Figure S42 Region 4g. *Ambn*/*Amelx* double null FIB-SEM images. ***Top:*** Montage of 10000x TLD images. ***Middle:*** Montage of 20000x TLD images from boxed area showing ameloblast cell pathology associated with the deposition of a mineral crust on the dentin surface. ***Bottom:*** 35000x TLD images showing the initial mineral crust that is deposited in place of a characteristic field of initial enamel.

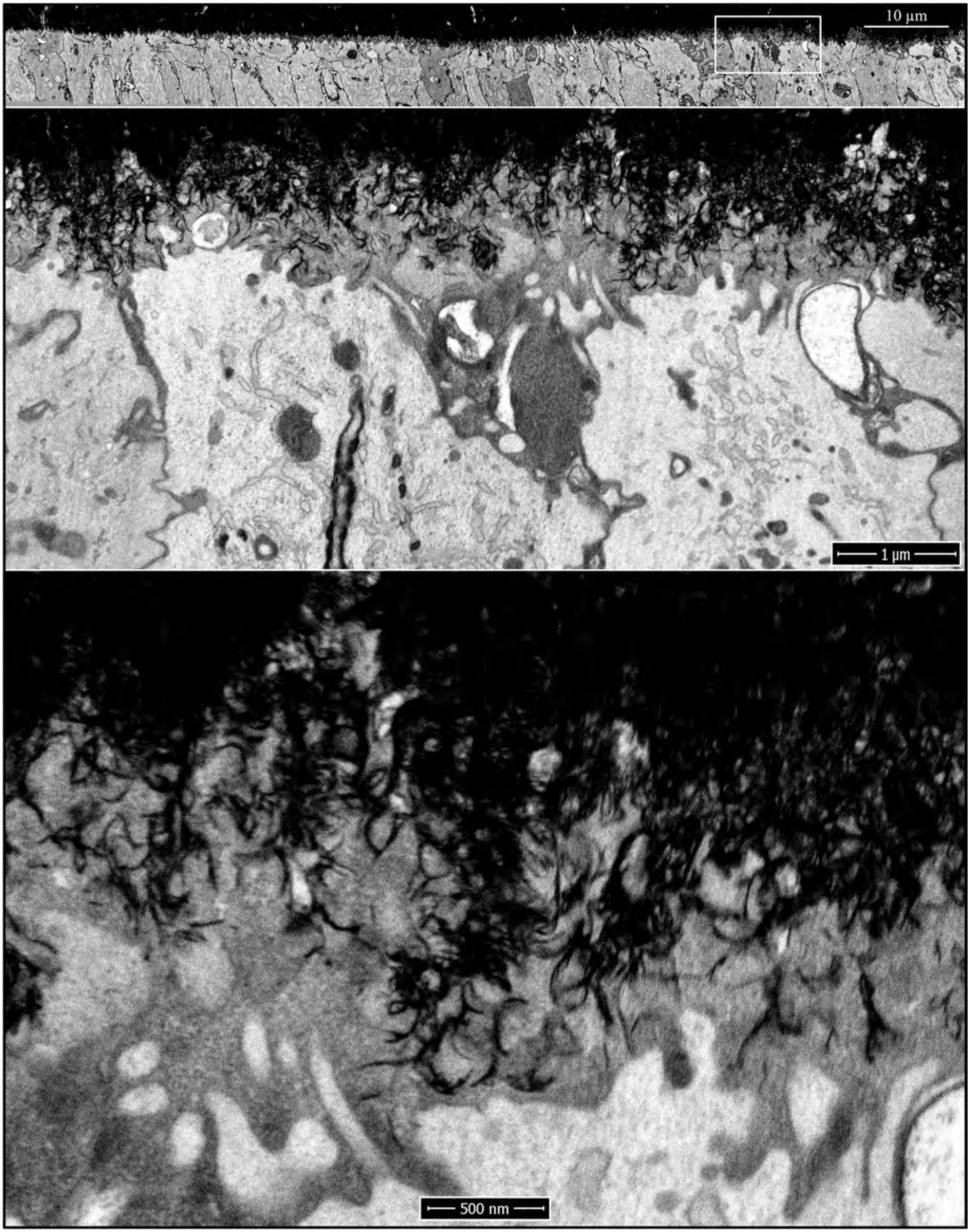

Figure S43 Region 4h. *Ambn*/*Amelx* double null FIB-SEM images. ***Top:*** Montage of 10000x TLD images. ***Middle:*** Montage of 20000x TLD images from boxed area. ***Bottom:*** 35000x TLD images.

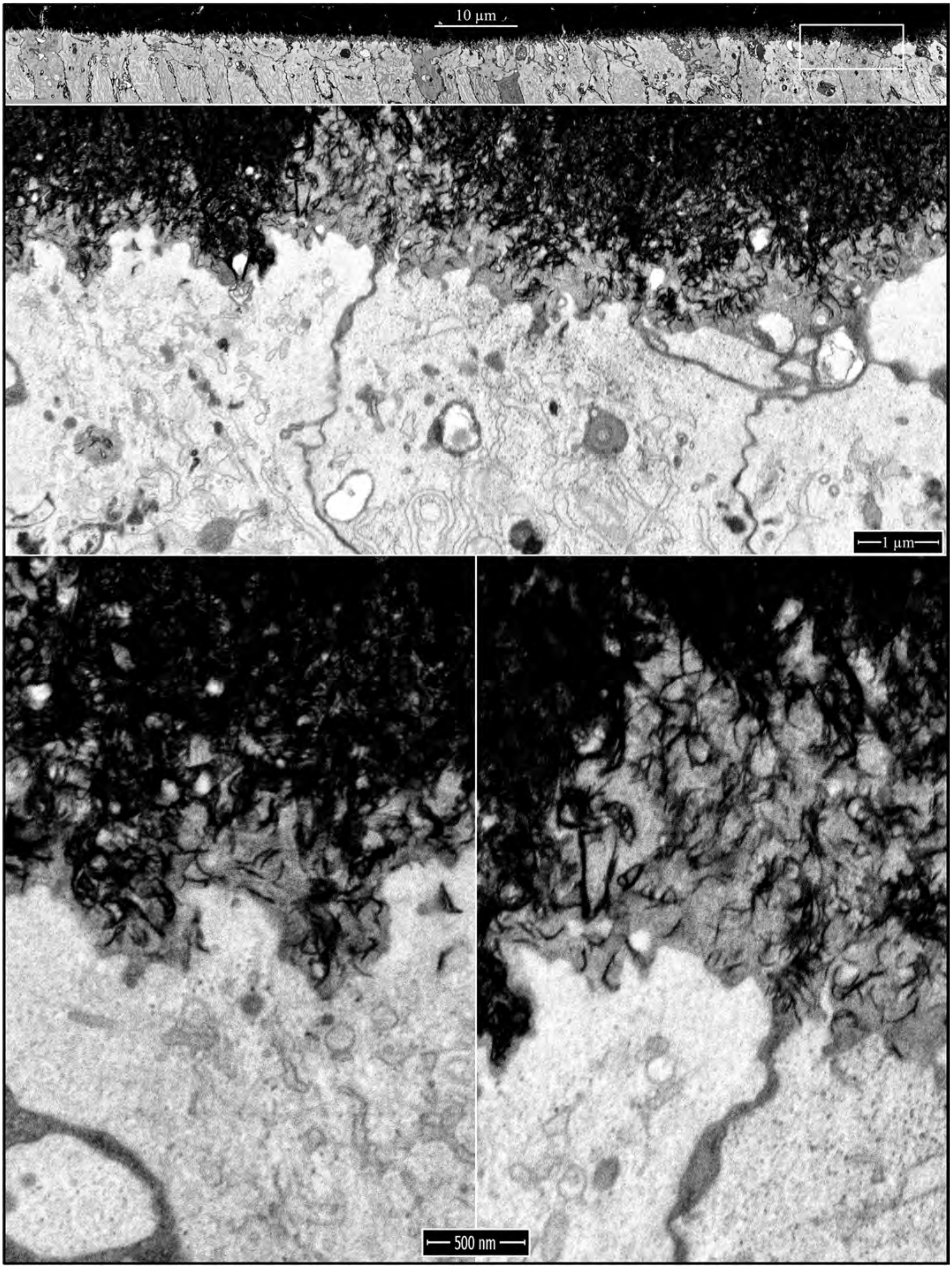

Figure S44 Region 4i. *Ambn*/*Amelx* double null FIB-SEM images. ***Top:*** Montage of 10000x TLD images. ***Middle:*** Montage of 20000x TLD images from boxed area. ***Bottom:*** 35000x TLD images.
