# Supplementary material for: AMBN mutations causing hypoplastic amelogenesis imperfecta and Ambn knockout‐NLS‐lacZ knockin mice exhibiting failed amelogenesis and Ambn tissue‐specificity
Source: Mol Genet Genomic Med. 2019 Aug 11;7(9):e929. doi: 10.1002/mgg3.929 (PMC6732285; doi:10.1002/mgg3.929)
Supplement: Supplementary file 4 [file MGG3-7-e929-s004.pdf]

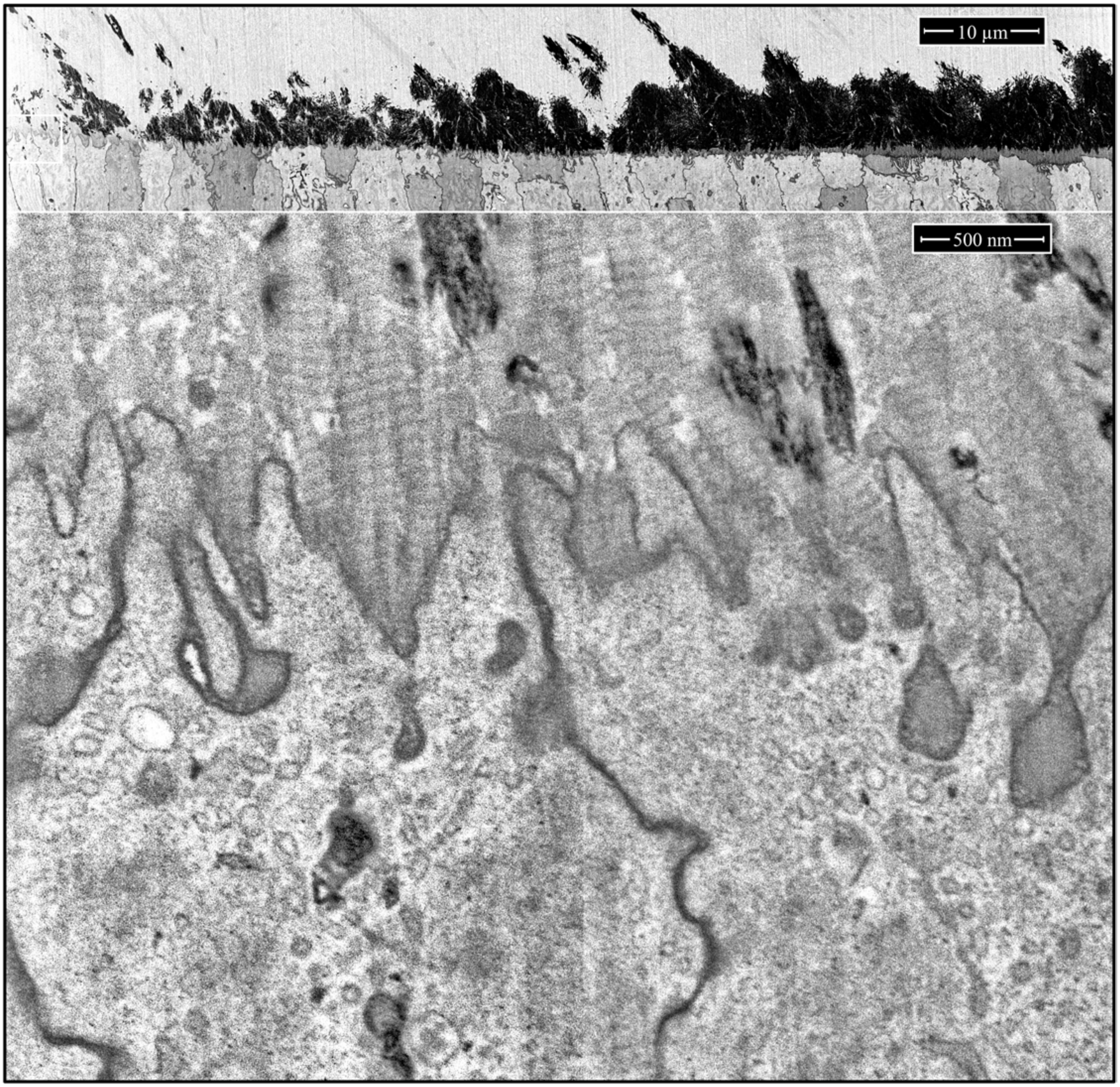

Figure S45. 7-week wild-type mandibular incisor FIB-SEM ~1 mm from its basal end (box 1). ***Top:*** Montage of 10000x TLD images of the ameloblast-extracellular matrix interface starting immediately after the onset of dentin mineralization (left), and progressing through the expansion of dentin mineral into a continuous layer, the onset of enamel formation and deposition of the initial enamel (right). ***Bottom:*** Montage of 35000x TLD images of the white box outlined above. The basement membrane has already been degraded and reabsorbed by the ameloblast, which extends finger-like projections into the underlying predentin. Banded collagen fibers butt into ameloblasts at nearly right angles. Islands of mineral appear in predentin nearer to the ameloblast then the odontoblast and expand up the collagen fibers.

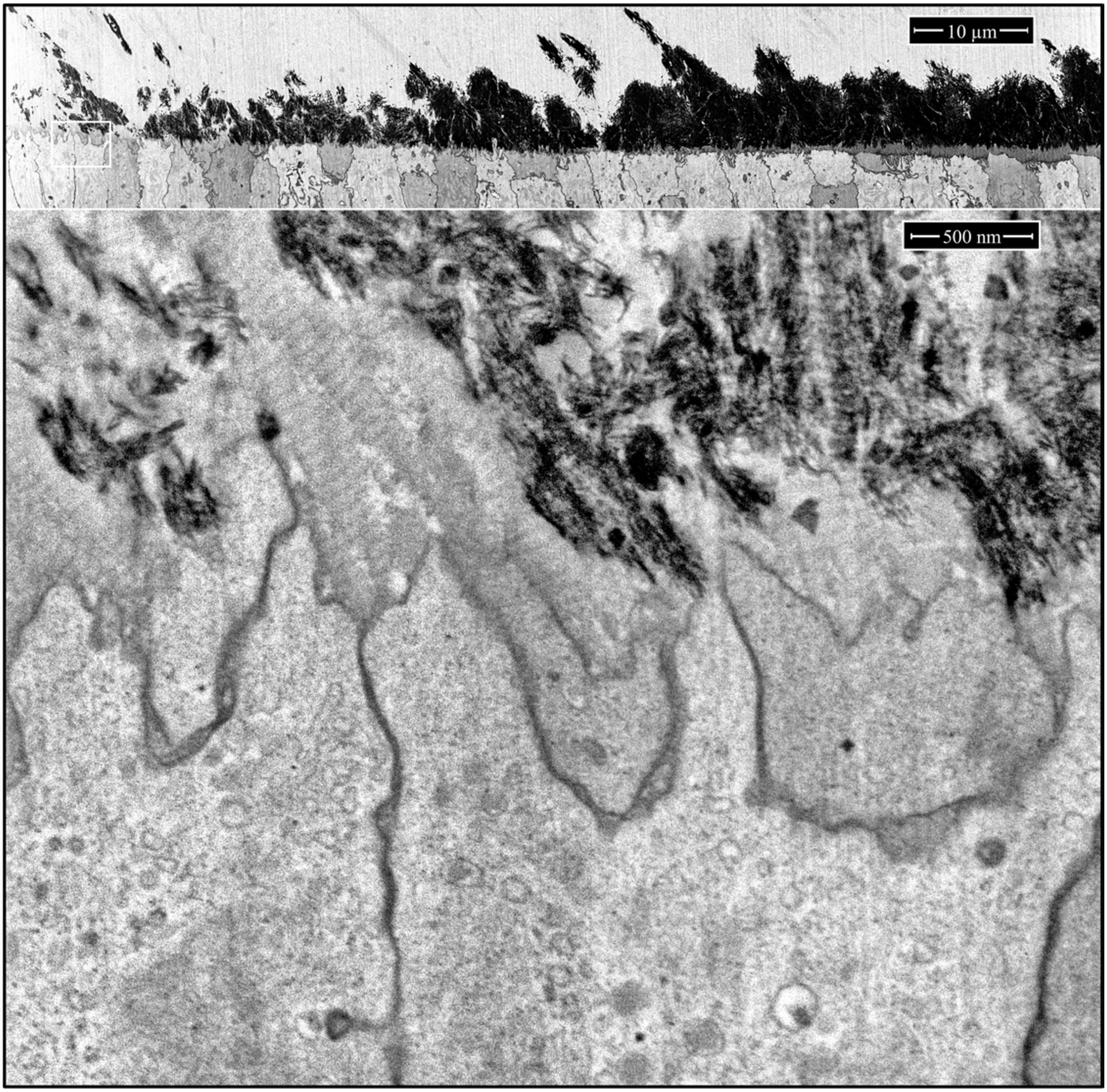

Figure S46. 7-week wild-type mandibular incisor FIB-SEM ~1 mm from its basal end (box 2). ***Top:*** Montage of 10000x TLD images of the ameloblast-extracellular matrix interface starting immediately after the onset of dentin mineralization (left), and progressing through the expansion of dentin mineral into a continuous layer, the onset of enamel formation and deposition of the initial enamel (right). ***Bottom:*** Montage of 35000x TLD images of the white box outlined above. New islands of dentin mineral appear along clusters of collagen fibers deep into the predentin matrix near the odontoblast. The dentin mineral coalesces into a continuous mineral layer and expands toward the ameloblasts on one side and the odontoblasts on the other.

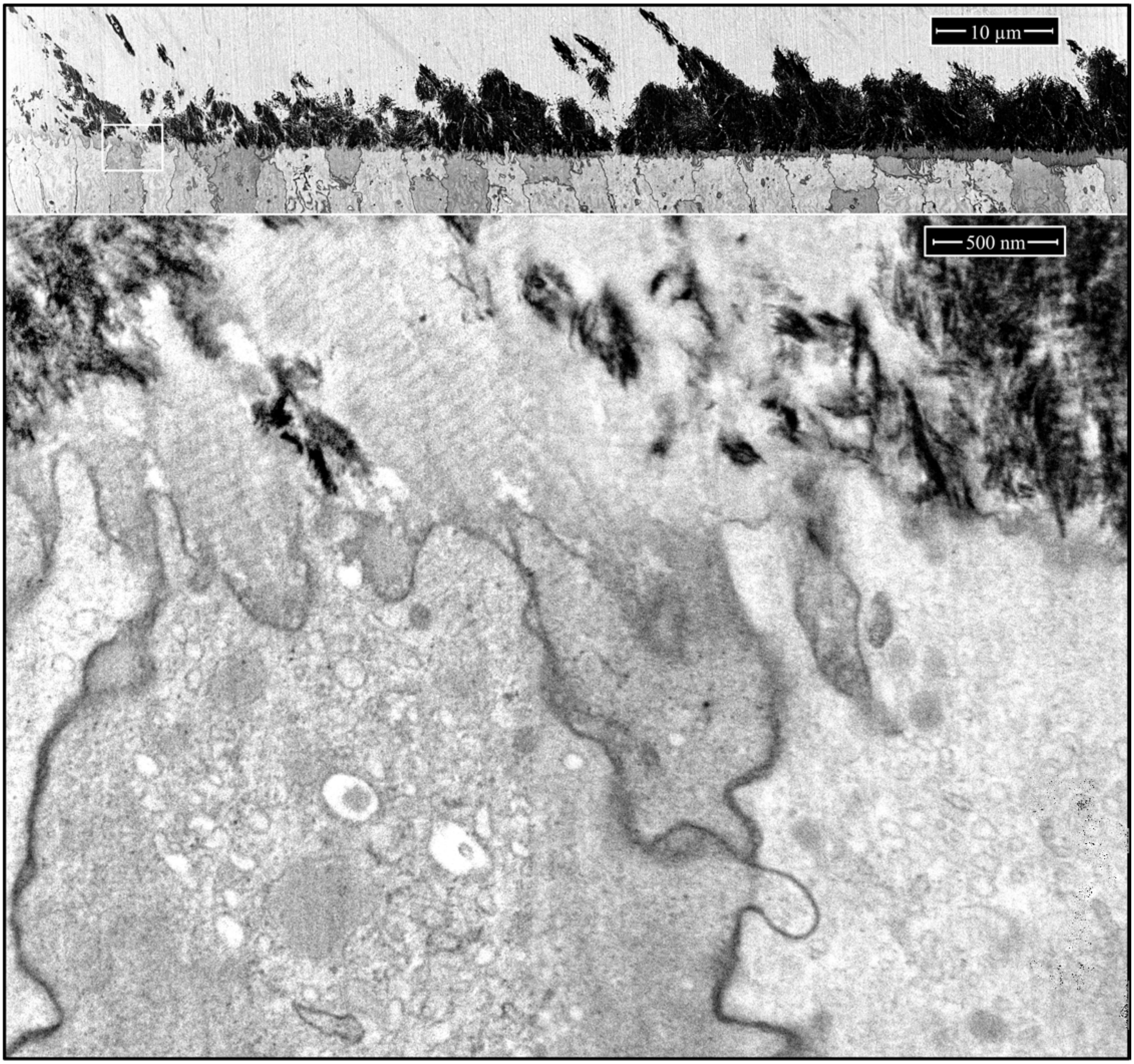

Figure S47. 7-week wild-type mandibular incisor FIB-SEM ~1 mm from its basal end (box 3). ***Top:*** Montage of 10000x TLD images of the ameloblast-extracellular matrix interface illustrating early dentin and enamel formation. ***Bottom:*** Montage of 35000x TLD images of the white box outlined above. As the dentin mineral expands toward the ameloblast patches of predentin are sometimes observed near the ameloblast.

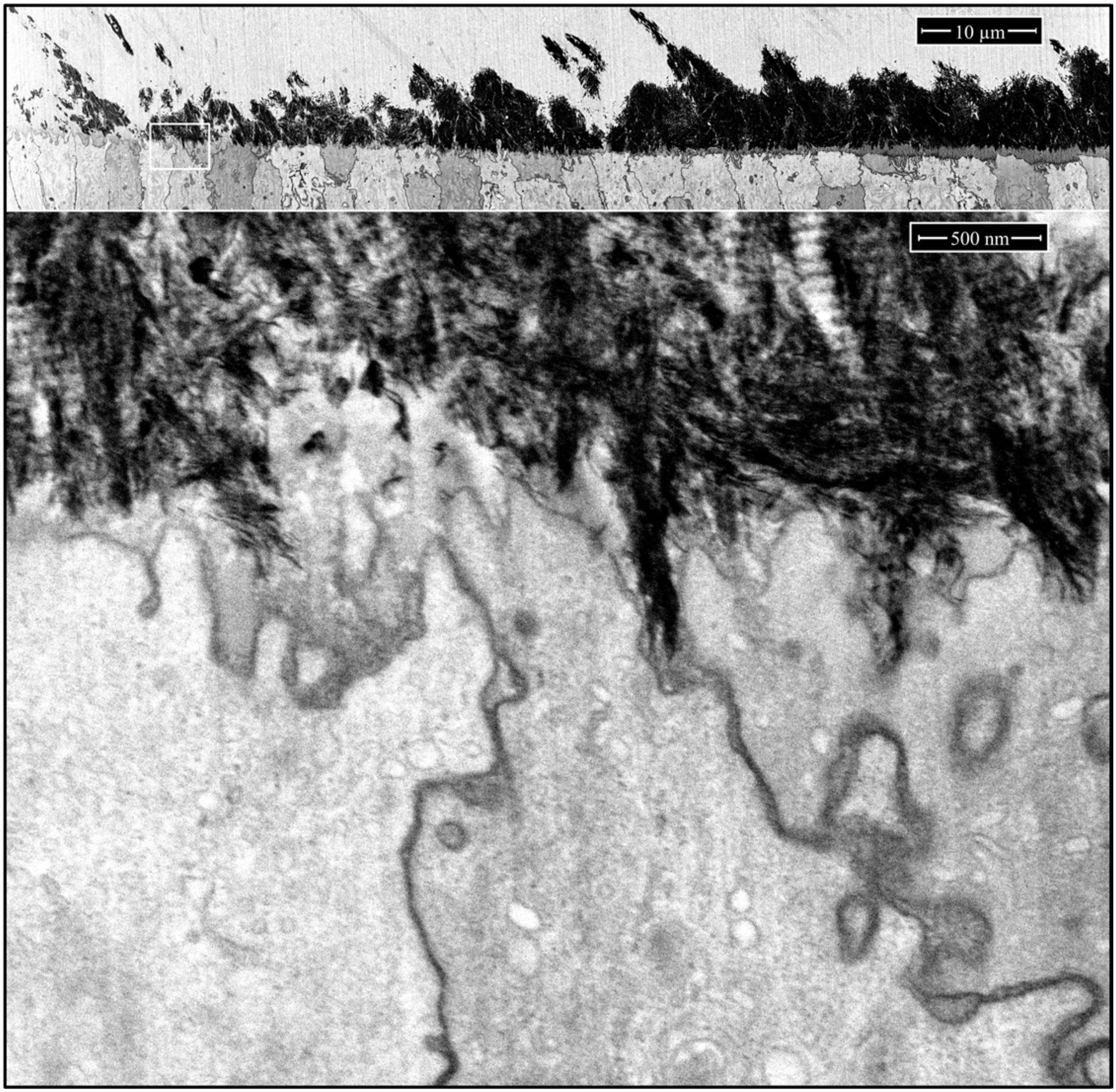

Figure S48. 7-week wild-type mandibular incisor FIB-SEM ~1 mm from its basal end (box 4). ***Top:*** Montage of 10000x TLD images of the ameloblast-extracellular matrix interface illustrating early dentin and enamel formation. ***Bottom:*** Montage of 35000x TLD images of the white box outlined above. As the dentin mineral expands toward the ameloblast patches of predentin are sometimes observed near the ameloblast. In most places the dentin mineral expands up the collagen fibers in close proximity to the ameloblast distal membrane.

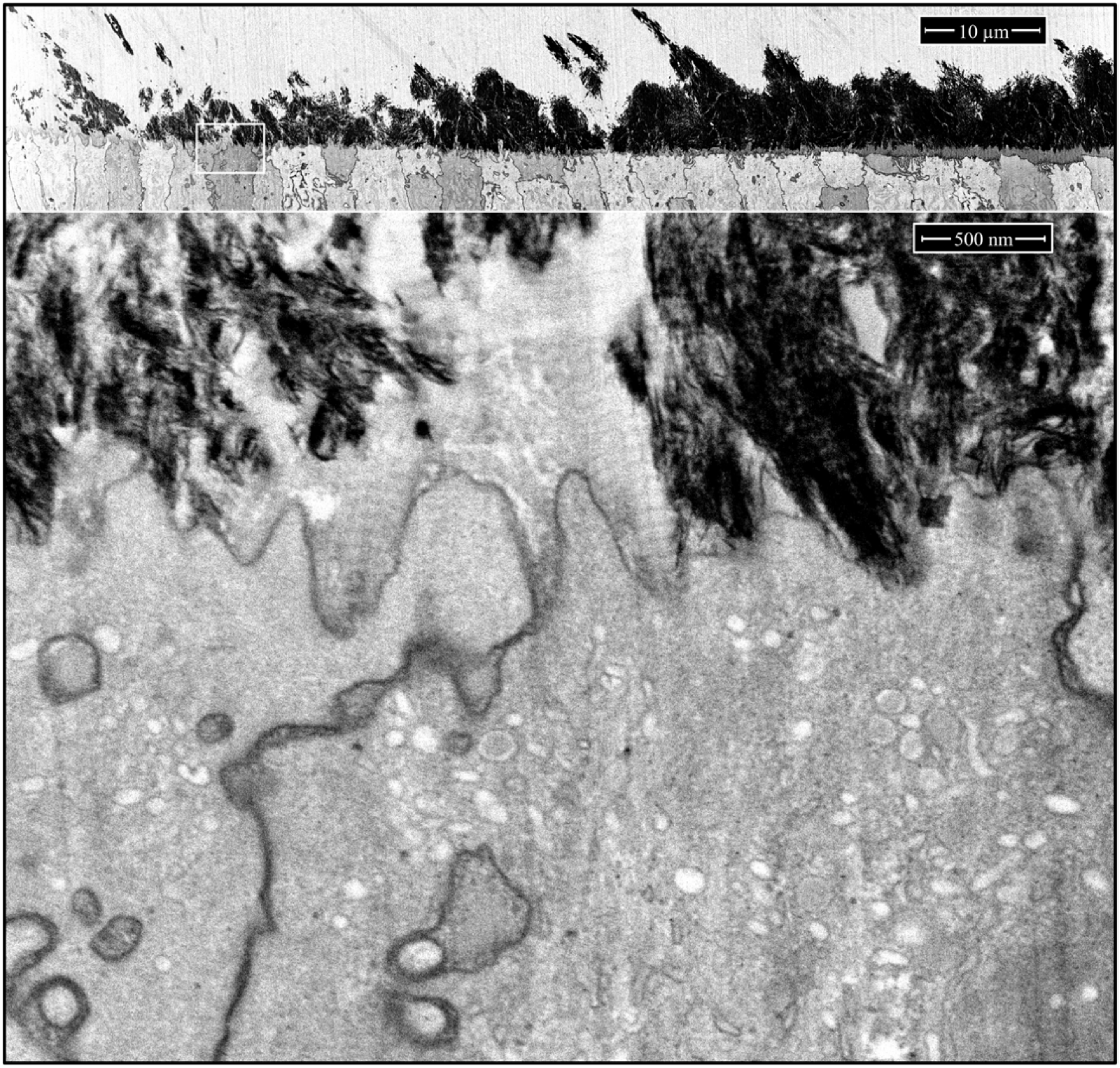

Figure S49. 7-week wild-type mandibular incisor FIB-SEM ~1 mm from its basal end (box 5). ***Top:*** Montage of 10000x TLD images of the ameloblast-extracellular matrix interface illustrating early dentin and enamel formation. ***Bottom:*** Montage of 35000x TLD images of the white box outlined above. As the dentin mineral expands toward the ameloblast patches of predentin are sometimes observed near the ameloblast. In most places the dentin mineral expands up the collagen fibers in close proximity to the ameloblast distal membrane.

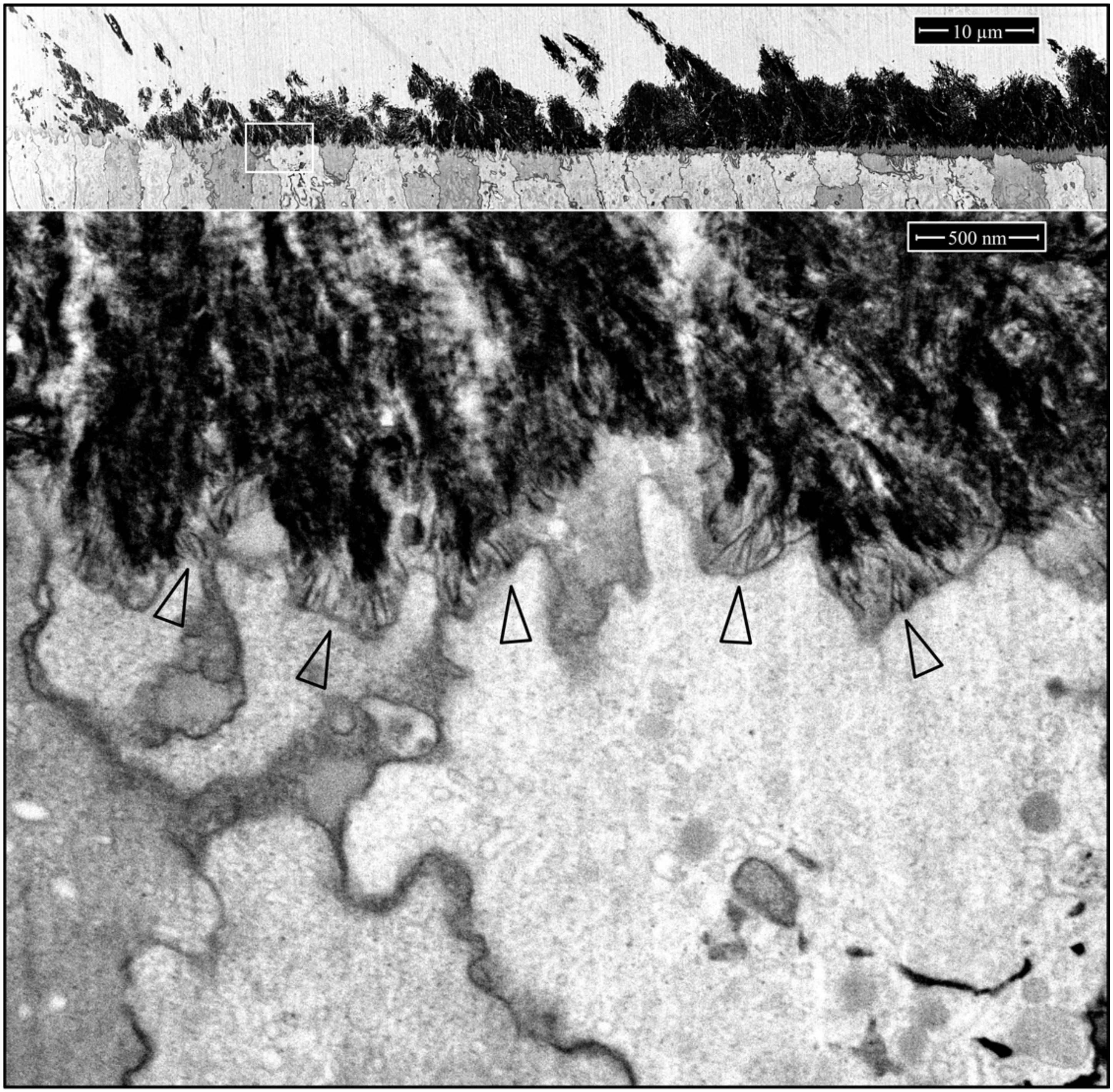

Figure S50. 7-week wild-type mandibular incisor FIB-SEM ~1 mm from its basal end (box 6). ***Top:*** Montage of 10000x TLD images of the ameloblast-extracellular matrix interface illustrating early dentin and enamel formation. ***Bottom:*** Montage of 35000x TLD images of the white box outlined above. The first enamel ribbons are observed to extend from the collagen-associated dentin mineral to the ameloblast membrane.

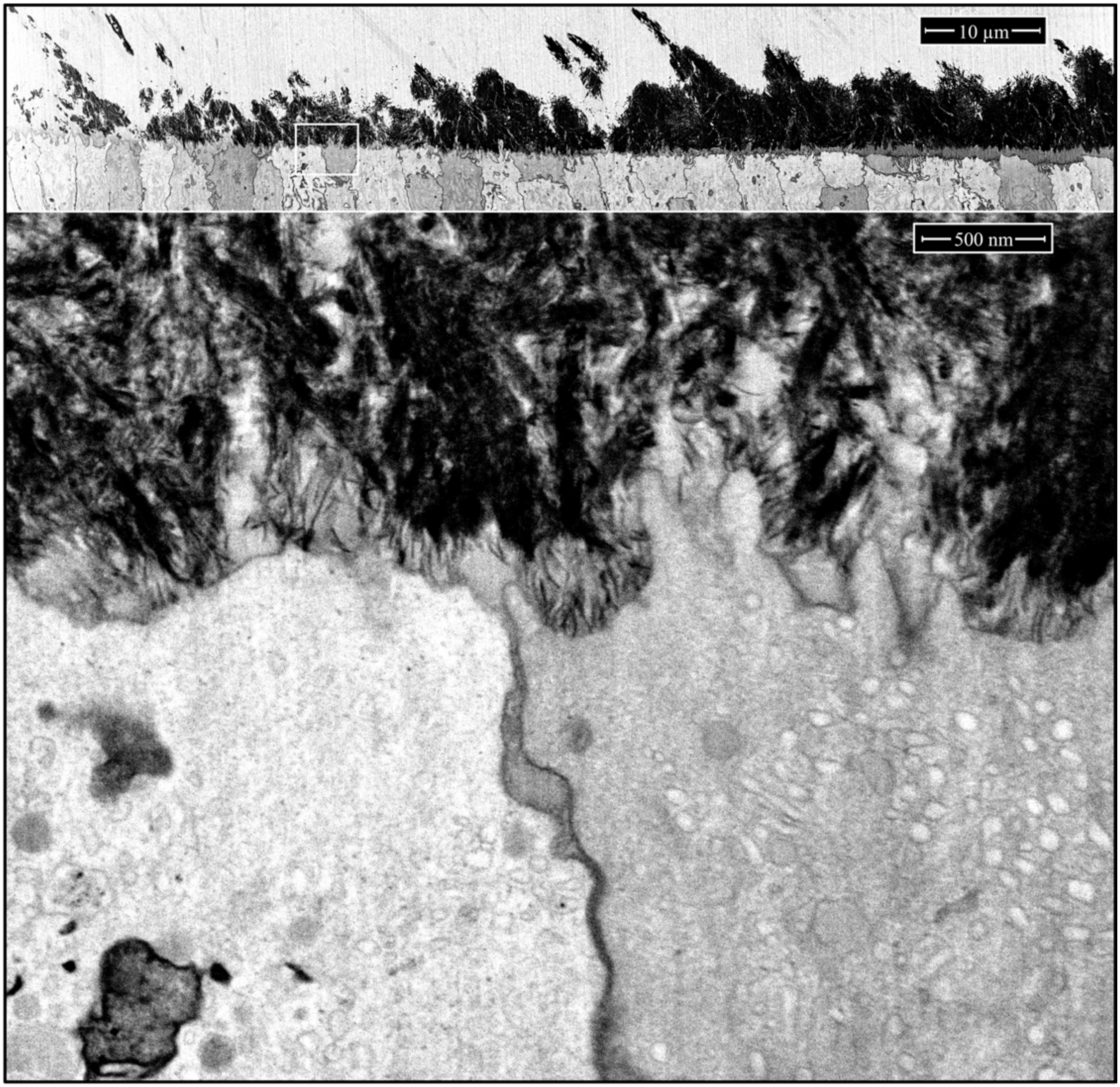

Figure S51. 7-week wild-type mandibular incisor FIB-SEM ~1 mm from its basal end (box 7). ***Top:*** Montage of 10000x TLD images of the ameloblast-extracellular matrix interface illustrating early dentin and enamel formation. ***Bottom:*** Montage of 35000x TLD images of the white box outlined above. The first enamel ribbons are observed to extend from the collagen-associated dentin mineral to the ameloblast membrane.

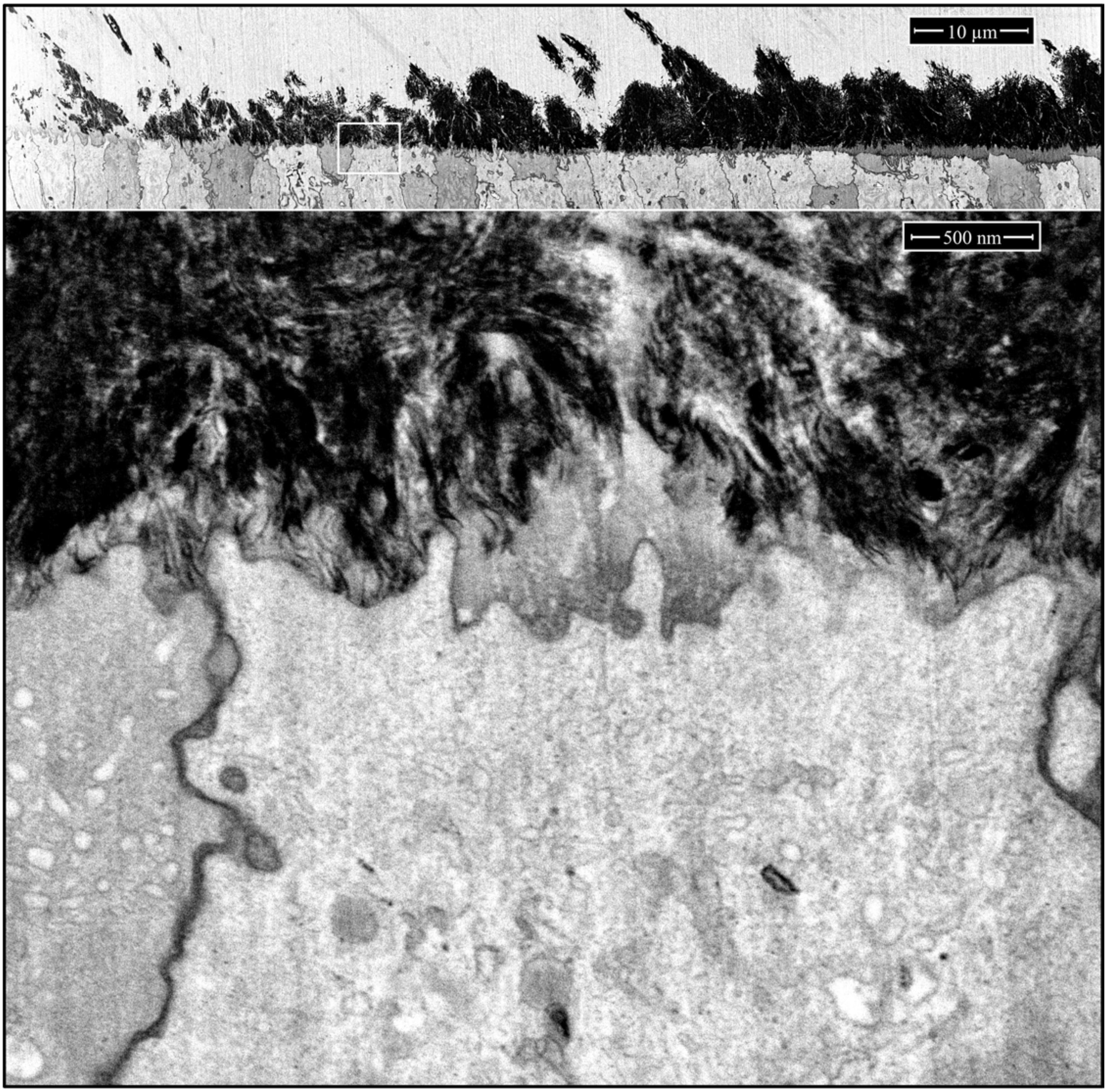

Figure S52. 7-week wild-type mandibular incisor FIB-SEM ~1 mm from its basal end (box 8). ***Top:*** Montage of 10000x TLD images of the ameloblast-extracellular matrix interface illustrating early dentin and enamel formation. ***Bottom:*** Montage of 35000x TLD images of the white box outlined above. The first enamel ribbons are observed to extend from the collagen-associated dentin mineral to the ameloblast membrane.

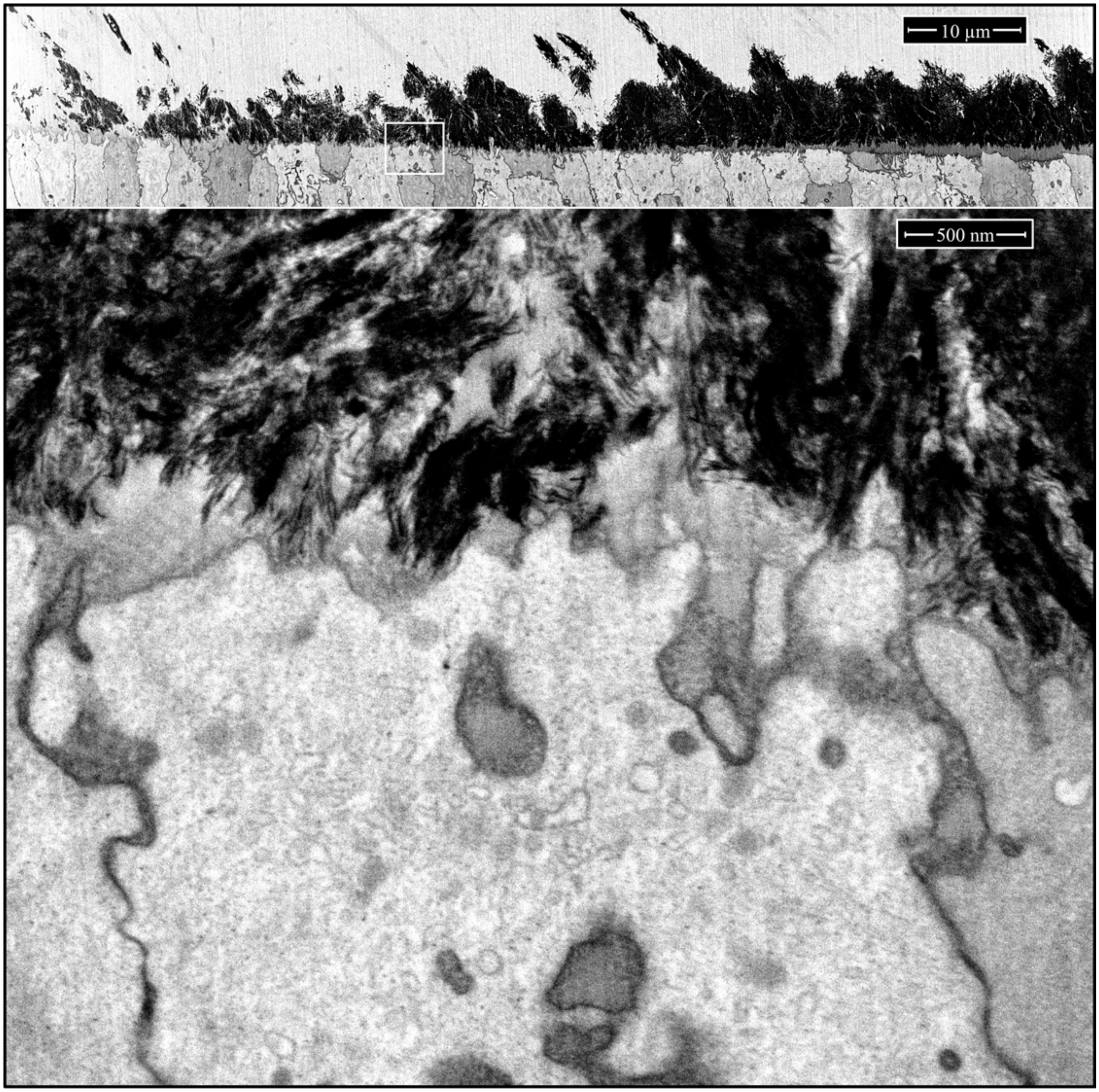

Figure S53. 7-week wild-type mandibular incisor FIB-SEM ~1 mm from its basal end (box 9). ***Top:*** Montage of 10000x TLD images of the ameloblast-extracellular matrix interface illustrating early dentin and enamel formation. ***Bottom:*** Montage of 35000x TLD images of the white box outlined above. The first enamel ribbons are observed to extend from the collagen-associated dentin mineral to the ameloblast membrane.

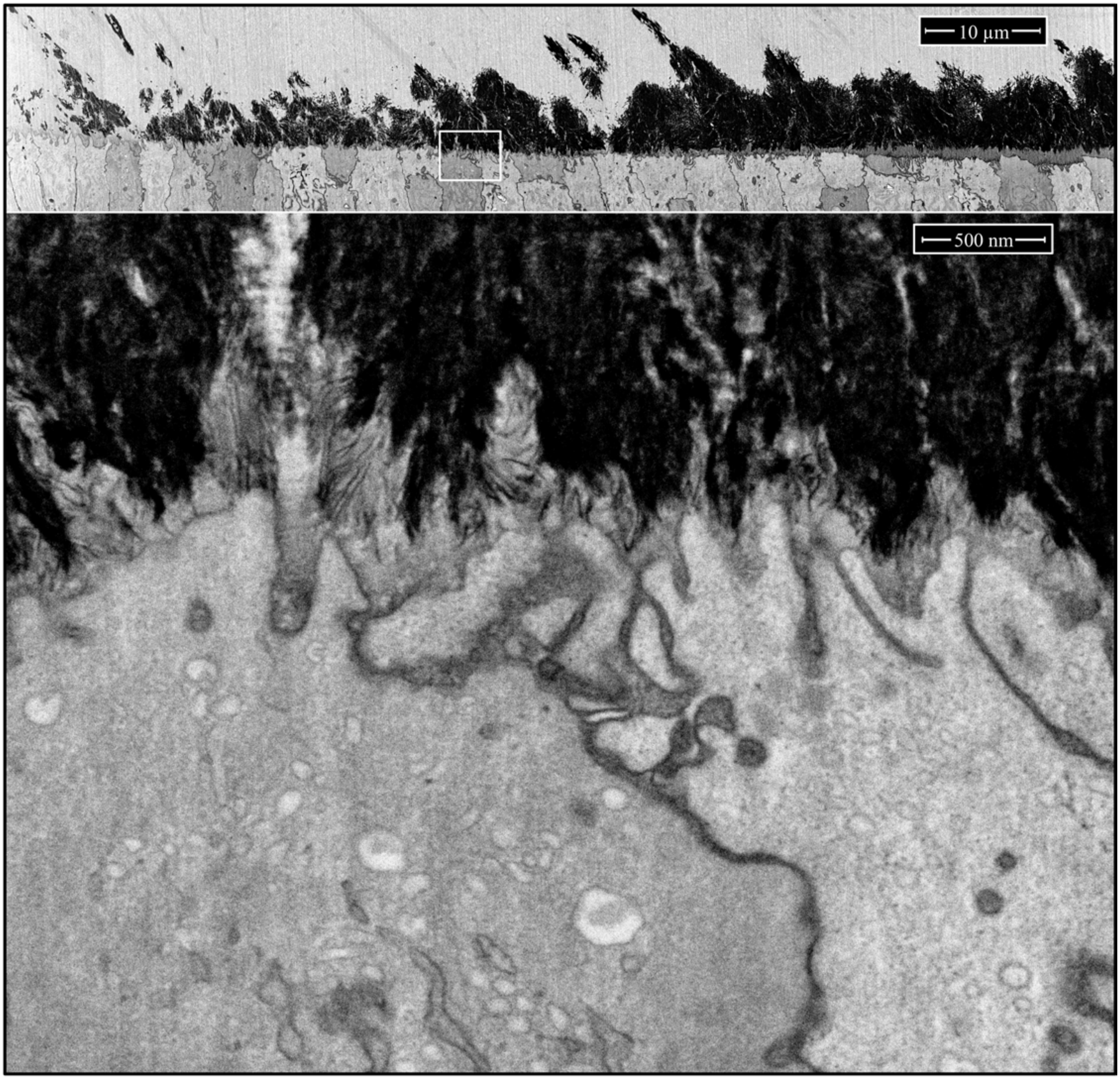

Figure S54. 7-week wild-type mandibular incisor FIB-SEM ~1 mm from its basal end (box 10). ***Top:*** Montage of 10000x TLD images of the ameloblast-extracellular matrix interface illustrating early dentin and enamel formation. ***Bottom:*** Montage of 35000x TLD images of the white box outlined above. The first enamel ribbons are observed to extend from the collagen-associated dentin mineral to the ameloblast membrane.

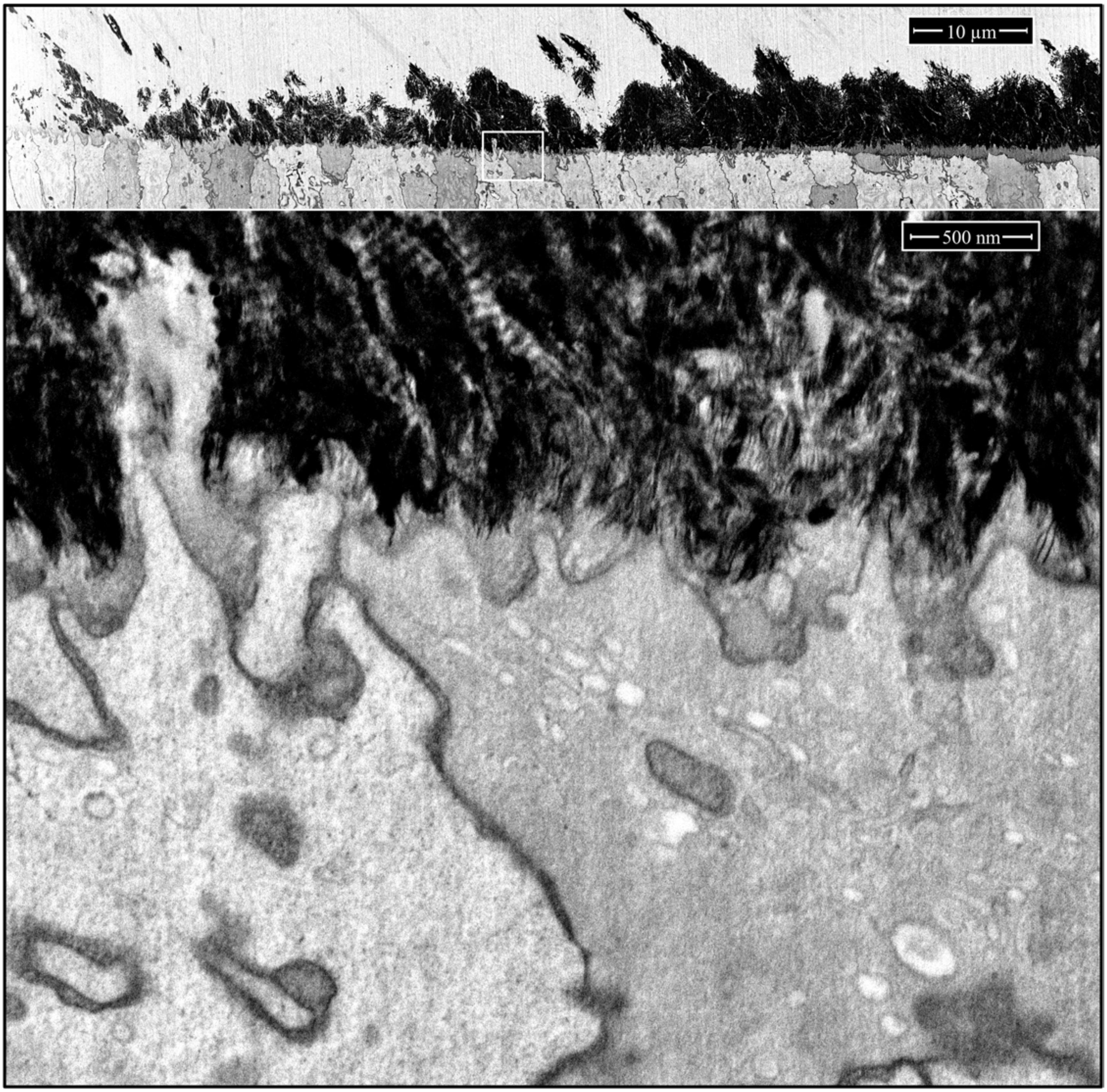

Figure S55. 7-week wild-type mandibular incisor FIB-SEM ~1 mm from its basal end (box 11). ***Top:*** Montage of 10000x TLD images of the ameloblast-extracellular matrix interface illustrating early dentin and enamel formation. ***Bottom:*** Montage of 35000x TLD images of the white box outlined above. The first enamel ribbons are observed to extend from the collagen-associated dentin mineral to the ameloblast membrane.

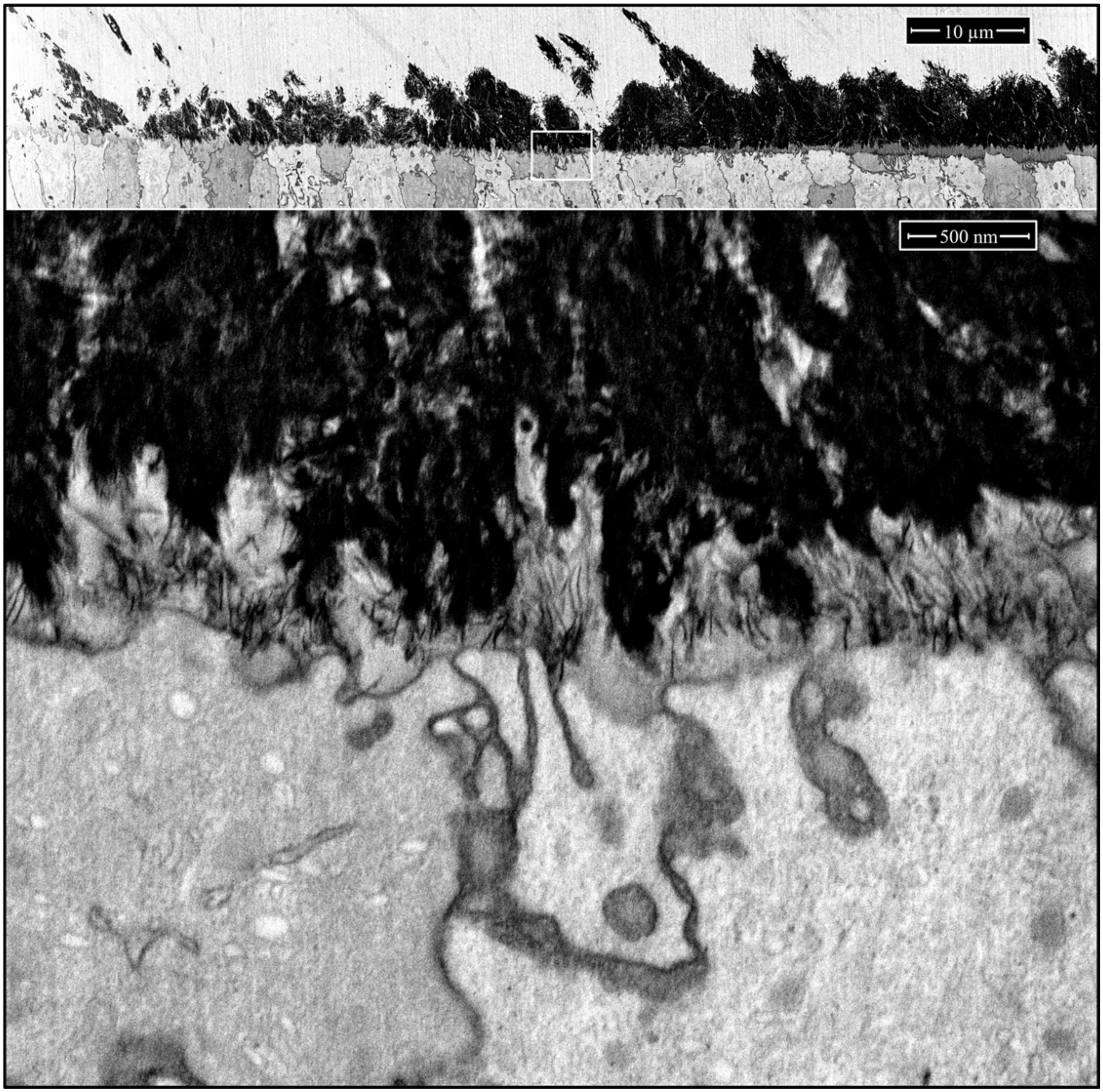

Figure S56. 7-week wild-type mandibular incisor FIB-SEM ~1 mm from its basal end (box 12). ***Top:*** Montage of 10000x TLD images of the ameloblast-extracellular matrix interface illustrating early dentin and enamel formation. ***Bottom:*** Montage of 35000x TLD images of the white box outlined above. The first enamel ribbons are observed to extend from the collagen-associated dentin mineral to the ameloblast membrane.

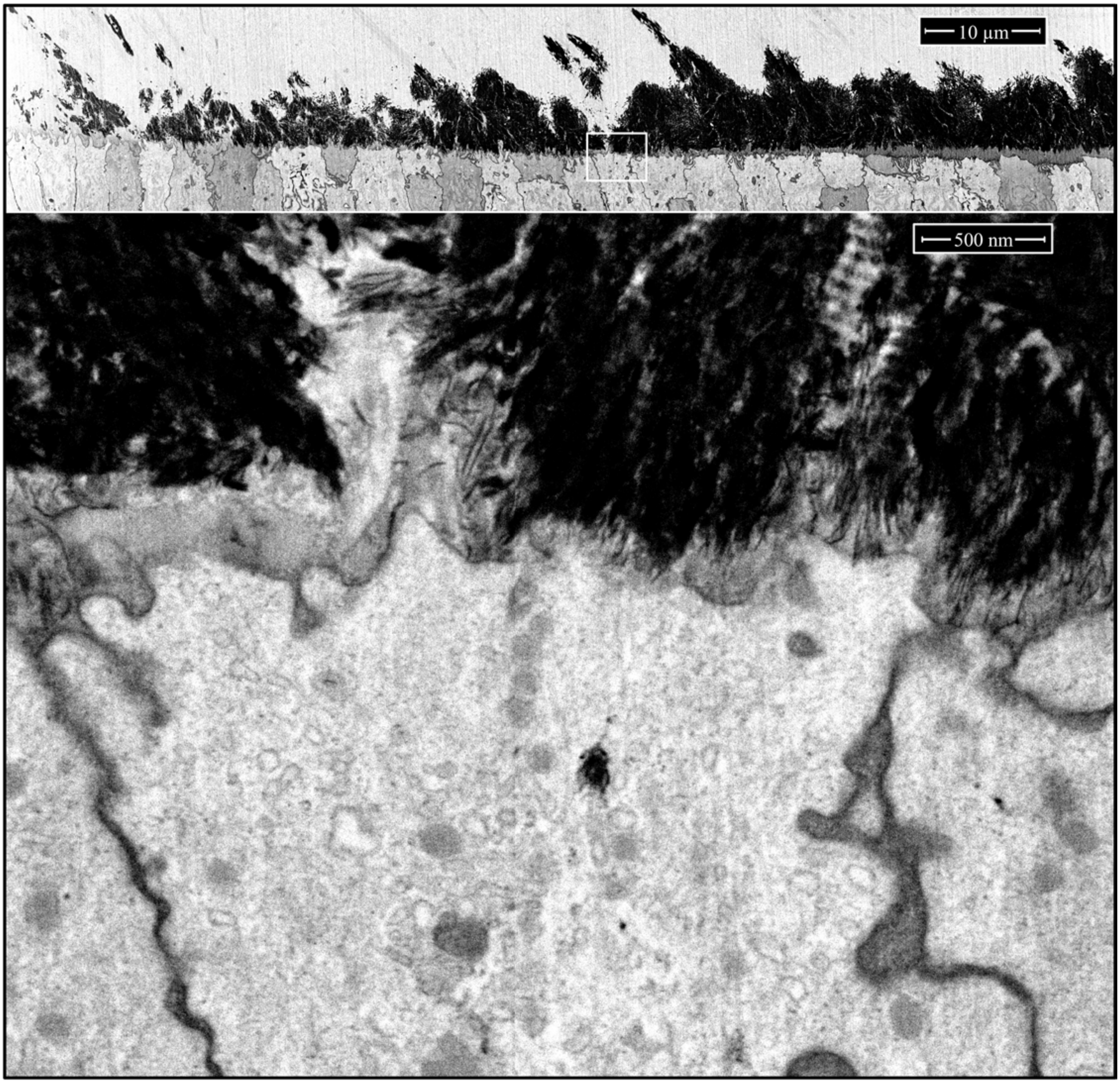

Figure S57. 7-week wild-type mandibular incisor FIB-SEM ~1 mm from its basal end (box 13). ***Top:*** Montage of 10000x TLD images of the ameloblast-extracellular matrix interface illustrating early dentin and enamel formation. ***Bottom:*** Montage of 35000x TLD images of the white box outlined above. The first enamel ribbons are observed to extend from the collagen-associated dentin mineral to the ameloblast membrane.

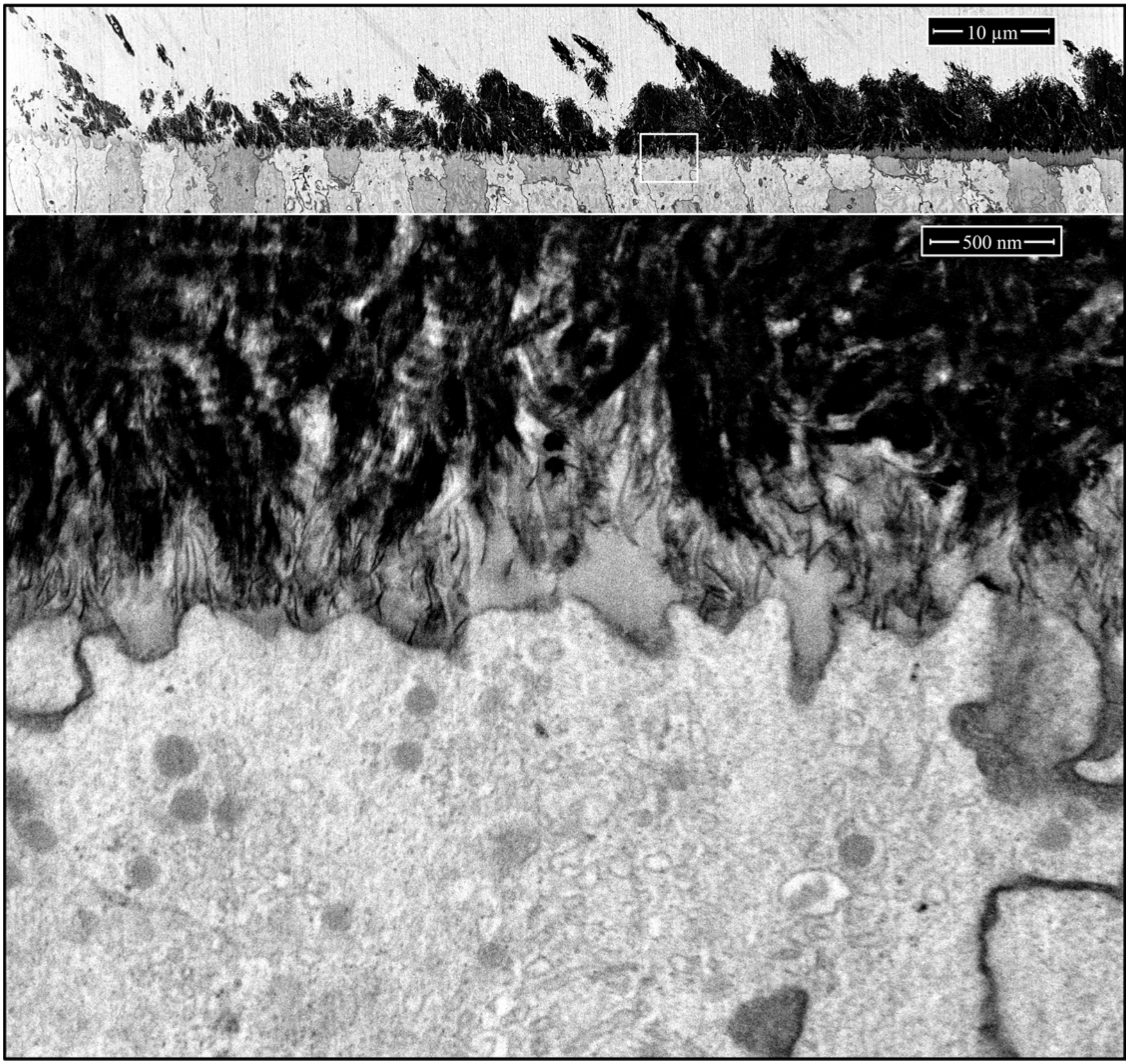

Figure S58. 7-week wild-type mandibular incisor FIB-SEM ~1 mm from its basal end (box 14). ***Top:*** Montage of 10000x TLD images of the ameloblast-extracellular matrix interface illustrating early dentin and enamel formation. ***Bottom:*** Montage of 35000x TLD images of the white box outlined above. The first enamel ribbons are observed to extend from the collagen-associated dentin mineral to the ameloblast membrane. Sometimes small patches of extracellular matrix, presumably amelogenin, localize along the enamel distal membrane and lack ribbons.

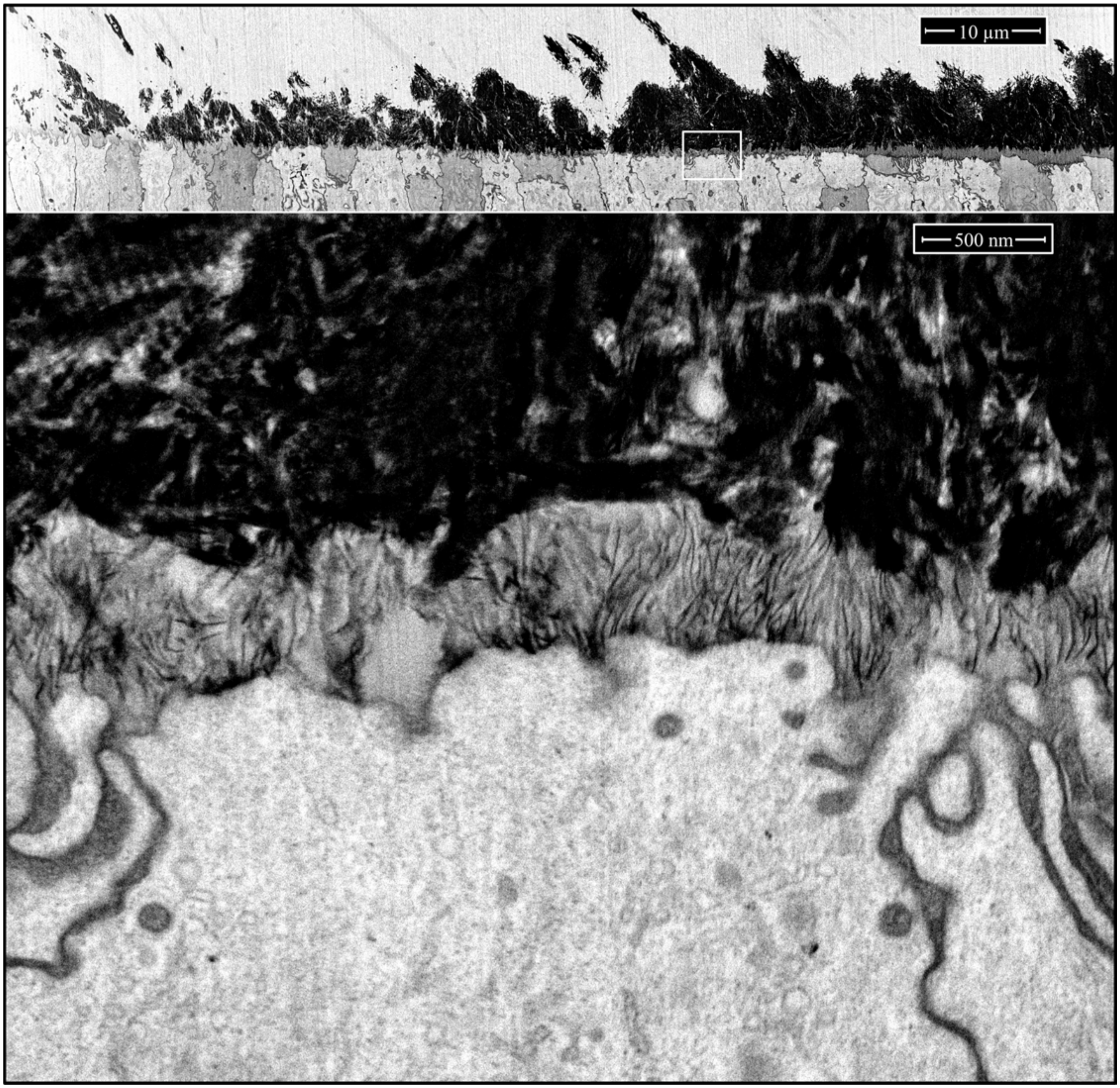

Figure S59. 7-week wild-type mandibular incisor FIB-SEM ~1 mm from its basal end (box 15). ***Top:*** Montage of 10000x TLD images of the ameloblast-extracellular matrix interface illustrating early dentin and enamel formation. ***Bottom:*** Montage of 35000x TLD images of the white box outlined above. The first enamel ribbons are observed to extend from the collagen-associated dentin mineral to the ameloblast membrane. Sometimes small patches of extracellular matrix, presumably amelogenin, localize along the enamel distal membrane and lack ribbons.

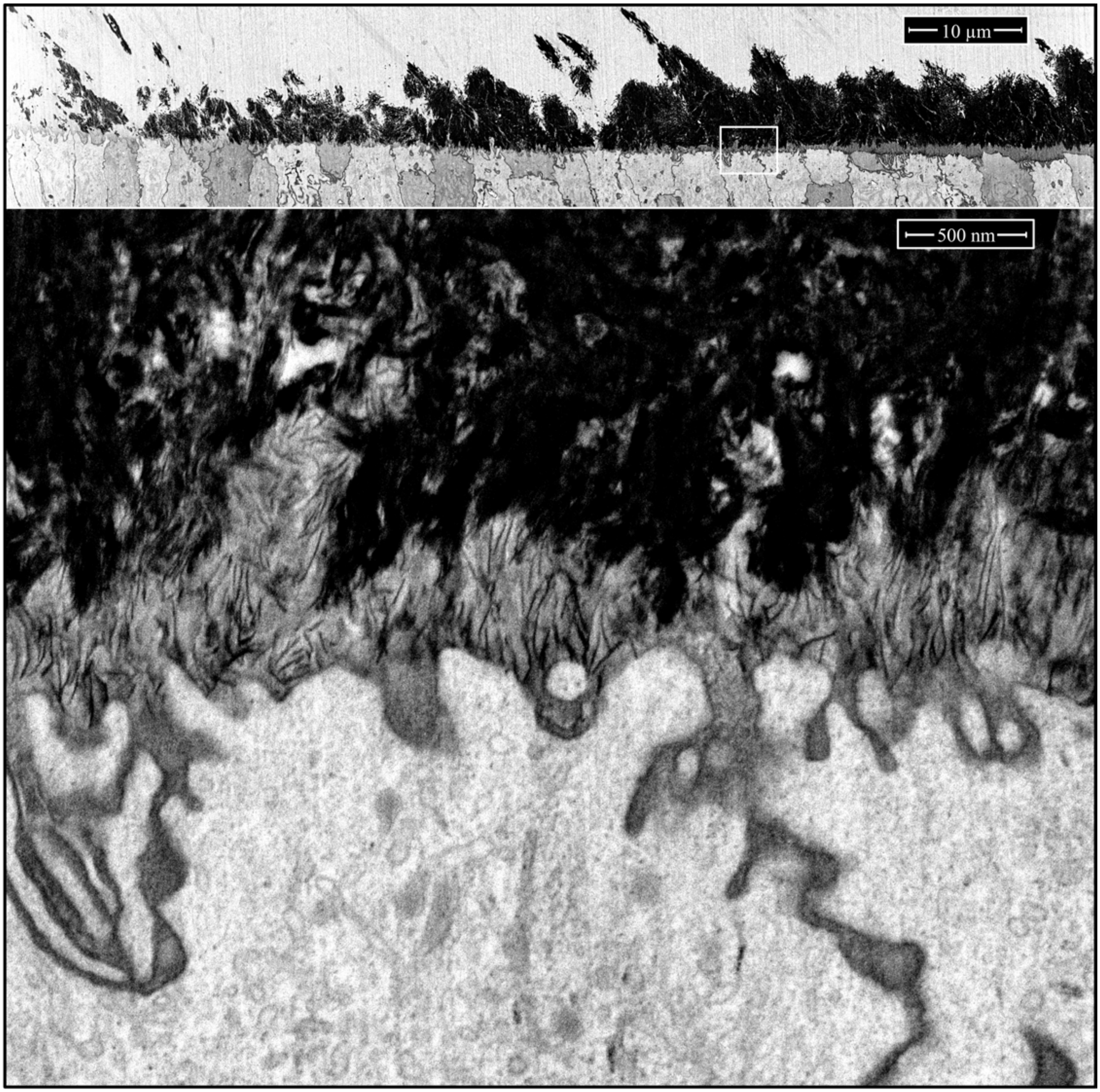

Figure S60. 7-week wild-type mandibular incisor FIB-SEM ~1 mm from its basal end (box 16). ***Top:*** Montage of 10000x TLD images of the ameloblast-extracellular matrix interface illustrating early dentin and enamel formation. ***Bottom:*** Montage of 35000x TLD images of the white box outlined above. The first enamel ribbons extend from the collagen-associated dentin mineral to the ameloblast membrane, which is not smooth, but has a very irregular membrane surface.

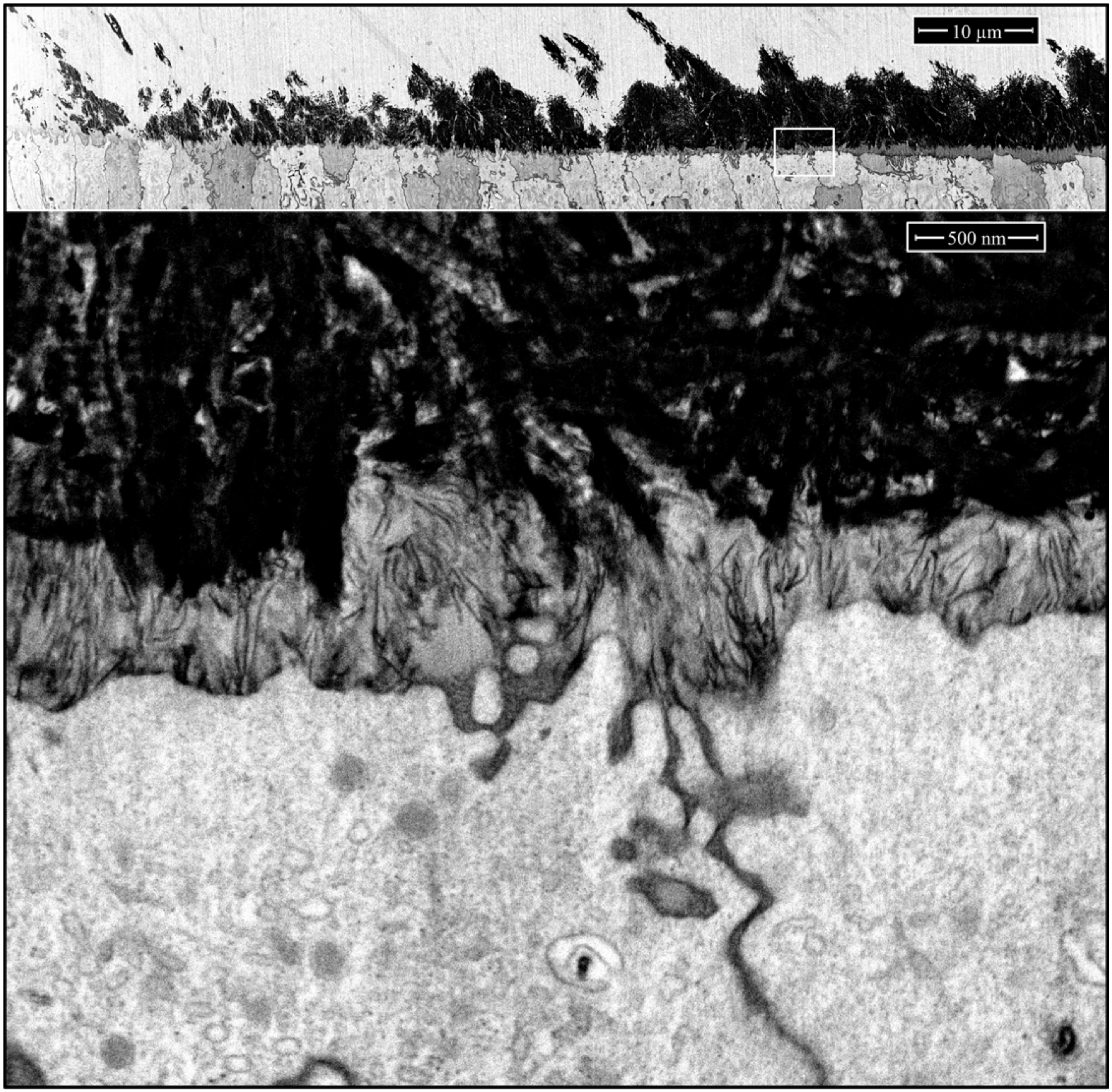

Figure S61. 7-week wild-type mandibular incisor FIB-SEM ~1 mm from its basal end (box 17). ***Top:*** Montage of 10000x TLD images of the ameloblast-extracellular matrix interface illustrating early dentin and enamel formation. ***Bottom:*** Montage of 35000x TLD images of the white box outlined above. The first enamel ribbons extend from the collagen-associated dentin mineral to the ameloblast membrane, which becomes less irregular and smoother as the initial enamel expands.

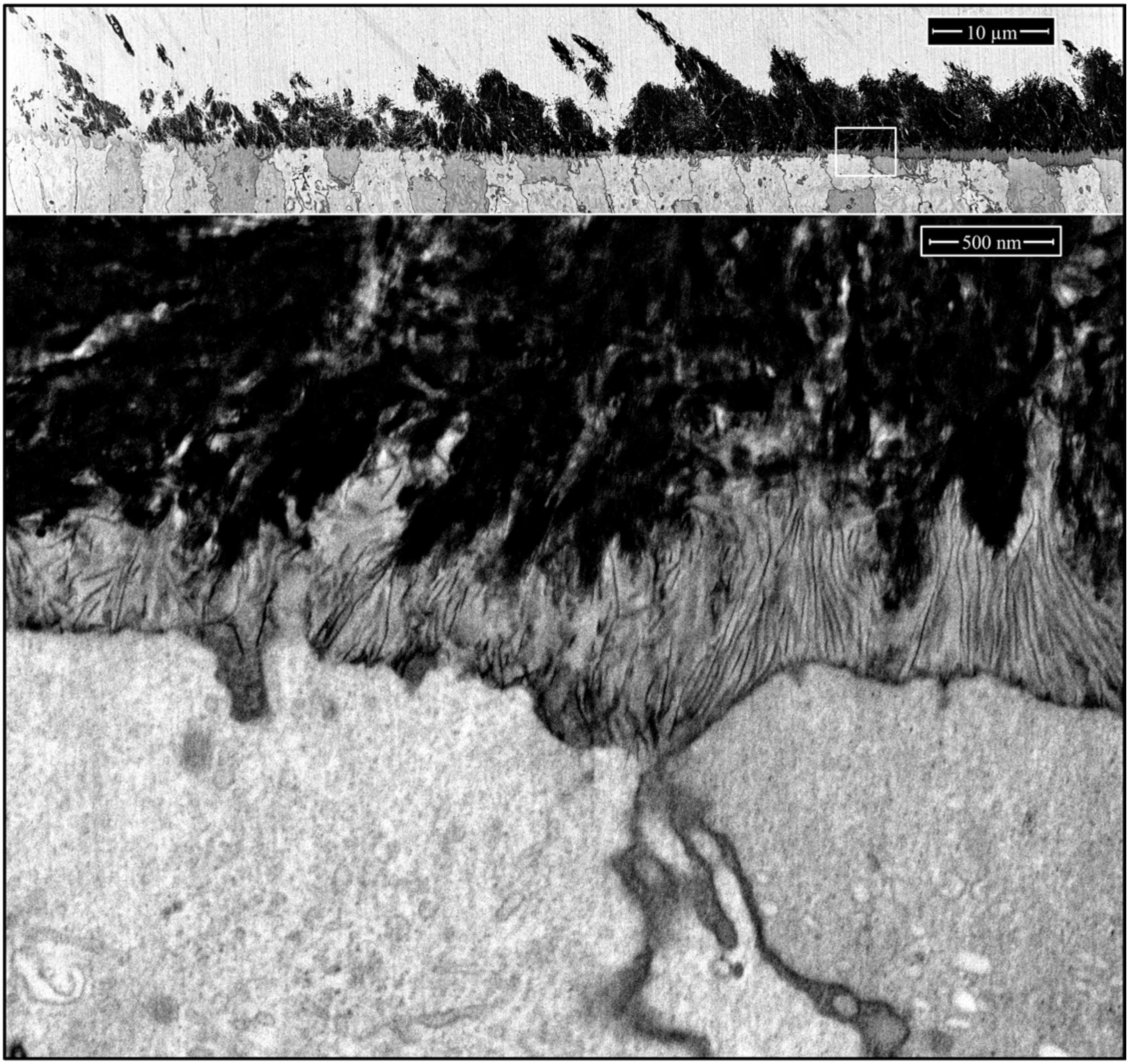

Figure S62. 7-week wild-type mandibular incisor FIB-SEM ~1 mm from its basal end (box 18). ***Top:*** Montage of 10000x TLD images of the ameloblast-extracellular matrix interface illustrating early dentin and enamel formation. ***Bottom:*** Montage of 35000x TLD images of the white box outlined above. The first enamel ribbons extend from the collagen-associated dentin mineral to the ameloblast membrane, which becomes less irregular and smoother as the initial enamel expands.

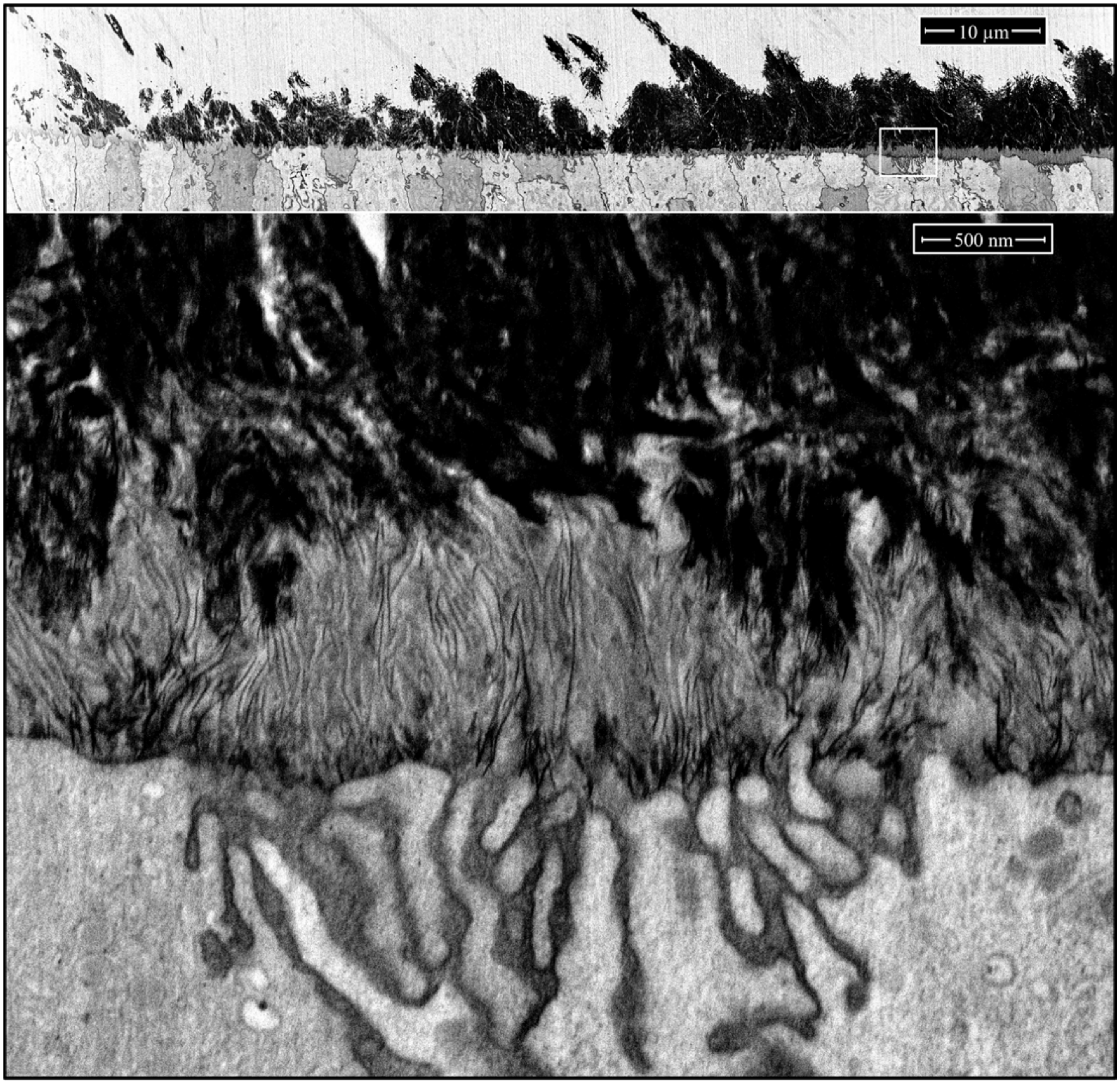

Figure S63. 7-week wild-type mandibular incisor FIB-SEM ~1 mm from its basal end (box 19). ***Top:*** Montage of 10000x TLD images of the ameloblast-extracellular matrix interface illustrating early dentin and enamel formation. ***Bottom:*** Montage of 35000x TLD images of the white box outlined above. The enamel ribbons extend from the collagen-associated dentin mineral to the ameloblast membrane, which is still highly convoluted at the ameloblast cell-cell junctions.

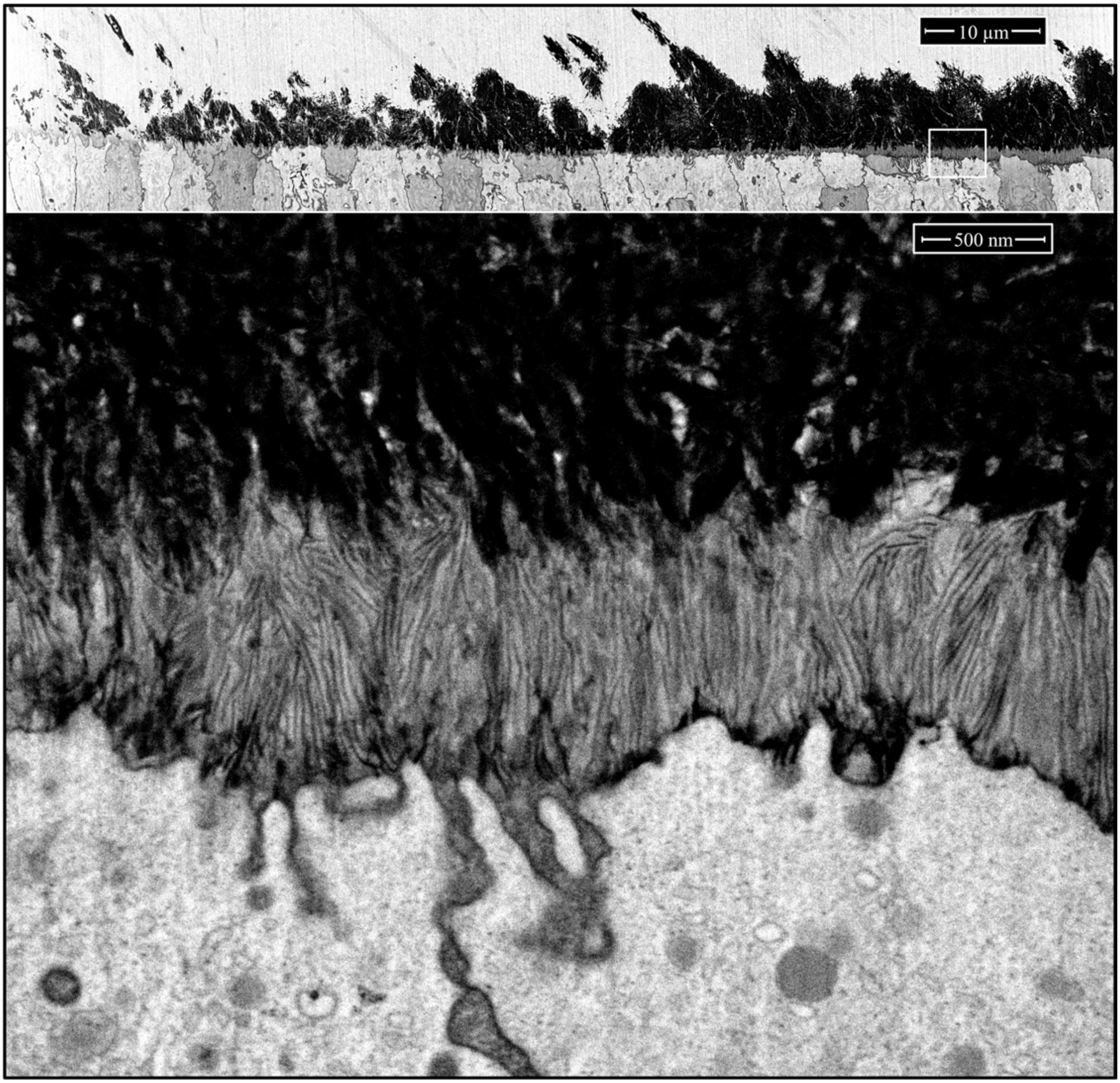

Figure S64. 7-week wild-type mandibular incisor FIB-SEM ~1 mm from its basal end (box 20). ***Top:*** Montage of 10000x TLD images of the ameloblast-extracellular matrix interface illustrating early dentin and enamel formation. ***Bottom:*** Montage of 35000x TLD images of the white box outlined above. Continued formation of the initial enamel.

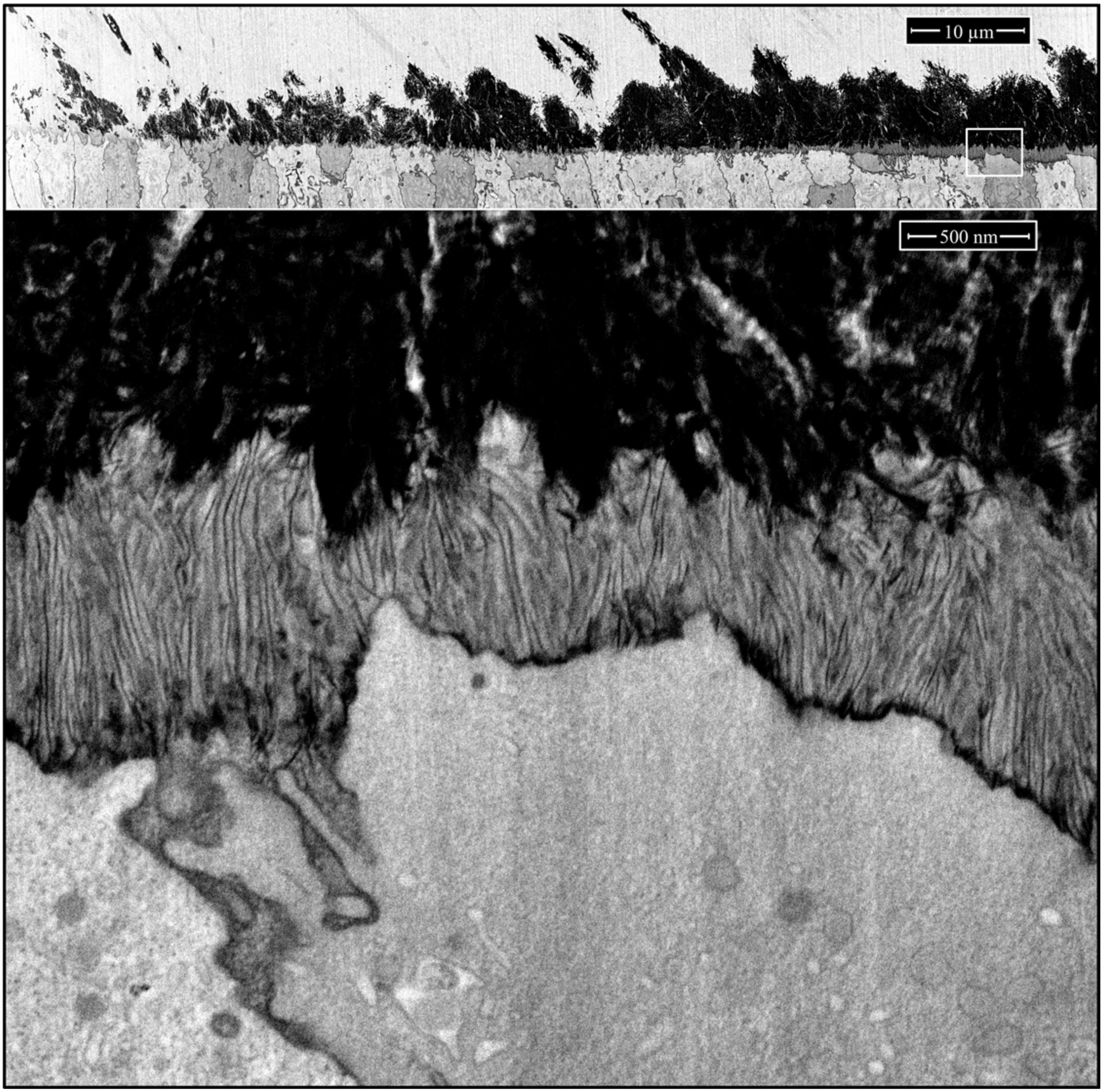

Figure S65. 7-week wild-type mandibular incisor FIB-SEM ~1 mm from its basal end (box 21). ***Top:*** Montage of 10000x TLD images of the ameloblast-extracellular matrix interface illustrating early dentin and enamel formation. ***Bottom:*** Montage of 35000x TLD images of the white box outlined above. Late during the formation of initial enamel, the rates of extension of the enamel ribbons along the ameloblast cell body becomes slower relative to their rate of extension along the ameloblast intercellular junctions, where prongs of interrod enamel extend that define the ameloblast Tomes' process.

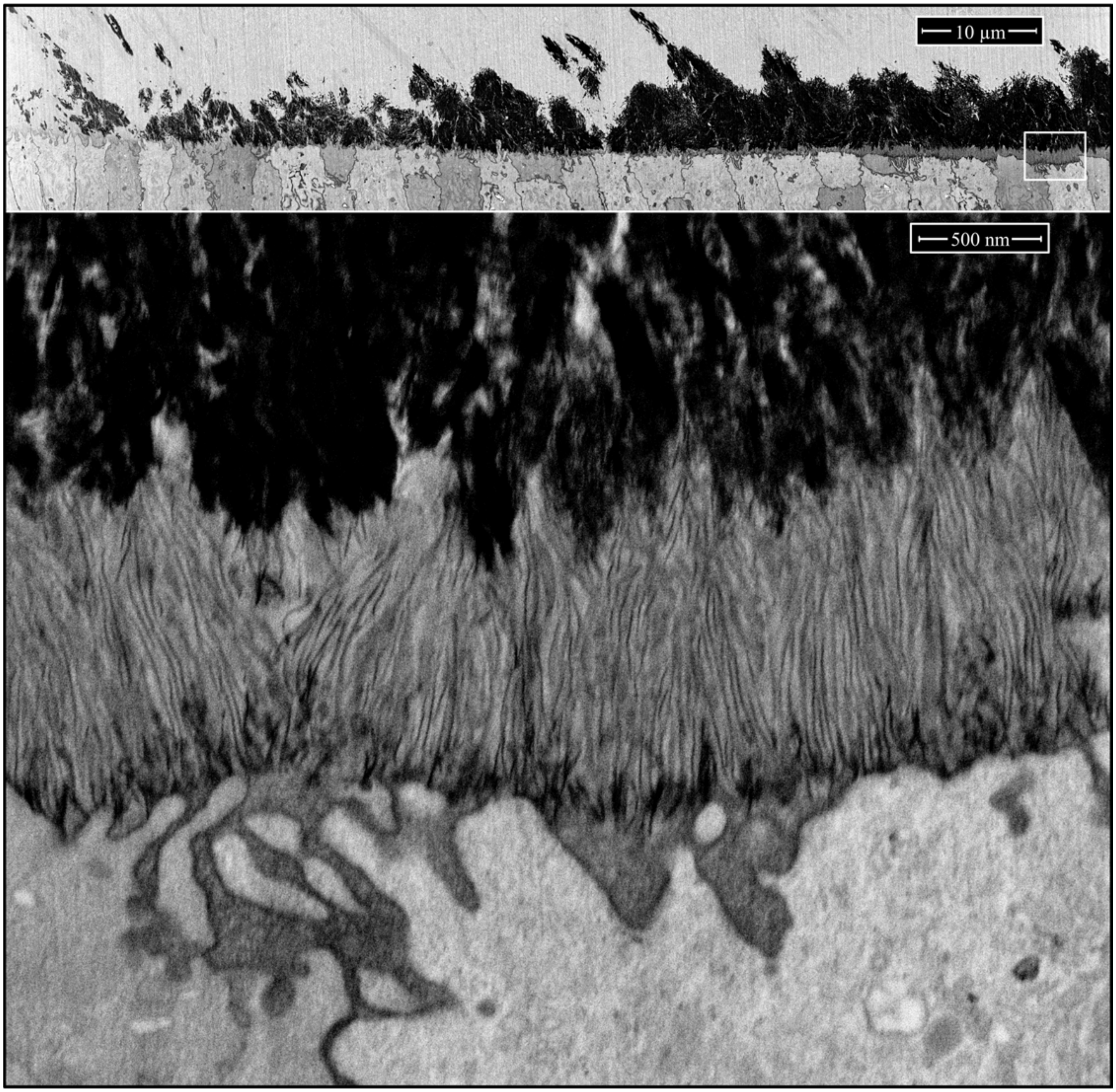

Figure S66. 7-week wild-type mandibular incisor FIB-SEM ~1 mm from its basal end (box 22). ***Top:*** Montage of 10000x TLD images of the ameloblast-extracellular matrix interface illustrating early dentin and enamel formation. ***Bottom:*** Montage of 35000x TLD images of the white box outlined above. The enamel ribbons extend from the dentin mineral to the ameloblast membrane, which is still highly convoluted at the ameloblast cell-cell junction.
