# Supplementary material for: AMBN mutations causing hypoplastic amelogenesis imperfecta and Ambn knockout‐NLS‐lacZ knockin mice exhibiting failed amelogenesis and Ambn tissue‐specificity
Source: Mol Genet Genomic Med. 2019 Aug 11;7(9):e929. doi: 10.1002/mgg3.929 (PMC6732285; doi:10.1002/mgg3.929)
Supplement: Supplementary file 5 [file MGG3-7-e929-s005.pdf]

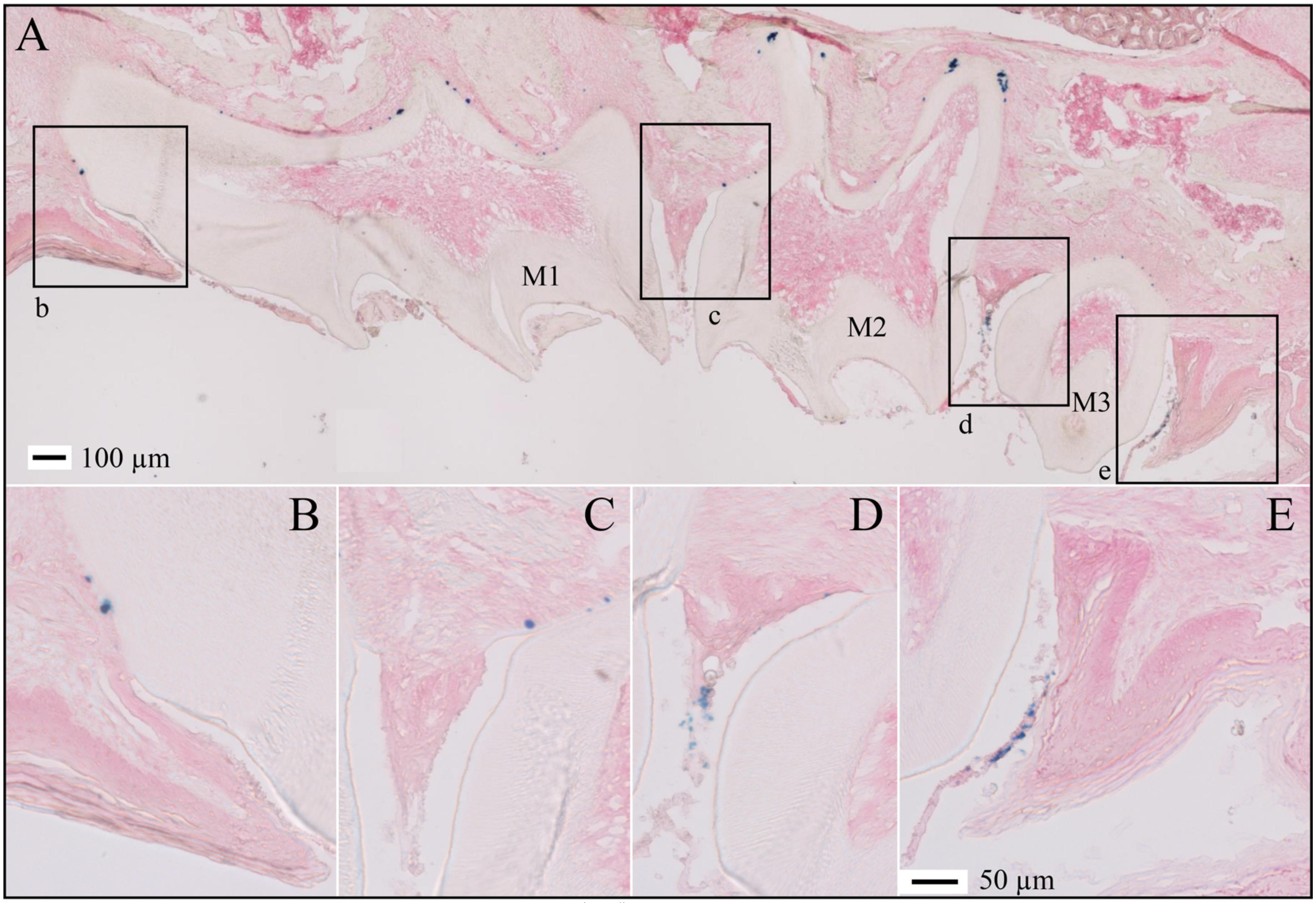

Figure S67. X-gal staining (15.5 h) of *Ambn*$^{lacZ/lacZ}$ molar junctional epithelium at D28. ***A:*** Decalcified section of maxillary first (M1), second (M2) and third (M3) molars. ***B-E:*** Higher magnification views of boxed areas in panel A. Only spotty X-gal staining was observed in the junctional epithelium.

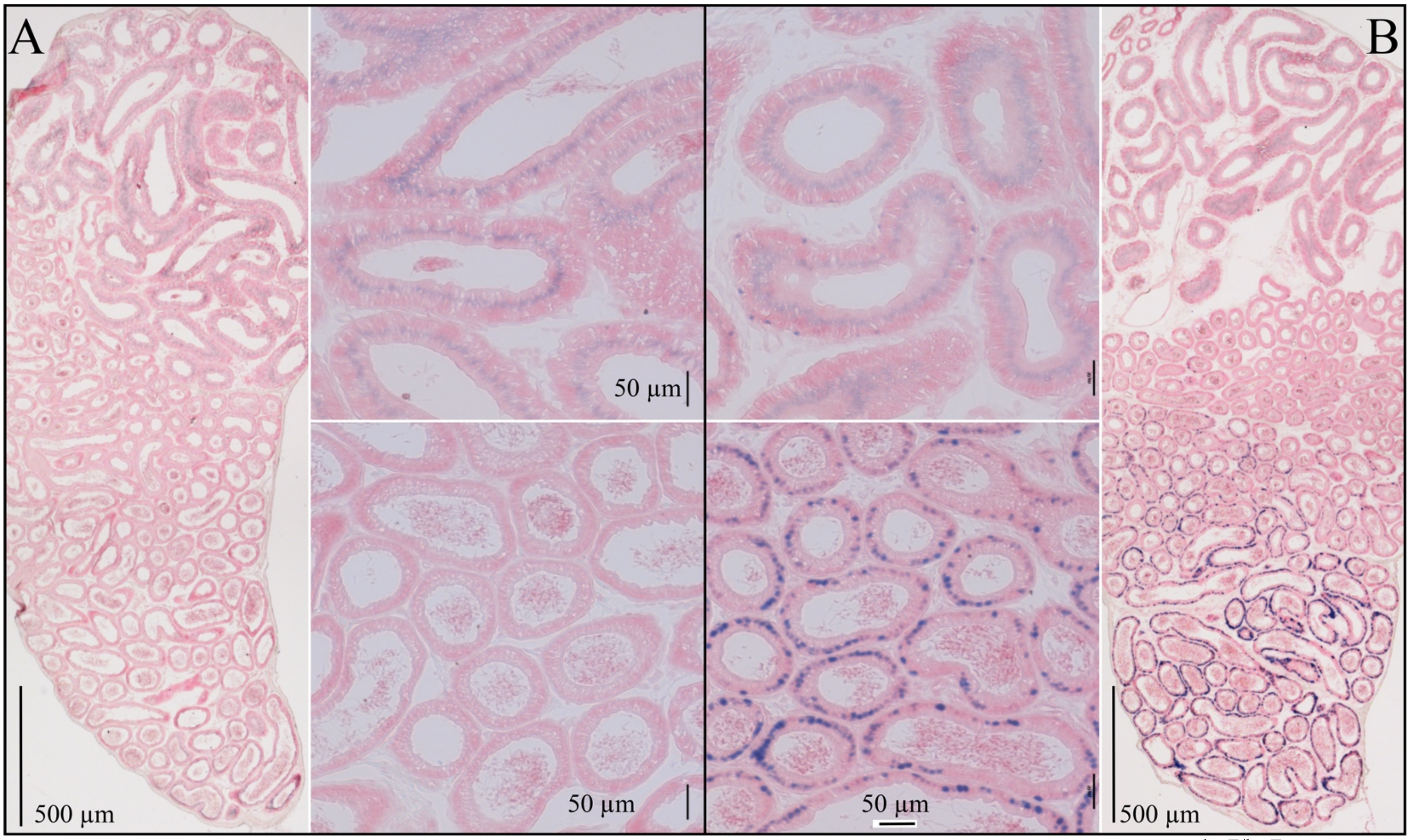

Figure S68. X-gal staining (1 h) of epididymis. ***A:*** Wild-type negative control. ***B:*** *Ambn*$^{lacZ/lacZ}$. The head of the epididymis (top) showed mild background, non-nuclear, X-gal staining that was present in both WT and *Ambn*$^{lacZ/lacZ}$ mice, and was not localized to the nucleus. The tail of the epididymis was positive for nuclear X-gal staining whereas the wild-type was negative.

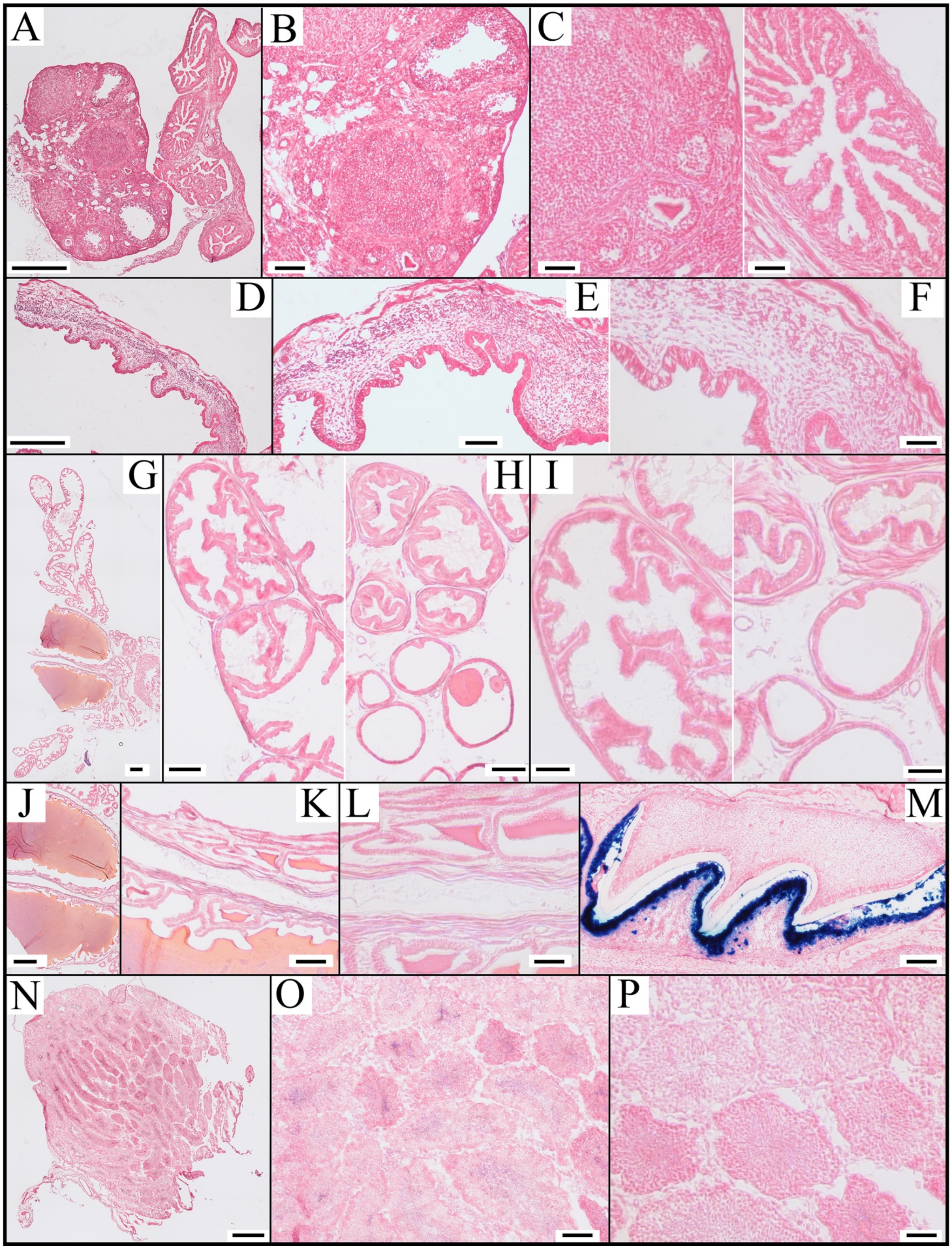

Figure S69. X-gal staining (15.5 h, overnight) of organs. All images are from 7-week-old *Ambn*$^{lacZ/lacZ}$ mice. Tissues were incubated in X-gal overnight to detect even trace expression. All organs tested here were negative, except the developing tooth (panel M). ***A-C:*** ovary and oviduct (***B:*** ovary only); ***D-F:*** uterus; ***G-I:*** prostate; ***J-L:*** seminal vesicle; ***M:*** Day 6 *Ambn*$^{lacZ/lacZ}$ maxillary first molar (positive control); ***N-P:*** testis. ***Scale Bars:*** A, D, G, J, N: 500 µm; B, E, H, K, M, O: 100 µm; C, F, I, L, P: 50 µm.

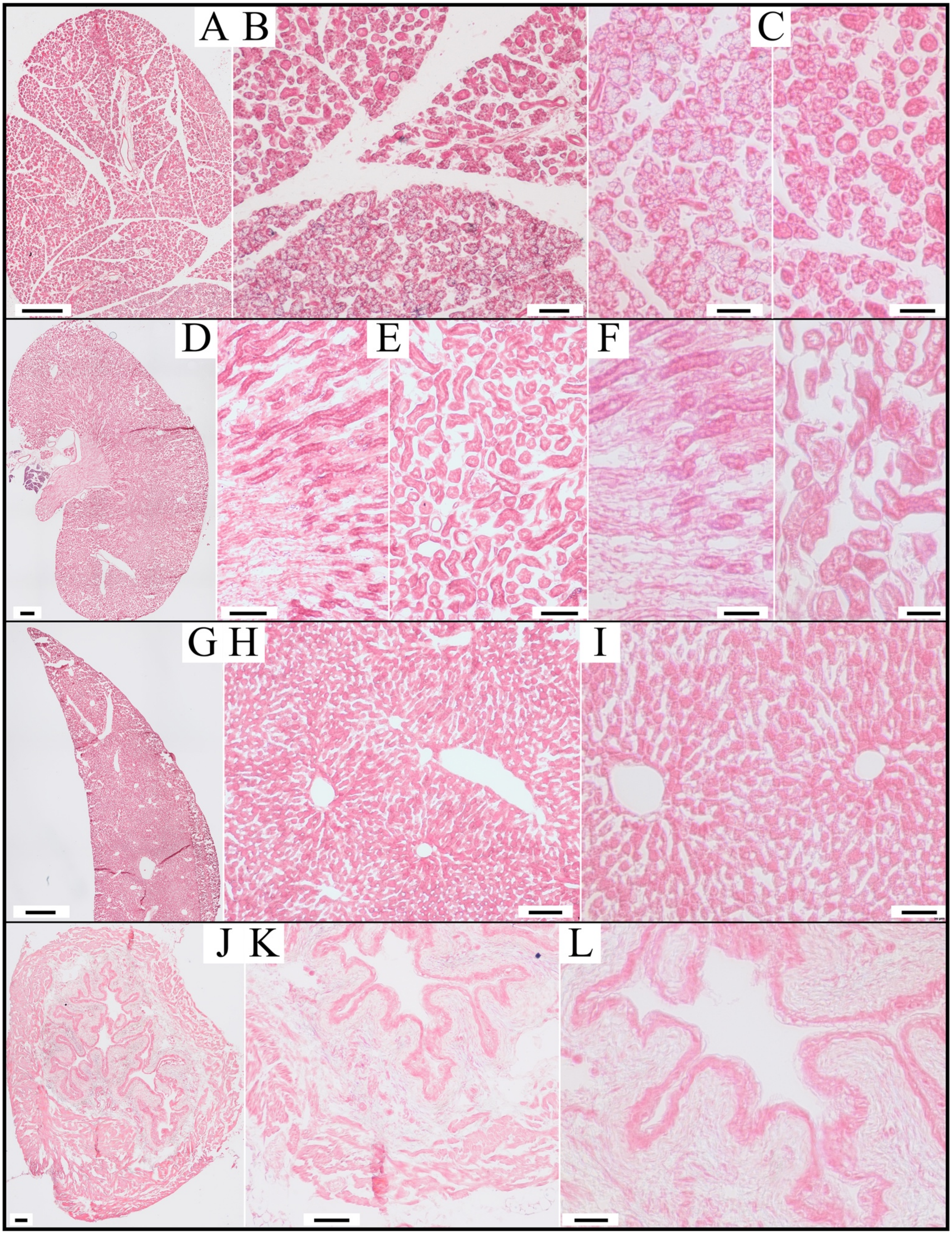

Figure S70. X-gal staining (15.5 h, overnight) of more body organs. All images are from 7-week-old *Ambn*$^{lacZ/lacZ}$ mice. Tissues were incubated in X-gal overnight to detect even trace expression. ***A-C:*** submandibular salivary gland; ***D-F:*** kidney; ***G-I:*** liver; ***J-L:*** bladder. ***Scale Bars:*** A, G, J: 500 µm; B, E, H, K: 100 µm; C, F, I, L: 50 µm.
